# Supplementary material for: Temporal trend in the national and sub-national burden of cancers attributable to risk factors in Iran from 1990 to 2021: Findings from the global burden of disease study 2021
Source: PLoS One. 2025 Aug 26;20(8):e0330993. doi: 10.1371/journal.pone.0330993 (PMC12380304; doi:10.1371/journal.pone.0330993)
Supplement: S1 Table — DALYs: Disability-Adjusted Life Years. YLDs: Years Lived with Disability. YLLs: Years of Life Lost. (DOCX) [file pone.0330993.s001.docx]

**Temporal trend in the national and sub-national burden of cancers attributable to risk factors in Iran from 1990 to 2021: findings from the Global Burden of Disease Study 2021**

Seyede Maryam Mousavi^1, 2^¶, Sobhan Younesian^1,2^¶, Saba Katebian^1^, Ali Golestani^1^, Shaghayegh Khanmohammadi^1,3^, Sepehr Khosravi^1^, Yasaman Etemadi^1^, Nazila Rezaei^1^, Sina Azadnajafabad^1*^, Bagher Larijani^4*^

**Authors’ affiliations:**

1. **Non-Communicable Diseases Research Center, Endocrinology and Metabolism Population Sciences Institute, Tehran University of Medical Sciences, Tehran, Iran**
2. **School of Medicine, Tehran University of Medical Sciences, Tehran, Iran**
3. **Research Center for Immunodeficiencies, Pediatrics Center of Excellence, Children’s Medical Center, Tehran University of Medical Sciences, Tehran, Iran**
4. **Endocrinology and Metabolism Research Center, Endocrinology and Metabolism Clinical Sciences Institute, Tehran University of Medical Sciences, Tehran, Iran**

***Corresponding authors:**

Sina Azadnajafabad (E-mail: [sina.azad.u@gmail.com](mailto:sina.azad.u@gmail.com))

Bagher Larijani (E-mail: [emrc@tums.ac.ir](mailto:emrc@tums.ac.ir))

¶ These authors contributed equally to this work.

Supplementary methods and results to “Temporal trend in the national and sub-national burden of cancers attributable to risk factors in Iran from 1990 to 2021: findings from the Global Burden of Disease Study 2021”

**S1 Table Title:** DALYs, deaths, YLDs, and YLLs of cancer attributable to level 2 risk factors by sex in Iran in 1990 and 2021 and their percent change.

| **Risk factors** | **Measure** | **Age, Metric** | **Year** | | | | | | **Percent Change (1990-2021)** | | |
| --- | --- | --- | --- | --- | --- | --- | --- | --- | --- | --- | --- |
|  |  |  | **1990** | | | **2021** | | |  |  |  |
|  |  |  | **Both** | **Female** | **Male** | **Both** | **Female** | **Male** | **Both** | **Female** | **Male** |
| Air pollution | DALYs | All age number | 16592.76 (10219.88 to 23651.68) | 3870.79 (2243.39 to 5797.46) | 12721.97 (8102.11 to 18524.07) | 43794.33 (27407.17 to 59853.8) | 13755.09 (8515.13 to 19500.52) | 30039.24 (18812.4 to 41140.9) | 163.94% (102.83% to 253.38%) | 255.36% (140.14% to 429.07%) | 136.12% (72.98% to 221.26%) |
|  |  | Age-standardized rate (per 100,000) | 58.76 (36.26 to 84.06) | 28.63 (16.62 to 43.22) | 86.67 (55.01 to 126.58) | 53.82 (33.55 to 73.38) | 33.72 (20.78 to 47.79) | 74.2 (46.49 to 101.68) | -8.4% (-29.83% to 23.05%) | 17.8% (-19.87% to 75.86%) | -14.39% (-37.36% to 16.15%) |
|  | Deaths | All age number | 590.85 (365.76 to 845.26) | 134.11 (77.84 to 202.7) | 456.74 (290.77 to 667.72) | 1723.99 (1079.87 to 2358.84) | 545.29 (332.23 to 772.64) | 1178.71 (739.24 to 1618.66) | 191.78% (122.85% to 291.92%) | 306.6% (177.92% to 507.49%) | 158.07% (88.61% to 249.45%) |
|  |  | Age-standardized rate (per 100,000) | 2.44 (1.51 to 3.52) | 1.19 (0.69 to 1.8) | 3.66 (2.32 to 5.36) | 2.33 (1.46 to 3.2) | 1.49 (0.91 to 2.12) | 3.18 (2.0 to 4.38) | -4.58% (-27.1% to 28.76%) | 24.88% (-13.58% to 87.52%) | -12.91% (-36.49% to 18.38%) |
|  | YLDs | All age number | 134.07 (76.08 to 213.52) | 31.56 (16.63 to 50.77) | 102.52 (58.55 to 161.18) | 376.87 (208.73 to 561.1) | 119.63 (66.32 to 183.79) | 257.24 (144.0 to 386.58) | 181.1% (114.43% to 281.19%) | 279.11% (158.66% to 465.96%) | 150.93% (80.58% to 247.48%) |
|  |  | Age-standardized rate (per 100,000) | 0.51 (0.29 to 0.81) | 0.25 (0.13 to 0.41) | 0.75 (0.43 to 1.19) | 0.48 (0.27 to 0.72) | 0.31 (0.17 to 0.47) | 0.66 (0.37 to 1.0) | -4.72% (-27.39% to 30.31%) | 21.47% (-16.94% to 82.35%) | -11.46% (-35.87% to 22.39%) |
|  | YLLs | All age number | 16458.69 (10147.35 to 23460.36) | 3839.24 (2225.01 to 5748.61) | 12619.46 (8028.11 to 18392.56) | 43417.46 (27162.97 to 59298.54) | 13635.46 (8444.9 to 19317.48) | 29782.0 (18654.95 to 40800.71) | 163.8% (102.78% to 253.31%) | 255.16% (140.04% to 428.71%) | 136.0% (72.93% to 221.0%) |
|  |  | Age-standardized rate (per 100,000) | 58.25 (35.94 to 83.32) | 28.38 (16.48 to 42.87) | 85.92 (54.56 to 125.53) | 53.34 (33.24 to 72.65) | 33.42 (20.55 to 47.32) | 73.54 (46.06 to 100.81) | -8.43% (-29.83% to 23.05%) | 17.76% (-19.9% to 75.79%) | -14.41% (-37.37% to 16.06%) |
| Alcohol use | DALYs | All age number | 1068.0 (821.06 to 1468.88) | 319.04 (237.37 to 434.4) | 748.96 (561.93 to 1045.33) | 7377.68 (6176.96 to 8874.84) | 1376.7 (1088.63 to 1714.47) | 6000.98 (4939.26 to 7269.05) | 590.8% (423.39% to 777.43%) | 331.52% (217.07% to 438.49%) | 701.24% (471.38% to 983.9%) |
|  |  | Age-standardized rate (per 100,000) | 3.95 (3.06 to 5.39) | 2.51 (1.87 to 3.43) | 5.25 (3.99 to 7.3) | 8.93 (7.51 to 10.73) | 3.4 (2.7 to 4.24) | 14.51 (12.01 to 17.6) | 126.42% (73.12% to 184.95%) | 35.73% (-0.38% to 66.63%) | 176.23% (98.35% to 262.84%) |
|  | Deaths | All age number | 41.06 (31.52 to 56.32) | 12.08 (9.0 to 16.73) | 28.98 (21.59 to 40.53) | 275.57 (230.3 to 329.83) | 54.62 (43.21 to 68.77) | 220.94 (182.93 to 265.7) | 571.05% (409.81% to 741.9%) | 351.99% (234.07% to 458.24%) | 662.4% (441.34% to 902.05%) |
|  |  | Age-standardized rate (per 100,000) | 0.17 (0.13 to 0.23) | 0.11 (0.08 to 0.15) | 0.23 (0.18 to 0.33) | 0.37 (0.3 to 0.44) | 0.15 (0.12 to 0.19) | 0.58 (0.49 to 0.7) | 109.58% (61.14% to 162.47%) | 33.7% (-1.91% to 63.08%) | 149.35% (79.37% to 222.12%) |
|  | YLDs | All age number | 8.81 (5.64 to 12.94) | 2.59 (1.7 to 3.85) | 6.22 (3.93 to 9.58) | 133.92 (93.22 to 184.44) | 29.95 (18.23 to 44.51) | 103.98 (71.42 to 145.24) | 1420.14% (955.32% to 2002.06%) | 1057.73% (650.12% to 1618.3%) | 1570.77% (1011.06% to 2298.56%) |
|  |  | Age-standardized rate (per 100,000) | 0.04 (0.02 to 0.05) | 0.02 (0.01 to 0.03) | 0.05 (0.03 to 0.07) | 0.16 (0.11 to 0.22) | 0.07 (0.04 to 0.1) | 0.25 (0.17 to 0.35) | 348.26% (215.63% to 510.63%) | 206.99% (103.02% to 351.05%) | 425.22% (260.14% to 638.69%) |
|  | YLLs | All age number | 1059.19 (814.89 to 1457.76) | 316.45 (235.8 to 430.73) | 742.74 (557.46 to 1039.14) | 7243.75 (6065.49 to 8710.2) | 1346.76 (1063.68 to 1679.51) | 5897.0 (4841.3 to 7155.29) | 583.9% (418.15% to 769.19%) | 325.58% (212.78% to 428.53%) | 693.96% (464.89% to 974.55%) |
|  |  | Age-standardized rate (per 100,000) | 3.91 (3.03 to 5.34) | 2.48 (1.86 to 3.4) | 5.21 (3.95 to 7.24) | 8.78 (7.34 to 10.57) | 3.33 (2.64 to 4.16) | 14.26 (11.78 to 17.29) | 124.41% (71.87% to 182.43%) | 34.19% (-1.58% to 64.69%) | 173.96% (96.51% to 259.19%) |
| Dietary risks | DALYs | All age number | 40667.94 (9759.88 to 91698.45) | 19725.2 (4827.65 to 39082.0) | 20942.74 (5560.75 to 53941.15) | 90945.01 (24742.56 to 178656.82) | 49810.01 (11913.91 to 90820.0) | 41135.01 (13550.2 to 92229.81) | 123.63% (70.31% to 195.66%) | 152.52% (73.21% to 244.28%) | 96.42% (55.65% to 167.46%) |
|  |  | Age-standardized rate (per 100,000) | 141.54 (35.06 to 318.23) | 140.75 (35.84 to 275.11) | 141.53 (38.96 to 365.01) | 107.98 (30.17 to 212.32) | 116.12 (28.88 to 209.6) | 100.36 (33.88 to 225.98) | -23.71% (-41.01% to -0.27%) | -17.5% (-42.39% to 10.96%) | -29.09% (-43.73% to -3.77%) |
|  | Deaths | All age number | 1361.18 (335.33 to 3102.75) | 633.95 (162.91 to 1240.8) | 727.23 (192.91 to 1894.27) | 3276.33 (949.97 to 6410.81) | 1698.9 (451.8 to 3024.53) | 1577.43 (536.18 to 3544.36) | 140.7% (87.68% to 217.55%) | 167.99% (100.31% to 258.8%) | 116.91% (69.03% to 200.01%) |
|  |  | Age-standardized rate (per 100,000) | 5.67 (1.48 to 12.64) | 5.47 (1.48 to 10.48) | 5.84 (1.64 to 14.95) | 4.36 (1.31 to 8.57) | 4.49 (1.25 to 7.91) | 4.26 (1.45 to 9.61) | -23.04% (-38.67% to -1.36%) | -17.94% (-37.95% to 7.41%) | -26.94% (-42.8% to -2.77%) |
|  | YLDs | All age number | 897.46 (218.46 to 1766.34) | 575.36 (99.85 to 1199.14) | 322.1 (119.26 to 674.4) | 4368.85 (819.7 to 8606.57) | 3268.19 (413.85 to 6813.15) | 1100.66 (416.74 to 2012.78) | 386.8% (190.82% to 524.19%) | 468.02% (235.14% to 631.41%) | 241.71% (149.64% to 369.42%) |
|  |  | Age-standardized rate (per 100,000) | 3.15 (0.81 to 6.1) | 4.03 (0.77 to 8.18) | 2.32 (0.9 to 4.82) | 5.0 (1.03 to 9.74) | 7.29 (1.0 to 14.96) | 2.75 (1.06 to 5.04) | 58.78% (-3.96% to 103.48%) | 80.6% (5.21% to 131.44%) | 18.42% (-13.59% to 61.34%) |
|  | YLLs | All age number | 39770.47 (9524.61 to 89808.19) | 19149.84 (4712.95 to 37939.28) | 20620.63 (5427.42 to 53219.17) | 86576.16 (23737.49 to 170245.45) | 46541.82 (11425.23 to 84712.49) | 40034.35 (13132.45 to 90630.63) | 117.69% (68.32% to 188.45%) | 143.04% (70.67% to 231.27%) | 94.15% (54.19% to 164.38%) |
|  |  | Age-standardized rate (per 100,000) | 138.39 (34.17 to 312.66) | 136.71 (35.02 to 268.68) | 139.21 (37.94 to 359.2) | 102.97 (28.93 to 203.4) | 108.84 (27.71 to 195.54) | 97.61 (32.67 to 221.48) | -25.59% (-42.0% to -2.99%) | -20.39% (-43.25% to 6.37%) | -29.88% (-44.11% to -4.98%) |
| Drug use | DALYs | All age number | 970.25 (615.14 to 1476.22) | 251.11 (136.25 to 454.28) | 719.14 (476.24 to 1083.67) | 5103.39 (3531.3 to 6914.06) | 1313.0 (724.44 to 2095.88) | 3790.38 (2746.59 to 5011.8) | 425.99% (302.07% to 593.77%) | 422.87% (269.58% to 590.26%) | 427.07% (290.19% to 610.25%) |
|  |  | Age-standardized rate (per 100,000) | 3.25 (2.0 to 4.96) | 1.76 (0.95 to 3.19) | 4.56 (2.92 to 6.93) | 6.54 (4.51 to 8.85) | 3.2 (1.76 to 5.17) | 9.93 (7.18 to 13.05) | 101.43% (53.16% to 168.6%) | 81.78% (26.59% to 142.87%) | 117.56% (60.45% to 192.8%) |
|  | Deaths | All age number | 33.26 (19.98 to 51.86) | 8.13 (4.37 to 14.83) | 25.14 (15.75 to 38.73) | 217.92 (149.38 to 294.05) | 50.8 (28.34 to 83.36) | 167.12 (118.36 to 219.28) | 555.15% (390.89% to 773.79%) | 525.16% (339.83% to 739.86%) | 564.85% (384.28% to 806.95%) |
|  |  | Age-standardized rate (per 100,000) | 0.12 (0.07 to 0.2) | 0.06 (0.03 to 0.11) | 0.18 (0.11 to 0.28) | 0.31 (0.21 to 0.41) | 0.14 (0.08 to 0.22) | 0.47 (0.33 to 0.62) | 148.3% (83.75% to 236.75%) | 110.97% (44.43% to 189.29%) | 169.8% (95.41% to 269.59%) |
|  | YLDs | All age number | 7.44 (4.21 to 12.16) | 1.84 (0.89 to 3.36) | 5.61 (3.22 to 9.12) | 46.74 (28.24 to 70.91) | 11.27 (5.77 to 20.57) | 35.47 (21.67 to 53.02) | 527.79% (375.66% to 739.34%) | 513.08% (326.6% to 720.56%) | 532.61% (364.4% to 761.76%) |
|  |  | Age-standardized rate (per 100,000) | 0.03 (0.01 to 0.04) | 0.01 (0.01 to 0.03) | 0.04 (0.02 to 0.06) | 0.06 (0.04 to 0.1) | 0.03 (0.01 to 0.05) | 0.1 (0.06 to 0.15) | 139.11% (79.0% to 223.85%) | 108.87% (43.43% to 186.41%) | 159.26% (87.93% to 254.47%) |
|  | YLLs | All age number | 962.81 (610.26 to 1465.55) | 249.28 (135.11 to 450.67) | 713.53 (473.34 to 1076.17) | 5056.65 (3498.95 to 6856.68) | 1301.74 (718.39 to 2079.52) | 3754.91 (2722.26 to 4973.17) | 425.2% (301.53% to 592.79%) | 422.21% (269.1% to 589.26%) | 426.24% (289.55% to 609.1%) |
|  |  | Age-standardized rate (per 100,000) | 3.22 (1.98 to 4.92) | 1.75 (0.94 to 3.17) | 4.53 (2.9 to 6.86) | 6.47 (4.47 to 8.76) | 3.17 (1.75 to 5.11) | 9.83 (7.1 to 12.95) | 101.12% (52.9% to 168.14%) | 81.56% (26.43% to 142.62%) | 117.21% (60.13% to 192.28%) |
| High body-mass index | DALYs | All age number | 13704.48 (7040.53 to 20049.33) | 8540.76 (4034.07 to 13078.92) | 5163.72 (2801.12 to 7645.0) | 74206.13 (31550.84 to 116395.61) | 43644.13 (18505.19 to 68299.14) | 30561.99 (13884.01 to 48356.55) | 441.47% (315.15% to 543.47%) | 411.01% (288.43% to 523.4%) | 491.86% (342.18% to 646.52%) |
|  |  | Age-standardized rate (per 100,000) | 44.64 (21.9 to 66.71) | 59.03 (26.26 to 91.33) | 31.51 (16.82 to 46.41) | 89.9 (37.18 to 142.28) | 107.31 (43.74 to 169.87) | 72.2 (32.56 to 114.81) | 101.37% (54.82% to 137.39%) | 81.78% (39.88% to 118.92%) | 129.16% (73.16% to 188.65%) |
|  | Deaths | All age number | 432.56 (206.43 to 651.69) | 273.3 (117.66 to 428.48) | 159.26 (84.28 to 235.48) | 2663.15 (1072.8 to 4241.56) | 1596.69 (627.74 to 2545.98) | 1066.46 (472.52 to 1695.92) | 515.68% (377.42% to 623.24%) | 484.24% (346.31% to 601.46%) | 569.63% (407.54% to 742.98%) |
|  |  | Age-standardized rate (per 100,000) | 1.66 (0.77 to 2.54) | 2.2 (0.93 to 3.48) | 1.14 (0.6 to 1.7) | 3.52 (1.4 to 5.64) | 4.26 (1.64 to 6.86) | 2.78 (1.23 to 4.43) | 112.89% (66.78% to 147.93%) | 94.08% (48.89% to 133.03%) | 144.19% (83.3% to 208.08%) |
|  | YLDs | All age number | 328.6 (145.44 to 539.75) | 242.68 (103.35 to 409.1) | 85.92 (42.95 to 139.38) | 3230.55 (1316.22 to 5465.14) | 2378.85 (876.36 to 4071.17) | 851.7 (395.04 to 1429.59) | 883.13% (669.93% to 1083.54%) | 880.25% (653.99% to 1133.48%) | 891.26% (666.41% to 1148.02%) |
|  |  | Age-standardized rate (per 100,000) | 1.15 (0.49 to 1.91) | 1.8 (0.71 to 3.11) | 0.56 (0.28 to 0.92) | 4.01 (1.51 to 6.85) | 5.95 (2.08 to 10.31) | 2.05 (0.93 to 3.43) | 249.88% (176.02% to 322.88%) | 230.65% (156.2% to 310.6%) | 262.38% (182.58% to 352.26%) |
|  | YLLs | All age number | 13375.88 (6888.71 to 19509.63) | 8298.08 (3938.69 to 12677.05) | 5077.8 (2757.79 to 7515.79) | 70975.58 (29996.02 to 111535.64) | 41265.28 (17444.82 to 64358.62) | 29710.3 (13479.25 to 47001.99) | 430.62% (306.38% to 530.55%) | 397.29% (276.87% to 506.23%) | 485.1% (336.98% to 639.73%) |
|  |  | Age-standardized rate (per 100,000) | 43.49 (21.4 to 64.91) | 57.23 (25.54 to 88.63) | 30.94 (16.51 to 45.59) | 85.88 (35.38 to 135.95) | 101.36 (41.26 to 159.96) | 70.16 (31.62 to 111.35) | 97.45% (51.42% to 132.58%) | 77.1% (35.54% to 113.76%) | 126.73% (71.2% to 185.48%) |
| High fasting plasma glucose | DALYs | All age number | 7036.1 (859.21 to 13746.67) | 3136.76 (305.01 to 6382.05) | 3899.34 (532.64 to 7495.0) | 45311.71 (4263.02 to 87728.16) | 22021.41 (902.7 to 43168.23) | 23290.3 (3355.3 to 44414.46) | 543.99% (430.69% to 656.73%) | 602.04% (377.31% to 772.14%) | 497.29% (398.64% to 627.55%) |
|  |  | Age-standardized rate (per 100,000) | 27.52 (3.45 to 53.39) | 25.56 (2.76 to 51.59) | 29.37 (3.91 to 56.54) | 58.73 (5.83 to 114.1) | 56.08 (3.0 to 109.16) | 61.6 (8.66 to 117.6) | 113.4% (77.83% to 148.66%) | 119.42% (53.31% to 173.2%) | 109.72% (75.14% to 154.59%) |
|  | Deaths | All age number | 274.68 (35.07 to 535.28) | 120.71 (14.06 to 242.16) | 153.97 (20.09 to 295.39) | 1952.64 (208.31 to 3779.01) | 915.91 (66.91 to 1769.06) | 1036.73 (141.27 to 1986.58) | 610.88% (499.7% to 731.9%) | 658.77% (467.21% to 844.16%) | 573.34% (462.56% to 711.75%) |
|  |  | Age-standardized rate (per 100,000) | 1.28 (0.17 to 2.46) | 1.17 (0.15 to 2.36) | 1.4 (0.18 to 2.72) | 2.76 (0.3 to 5.32) | 2.58 (0.21 to 4.98) | 2.96 (0.4 to 5.66) | 115.11% (80.9% to 150.23%) | 120.02% (64.54% to 172.84%) | 111.65% (76.42% to 153.99%) |
|  | YLDs | All age number | 161.46 (11.49 to 333.39) | 92.12 (-4.65 to 207.76) | 69.33 (13.67 to 135.87) | 1602.54 (-7.74 to 3379.17) | 1053.92 (-105.5 to 2299.02) | 548.62 (99.07 to 1075.61) | 892.55% (509.86% to 1077.76%) | 1044.03% (633.86% to 1367.15%) | 691.29% (547.57% to 875.33%) |
|  |  | Age-standardized rate (per 100,000) | 0.64 (0.05 to 1.31) | 0.73 (-0.02 to 1.63) | 0.56 (0.11 to 1.09) | 2.02 (0.02 to 4.24) | 2.54 (-0.21 to 5.52) | 1.49 (0.27 to 2.93) | 216.13% (89.38% to 267.79%) | 248.77% (109.08% to 349.17%) | 169.15% (118.56% to 226.75%) |
|  | YLLs | All age number | 6874.64 (853.27 to 13475.47) | 3044.63 (309.92 to 6180.54) | 3830.0 (519.18 to 7363.26) | 43709.17 (4270.76 to 84076.68) | 20967.49 (1008.19 to 40799.17) | 22741.68 (3258.16 to 43444.23) | 535.8% (426.12% to 643.35%) | 588.67% (383.52% to 757.04%) | 493.78% (395.3% to 622.88%) |
|  |  | Age-standardized rate (per 100,000) | 26.88 (3.41 to 52.28) | 24.83 (2.78 to 49.99) | 28.82 (3.8 to 55.48) | 56.71 (5.8 to 109.55) | 53.54 (3.21 to 103.83) | 60.11 (8.4 to 115.36) | 110.96% (76.07% to 146.37%) | 115.62% (54.29% to 168.49%) | 108.58% (74.14% to 153.53%) |
| Low physical activity | DALYs | All age number | 4034.41 (1996.35 to 6209.76) | 3034.5 (1333.11 to 4885.46) | 999.91 (521.16 to 1631.6) | 13637.66 (6328.05 to 20683.77) | 10355.35 (4448.99 to 16070.66) | 3282.3 (1716.68 to 5188.75) | 238.03% (156.71% to 359.8%) | 241.25% (146.41% to 385.43%) | 228.26% (85.89% to 448.86%) |
|  |  | Age-standardized rate (per 100,000) | 14.93 (7.65 to 22.93) | 22.59 (10.6 to 36.01) | 7.67 (4.05 to 12.65) | 16.7 (8.13 to 25.1) | 24.86 (11.2 to 38.33) | 8.61 (4.56 to 13.66) | 11.86% (-14.8% to 51.04%) | 10.03% (-21.55% to 55.11%) | 12.27% (-36.1% to 90.36%) |
|  | Deaths | All age number | 140.93 (72.58 to 220.0) | 102.24 (49.43 to 161.97) | 38.7 (19.47 to 64.49) | 520.71 (270.81 to 777.27) | 374.28 (181.16 to 576.14) | 146.43 (75.2 to 230.2) | 269.47% (180.25% to 402.33%) | 266.09% (162.47% to 417.52%) | 278.4% (110.69% to 540.76%) |
|  |  | Age-standardized rate (per 100,000) | 0.65 (0.35 to 0.98) | 0.93 (0.47 to 1.48) | 0.37 (0.2 to 0.63) | 0.71 (0.38 to 1.05) | 1.02 (0.51 to 1.55) | 0.42 (0.22 to 0.67) | 10.04% (-17.96% to 50.94%) | 9.1% (-22.72% to 58.96%) | 13.35% (-36.37% to 106.09%) |
|  | YLDs | All age number | 136.96 (55.13 to 238.7) | 111.58 (40.05 to 196.24) | 25.38 (12.3 to 46.25) | 861.08 (318.51 to 1486.19) | 731.81 (244.15 to 1281.94) | 129.27 (62.61 to 211.75) | 528.73% (373.51% to 723.58%) | 555.88% (375.88% to 804.87%) | 409.34% (184.52% to 770.31%) |
|  |  | Age-standardized rate (per 100,000) | 0.5 (0.21 to 0.85) | 0.81 (0.31 to 1.43) | 0.21 (0.1 to 0.37) | 1.01 (0.39 to 1.73) | 1.68 (0.59 to 2.91) | 0.35 (0.17 to 0.57) | 102.15% (52.11% to 164.48%) | 107.02% (50.59% to 184.42%) | 66.78% (-6.19% to 187.81%) |
|  | YLLs | All age number | 3897.46 (1933.2 to 6012.51) | 2922.93 (1296.53 to 4699.19) | 974.53 (506.54 to 1595.55) | 12776.58 (6014.57 to 19214.49) | 9623.54 (4213.82 to 14640.73) | 3153.04 (1650.6 to 4996.74) | 227.82% (148.47% to 345.34%) | 229.24% (136.65% to 366.83%) | 223.54% (83.81% to 441.05%) |
|  |  | Age-standardized rate (per 100,000) | 14.43 (7.39 to 22.14) | 21.78 (10.33 to 34.8) | 7.46 (3.93 to 12.31) | 15.69 (7.76 to 23.38) | 23.17 (10.55 to 35.58) | 8.26 (4.41 to 13.06) | 8.72% (-16.87% to 47.33%) | 6.41% (-24.14% to 49.87%) | 10.75% (-36.94% to 88.0%) |
| Occupational risks | DALYs | All age number | 5812.42 (4092.95 to 7989.66) | 356.94 (233.95 to 517.42) | 5455.47 (3860.19 to 7566.87) | 15177.37 (10669.54 to 20139.33) | 1437.76 (1010.13 to 1914.24) | 13739.61 (9572.89 to 18262.16) | 161.12% (107.26% to 229.64%) | 302.8% (187.52% to 473.24%) | 151.85% (96.07% to 220.23%) |
|  |  | Age-standardized rate (per 100,000) | 17.87 (12.6 to 24.63) | 2.1 (1.4 to 3.04) | 32.06 (22.74 to 44.39) | 17.16 (12.14 to 22.82) | 3.18 (2.24 to 4.23) | 31.17 (21.85 to 41.46) | -3.96% (-23.72% to 21.25%) | 51.36% (7.44% to 112.39%) | -2.76% (-24.13% to 23.8%) |
|  | Deaths | All age number | 178.22 (125.44 to 246.29) | 9.43 (6.32 to 13.64) | 168.78 (119.57 to 234.14) | 483.7 (344.21 to 641.4) | 42.55 (29.99 to 57.08) | 441.14 (313.3 to 585.61) | 171.41% (114.28% to 243.13%) | 351.05% (218.32% to 535.54%) | 161.37% (102.85% to 232.49%) |
|  |  | Age-standardized rate (per 100,000) | 0.6 (0.43 to 0.82) | 0.06 (0.04 to 0.09) | 1.08 (0.78 to 1.49) | 0.59 (0.42 to 0.77) | 0.1 (0.07 to 0.14) | 1.07 (0.77 to 1.42) | -1.83% (-22.38% to 24.06%) | 58.06% (12.33% to 121.19%) | -0.74% (-22.67% to 26.24%) |
|  | YLDs | All age number | 54.89 (34.96 to 81.56) | 3.24 (1.94 to 4.96) | 51.65 (32.87 to 77.51) | 164.27 (102.12 to 235.96) | 15.62 (9.7 to 23.57) | 148.66 (91.67 to 212.05) | 199.25% (144.63% to 267.16%) | 381.38% (251.32% to 538.75%) | 187.81% (131.85% to 257.37%) |
|  |  | Age-standardized rate (per 100,000) | 0.18 (0.11 to 0.26) | 0.02 (0.01 to 0.03) | 0.32 (0.2 to 0.47) | 0.19 (0.12 to 0.27) | 0.04 (0.02 to 0.05) | 0.35 (0.21 to 0.49) | 8.34% (-11.86% to 32.74%) | 70.08% (23.29% to 124.38%) | 9.74% (-12.04% to 36.0%) |
|  | YLLs | All age number | 5757.52 (4047.86 to 7921.79) | 353.7 (231.46 to 512.98) | 5403.82 (3818.68 to 7500.72) | 15013.1 (10536.43 to 19946.19) | 1422.15 (999.77 to 1895.27) | 13590.95 (9454.01 to 18103.5) | 160.76% (106.83% to 229.32%) | 302.08% (186.98% to 472.51%) | 151.51% (95.69% to 219.86%) |
|  |  | Age-standardized rate (per 100,000) | 17.69 (12.46 to 24.36) | 2.08 (1.39 to 3.01) | 31.74 (22.49 to 43.95) | 16.97 (11.98 to 22.6) | 3.15 (2.22 to 4.19) | 30.83 (21.57 to 41.01) | -4.08% (-23.83% to 21.08%) | 51.18% (7.2% to 112.24%) | -2.88% (-24.29% to 23.62%) |
| Other environmental risks | DALYs | All age number | 3758.19 (-1894.88 to 10250.32) | 885.12 (-478.8 to 2413.96) | 2873.07 (-1456.62 to 7874.43) | 9983.19 (-5023.79 to 26629.55) | 3118.88 (-1493.75 to 8313.13) | 6864.31 (-3514.06 to 18927.06) | 165.64% (110.17% to 251.61%) | 252.37% (134.92% to 400.0%) | 138.92% (78.98% to 220.34%) |
|  |  | Age-standardized rate (per 100,000) | 13.04 (-6.62 to 36.04) | 6.33 (-3.43 to 17.48) | 19.27 (-9.82 to 52.97) | 12.34 (-6.2 to 33.11) | 7.7 (-3.73 to 20.54) | 17.04 (-8.74 to 47.16) | -5.36% (-25.07% to 26.29%) | 21.75% (-17.81% to 72.92%) | -11.56% (-33.8% to 19.06%) |
|  | Deaths | All age number | 131.04 (-66.6 to 362.03) | 29.66 (-16.12 to 82.17) | 101.38 (-51.77 to 278.46) | 392.62 (-195.6 to 1056.57) | 123.1 (-60.04 to 329.36) | 269.52 (-138.35 to 752.31) | 199.61% (135.12% to 298.1%) | 315.0% (180.69% to 488.74%) | 165.84% (97.62% to 255.97%) |
|  |  | Age-standardized rate (per 100,000) | 0.54 (-0.28 to 1.51) | 0.26 (-0.14 to 0.72) | 0.81 (-0.41 to 2.23) | 0.53 (-0.27 to 1.43) | 0.34 (-0.17 to 0.9) | 0.73 (-0.38 to 2.05) | -0.89% (-22.2% to 32.35%) | 29.99% (-11.19% to 86.25%) | -9.56% (-32.65% to 20.79%) |
|  | YLDs | All age number | 29.71 (-15.77 to 86.25) | 6.98 (-3.93 to 21.13) | 22.73 (-11.74 to 67.1) | 85.32 (-43.09 to 222.21) | 26.87 (-13.36 to 72.45) | 58.45 (-29.35 to 156.68) | 187.15% (123.1% to 282.31%) | 285.11% (158.26% to 453.56%) | 157.09% (90.45% to 245.62%) |
|  |  | Age-standardized rate (per 100,000) | 0.11 (-0.06 to 0.32) | 0.06 (-0.03 to 0.17) | 0.16 (-0.08 to 0.48) | 0.11 (-0.06 to 0.29) | 0.07 (-0.03 to 0.19) | 0.15 (-0.08 to 0.41) | -1.23% (-23.28% to 32.17%) | 26.17% (-15.06% to 82.66%) | -8.21% (-32.13% to 23.39%) |
|  | YLLs | All age number | 3728.47 (-1879.32 to 10160.66) | 878.14 (-475.13 to 2395.92) | 2850.34 (-1444.94 to 7807.83) | 9897.87 (-4969.67 to 26396.67) | 3092.01 (-1480.33 to 8248.17) | 6805.86 (-3481.98 to 18782.86) | 165.47% (110.02% to 251.31%) | 252.11% (134.71% to 399.59%) | 138.77% (78.92% to 220.24%) |
|  |  | Age-standardized rate (per 100,000) | 12.93 (-6.56 to 35.7) | 6.27 (-3.4 to 17.32) | 19.1 (-9.74 to 52.49) | 12.23 (-6.13 to 32.81) | 7.63 (-3.69 to 20.34) | 16.89 (-8.66 to 46.79) | -5.4% (-25.11% to 26.28%) | 21.72% (-17.82% to 72.85%) | -11.59% (-33.8% to 19.0%) |
| Tobacco | DALYs | All age number | 89361.12 (71651.72 to 111121.74) | 8325.1 (5442.46 to 11733.26) | 81036.02 (64998.05 to 100754.58) | 204874.04 (167242.91 to 245862.04) | 23288.06 (13520.71 to 33026.99) | 181585.98 (152150.4 to 214025.69) | 129.27% (93.71% to 171.25%) | 179.73% (101.55% to 268.53%) | 124.08% (85.67% to 166.78%) |
|  |  | Age-standardized rate (per 100,000) | 313.29 (249.76 to 391.01) | 59.03 (39.18 to 82.27) | 549.17 (439.81 to 687.19) | 251.54 (204.69 to 301.67) | 54.91 (32.54 to 77.55) | 450.47 (376.82 to 533.21) | -19.71% (-32.36% to -5.03%) | -6.98% (-32.65% to 22.57%) | -17.97% (-31.92% to -2.45%) |
|  | Deaths | All age number | 3177.78 (2525.7 to 3978.79) | 273.33 (181.81 to 383.77) | 2904.45 (2309.97 to 3642.01) | 7930.29 (6475.06 to 9611.45) | 829.33 (508.32 to 1169.36) | 7100.96 (5886.26 to 8490.99) | 149.55% (109.32% to 197.21%) | 203.42% (118.22% to 308.39%) | 144.49% (102.38% to 192.63%) |
|  |  | Age-standardized rate (per 100,000) | 12.65 (9.97 to 15.87) | 2.21 (1.47 to 3.04) | 22.79 (18.0 to 28.65) | 10.63 (8.6 to 12.95) | 2.14 (1.31 to 3.05) | 19.17 (15.8 to 23.09) | -15.95% (-29.57% to -0.18%) | -2.78% (-29.22% to 31.02%) | -15.86% (-30.36% to 0.68%) |
|  | YLDs | All age number | 1204.31 (823.59 to 1692.7) | 137.75 (72.97 to 219.83) | 1066.56 (744.36 to 1481.29) | 4097.3 (2737.35 to 5776.93) | 635.98 (242.47 to 1099.98) | 3461.32 (2437.46 to 4788.13) | 240.22% (188.17% to 300.94%) | 361.68% (206.23% to 480.5%) | 224.53% (171.94% to 285.34%) |
|  |  | Age-standardized rate (per 100,000) | 4.35 (3.01 to 6.09) | 0.97 (0.53 to 1.54) | 7.52 (5.23 to 10.45) | 5.09 (3.43 to 7.15) | 1.43 (0.58 to 2.44) | 8.82 (6.2 to 12.14) | 16.94% (-0.61% to 37.03%) | 46.98% (-0.31% to 83.42%) | 17.22% (-1.22% to 38.36%) |
|  | YLLs | All age number | 88156.8 (70670.44 to 109593.98) | 8187.34 (5352.7 to 11512.8) | 79969.46 (64203.33 to 99518.06) | 200776.74 (164331.04 to 241433.42) | 22652.08 (13266.09 to 31956.66) | 178124.66 (149532.54 to 209821.46) | 127.75% (92.59% to 169.97%) | 176.67% (99.96% to 265.03%) | 122.74% (84.49% to 165.14%) |
|  |  | Age-standardized rate (per 100,000) | 308.94 (246.05 to 385.35) | 58.06 (38.6 to 81.07) | 541.65 (433.46 to 677.61) | 246.45 (200.84 to 295.81) | 53.48 (31.86 to 75.41) | 441.65 (369.86 to 523.2) | -20.23% (-32.87% to -5.59%) | -7.89% (-33.05% to 21.66%) | -18.46% (-32.33% to -2.95%) |
| Unsafe sex | DALYs | All age number | 12829.14 (11042.21 to 15801.58) | 12829.14 (11042.21 to 15801.58) | NA | 17792.5 (15455.22 to 20145.64) | 17792.5 (15455.22 to 20145.64) | NA | 38.69% (6.32% to 66.66%) | 38.69% (6.32% to 66.66%) | NA |
|  |  | Age-standardized rate (per 100,000) | 39.16 (33.64 to 47.95) | 80.82 (69.44 to 98.95) | NA | 19.81 (17.19 to 22.39) | 39.78 (34.52 to 44.96) | NA | -49.41% (-60.73% to -39.87%) | -50.78% (-61.81% to -41.47%) | NA |
|  | Deaths | All age number | 354.0 (305.66 to 433.18) | 354.0 (305.66 to 433.18) | NA | 554.54 (476.9 to 628.23) | 554.54 (476.9 to 628.23) | NA | 56.65% (21.19% to 86.15%) | 56.65% (21.19% to 86.15%) | NA |
|  |  | Age-standardized rate (per 100,000) | 1.28 (1.11 to 1.56) | 2.61 (2.27 to 3.18) | NA | 0.68 (0.59 to 0.77) | 1.37 (1.18 to 1.55) | NA | -46.62% (-58.8% to -37.05%) | -47.41% (-59.42% to -37.99%) | NA |
|  | YLDs | All age number | 230.42 (158.71 to 315.44) | 230.42 (158.71 to 315.44) | NA | 459.89 (325.2 to 622.31) | 459.89 (325.2 to 622.31) | NA | 99.59% (53.02% to 150.0%) | 99.59% (53.02% to 150.0%) | NA |
|  |  | Age-standardized rate (per 100,000) | 0.68 (0.46 to 0.92) | 1.39 (0.95 to 1.89) | NA | 0.48 (0.35 to 0.65) | 0.98 (0.7 to 1.31) | NA | -28.24% (-44.72% to -11.76%) | -29.45% (-45.57% to -13.16%) | NA |
|  | YLLs | All age number | 12598.72 (10824.66 to 15535.6) | 12598.72 (10824.66 to 15535.6) | NA | 17332.61 (15025.86 to 19652.1) | 17332.61 (15025.86 to 19652.1) | NA | 37.57% (5.54% to 65.15%) | 37.57% (5.54% to 65.15%) | NA |
|  |  | Age-standardized rate (per 100,000) | 38.48 (33.15 to 47.15) | 79.44 (68.42 to 97.23) | NA | 19.33 (16.71 to 21.91) | 38.8 (33.56 to 43.96) | NA | -49.78% (-60.98% to -40.37%) | -51.15% (-62.06% to -41.94%) | NA |

**S1 Table Footnote:** DALYs: Disability-Adjusted Life Years. YLDs: Years Lived with Disability. YLLs: Years of Life Lost.
