# Supplementary material for: Temporal trend in the national and sub-national burden of cancers attributable to risk factors in Iran from 1990 to 2021: Findings from the global burden of disease study 2021
Source: PLoS One. 2025 Aug 26;20(8):e0330993. doi: 10.1371/journal.pone.0330993 (PMC12380304; doi:10.1371/journal.pone.0330993)
Supplement: S2 Table — GBD 2021 did not estimate any burden attributable to risk factors for the following 11 level 3 cancers: Brain and central nervous system cancer, Eye cancer, Hodgkin lymphoma, Malignant neoplasm of bone and articular cartilage, Malignant skin melanoma, Neuroblastoma and other peripheral nervous cell tumors, Non-melanoma skin cancer, Other malignant neoplasms, Other neoplasms, Soft tissue and other extraosseous sarcomas, and Testicular cancer. The risk-factor-attributable burden of cervical cancer, ovarian cancer, and uterine cancer was not estimated for males, and the risk-factor-attributable burden of prostate cancer was not estimated for females. DALYs: Disability-Adjusted Life Years. YLDs: Years Lived with Disability. YLLs: Years of Life Lost. (DOCX) [file pone.0330993.s002.docx]

**Temporal trend in the national and sub-national burden of cancers attributable to risk factors in Iran from 1990 to 2021: findings from the Global Burden of Disease Study 2021**

Seyede Maryam Mousavi^1, 2^¶, Sobhan Younesian^1,2^¶, Saba Katebian^1^, Ali Golestani^1^, Shaghayegh Khanmohammadi^1,3^, Sepehr Khosravi^1^, Yasaman Etemadi^1^, Nazila Rezaei^1^, Sina Azadnajafabad^1*^, Bagher Larijani^4*^

**Authors’ affiliations:**

1. **Non-Communicable Diseases Research Center, Endocrinology and Metabolism Population Sciences Institute, Tehran University of Medical Sciences, Tehran, Iran**
2. **School of Medicine, Tehran University of Medical Sciences, Tehran, Iran**
3. **Research Center for Immunodeficiencies, Pediatrics Center of Excellence, Children’s Medical Center, Tehran University of Medical Sciences, Tehran, Iran**
4. **Endocrinology and Metabolism Research Center, Endocrinology and Metabolism Clinical Sciences Institute, Tehran University of Medical Sciences, Tehran, Iran**

***Corresponding authors:**

Sina Azadnajafabad (E-mail: [sina.azad.u@gmail.com](mailto:sina.azad.u@gmail.com))

Bagher Larijani (E-mail: [emrc@tums.ac.ir](mailto:emrc@tums.ac.ir))

¶ These authors contributed equally to this work.

Supplementary methods and results to “Temporal trend in the national and sub-national burden of cancers attributable to risk factors in Iran from 1990 to 2021: findings from the Global Burden of Disease Study 2021”

**S2 Table Title:** DALYs, deaths, YLDs, and YLLs of neoplasms and level 3 cancers attributable risk factors among females, males, and both sexes in Iran for the years 1990, 2021, and their percent change.

| Risk factors | Measure | Age, Metric | Year | | | | | | Percent Change (1990-2021) | | |
| --- | --- | --- | --- | --- | --- | --- | --- | --- | --- | --- | --- |
|  |  |  | 1990 | | | 2021 | | |  |  |  |
|  |  |  | Both | Female | Male | Both | Female | Male | Both | Female | Male |
| Neoplasms | DALYs | All age number | 169057.47 (135238.74 to 218941.85) | 55645.71 (38161.28 to 74699.43) | 113411.76 (91220.05 to 149142.32) | 447268.61 (350568.82 to 550593.51) | 165709.97 (108823.27 to 218899.75) | 281558.64 (236594.8 to 340049.01) | 164.57% (124.58% to 207.05%) | 197.79% (138.06% to 259.31%) | 148.26% (103.2% to 194.34%) |
|  |  | Age-standardized rate (per 100,000) | 584.85 (467.81 to 762.74) | 387.69 (264.57 to 524.82) | 766.6 (619.83 to 1010.0) | 544.42 (427.39 to 669.64) | 395.53 (258.69 to 523.92) | 696.22 (585.81 to 842.78) | -6.91% (-21.36% to 8.15%) | 2.02% (-18.42% to 23.12%) | -9.18% (-25.19% to 7.75%) |
|  | Deaths | All age number | 5778.17 (4639.16 to 7583.0) | 1758.85 (1207.42 to 2375.57) | 4019.31 (3240.63 to 5312.21) | 16893.25 (13331.94 to 20914.49) | 5933.16 (3863.45 to 7801.84) | 10960.09 (9195.23 to 13182.79) | 192.36% (146.15% to 241.64%) | 237.33% (168.59% to 306.94%) | 172.69% (125.22% to 224.12%) |
|  |  | Age-standardized rate (per 100,000) | 23.34 (18.59 to 30.75) | 14.62 (9.93 to 19.96) | 31.81 (25.62 to 42.21) | 22.66 (17.9 to 28.14) | 15.76 (10.22 to 20.8) | 29.66 (24.84 to 35.87) | -2.94% (-18.34% to 12.71%) | 7.81% (-14.25% to 29.48%) | -6.77% (-23.14% to 10.58%) |
|  | YLDs | All age number | 2845.35 (1847.75 to 4070.69) | 1311.37 (681.54 to 2036.5) | 1533.98 (1069.24 to 2137.58) | 13559.52 (8146.46 to 20113.56) | 7782.84 (3716.82 to 12517.0) | 5776.69 (4051.35 to 7933.35) | 376.55% (286.23% to 462.01%) | 493.49% (359.83% to 619.06%) | 276.58% (214.66% to 346.92%) |
|  |  | Age-standardized rate (per 100,000) | 10.05 (6.61 to 14.41) | 9.16 (4.71 to 14.01) | 10.9 (7.64 to 15.19) | 16.3 (10.0 to 24.07) | 18.04 (8.56 to 28.91) | 14.62 (10.24 to 20.11) | 62.08% (30.17% to 90.13%) | 96.99% (53.01% to 139.18%) | 34.13% (11.69% to 58.62%) |
|  | YLLs | All age number | 166212.12 (133170.12 to 215972.6) | 54334.34 (37506.63 to 72761.98) | 111877.78 (90113.1 to 147301.29) | 433709.08 (341463.17 to 535885.13) | 157927.13 (104944.03 to 207852.0) | 275781.95 (232326.61 to 333612.37) | 160.94% (121.64% to 203.05%) | 190.66% (132.45% to 251.2%) | 146.5% (101.7% to 192.79%) |
|  |  | Age-standardized rate (per 100,000) | 574.8 (460.34 to 751.92) | 378.53 (259.51 to 511.17) | 755.69 (610.9 to 997.17) | 528.13 (416.41 to 652.31) | 377.49 (248.29 to 497.18) | 681.59 (573.3 to 826.37) | -8.12% (-22.28% to 6.74%) | -0.27% (-20.19% to 20.22%) | -9.81% (-25.76% to 7.1%) |
| Bladder cancer | DALYs | All age number | 3477.97 (2436.55 to 4655.38) | 203.8 (93.83 to 346.94) | 3274.17 (2325.89 to 4430.05) | 10392.02 (7234.22 to 13903.21) | 839.93 (182.58 to 1580.71) | 9552.09 (6769.1 to 12509.86) | 198.8% (116.8% to 308.52%) | 312.14% (69.71% to 471.55%) | 191.74% (109.16% to 308.27%) |
|  |  | Age-standardized rate (per 100,000) | 13.32 (9.07 to 17.81) | 1.83 (0.75 to 3.29) | 24.2 (16.91 to 32.69) | 13.73 (9.43 to 18.65) | 2.35 (0.46 to 4.49) | 25.25 (17.51 to 33.62) | 3.12% (-24.49% to 40.24%) | 28.15% (-43.63% to 73.66%) | 4.35% (-23.83% to 44.6%) |
|  | Deaths | All age number | 131.59 (90.04 to 176.37) | 8.71 (3.5 to 15.57) | 122.89 (85.6 to 166.12) | 448.16 (302.75 to 621.12) | 42.72 (7.25 to 83.37) | 405.45 (274.08 to 554.46) | 240.57% (145.29% to 370.77%) | 390.71% (98.11% to 572.47%) | 229.93% (134.64% to 370.49%) |
|  |  | Age-standardized rate (per 100,000) | 0.6 (0.39 to 0.81) | 0.09 (0.03 to 0.18) | 1.1 (0.74 to 1.48) | 0.65 (0.43 to 0.91) | 0.13 (0.02 to 0.26) | 1.16 (0.78 to 1.61) | 8.65% (-20.31% to 49.06%) | 37.66% (-41.58% to 84.02%) | 5.75% (-22.68% to 48.63%) |
|  | YLDs | All age number | 172.74 (108.0 to 262.31) | 6.99 (3.1 to 12.53) | 165.76 (103.7 to 252.61) | 864.61 (543.07 to 1288.48) | 42.26 (9.91 to 81.5) | 822.35 (515.86 to 1207.4) | 400.51% (261.04% to 583.96%) | 504.93% (167.17% to 749.04%) | 396.11% (257.77% to 589.5%) |
|  |  | Age-standardized rate (per 100,000) | 0.63 (0.39 to 0.94) | 0.06 (0.02 to 0.11) | 1.16 (0.73 to 1.73) | 1.11 (0.69 to 1.65) | 0.11 (0.02 to 0.23) | 2.12 (1.32 to 3.13) | 75.6% (29.05% to 136.5%) | 88.97% (-14.42% to 158.76%) | 82.75% (33.26% to 151.01%) |
|  | YLLs | All age number | 3305.23 (2322.7 to 4422.27) | 196.81 (90.22 to 335.28) | 3108.41 (2202.42 to 4181.29) | 9527.41 (6705.37 to 12847.33) | 797.67 (171.96 to 1491.57) | 8729.74 (6159.24 to 11502.07) | 188.25% (109.22% to 296.17%) | 305.3% (66.41% to 463.2%) | 180.84% (100.92% to 294.21%) |
|  |  | Age-standardized rate (per 100,000) | 12.69 (8.65 to 16.95) | 1.77 (0.72 to 3.18) | 23.04 (16.04 to 31.01) | 12.63 (8.71 to 17.26) | 2.23 (0.43 to 4.26) | 23.14 (15.94 to 31.01) | -0.48% (-26.77% to 35.71%) | 26.08% (-44.67% to 71.04%) | 0.41% (-26.66% to 39.88%) |
| Breast cancer | DALYs | All age number | 7275.35 (1095.14 to 12856.87) | 7214.01 (1098.41 to 12718.37) | 61.34 (0.33 to 141.22) | 35762.48 (7237.7 to 59023.07) | 35551.27 (7234.08 to 58615.4) | 211.2 (16.41 to 440.69) | 391.56% (269.02% to 638.23%) | 392.81% (268.06% to 637.48%) | 244.31% (78.97% to 1101.79%) |
|  |  | Age-standardized rate (per 100,000) | 23.18 (3.68 to 40.37) | 47.77 (7.82 to 83.0) | 0.38 (0.0 to 0.91) | 40.4 (8.09 to 66.41) | 80.46 (16.18 to 132.35) | 0.48 (0.03 to 1.01) | 74.3% (29.1% to 159.94%) | 68.44% (26.03% to 146.23%) | 25.64% (-35.72% to 330.95%) |
|  | Deaths | All age number | 202.1 (35.97 to 341.8) | 200.21 (35.76 to 338.79) | 1.9 (0.01 to 4.52) | 1052.87 (209.43 to 1727.86) | 1046.16 (208.88 to 1718.36) | 6.71 (0.43 to 14.48) | 420.95% (278.82% to 655.91%) | 422.53% (278.7% to 655.73%) | 254.11% (89.29% to 1010.09%) |
|  |  | Age-standardized rate (per 100,000) | 0.73 (0.14 to 1.23) | 1.49 (0.29 to 2.52) | 0.01 (0.0 to 0.03) | 1.29 (0.27 to 2.12) | 2.58 (0.53 to 4.22) | 0.02 (0.0 to 0.04) | 76.53% (28.53% to 155.15%) | 72.75% (25.85% to 147.04%) | 24.12% (-35.68% to 281.87%) |
|  | YLDs | All age number | 482.94 (73.11 to 941.05) | 480.52 (73.19 to 936.85) | 2.42 (0.01 to 5.74) | 3985.5 (797.27 to 7189.89) | 3971.38 (793.01 to 7164.71) | 14.12 (0.9 to 32.79) | 725.26% (529.72% to 1097.64%) | 726.48% (529.15% to 1097.14%) | 482.99% (223.64% to 1804.76%) |
|  |  | Age-standardized rate (per 100,000) | 1.59 (0.27 to 3.04) | 3.28 (0.58 to 6.26) | 0.02 (0.0 to 0.04) | 4.54 (0.96 to 8.09) | 9.04 (1.92 to 16.14) | 0.03 (0.0 to 0.08) | 185.3% (116.38% to 313.97%) | 175.34% (109.91% to 292.47%) | 105.67% (13.92% to 558.86%) |
|  | YLLs | All age number | 6792.41 (1015.25 to 12077.53) | 6733.49 (1018.41 to 11967.13) | 58.92 (0.31 to 136.11) | 31776.98 (6266.61 to 53166.49) | 31579.9 (6263.57 to 52843.88) | 197.08 (15.36 to 409.23) | 367.83% (250.51% to 605.2%) | 369.0% (250.19% to 604.24%) | 234.5% (73.27% to 1071.37%) |
|  |  | Age-standardized rate (per 100,000) | 21.59 (3.42 to 37.61) | 44.49 (7.29 to 77.3) | 0.37 (0.0 to 0.88) | 35.87 (7.06 to 59.72) | 71.42 (14.11 to 118.93) | 0.45 (0.03 to 0.93) | 66.12% (22.63% to 149.01%) | 60.55% (19.58% to 133.98%) | 22.09% (-37.58% to 321.12%) |
| Cervical cancer | DALYs | All age number | 12829.14 (11042.21 to 15801.58) | 12829.14 (11042.21 to 15801.58) | NA | 17792.5 (15455.22 to 20145.64) | 17792.5 (15455.22 to 20145.64) | NA | 38.69% (6.32% to 66.66%) | 38.69% (6.32% to 66.66%) | NA |
|  |  | Age-standardized rate (per 100,000) | 39.16 (33.64 to 47.95) | 80.82 (69.44 to 98.95) | NA | 19.81 (17.19 to 22.39) | 39.78 (34.52 to 44.96) | NA | -49.41% (-60.73% to -39.87%) | -50.78% (-61.81% to -41.47%) | NA |
|  | Deaths | All age number | 354.0 (305.66 to 433.18) | 354.0 (305.66 to 433.18) | NA | 554.54 (476.9 to 628.23) | 554.54 (476.9 to 628.23) | NA | 56.65% (21.19% to 86.15%) | 56.65% (21.19% to 86.15%) | NA |
|  |  | Age-standardized rate (per 100,000) | 1.28 (1.11 to 1.56) | 2.61 (2.27 to 3.18) | NA | 0.68 (0.59 to 0.77) | 1.37 (1.18 to 1.55) | NA | -46.62% (-58.8% to -37.05%) | -47.41% (-59.42% to -37.99%) | NA |
|  | YLDs | All age number | 230.42 (158.71 to 315.44) | 230.42 (158.71 to 315.44) | NA | 459.89 (325.2 to 622.31) | 459.89 (325.2 to 622.31) | NA | 99.59% (53.02% to 150.0%) | 99.59% (53.02% to 150.0%) | NA |
|  |  | Age-standardized rate (per 100,000) | 0.68 (0.46 to 0.92) | 1.39 (0.95 to 1.89) | NA | 0.48 (0.35 to 0.65) | 0.98 (0.7 to 1.31) | NA | -28.24% (-44.72% to -11.76%) | -29.45% (-45.57% to -13.16%) | NA |
|  | YLLs | All age number | 12598.72 (10824.66 to 15535.6) | 12598.72 (10824.66 to 15535.6) | NA | 17332.61 (15025.86 to 19652.1) | 17332.61 (15025.86 to 19652.1) | NA | 37.57% (5.54% to 65.15%) | 37.57% (5.54% to 65.15%) | NA |
|  |  | Age-standardized rate (per 100,000) | 38.48 (33.15 to 47.15) | 79.44 (68.42 to 97.23) | NA | 19.33 (16.71 to 21.91) | 38.8 (33.56 to 43.96) | NA | -49.78% (-60.98% to -40.37%) | -51.15% (-62.06% to -41.94%) | NA |
| Colon and rectum cancer | DALYs | All age number | 26005.57 (15632.41 to 35412.49) | 12491.13 (7362.81 to 17715.86) | 13514.44 (7952.77 to 19476.35) | 78371.78 (49527.95 to 100600.11) | 36232.28 (23695.26 to 46605.06) | 42139.5 (25466.45 to 55877.13) | 201.37% (143.6% to 297.41%) | 190.06% (121.7% to 308.36%) | 211.81% (137.35% to 309.41%) |
|  |  | Age-standardized rate (per 100,000) | 92.9 (56.49 to 125.6) | 92.97 (56.25 to 130.46) | 92.55 (54.43 to 132.56) | 96.08 (60.95 to 123.14) | 89.39 (59.1 to 114.87) | 102.99 (62.15 to 136.33) | 3.42% (-15.94% to 34.66%) | -3.85% (-26.13% to 33.03%) | 11.28% (-15.69% to 45.24%) |
|  | Deaths | All age number | 892.23 (542.08 to 1208.64) | 427.99 (261.55 to 600.48) | 464.25 (274.26 to 663.17) | 3023.46 (1926.8 to 3861.95) | 1416.27 (943.21 to 1818.59) | 1607.18 (972.27 to 2120.38) | 238.86% (175.52% to 339.53%) | 230.92% (154.37% to 354.73%) | 246.19% (163.06% to 357.31%) |
|  |  | Age-standardized rate (per 100,000) | 3.87 (2.38 to 5.22) | 3.88 (2.42 to 5.37) | 3.85 (2.24 to 5.42) | 4.13 (2.65 to 5.27) | 3.92 (2.61 to 5.05) | 4.35 (2.64 to 5.74) | 6.59% (-12.49% to 35.72%) | 1.09% (-21.58% to 35.74%) | 13.08% (-14.56% to 48.64%) |
|  | YLDs | All age number | 610.5 (355.65 to 937.87) | 286.73 (163.79 to 441.58) | 323.76 (177.11 to 519.4) | 3100.03 (1812.52 to 4643.8) | 1524.54 (898.57 to 2223.99) | 1575.49 (873.04 to 2409.16) | 407.79% (313.97% to 555.46%) | 431.69% (303.17% to 639.66%) | 386.62% (274.9% to 541.89%) |
|  |  | Age-standardized rate (per 100,000) | 2.27 (1.33 to 3.43) | 2.17 (1.25 to 3.29) | 2.37 (1.34 to 3.73) | 3.83 (2.25 to 5.7) | 3.68 (2.18 to 5.33) | 3.98 (2.22 to 6.08) | 68.55% (37.97% to 115.95%) | 69.39% (29.85% to 130.74%) | 68.16% (28.77% to 119.31%) |
|  | YLLs | All age number | 25395.07 (15223.37 to 34569.73) | 12204.4 (7173.89 to 17308.7) | 13190.67 (7761.34 to 19005.54) | 75271.74 (47505.31 to 96580.81) | 34707.73 (22607.82 to 44629.79) | 40564.01 (24565.1 to 53787.41) | 196.4% (139.38% to 290.29%) | 184.39% (117.82% to 301.14%) | 207.52% (133.95% to 304.68%) |
|  |  | Age-standardized rate (per 100,000) | 90.63 (54.96 to 122.86) | 90.8 (54.8 to 127.68) | 90.18 (53.05 to 128.96) | 92.25 (58.46 to 118.24) | 85.71 (56.46 to 110.21) | 99.0 (59.79 to 130.67) | 1.79% (-17.18% to 32.61%) | -5.6% (-27.45% to 30.72%) | 9.78% (-16.97% to 43.6%) |
| Esophageal cancer | DALYs | All age number | 13658.52 (7063.94 to 19426.96) | 3446.85 (192.88 to 6194.9) | 10211.67 (6683.8 to 13661.18) | 22334.27 (16306.15 to 29133.82) | 3014.97 (975.72 to 5442.29) | 19319.29 (14740.38 to 24316.94) | 63.52% (27.04% to 162.57%) | -12.53% (-46.25% to 66.61%) | 89.19% (45.64% to 155.16%) |
|  |  | Age-standardized rate (per 100,000) | 49.82 (25.66 to 70.95) | 26.51 (1.42 to 47.54) | 71.57 (47.16 to 95.99) | 28.59 (20.79 to 37.4) | 7.85 (2.38 to 14.31) | 49.57 (37.44 to 62.75) | -42.6% (-55.08% to -9.15%) | -70.38% (-81.65% to -44.94%) | -30.74% (-46.3% to -6.2%) |
|  | Deaths | All age number | 503.65 (266.18 to 712.16) | 125.67 (6.87 to 226.06) | 377.98 (251.5 to 502.42) | 967.24 (686.07 to 1277.55) | 137.4 (35.37 to 253.57) | 829.84 (619.09 to 1052.16) | 92.05% (52.45% to 191.44%) | 9.33% (-32.89% to 89.98%) | 119.55% (71.35% to 192.88%) |
|  |  | Age-standardized rate (per 100,000) | 2.15 (1.11 to 3.08) | 1.15 (0.06 to 2.07) | 3.14 (2.03 to 4.17) | 1.36 (0.95 to 1.8) | 0.4 (0.09 to 0.74) | 2.32 (1.71 to 2.95) | -36.91% (-49.58% to -3.68%) | -65.34% (-78.86% to -40.06%) | -26.05% (-41.72% to -0.86%) |
|  | YLDs | All age number | 134.37 (65.37 to 218.6) | 34.86 (1.86 to 67.65) | 99.52 (57.23 to 148.64) | 263.7 (172.52 to 383.21) | 40.34 (10.8 to 81.47) | 223.37 (147.8 to 316.56) | 96.25% (54.99% to 206.76%) | 15.73% (-29.97% to 123.06%) | 124.45% (73.24% to 204.73%) |
|  |  | Age-standardized rate (per 100,000) | 0.52 (0.25 to 0.84) | 0.29 (0.01 to 0.55) | 0.74 (0.43 to 1.12) | 0.35 (0.22 to 0.51) | 0.11 (0.03 to 0.22) | 0.59 (0.39 to 0.84) | -32.75% (-46.72% to 5.75%) | -61.92% (-76.8% to -28.79%) | -20.09% (-38.23% to 8.74%) |
|  | YLLs | All age number | 13524.15 (6991.81 to 19208.86) | 3412.0 (191.02 to 6140.59) | 10112.15 (6610.09 to 13504.89) | 22070.56 (16131.33 to 28751.1) | 2974.64 (963.51 to 5373.56) | 19095.92 (14563.05 to 24003.64) | 63.19% (26.82% to 162.06%) | -12.82% (-46.39% to 66.09%) | 88.84% (45.33% to 154.77%) |
|  |  | Age-standardized rate (per 100,000) | 49.3 (25.38 to 70.15) | 26.22 (1.41 to 47.08) | 70.82 (46.68 to 94.93) | 28.24 (20.54 to 36.94) | 7.74 (2.35 to 14.14) | 48.97 (37.0 to 61.9) | -42.71% (-55.16% to -9.31%) | -70.47% (-81.7% to -45.08%) | -30.85% (-46.39% to -6.35%) |
| Gallbladder and biliary tract cancer | DALYs | All age number | 389.36 (233.58 to 580.53) | 279.96 (165.0 to 437.4) | 109.39 (57.1 to 155.65) | 2670.96 (1369.99 to 3813.8) | 1622.23 (886.28 to 2315.94) | 1048.72 (363.25 to 1602.97) | 585.99% (307.45% to 828.41%) | 479.44% (250.5% to 739.48%) | 858.69% (452.67% to 1199.48%) |
|  |  | Age-standardized rate (per 100,000) | 1.4 (0.85 to 2.07) | 2.11 (1.25 to 3.32) | 0.74 (0.38 to 1.06) | 3.33 (1.72 to 4.75) | 4.08 (2.23 to 5.82) | 2.57 (0.9 to 3.92) | 138.13% (42.47% to 220.28%) | 93.77% (17.63% to 174.28%) | 246.69% (107.24% to 364.69%) |
|  | Deaths | All age number | 13.92 (8.39 to 20.53) | 10.07 (5.9 to 15.95) | 3.85 (1.99 to 5.48) | 108.23 (55.75 to 154.15) | 67.15 (36.43 to 95.13) | 41.08 (14.65 to 62.1) | 677.46% (366.44% to 938.96%) | 566.53% (304.47% to 843.2%) | 968.01% (534.73% to 1348.76%) |
|  |  | Age-standardized rate (per 100,000) | 0.06 (0.04 to 0.09) | 0.09 (0.05 to 0.14) | 0.03 (0.02 to 0.04) | 0.15 (0.08 to 0.21) | 0.19 (0.1 to 0.26) | 0.11 (0.04 to 0.17) | 151.06% (50.27% to 230.05%) | 111.17% (28.17% to 193.19%) | 257.65% (124.48% to 378.31%) |
|  | YLDs | All age number | 3.4 (1.92 to 5.42) | 2.44 (1.35 to 4.07) | 0.96 (0.44 to 1.54) | 29.86 (14.26 to 47.77) | 18.0 (9.08 to 28.95) | 11.87 (3.95 to 19.51) | 778.12% (421.22% to 1080.35%) | 636.78% (344.68% to 953.81%) | 1138.5% (628.72% to 1587.03%) |
|  |  | Age-standardized rate (per 100,000) | 0.01 (0.01 to 0.02) | 0.02 (0.01 to 0.03) | 0.01 (0.0 to 0.01) | 0.04 (0.02 to 0.06) | 0.05 (0.02 to 0.08) | 0.03 (0.01 to 0.05) | 190.09% (73.5% to 285.62%) | 136.87% (43.61% to 230.97%) | 325.56% (163.03% to 474.12%) |
|  | YLLs | All age number | 385.95 (231.79 to 576.26) | 277.52 (163.54 to 434.48) | 108.43 (56.62 to 154.11) | 2641.09 (1354.69 to 3767.01) | 1604.23 (874.74 to 2287.5) | 1036.86 (358.99 to 1579.85) | 584.3% (306.5% to 825.92%) | 478.06% (249.68% to 737.56%) | 856.21% (451.19% to 1196.29%) |
|  |  | Age-standardized rate (per 100,000) | 1.39 (0.84 to 2.05) | 2.09 (1.23 to 3.29) | 0.74 (0.37 to 1.05) | 3.29 (1.7 to 4.69) | 4.03 (2.2 to 5.75) | 2.54 (0.89 to 3.86) | 137.62% (42.12% to 219.7%) | 93.36% (17.37% to 173.68%) | 245.91% (106.76% to 363.72%) |
| Kidney cancer | DALYs | All age number | 1302.55 (737.49 to 1814.96) | 416.48 (172.13 to 665.94) | 886.07 (531.0 to 1240.57) | 7094.93 (3914.36 to 10586.0) | 2277.65 (984.98 to 3523.87) | 4817.28 (2824.67 to 7278.6) | 444.7% (327.24% to 587.67%) | 446.88% (315.86% to 593.23%) | 443.67% (295.87% to 697.81%) |
|  |  | Age-standardized rate (per 100,000) | 4.42 (2.49 to 6.17) | 2.91 (1.2 to 4.65) | 5.8 (3.47 to 8.16) | 8.55 (4.72 to 12.84) | 5.5 (2.36 to 8.44) | 11.62 (6.85 to 17.63) | 93.53% (52.41% to 145.32%) | 88.97% (45.18% to 137.72%) | 100.46% (45.92% to 195.74%) |
|  | Deaths | All age number | 43.49 (24.75 to 60.6) | 13.29 (5.57 to 21.2) | 30.19 (18.03 to 42.47) | 257.42 (143.19 to 386.28) | 81.21 (34.34 to 124.11) | 176.21 (103.59 to 266.9) | 491.98% (363.55% to 653.48%) | 511.0% (370.88% to 665.53%) | 483.6% (323.92% to 764.85%) |
|  |  | Age-standardized rate (per 100,000) | 0.17 (0.1 to 0.24) | 0.11 (0.05 to 0.18) | 0.23 (0.13 to 0.32) | 0.34 (0.19 to 0.52) | 0.22 (0.09 to 0.33) | 0.47 (0.27 to 0.7) | 101.99% (56.86% to 159.48%) | 100.65% (53.76% to 153.07%) | 105.22% (47.72% to 203.49%) |
|  | YLDs | All age number | 35.94 (17.9 to 56.75) | 17.32 (7.01 to 29.29) | 18.62 (10.22 to 29.56) | 328.86 (160.87 to 538.38) | 170.13 (71.22 to 285.32) | 158.73 (82.47 to 255.64) | 815.08% (635.09% to 1028.59%) | 882.23% (632.94% to 1225.12%) | 752.6% (515.26% to 1153.54%) |
|  |  | Age-standardized rate (per 100,000) | 0.12 (0.06 to 0.19) | 0.11 (0.05 to 0.19) | 0.12 (0.07 to 0.19) | 0.38 (0.19 to 0.62) | 0.38 (0.16 to 0.63) | 0.38 (0.2 to 0.61) | 221.54% (155.78% to 299.02%) | 240.69% (157.11% to 351.41%) | 208.49% (123.07% to 352.98%) |
|  | YLLs | All age number | 1266.61 (719.62 to 1769.78) | 399.16 (166.23 to 639.7) | 867.45 (519.08 to 1213.35) | 6766.07 (3744.75 to 10083.09) | 2107.52 (901.57 to 3248.18) | 4658.55 (2721.08 to 7048.88) | 434.19% (318.21% to 575.14%) | 427.99% (301.73% to 567.82%) | 437.04% (291.09% to 687.72%) |
|  |  | Age-standardized rate (per 100,000) | 4.3 (2.43 to 6.0) | 2.8 (1.17 to 4.47) | 5.68 (3.39 to 8.0) | 8.17 (4.53 to 12.26) | 5.12 (2.18 to 7.84) | 11.24 (6.63 to 17.08) | 90.03% (49.64% to 141.16%) | 82.94% (40.68% to 129.87%) | 98.12% (44.17% to 192.27%) |
| Larynx cancer | DALYs | All age number | 12422.65 (10180.07 to 14633.47) | 915.17 (525.81 to 1258.36) | 11507.48 (9454.75 to 13668.49) | 22782.04 (19485.21 to 26385.95) | 1745.1 (1074.43 to 2353.51) | 21036.94 (17946.9 to 24568.79) | 83.39% (52.67% to 132.14%) | 90.69% (32.47% to 187.04%) | 82.81% (49.62% to 132.92%) |
|  |  | Age-standardized rate (per 100,000) | 42.72 (34.7 to 50.43) | 6.54 (3.73 to 8.99) | 76.12 (62.25 to 90.06) | 27.58 (23.57 to 31.92) | 4.17 (2.51 to 5.63) | 51.18 (43.61 to 59.79) | -35.44% (-46.21% to -18.55%) | -36.16% (-55.61% to -3.98%) | -32.77% (-45.02% to -14.6%) |
|  | Deaths | All age number | 427.08 (347.13 to 506.12) | 30.51 (17.42 to 41.98) | 396.58 (324.24 to 470.17) | 834.11 (709.68 to 971.12) | 62.84 (36.67 to 86.62) | 771.28 (652.96 to 907.05) | 95.31% (62.07% to 145.42%) | 105.98% (43.63% to 207.19%) | 94.48% (57.82% to 146.33%) |
|  |  | Age-standardized rate (per 100,000) | 1.65 (1.33 to 1.96) | 0.24 (0.14 to 0.35) | 3.0 (2.44 to 3.58) | 1.1 (0.93 to 1.29) | 0.16 (0.09 to 0.23) | 2.04 (1.73 to 2.41) | -33.33% (-44.72% to -16.52%) | -33.2% (-54.26% to 1.32%) | -32.02% (-44.61% to -13.89%) |
|  | YLDs | All age number | 282.07 (195.05 to 382.81) | 21.95 (12.82 to 33.52) | 260.12 (179.12 to 350.83) | 883.58 (620.23 to 1200.07) | 65.34 (37.67 to 100.06) | 818.24 (568.31 to 1097.36) | 213.25% (160.19% to 300.88%) | 197.71% (106.75% to 352.18%) | 214.56% (156.88% to 306.29%) |
|  |  | Age-standardized rate (per 100,000) | 1.0 (0.69 to 1.35) | 0.16 (0.09 to 0.25) | 1.78 (1.23 to 2.37) | 1.09 (0.77 to 1.47) | 0.16 (0.09 to 0.25) | 2.03 (1.42 to 2.72) | 9.07% (-9.62% to 38.22%) | -2.1% (-31.65% to 47.28%) | 14.39% (-6.17% to 47.14%) |
|  | YLLs | All age number | 12140.58 (9940.64 to 14301.93) | 893.22 (513.31 to 1226.96) | 11247.36 (9236.89 to 13342.37) | 21898.45 (18718.83 to 25392.53) | 1679.75 (1040.61 to 2271.07) | 20218.7 (17257.72 to 23513.68) | 80.37% (50.04% to 128.31%) | 88.06% (30.98% to 183.18%) | 79.76% (47.09% to 129.18%) |
|  |  | Age-standardized rate (per 100,000) | 41.72 (33.97 to 49.3) | 6.37 (3.64 to 8.77) | 74.34 (60.74 to 87.78) | 26.49 (22.6 to 30.71) | 4.01 (2.43 to 5.44) | 49.14 (41.84 to 57.31) | -36.51% (-47.06% to -19.9%) | -37.03% (-56.2% to -5.41%) | -33.89% (-45.93% to -16.09%) |
| Leukemia | DALYs | All age number | 8335.21 (4418.28 to 12134.69) | 2614.38 (1358.77 to 3516.16) | 5720.83 (2545.75 to 9046.97) | 20594.82 (12329.77 to 28664.46) | 6561.75 (3601.17 to 8741.98) | 14033.07 (6961.43 to 20895.35) | 147.08% (98.52% to 214.78%) | 150.99% (108.51% to 219.39%) | 145.3% (89.65% to 241.42%) |
|  |  | Age-standardized rate (per 100,000) | 26.3 (13.88 to 38.82) | 15.45 (8.13 to 20.73) | 36.54 (15.29 to 58.65) | 24.4 (14.27 to 34.53) | 15.07 (8.28 to 20.09) | 33.79 (16.08 to 51.42) | -7.21% (-25.78% to 18.94%) | -2.44% (-18.55% to 22.58%) | -7.53% (-29.71% to 30.61%) |
|  | Deaths | All age number | 255.77 (132.22 to 382.06) | 69.23 (36.5 to 93.04) | 186.54 (78.35 to 300.91) | 709.95 (401.85 to 1042.7) | 204.94 (112.97 to 275.35) | 505.01 (232.0 to 798.4) | 177.58% (121.25% to 254.0%) | 196.04% (146.97% to 270.73%) | 170.73% (107.42% to 273.42%) |
|  |  | Age-standardized rate (per 100,000) | 0.98 (0.5 to 1.5) | 0.5 (0.27 to 0.67) | 1.46 (0.58 to 2.45) | 0.94 (0.52 to 1.4) | 0.52 (0.29 to 0.7) | 1.35 (0.6 to 2.17) | -4.32% (-23.73% to 23.02%) | 5.01% (-12.51% to 30.27%) | -7.5% (-29.19% to 27.42%) |
|  | YLDs | All age number | 100.17 (45.93 to 169.9) | 26.61 (12.76 to 40.65) | 73.56 (27.14 to 133.49) | 416.86 (186.81 to 668.57) | 116.9 (51.52 to 183.46) | 299.96 (112.99 to 517.59) | 316.15% (221.54% to 461.72%) | 339.33% (251.14% to 469.42%) | 307.76% (191.16% to 496.15%) |
|  |  | Age-standardized rate (per 100,000) | 0.36 (0.16 to 0.62) | 0.18 (0.09 to 0.28) | 0.53 (0.19 to 0.98) | 0.53 (0.24 to 0.86) | 0.29 (0.13 to 0.45) | 0.78 (0.29 to 1.39) | 47.44% (12.42% to 99.49%) | 56.27% (26.07% to 99.54%) | 46.87% (3.66% to 115.17%) |
|  | YLLs | All age number | 8235.04 (4362.32 to 11978.36) | 2587.77 (1345.72 to 3477.48) | 5647.27 (2510.17 to 8920.44) | 20177.97 (12039.38 to 28034.33) | 6444.85 (3557.65 to 8573.84) | 13733.12 (6844.25 to 20479.72) | 145.03% (96.79% to 211.71%) | 149.05% (106.78% to 217.87%) | 143.18% (87.77% to 238.55%) |
|  |  | Age-standardized rate (per 100,000) | 25.94 (13.67 to 38.22) | 15.26 (8.03 to 20.47) | 36.01 (15.07 to 57.75) | 23.87 (14.03 to 33.57) | 14.78 (8.15 to 19.67) | 33.01 (15.78 to 50.01) | -7.97% (-26.58% to 17.99%) | -3.15% (-19.24% to 21.62%) | -8.33% (-30.27% to 29.43%) |
| Lip and oral cavity cancer | DALYs | All age number | 600.34 (403.64 to 814.2) | 82.25 (54.49 to 120.83) | 518.09 (337.63 to 724.97) | 2022.97 (1460.84 to 2550.77) | 269.01 (190.14 to 368.4) | 1753.96 (1251.66 to 2233.01) | 236.97% (165.79% to 326.08%) | 227.08% (126.62% to 362.19%) | 238.54% (152.61% to 339.29%) |
|  |  | Age-standardized rate (per 100,000) | 2.09 (1.41 to 2.83) | 0.66 (0.44 to 0.96) | 3.42 (2.22 to 4.82) | 2.4 (1.72 to 3.03) | 0.67 (0.47 to 0.94) | 4.12 (2.92 to 5.31) | 14.53% (-10.25% to 43.92%) | 2.6% (-30.0% to 48.27%) | 20.62% (-10.15% to 55.67%) |
|  | Deaths | All age number | 20.49 (13.71 to 28.0) | 3.05 (2.03 to 4.48) | 17.44 (11.22 to 24.62) | 72.8 (52.17 to 93.26) | 10.91 (7.67 to 16.02) | 61.89 (42.75 to 80.56) | 255.35% (182.45% to 347.0%) | 257.88% (148.34% to 421.99%) | 254.91% (166.85% to 352.79%) |
|  |  | Age-standardized rate (per 100,000) | 0.08 (0.06 to 0.11) | 0.03 (0.02 to 0.04) | 0.13 (0.08 to 0.19) | 0.1 (0.07 to 0.12) | 0.03 (0.02 to 0.05) | 0.16 (0.11 to 0.21) | 15.32% (-9.75% to 45.0%) | 3.59% (-32.71% to 60.14%) | 19.98% (-10.68% to 55.86%) |
|  | YLDs | All age number | 12.27 (7.61 to 18.43) | 2.0 (1.16 to 3.15) | 10.27 (6.18 to 15.72) | 63.73 (41.71 to 93.56) | 10.45 (6.62 to 16.08) | 53.28 (34.56 to 78.02) | 419.29% (307.31% to 572.16%) | 423.06% (253.55% to 660.67%) | 418.55% (285.25% to 588.01%) |
|  |  | Age-standardized rate (per 100,000) | 0.04 (0.03 to 0.07) | 0.02 (0.01 to 0.03) | 0.07 (0.04 to 0.11) | 0.08 (0.05 to 0.11) | 0.03 (0.02 to 0.04) | 0.13 (0.08 to 0.19) | 73.18% (34.78% to 119.46%) | 59.72% (6.87% to 134.36%) | 81.41% (35.59% to 138.02%) |
|  | YLLs | All age number | 588.07 (396.19 to 798.02) | 80.25 (53.3 to 118.02) | 507.82 (331.15 to 711.23) | 1959.25 (1415.86 to 2461.64) | 258.56 (183.96 to 355.16) | 1700.69 (1211.98 to 2170.03) | 233.17% (162.73% to 321.46%) | 222.2% (122.92% to 356.11%) | 234.9% (148.93% to 335.43%) |
|  |  | Age-standardized rate (per 100,000) | 2.05 (1.38 to 2.77) | 0.64 (0.43 to 0.93) | 3.35 (2.18 to 4.71) | 2.32 (1.66 to 2.93) | 0.65 (0.46 to 0.9) | 3.99 (2.81 to 5.15) | 13.25% (-11.25% to 42.2%) | 1.14% (-31.18% to 46.35%) | 19.34% (-11.51% to 54.17%) |
| Liver cancer | DALYs | All age number | 4296.9 (2943.24 to 6018.79) | 1596.58 (984.27 to 2407.23) | 2700.32 (1890.5 to 3752.95) | 22005.33 (15134.25 to 29548.25) | 8179.27 (4685.74 to 11997.4) | 13826.06 (10324.15 to 17748.71) | 412.12% (295.54% to 540.88%) | 412.3% (259.14% to 536.23%) | 412.02% (270.27% to 571.53%) |
|  |  | Age-standardized rate (per 100,000) | 15.09 (10.32 to 21.0) | 11.92 (7.21 to 17.96) | 17.84 (12.6 to 24.55) | 27.41 (18.74 to 36.68) | 20.33 (11.54 to 29.78) | 34.59 (25.93 to 44.26) | 81.64% (40.48% to 125.3%) | 70.58% (18.88% to 109.72%) | 93.89% (40.67% to 153.06%) |
|  | Deaths | All age number | 152.37 (104.54 to 212.79) | 56.1 (33.74 to 85.3) | 96.27 (67.59 to 132.91) | 890.81 (605.73 to 1193.84) | 332.73 (185.81 to 483.87) | 558.09 (421.94 to 717.89) | 484.63% (348.13% to 629.16%) | 493.1% (312.54% to 631.95%) | 479.7% (311.85% to 659.33%) |
|  |  | Age-standardized rate (per 100,000) | 0.62 (0.42 to 0.88) | 0.49 (0.28 to 0.77) | 0.72 (0.51 to 0.98) | 1.22 (0.83 to 1.62) | 0.92 (0.5 to 1.34) | 1.52 (1.16 to 1.96) | 97.72% (51.31% to 144.62%) | 85.65% (29.86% to 130.69%) | 110.57% (50.79% to 174.07%) |
|  | YLDs | All age number | 33.6 (19.86 to 52.17) | 12.32 (6.48 to 20.12) | 21.28 (12.99 to 33.05) | 195.11 (119.42 to 295.18) | 72.55 (35.88 to 119.04) | 122.57 (79.19 to 176.76) | 480.73% (346.02% to 624.41%) | 488.82% (310.59% to 623.43%) | 476.04% (313.13% to 655.48%) |
|  |  | Age-standardized rate (per 100,000) | 0.13 (0.08 to 0.2) | 0.1 (0.05 to 0.17) | 0.15 (0.09 to 0.24) | 0.26 (0.16 to 0.39) | 0.19 (0.09 to 0.31) | 0.32 (0.21 to 0.47) | 100.21% (53.59% to 148.79%) | 88.22% (31.32% to 133.59%) | 113.42% (53.79% to 178.04%) |
|  | YLLs | All age number | 4263.3 (2920.71 to 5978.82) | 1584.26 (977.71 to 2388.07) | 2679.05 (1875.77 to 3725.88) | 21810.22 (15007.25 to 29301.4) | 8106.72 (4645.2 to 11893.89) | 13703.5 (10248.96 to 17568.27) | 411.58% (295.15% to 540.22%) | 411.7% (258.55% to 535.55%) | 411.51% (269.91% to 570.76%) |
|  |  | Age-standardized rate (per 100,000) | 14.96 (10.23 to 20.84) | 11.82 (7.15 to 17.79) | 17.69 (12.49 to 24.36) | 27.15 (18.57 to 36.35) | 20.14 (11.44 to 29.5) | 34.27 (25.73 to 43.89) | 81.48% (40.33% to 125.12%) | 70.43% (18.75% to 109.54%) | 93.73% (40.55% to 152.84%) |
| Mesothelioma | DALYs | All age number | 216.96 (156.64 to 277.61) | 7.08 (2.45 to 20.52) | 209.88 (143.49 to 269.8) | 602.99 (463.78 to 742.39) | 26.1 (13.66 to 74.83) | 576.89 (440.62 to 707.4) | 177.93% (89.24% to 295.53%) | 268.62% (81.73% to 671.3%) | 174.87% (82.88% to 292.83%) |
|  |  | Age-standardized rate (per 100,000) | 0.83 (0.61 to 1.03) | 0.08 (0.03 to 0.17) | 1.54 (1.08 to 1.94) | 0.79 (0.62 to 0.96) | 0.08 (0.05 to 0.2) | 1.52 (1.18 to 1.83) | -4.12% (-32.75% to 35.33%) | 4.23% (-42.38% to 96.64%) | -1.64% (-32.83% to 40.87%) |
|  | Deaths | All age number | 8.46 (6.2 to 10.44) | 0.38 (0.17 to 0.89) | 8.08 (5.76 to 10.08) | 27.6 (22.41 to 32.59) | 1.59 (0.97 to 3.45) | 26.01 (20.58 to 30.82) | 226.17% (138.35% to 357.01%) | 316.57% (133.79% to 674.39%) | 221.89% (128.28% to 360.32%) |
|  |  | Age-standardized rate (per 100,000) | 0.04 (0.03 to 0.05) | 0.0 (0.0 to 0.01) | 0.07 (0.05 to 0.09) | 0.04 (0.03 to 0.05) | 0.01 (0.0 to 0.01) | 0.07 (0.06 to 0.09) | 2.97% (-22.26% to 42.01%) | 6.82% (-32.05% to 83.85%) | 2.18% (-25.82% to 43.22%) |
|  | YLDs | All age number | 3.24 (2.02 to 4.62) | 0.11 (0.04 to 0.29) | 3.13 (1.96 to 4.52) | 10.06 (6.59 to 13.88) | 0.47 (0.23 to 1.29) | 9.6 (6.31 to 13.17) | 210.52% (119.13% to 338.63%) | 317.3% (115.05% to 743.05%) | 206.71% (111.52% to 341.76%) |
|  |  | Age-standardized rate (per 100,000) | 0.01 (0.01 to 0.02) | 0.0 (0.0 to 0.0) | 0.03 (0.02 to 0.04) | 0.01 (0.01 to 0.02) | 0.0 (0.0 to 0.0) | 0.03 (0.02 to 0.04) | 3.66% (-24.67% to 44.53%) | 12.78% (-34.62% to 104.8%) | 4.83% (-26.23% to 48.99%) |
|  | YLLs | All age number | 213.72 (154.19 to 273.48) | 6.97 (2.4 to 20.24) | 206.75 (141.24 to 266.33) | 592.93 (455.73 to 730.51) | 25.63 (13.37 to 73.58) | 567.3 (433.38 to 697.62) | 177.43% (88.81% to 294.95%) | 267.84% (81.09% to 670.25%) | 174.39% (82.4% to 291.99%) |
|  |  | Age-standardized rate (per 100,000) | 0.82 (0.6 to 1.02) | 0.07 (0.03 to 0.17) | 1.52 (1.06 to 1.91) | 0.78 (0.61 to 0.94) | 0.08 (0.04 to 0.2) | 1.49 (1.16 to 1.8) | -4.25% (-32.87% to 35.16%) | 4.08% (-42.49% to 96.47%) | -1.75% (-32.93% to 40.73%) |
| Multiple myeloma | DALYs | All age number | 207.69 (-78.85 to 550.83) | 103.75 (-43.14 to 299.49) | 103.94 (-27.95 to 283.81) | 1692.64 (-754.87 to 4376.34) | 825.14 (-387.56 to 2105.28) | 867.5 (-363.33 to 2357.39) | 714.99% (446.74% to 1168.96%) | 695.32% (285.06% to 1240.73%) | 734.63% (493.03% to 1276.59%) |
|  |  | Age-standardized rate (per 100,000) | 0.7 (-0.26 to 1.87) | 0.74 (-0.31 to 2.07) | 0.67 (-0.17 to 1.83) | 2.05 (-0.92 to 5.26) | 1.99 (-0.94 to 5.07) | 2.1 (-0.86 to 5.77) | 191.88% (98.33% to 349.17%) | 168.76% (32.89% to 351.02%) | 214.98% (121.99% to 416.16%) |
|  | Deaths | All age number | 6.99 (-2.59 to 18.51) | 3.5 (-1.48 to 9.76) | 3.49 (-0.91 to 9.67) | 61.77 (-27.83 to 157.77) | 30.15 (-14.17 to 76.65) | 31.61 (-12.73 to 87.44) | 784.1% (494.37% to 1262.2%) | 762.36% (333.42% to 1348.69%) | 805.88% (540.6% to 1381.58%) |
|  |  | Age-standardized rate (per 100,000) | 0.03 (-0.01 to 0.07) | 0.03 (-0.01 to 0.08) | 0.03 (-0.01 to 0.07) | 0.08 (-0.04 to 0.21) | 0.08 (-0.04 to 0.2) | 0.08 (-0.03 to 0.23) | 205.49% (107.79% to 371.13%) | 180.65% (46.33% to 367.38%) | 229.85% (131.74% to 445.89%) |
|  | YLDs | All age number | 3.64 (-1.22 to 10.06) | 1.83 (-0.7 to 5.44) | 1.81 (-0.5 to 5.15) | 45.24 (-19.52 to 123.56) | 22.81 (-11.02 to 62.55) | 22.43 (-9.81 to 62.25) | 1142.17% (719.24% to 1864.62%) | 1148.17% (498.85% to 2126.93%) | 1136.12% (734.59% to 1997.83%) |
|  |  | Age-standardized rate (per 100,000) | 0.01 (-0.0 to 0.04) | 0.01 (-0.01 to 0.04) | 0.01 (-0.0 to 0.03) | 0.06 (-0.02 to 0.15) | 0.06 (-0.03 to 0.15) | 0.06 (-0.02 to 0.15) | 331.67% (188.92% to 574.49%) | 307.77% (101.06% to 623.34%) | 354.11% (207.58% to 661.38%) |
|  | YLLs | All age number | 204.05 (-77.76 to 540.39) | 101.92 (-42.5 to 294.72) | 102.12 (-27.53 to 278.71) | 1647.4 (-738.26 to 4254.72) | 802.34 (-375.82 to 2045.81) | 845.07 (-352.94 to 2294.32) | 707.37% (441.21% to 1159.0%) | 687.2% (282.3% to 1226.95%) | 727.5% (487.78% to 1263.74%) |
|  |  | Age-standardized rate (per 100,000) | 0.69 (-0.26 to 1.84) | 0.73 (-0.3 to 2.03) | 0.65 (-0.17 to 1.8) | 1.99 (-0.9 to 5.11) | 1.93 (-0.91 to 4.92) | 2.04 (-0.84 to 5.62) | 189.25% (96.59% to 345.34%) | 166.13% (31.88% to 346.02%) | 212.36% (120.59% to 412.42%) |
| Nasopharynx cancer | DALYs | All age number | 219.6 (155.08 to 294.26) | 15.9 (10.92 to 22.43) | 203.7 (143.55 to 274.68) | 506.22 (393.74 to 621.98) | 29.24 (21.07 to 40.01) | 476.98 (368.49 to 587.3) | 130.52% (81.74% to 211.63%) | 83.93% (29.75% to 171.38%) | 134.16% (81.9% to 219.5%) |
|  |  | Age-standardized rate (per 100,000) | 0.72 (0.5 to 0.97) | 0.11 (0.07 to 0.15) | 1.28 (0.9 to 1.75) | 0.57 (0.44 to 0.71) | 0.07 (0.05 to 0.09) | 1.07 (0.83 to 1.34) | -20.61% (-37.2% to 7.22%) | -37.5% (-56.32% to -8.69%) | -16.44% (-34.59% to 13.14%) |
|  | Deaths | All age number | 7.05 (4.94 to 9.62) | 0.49 (0.33 to 0.7) | 6.56 (4.61 to 8.98) | 16.18 (12.32 to 20.53) | 0.96 (0.67 to 1.33) | 15.22 (11.56 to 19.11) | 129.71% (82.21% to 209.53%) | 97.1% (36.37% to 189.34%) | 132.13% (82.48% to 212.59%) |
|  |  | Age-standardized rate (per 100,000) | 0.03 (0.02 to 0.04) | 0.0 (0.0 to 0.01) | 0.05 (0.03 to 0.06) | 0.02 (0.01 to 0.03) | 0.0 (0.0 to 0.0) | 0.04 (0.03 to 0.05) | -22.28% (-38.34% to 3.67%) | -35.05% (-54.71% to -3.07%) | -19.64% (-36.62% to 7.98%) |
|  | YLDs | All age number | 2.87 (1.78 to 4.33) | 0.2 (0.12 to 0.32) | 2.67 (1.65 to 4.04) | 8.61 (5.97 to 12.27) | 0.52 (0.33 to 0.78) | 8.09 (5.62 to 11.56) | 199.68% (137.95% to 306.74%) | 155.85% (79.82% to 281.29%) | 203.03% (138.48% to 312.46%) |
|  |  | Age-standardized rate (per 100,000) | 0.01 (0.01 to 0.02) | 0.0 (0.0 to 0.0) | 0.02 (0.01 to 0.03) | 0.01 (0.01 to 0.01) | 0.0 (0.0 to 0.0) | 0.02 (0.01 to 0.03) | -1.49% (-20.75% to 33.26%) | -17.64% (-42.27% to 23.74%) | 2.65% (-18.02% to 39.13%) |
|  | YLLs | All age number | 216.72 (152.83 to 290.66) | 15.69 (10.79 to 22.12) | 201.03 (141.76 to 271.03) | 497.61 (386.57 to 612.01) | 28.72 (20.66 to 39.23) | 468.89 (361.72 to 577.18) | 129.61% (80.92% to 210.38%) | 83.0% (29.08% to 169.9%) | 133.25% (81.1% to 218.44%) |
|  |  | Age-standardized rate (per 100,000) | 0.71 (0.5 to 0.96) | 0.11 (0.07 to 0.15) | 1.26 (0.88 to 1.73) | 0.56 (0.44 to 0.7) | 0.07 (0.05 to 0.09) | 1.05 (0.81 to 1.32) | -20.88% (-37.46% to 6.89%) | -37.77% (-56.51% to -9.08%) | -16.71% (-34.84% to 12.86%) |
| Non-Hodgkin lymphoma | DALYs | All age number | 506.95 (162.71 to 872.08) | 222.95 (65.14 to 407.45) | 284.0 (91.25 to 478.09) | 2946.06 (907.3 to 5272.41) | 1329.55 (394.88 to 2357.54) | 1616.51 (483.68 to 2936.51) | 481.13% (308.5% to 684.78%) | 496.35% (299.07% to 817.34%) | 469.19% (261.62% to 643.34%) |
|  |  | Age-standardized rate (per 100,000) | 1.53 (0.5 to 2.64) | 1.4 (0.41 to 2.58) | 1.64 (0.53 to 2.77) | 3.36 (1.04 to 6.01) | 3.1 (0.92 to 5.46) | 3.61 (1.08 to 6.53) | 119.91% (56.18% to 199.26%) | 121.5% (49.67% to 242.92%) | 119.69% (37.61% to 187.85%) |
|  | Deaths | All age number | 14.41 (4.71 to 24.94) | 6.33 (1.85 to 11.88) | 8.07 (2.6 to 13.66) | 91.17 (28.19 to 161.77) | 42.87 (12.84 to 74.48) | 48.3 (14.55 to 86.96) | 532.85% (345.12% to 755.27%) | 576.93% (358.56% to 945.89%) | 498.28% (272.64% to 682.92%) |
|  |  | Age-standardized rate (per 100,000) | 0.05 (0.02 to 0.09) | 0.05 (0.01 to 0.09) | 0.05 (0.02 to 0.09) | 0.11 (0.04 to 0.2) | 0.11 (0.03 to 0.19) | 0.12 (0.04 to 0.21) | 125.19% (60.43% to 207.76%) | 132.05% (57.88% to 263.87%) | 120.6% (38.53% to 189.18%) |
|  | YLDs | All age number | 7.59 (2.15 to 13.84) | 3.35 (0.89 to 6.45) | 4.24 (1.4 to 7.6) | 125.14 (36.0 to 231.83) | 54.64 (15.4 to 102.1) | 70.5 (20.03 to 134.47) | 1549.7% (1019.41% to 2258.27%) | 1531.75% (955.63% to 2524.67%) | 1563.89% (919.9% to 2261.38%) |
|  |  | Age-standardized rate (per 100,000) | 0.02 (0.01 to 0.04) | 0.02 (0.01 to 0.04) | 0.02 (0.01 to 0.04) | 0.14 (0.04 to 0.26) | 0.12 (0.03 to 0.23) | 0.15 (0.04 to 0.29) | 515.53% (321.81% to 783.07%) | 496.84% (292.87% to 854.33%) | 534.16% (284.74% to 798.87%) |
|  | YLLs | All age number | 499.37 (160.18 to 858.88) | 219.6 (64.19 to 400.96) | 279.77 (89.76 to 470.68) | 2820.92 (874.12 to 5044.37) | 1274.91 (378.75 to 2253.01) | 1546.01 (463.56 to 2799.33) | 464.9% (297.15% to 662.36%) | 480.56% (289.24% to 792.66%) | 452.61% (251.56% to 621.35%) |
|  |  | Age-standardized rate (per 100,000) | 1.5 (0.49 to 2.6) | 1.38 (0.4 to 2.54) | 1.62 (0.52 to 2.73) | 3.22 (1.0 to 5.76) | 2.97 (0.88 to 5.23) | 3.46 (1.04 to 6.21) | 113.95% (51.82% to 190.99%) | 115.81% (45.41% to 233.14%) | 113.49% (33.63% to 180.0%) |
| Other pharynx cancer | DALYs | All age number | 289.82 (205.86 to 386.92) | 49.13 (31.31 to 75.28) | 240.69 (164.73 to 322.09) | 693.55 (532.04 to 871.58) | 104.12 (65.47 to 175.81) | 589.42 (451.69 to 747.92) | 139.3% (83.77% to 225.31%) | 111.93% (41.42% to 213.12%) | 144.89% (80.78% to 246.98%) |
|  |  | Age-standardized rate (per 100,000) | 0.97 (0.68 to 1.3) | 0.34 (0.22 to 0.52) | 1.54 (1.05 to 2.07) | 0.81 (0.62 to 1.02) | 0.24 (0.15 to 0.4) | 1.38 (1.05 to 1.75) | -16.4% (-35.25% to 14.16%) | -29.45% (-52.49% to 4.21%) | -10.42% (-32.8% to 27.68%) |
|  | Deaths | All age number | 9.68 (6.8 to 13.0) | 1.6 (1.03 to 2.38) | 8.08 (5.49 to 10.87) | 24.17 (18.27 to 30.61) | 3.62 (2.2 to 5.77) | 20.56 (15.56 to 26.19) | 149.72% (92.21% to 240.87%) | 125.69% (53.59% to 235.86%) | 154.49% (89.22% to 263.8%) |
|  |  | Age-standardized rate (per 100,000) | 0.04 (0.02 to 0.05) | 0.01 (0.01 to 0.02) | 0.06 (0.04 to 0.08) | 0.03 (0.02 to 0.04) | 0.01 (0.01 to 0.01) | 0.05 (0.04 to 0.07) | -14.35% (-33.82% to 17.22%) | -27.09% (-50.65% to 7.59%) | -9.31% (-31.52% to 30.58%) |
|  | YLDs | All age number | 2.89 (1.74 to 4.41) | 0.54 (0.3 to 0.92) | 2.35 (1.4 to 3.53) | 11.14 (7.41 to 16.09) | 2.22 (1.16 to 3.94) | 8.92 (5.92 to 12.93) | 285.21% (195.73% to 426.9%) | 307.24% (167.98% to 509.43%) | 280.1% (179.33% to 449.96%) |
|  |  | Age-standardized rate (per 100,000) | 0.01 (0.01 to 0.02) | 0.0 (0.0 to 0.01) | 0.02 (0.01 to 0.02) | 0.01 (0.01 to 0.02) | 0.01 (0.0 to 0.01) | 0.02 (0.01 to 0.03) | 30.38% (0.52% to 77.01%) | 30.35% (-13.77% to 93.16%) | 34.32% (-0.07% to 93.77%) |
|  | YLLs | All age number | 286.93 (203.77 to 382.79) | 48.59 (30.9 to 74.34) | 238.34 (162.85 to 318.73) | 682.41 (522.82 to 856.84) | 101.91 (64.01 to 172.2) | 580.5 (445.88 to 737.01) | 137.83% (82.76% to 223.68%) | 109.74% (40.01% to 210.0%) | 143.56% (79.84% to 245.08%) |
|  |  | Age-standardized rate (per 100,000) | 0.96 (0.67 to 1.29) | 0.34 (0.22 to 0.51) | 1.52 (1.04 to 2.05) | 0.8 (0.61 to 1.0) | 0.24 (0.15 to 0.4) | 1.36 (1.04 to 1.73) | -16.9% (-35.69% to 13.58%) | -30.15% (-52.88% to 3.1%) | -10.89% (-33.15% to 27.0%) |
| Ovarian cancer | DALYs | All age number | 532.03 (101.21 to 1071.86) | 532.03 (101.21 to 1071.86) | NA | 4521.9 (1373.92 to 8041.36) | 4521.9 (1373.92 to 8041.36) | NA | 749.93% (415.53% to 1232.0%) | 749.93% (415.53% to 1232.0%) | NA |
|  |  | Age-standardized rate (per 100,000) | 1.67 (0.32 to 3.35) | 3.48 (0.68 to 6.99) | NA | 5.11 (1.54 to 9.07) | 10.22 (3.08 to 18.16) | NA | 206.74% (86.58% to 381.74%) | 193.95% (78.27% to 360.38%) | NA |
|  | Deaths | All age number | 15.43 (3.08 to 31.18) | 15.43 (3.08 to 31.18) | NA | 142.77 (42.69 to 253.84) | 142.77 (42.69 to 253.84) | NA | 825.0% (463.95% to 1322.48%) | 825.0% (463.95% to 1322.48%) | NA |
|  |  | Age-standardized rate (per 100,000) | 0.05 (0.01 to 0.11) | 0.11 (0.02 to 0.23) | NA | 0.17 (0.05 to 0.31) | 0.35 (0.1 to 0.62) | NA | 223.93% (97.55% to 409.95%) | 211.62% (90.18% to 388.35%) | NA |
|  | YLDs | All age number | 14.32 (2.91 to 29.56) | 14.32 (2.91 to 29.56) | NA | 140.56 (41.05 to 270.33) | 140.56 (41.05 to 270.33) | NA | 881.44% (498.97% to 1477.81%) | 881.44% (498.97% to 1477.81%) | NA |
|  |  | Age-standardized rate (per 100,000) | 0.04 (0.01 to 0.09) | 0.09 (0.02 to 0.19) | NA | 0.15 (0.05 to 0.3) | 0.31 (0.09 to 0.59) | NA | 253.16% (116.05% to 464.65%) | 240.38% (107.26% to 445.01%) | NA |
|  | YLLs | All age number | 517.71 (98.68 to 1049.38) | 517.71 (98.68 to 1049.38) | NA | 4381.34 (1328.19 to 7785.53) | 4381.34 (1328.19 to 7785.53) | NA | 746.29% (412.63% to 1227.31%) | 746.29% (412.63% to 1227.31%) | NA |
|  |  | Age-standardized rate (per 100,000) | 1.62 (0.32 to 3.29) | 3.39 (0.66 to 6.85) | NA | 4.95 (1.49 to 8.8) | 9.91 (2.98 to 17.6) | NA | 205.49% (85.71% to 379.5%) | 192.69% (77.7% to 358.17%) | NA |
| Pancreatic cancer | DALYs | All age number | 3691.98 (1815.81 to 5900.36) | 1058.46 (221.96 to 2094.09) | 2633.52 (1525.19 to 3908.25) | 25340.77 (9985.25 to 40288.53) | 8197.13 (1595.07 to 14592.41) | 17143.64 (8638.84 to 25848.99) | 586.37% (404.82% to 771.82%) | 674.44% (433.42% to 1056.62%) | 550.98% (371.51% to 749.92%) |
|  |  | Age-standardized rate (per 100,000) | 13.65 (6.3 to 22.14) | 8.72 (1.69 to 17.49) | 18.2 (10.11 to 27.36) | 32.17 (12.08 to 51.91) | 21.36 (4.01 to 38.11) | 43.08 (20.78 to 65.7) | 135.73% (76.86% to 198.07%) | 144.98% (73.78% to 269.61%) | 136.66% (74.2% to 207.93%) |
|  | Deaths | All age number | 138.63 (62.78 to 226.49) | 42.85 (8.2 to 86.11) | 95.78 (52.76 to 145.69) | 1061.83 (379.06 to 1730.28) | 366.5 (66.26 to 654.95) | 695.33 (318.61 to 1080.02) | 665.93% (446.9% to 879.6%) | 755.34% (493.13% to 1192.93%) | 625.93% (418.38% to 842.88%) |
|  |  | Age-standardized rate (per 100,000) | 0.6 (0.24 to 1.0) | 0.41 (0.07 to 0.84) | 0.77 (0.4 to 1.21) | 1.47 (0.5 to 2.42) | 1.04 (0.18 to 1.86) | 1.9 (0.83 to 3.0) | 145.8% (83.52% to 212.7%) | 152.95% (82.18% to 277.29%) | 145.15% (82.99% to 214.63%) |
|  | YLDs | All age number | 29.19 (12.31 to 50.89) | 8.7 (1.62 to 18.31) | 20.48 (10.41 to 33.75) | 222.88 (76.23 to 397.29) | 74.04 (12.47 to 142.52) | 148.84 (64.51 to 252.61) | 663.6% (450.16% to 867.34%) | 750.68% (486.98% to 1182.61%) | 626.59% (426.99% to 856.35%) |
|  |  | Age-standardized rate (per 100,000) | 0.12 (0.05 to 0.21) | 0.08 (0.01 to 0.17) | 0.16 (0.08 to 0.26) | 0.3 (0.1 to 0.54) | 0.2 (0.03 to 0.39) | 0.39 (0.16 to 0.68) | 151.08% (87.64% to 216.36%) | 157.19% (83.73% to 284.86%) | 152.0% (88.08% to 227.25%) |
|  | YLLs | All age number | 3662.79 (1802.02 to 5856.43) | 1049.76 (220.41 to 2074.71) | 2613.04 (1513.58 to 3878.52) | 25117.89 (9916.0 to 39994.12) | 8123.09 (1583.28 to 14478.37) | 16994.8 (8573.45 to 25635.23) | 585.76% (404.38% to 771.0%) | 673.81% (433.0% to 1055.84%) | 550.39% (371.11% to 749.25%) |
|  |  | Age-standardized rate (per 100,000) | 13.53 (6.24 to 21.93) | 8.64 (1.67 to 17.32) | 18.05 (10.03 to 27.07) | 31.87 (11.98 to 51.43) | 21.16 (3.98 to 37.7) | 42.69 (20.64 to 65.18) | 135.59% (76.75% to 197.92%) | 144.87% (73.7% to 269.42%) | 136.53% (74.11% to 207.66%) |
| Prostate cancer | DALYs | All age number | 38.82 (-956.32 to 896.16) | NA | 38.82 (-956.32 to 896.16) | 791.6 (-1390.89 to 2796.0) | NA | 791.6 (-1390.89 to 2796.0) | 1939.35% (-1878.28% to 2784.72%) | NA | 1939.35% (-1878.28% to 2784.72%) |
|  |  | Age-standardized rate (per 100,000) | -0.28 (-5.04 to 3.73) | NA | -0.75 (-10.45 to 7.37) | 0.91 (-2.22 to 3.76) | NA | 1.88 (-4.4 to 7.53) | -423.4% (-964.27% to 602.4%) | NA | -350.97% (-932.69% to 621.59%) |
|  | Deaths | All age number | -2.16 (-48.49 to 37.07) | NA | -2.16 (-48.49 to 37.07) | 19.2 (-102.03 to 124.54) | NA | 19.2 (-102.03 to 124.54) | -989.14% (-2434.35% to 1861.32%) | NA | -989.14% (-2434.35% to 1861.32%) |
|  |  | Age-standardized rate (per 100,000) | -0.04 (-0.31 to 0.18) | NA | -0.09 (-0.65 to 0.37) | 0.02 (-0.17 to 0.18) | NA | 0.04 (-0.33 to 0.37) | -150.34% (-512.48% to 421.26%) | NA | -147.3% (-495.24% to 418.0%) |
|  | YLDs | All age number | 5.02 (-49.26 to 54.59) | NA | 5.02 (-49.26 to 54.59) | 115.74 (-94.45 to 334.09) | NA | 115.74 (-94.45 to 334.09) | 2207.07% (-3766.23% to 8062.22%) | NA | 2207.07% (-3766.23% to 8062.22%) |
|  |  | Age-standardized rate (per 100,000) | 0.0 (-0.22 to 0.2) | NA | -0.0 (-0.44 to 0.39) | 0.14 (-0.15 to 0.42) | NA | 0.28 (-0.3 to 0.85) | 5288.22% (-2137.44% to 2153.28%) | NA | -12512.61% (-2391.95% to 2189.2%) |
|  | YLLs | All age number | 33.8 (-908.37 to 848.2) | NA | 33.8 (-908.37 to 848.2) | 675.85 (-1283.56 to 2478.04) | NA | 675.85 (-1283.56 to 2478.04) | 1899.61% (-2121.09% to 2684.94%) | NA | 1899.61% (-2121.09% to 2684.94%) |
|  |  | Age-standardized rate (per 100,000) | -0.29 (-4.83 to 3.53) | NA | -0.75 (-10.01 to 6.97) | 0.78 (-2.09 to 3.37) | NA | 1.6 (-4.11 to 6.79) | -372.37% (-803.95% to 541.2%) | NA | -314.41% (-746.3% to 631.78%) |
| Stomach cancer | DALYs | All age number | 19855.36 (8287.43 to 57926.15) | 3156.24 (564.29 to 15926.3) | 16699.12 (7574.14 to 42701.92) | 29035.44 (12468.28 to 86422.52) | 4441.35 (810.3 to 22721.32) | 24594.09 (11540.96 to 64252.42) | 46.23% (20.78% to 79.75%) | 40.72% (-0.22% to 97.57%) | 47.28% (19.85% to 83.56%) |
|  |  | Age-standardized rate (per 100,000) | 70.02 (29.3 to 203.9) | 22.18 (4.04 to 113.11) | 114.64 (52.05 to 293.19) | 36.0 (15.33 to 106.76) | 10.74 (1.93 to 55.75) | 61.54 (28.54 to 160.97) | -48.59% (-57.65% to -37.02%) | -51.58% (-65.63% to -34.34%) | -46.32% (-56.56% to -32.89%) |
|  | Deaths | All age number | 711.07 (295.97 to 2046.85) | 104.2 (18.79 to 534.82) | 606.87 (270.93 to 1548.31) | 1161.11 (491.24 to 3453.88) | 169.65 (29.85 to 897.05) | 991.47 (459.08 to 2602.92) | 63.29% (33.39% to 101.87%) | 62.8% (13.43% to 118.8%) | 63.37% (30.99% to 104.23%) |
|  |  | Age-standardized rate (per 100,000) | 2.89 (1.16 to 8.4) | 0.86 (0.15 to 4.44) | 4.87 (2.13 to 12.47) | 1.58 (0.66 to 4.72) | 0.45 (0.08 to 2.45) | 2.72 (1.24 to 7.17) | -45.21% (-55.05% to -32.3%) | -47.18% (-62.27% to -27.75%) | -44.26% (-55.12% to -30.32%) |
|  | YLDs | All age number | 180.92 (73.75 to 535.89) | 24.23 (3.98 to 122.69) | 156.68 (67.61 to 420.65) | 339.27 (133.77 to 1017.15) | 37.96 (6.12 to 202.59) | 301.31 (125.39 to 828.9) | 87.53% (52.82% to 134.31%) | 56.66% (9.05% to 113.84%) | 92.31% (53.89% to 142.51%) |
|  |  | Age-standardized rate (per 100,000) | 0.67 (0.27 to 1.99) | 0.18 (0.03 to 0.95) | 1.13 (0.49 to 3.02) | 0.43 (0.17 to 1.3) | 0.1 (0.02 to 0.52) | 0.77 (0.32 to 2.11) | -35.44% (-47.5% to -19.24%) | -47.35% (-62.45% to -28.19%) | -31.66% (-45.05% to -14.27%) |
|  | YLLs | All age number | 19674.44 (8208.7 to 57376.05) | 3132.0 (560.13 to 15793.04) | 16542.44 (7505.3 to 42295.59) | 28696.17 (12296.89 to 85578.57) | 4403.39 (803.65 to 22525.3) | 24292.78 (11378.08 to 63449.78) | 45.86% (20.43% to 79.19%) | 40.59% (-0.3% to 97.44%) | 46.85% (19.5% to 83.05%) |
|  |  | Age-standardized rate (per 100,000) | 69.35 (29.03 to 201.86) | 22.0 (4.0 to 112.12) | 113.51 (51.52 to 290.37) | 35.56 (15.11 to 105.61) | 10.64 (1.91 to 55.33) | 60.77 (28.12 to 158.94) | -48.72% (-57.76% to -37.21%) | -51.61% (-65.65% to -34.39%) | -46.46% (-56.68% to -33.08%) |
| Thyroid cancer | DALYs | All age number | 149.88 (90.98 to 211.14) | 106.34 (61.47 to 152.73) | 43.54 (25.61 to 60.75) | 1288.08 (520.29 to 1821.71) | 793.26 (297.63 to 1131.87) | 494.82 (198.37 to 701.08) | 759.41% (387.8% to 1049.49%) | 645.95% (325.36% to 981.65%) | 1036.57% (489.11% to 1457.23%) |
|  |  | Age-standardized rate (per 100,000) | 0.5 (0.3 to 0.71) | 0.74 (0.42 to 1.07) | 0.28 (0.17 to 0.39) | 1.5 (0.62 to 2.12) | 1.87 (0.71 to 2.65) | 1.14 (0.47 to 1.61) | 198.38% (73.78% to 294.21%) | 150.87% (46.15% to 257.88%) | 308.56% (114.68% to 460.12%) |
|  | Deaths | All age number | 4.36 (2.65 to 6.13) | 3.05 (1.7 to 4.43) | 1.31 (0.77 to 1.81) | 37.73 (16.27 to 52.88) | 23.12 (8.98 to 32.88) | 14.61 (6.26 to 20.75) | 765.17% (405.83% to 1034.53%) | 657.24% (341.65% to 961.54%) | 1017.29% (500.0% to 1440.26%) |
|  |  | Age-standardized rate (per 100,000) | 0.02 (0.01 to 0.02) | 0.03 (0.01 to 0.04) | 0.01 (0.01 to 0.01) | 0.05 (0.02 to 0.07) | 0.06 (0.02 to 0.09) | 0.04 (0.02 to 0.05) | 180.22% (67.69% to 265.84%) | 141.01% (42.8% to 234.91%) | 276.41% (104.71% to 417.81%) |
|  | YLDs | All age number | 20.16 (10.43 to 33.0) | 16.24 (7.98 to 27.51) | 3.91 (2.07 to 6.16) | 279.29 (100.45 to 459.78) | 197.57 (68.08 to 327.55) | 81.72 (28.54 to 132.5) | 1285.52% (727.81% to 1879.76%) | 1116.31% (618.14% to 1770.57%) | 1987.76% (980.94% to 2924.5%) |
|  |  | Age-standardized rate (per 100,000) | 0.06 (0.03 to 0.1) | 0.1 (0.05 to 0.17) | 0.02 (0.01 to 0.04) | 0.29 (0.11 to 0.48) | 0.42 (0.14 to 0.7) | 0.17 (0.06 to 0.27) | 395.9% (199.52% to 602.21%) | 324.37% (156.65% to 549.61%) | 657.67% (302.72% to 1001.23%) |
|  | YLLs | All age number | 129.72 (80.14 to 180.82) | 90.1 (51.57 to 129.94) | 39.62 (23.39 to 54.7) | 1008.79 (425.19 to 1407.7) | 595.69 (229.56 to 848.19) | 413.1 (172.29 to 587.13) | 677.66% (338.58% to 923.33%) | 561.15% (277.18% to 843.33%) | 942.6% (444.03% to 1321.43%) |
|  |  | Age-standardized rate (per 100,000) | 0.44 (0.27 to 0.62) | 0.65 (0.36 to 0.93) | 0.26 (0.15 to 0.35) | 1.21 (0.52 to 1.69) | 1.45 (0.56 to 2.06) | 0.97 (0.41 to 1.37) | 172.08% (58.38% to 252.54%) | 124.37% (30.51% to 215.19%) | 278.03% (99.93% to 414.58%) |
| Tracheal, bronchus, and lung cancer | DALYs | All age number | 51924.6 (41381.64 to 64801.88) | 7473.85 (5338.66 to 10455.81) | 44450.75 (35718.51 to 55719.0) | 133786.87 (117026.43 to 151661.01) | 27117.81 (20198.36 to 34470.92) | 106669.06 (94368.58 to 120582.96) | 157.66% (100.24% to 239.0%) | 262.84% (145.56% to 426.68%) | 139.97% (81.64% to 216.76%) |
|  |  | Age-standardized rate (per 100,000) | 181.39 (144.52 to 227.54) | 54.62 (38.97 to 75.59) | 298.39 (240.06 to 375.65) | 163.83 (142.88 to 185.62) | 66.17 (49.57 to 84.12) | 262.73 (232.11 to 296.81) | -9.68% (-29.94% to 18.49%) | 21.15% (-17.5% to 76.51%) | -11.95% (-33.38% to 16.34%) |
|  | Deaths | All age number | 1842.02 (1469.38 to 2314.09) | 256.66 (182.89 to 354.17) | 1585.37 (1273.99 to 1994.14) | 5193.58 (4497.43 to 5896.76) | 1058.53 (795.88 to 1343.79) | 4135.05 (3648.76 to 4682.84) | 181.95% (118.67% to 272.64%) | 312.43% (180.89% to 503.44%) | 160.83% (97.6% to 247.99%) |
|  |  | Age-standardized rate (per 100,000) | 7.36 (5.87 to 9.25) | 2.23 (1.56 to 3.03) | 12.31 (9.84 to 15.65) | 6.95 (5.97 to 7.91) | 2.86 (2.14 to 3.64) | 11.08 (9.73 to 12.58) | -5.6% (-26.67% to 24.48%) | 28.55% (-10.89% to 89.73%) | -9.93% (-31.81% to 19.88%) |
|  | YLDs | All age number | 418.23 (288.86 to 598.08) | 60.82 (37.48 to 93.93) | 357.4 (247.96 to 521.8) | 1144.65 (806.0 to 1509.42) | 235.08 (153.35 to 326.4) | 909.57 (631.8 to 1186.05) | 173.69% (111.18% to 258.49%) | 286.49% (161.92% to 466.24%) | 154.49% (88.82% to 238.55%) |
|  |  | Age-standardized rate (per 100,000) | 1.55 (1.07 to 2.22) | 0.48 (0.3 to 0.75) | 2.55 (1.77 to 3.7) | 1.46 (1.03 to 1.92) | 0.6 (0.39 to 0.83) | 2.33 (1.64 to 3.05) | -5.74% (-27.23% to 23.5%) | 25.09% (-14.83% to 84.35%) | -8.68% (-32.23% to 21.63%) |
|  | YLLs | All age number | 51506.37 (41021.63 to 64289.84) | 7413.03 (5296.16 to 10365.24) | 44093.35 (35423.27 to 55281.78) | 132642.22 (116010.08 to 150233.06) | 26882.73 (20028.43 to 34138.43) | 105759.5 (93446.95 to 119448.02) | 157.53% (100.12% to 238.94%) | 262.64% (145.35% to 426.31%) | 139.85% (81.57% to 216.62%) |
|  |  | Age-standardized rate (per 100,000) | 179.84 (143.27 to 225.53) | 54.14 (38.64 to 74.88) | 295.83 (237.81 to 372.25) | 162.37 (141.59 to 183.89) | 65.57 (49.09 to 83.28) | 260.4 (229.76 to 294.22) | -9.71% (-29.97% to 18.42%) | 21.11% (-17.54% to 76.43%) | -11.98% (-33.39% to 16.31%) |
| Uterine cancer | DALYs | All age number | 830.23 (486.21 to 1189.43) | 830.23 (486.21 to 1189.43) | NA | 4238.39 (1959.32 to 6104.7) | 4238.39 (1959.32 to 6104.7) | NA | 410.51% (250.56% to 598.62%) | 410.51% (250.56% to 598.62%) | NA |
|  |  | Age-standardized rate (per 100,000) | 2.78 (1.62 to 4.01) | 5.81 (3.39 to 8.36) | NA | 5.04 (2.38 to 7.23) | 10.04 (4.74 to 14.43) | NA | 80.93% (25.23% to 145.26%) | 72.83% (19.67% to 133.92%) | NA |
|  | Deaths | All age number | 25.53 (15.24 to 36.61) | 25.53 (15.24 to 36.61) | NA | 136.53 (66.17 to 192.9) | 136.53 (66.17 to 192.9) | NA | 434.84% (277.77% to 619.45%) | 434.84% (277.77% to 619.45%) | NA |
|  |  | Age-standardized rate (per 100,000) | 0.1 (0.06 to 0.14) | 0.2 (0.12 to 0.29) | NA | 0.18 (0.09 to 0.25) | 0.35 (0.17 to 0.5) | NA | 80.37% (29.47% to 142.98%) | 75.19% (25.57% to 136.4%) | NA |
|  | YLDs | All age number | 58.86 (30.13 to 95.85) | 58.86 (30.13 to 95.85) | NA | 525.19 (218.92 to 853.58) | 525.19 (218.92 to 853.58) | NA | 792.3% (518.06% to 1163.51%) | 792.3% (518.06% to 1163.51%) | NA |
|  |  | Age-standardized rate (per 100,000) | 0.19 (0.1 to 0.31) | 0.4 (0.21 to 0.65) | NA | 0.61 (0.26 to 0.98) | 1.22 (0.51 to 1.95) | NA | 217.71% (120.77% to 346.1%) | 201.15% (109.43% to 322.37%) | NA |
|  | YLLs | All age number | 771.37 (453.65 to 1111.87) | 771.37 (453.65 to 1111.87) | NA | 3713.2 (1735.14 to 5287.76) | 3713.2 (1735.14 to 5287.76) | NA | 381.38% (231.56% to 554.32%) | 381.38% (231.56% to 554.32%) | NA |
|  |  | Age-standardized rate (per 100,000) | 2.59 (1.54 to 3.73) | 5.41 (3.21 to 7.78) | NA | 4.43 (2.1 to 6.27) | 8.83 (4.19 to 12.51) | NA | 70.81% (18.95% to 129.69%) | 63.25% (13.39% to 119.63%) | NA |

**S2 Table Footnote:** GBD 2021 did not estimate any burden attributable to risk factors for the following 11 level 3 cancers: Brain and central nervous system cancer, Eye cancer, Hodgkin lymphoma, Malignant neoplasm of bone and articular cartilage, Malignant skin melanoma, Neuroblastoma and other peripheral nervous cell tumors, Non-melanoma skin cancer, Other malignant neoplasms, Other neoplasms, Soft tissue and other extraosseous sarcomas, and Testicular cancer. The risk-factor-attributable burden of cervical cancer, ovarian cancer, and uterine cancer was not estimated for males, and the risk-factor-attributable burden of prostate cancer was not estimated for females. DALYs: Disability-Adjusted Life Years. YLDs: Years Lived with Disability. YLLs: Years of Life Lost.
