# Supplementary material for: Temporal trend in the national and sub-national burden of cancers attributable to risk factors in Iran from 1990 to 2021: Findings from the global burden of disease study 2021
Source: PLoS One. 2025 Aug 26;20(8):e0330993. doi: 10.1371/journal.pone.0330993 (PMC12380304; doi:10.1371/journal.pone.0330993)
Supplement: S3 Table — DALYs: Disability-Adjusted Life Years. YLDs: Years Lived with Disability. YLLs: Years of Life Lost. (DOCX) [file pone.0330993.s003.docx]

**Temporal trend in the national and sub-national burden of cancers attributable to risk factors in Iran from 1990 to 2021: findings from the Global Burden of Disease Study 2021**

Seyede Maryam Mousavi^1, 2^¶, Sobhan Younesian^1,2^¶, Saba Katebian^1^, Ali Golestani^1^, Shaghayegh Khanmohammadi^1,3^, Sepehr Khosravi^1^, Yasaman Etemadi^1^, Nazila Rezaei^1^, Sina Azadnajafabad^1*^, Bagher Larijani^4*^

**Authors’ affiliations:**

1. **Non-Communicable Diseases Research Center, Endocrinology and Metabolism Population Sciences Institute, Tehran University of Medical Sciences, Tehran, Iran**
2. **School of Medicine, Tehran University of Medical Sciences, Tehran, Iran**
3. **Research Center for Immunodeficiencies, Pediatrics Center of Excellence, Children’s Medical Center, Tehran University of Medical Sciences, Tehran, Iran**
4. **Endocrinology and Metabolism Research Center, Endocrinology and Metabolism Clinical Sciences Institute, Tehran University of Medical Sciences, Tehran, Iran**

***Corresponding authors:**

Sina Azadnajafabad (E-mail: [sina.azad.u@gmail.com](mailto:sina.azad.u@gmail.com))

Bagher Larijani (E-mail: [emrc@tums.ac.ir](mailto:emrc@tums.ac.ir))

¶ These authors contributed equally to this work.

Supplementary methods and results to “Temporal trend in the national and sub-national burden of cancers attributable to risk factors in Iran from 1990 to 2021: findings from the Global Burden of Disease Study 2021”

**S3 Table Title:** DALYs, deaths, YLDs, and YLLs of cancer attributable to all and level 1 risk factors by sex at the subnational level in Iran in 1990 and 2021 and their percent change.

| **Location** | **Risk factors** | **Measure** | **Age, Metric** | **Year** | | | | | | **Percent Change (1990-2021)** | | |
| --- | --- | --- | --- | --- | --- | --- | --- | --- | --- | --- | --- | --- |
|  |  |  |  | **1990** | | | **2021** | | |  |  |  |
|  |  |  |  | **Both** | **Female** | **Male** | **Both** | **Female** | **Male** | **Both** | **Female** | **Male** |
| Alborz | All risk factors | DALYs | All age number | 3687.12 (2606.66 to 4906.87) | 1430.17 (824.42 to 2097.34) | 2256.95 (1562.01 to 3105.03) | 14927.01 (10634.85 to 19740.86) | 5732.11 (3524.8 to 8265.62) | 9194.9 (6724.93 to 12254.26) | 304.84% (195.54% to 453.92%) | 300.8% (163.88% to 527.99%) | 307.4% (171.84% to 532.11%) |
|  |  |  | Age-standardized rate (per 100,000) | 555.42 (393.29 to 736.09) | 439.53 (255.47 to 627.63) | 664.52 (463.18 to 902.73) | 528.04 (375.72 to 697.82) | 415.74 (258.49 to 596.09) | 638.94 (468.11 to 854.63) | -4.93% (-28.7% to 29.59%) | -5.41% (-37.0% to 44.13%) | -3.85% (-35.42% to 44.62%) |
|  |  | Deaths | All age number | 124.28 (88.35 to 165.54) | 45.19 (26.39 to 64.49) | 79.1 (54.96 to 107.88) | 542.49 (387.33 to 719.49) | 198.37 (123.31 to 281.66) | 344.12 (253.15 to 460.86) | 336.49% (225.15% to 497.46%) | 338.97% (195.43% to 567.37%) | 335.07% (193.95% to 556.11%) |
|  |  |  | Age-standardized rate (per 100,000) | 22.33 (15.88 to 29.61) | 16.87 (10.16 to 23.57) | 27.88 (19.8 to 37.03) | 22.66 (16.14 to 29.86) | 17.91 (11.14 to 25.44) | 27.49 (20.2 to 36.93) | 1.48% (-23.2% to 35.83%) | 6.19% (-25.89% to 56.06%) | -1.41% (-32.29% to 45.84%) |
|  |  | YLDs | All age number | 74.78 (43.3 to 109.19) | 40.41 (19.04 to 65.03) | 34.36 (20.59 to 49.53) | 538.04 (294.48 to 843.58) | 323.44 (143.96 to 554.56) | 214.6 (136.08 to 315.3) | 619.54% (417.74% to 879.0%) | 700.36% (426.68% to 1090.25%) | 524.5% (325.92% to 843.07%) |
|  |  |  | Age-standardized rate (per 100,000) | 11.32 (6.6 to 16.3) | 12.25 (5.95 to 19.67) | 10.67 (6.49 to 15.18) | 18.46 (10.46 to 28.69) | 21.85 (9.82 to 36.55) | 15.27 (9.84 to 22.16) | 63.05% (20.05% to 116.29%) | 78.43% (21.12% to 159.87%) | 43.06% (-1.89% to 111.48%) |
|  |  | YLLs | All age number | 3612.34 (2562.13 to 4812.77) | 1389.75 (804.8 to 2040.21) | 2222.59 (1539.88 to 3055.73) | 14388.97 (10270.4 to 18963.85) | 5408.67 (3342.56 to 7761.47) | 8980.3 (6595.34 to 11997.26) | 298.33% (191.47% to 445.87%) | 289.18% (156.85% to 510.35%) | 304.05% (170.13% to 526.82%) |
|  |  |  | Age-standardized rate (per 100,000) | 544.09 (385.34 to 721.22) | 427.29 (248.95 to 610.54) | 653.85 (456.18 to 888.14) | 509.58 (363.2 to 675.6) | 393.89 (245.92 to 560.65) | 623.67 (458.55 to 836.36) | -6.34% (-29.77% to 27.67%) | -7.82% (-38.33% to 40.05%) | -4.62% (-35.97% to 43.62%) |
|  | Behavioral risks | DALYs | All age number | 3041.73 (2080.99 to 4203.89) | 1075.18 (580.62 to 1640.9) | 1966.55 (1345.68 to 2812.16) | 10616.66 (7573.2 to 14545.44) | 3325.09 (1669.74 to 5006.25) | 7291.58 (5380.34 to 10072.47) | 249.03% (162.21% to 374.47%) | 209.26% (106.74% to 380.89%) | 270.78% (150.11% to 458.08%) |
|  |  |  | Age-standardized rate (per 100,000) | 458.37 (316.51 to 636.7) | 325.18 (177.58 to 492.38) | 582.42 (398.56 to 822.01) | 372.11 (267.91 to 505.14) | 232.13 (124.76 to 343.41) | 506.73 (375.17 to 700.0) | -18.82% (-39.07% to 9.12%) | -28.61% (-51.28% to 8.96%) | -13.0% (-40.86% to 28.1%) |
|  |  | Deaths | All age number | 102.57 (70.83 to 143.1) | 33.17 (18.22 to 49.95) | 69.4 (47.38 to 99.42) | 383.58 (278.42 to 521.43) | 109.65 (60.1 to 160.8) | 273.93 (201.59 to 378.78) | 273.96% (179.13% to 403.43%) | 230.52% (130.72% to 400.92%) | 294.72% (168.82% to 486.76%) |
|  |  |  | Age-standardized rate (per 100,000) | 18.32 (12.83 to 25.31) | 12.22 (6.73 to 18.12) | 24.45 (16.68 to 34.04) | 15.9 (11.62 to 21.52) | 9.65 (5.41 to 14.02) | 21.83 (15.91 to 30.21) | -13.24% (-33.83% to 14.42%) | -21.09% (-44.04% to 14.01%) | -10.72% (-37.93% to 28.98%) |
|  |  | YLDs | All age number | 60.91 (33.44 to 91.83) | 30.63 (12.72 to 52.5) | 30.27 (18.07 to 44.04) | 364.81 (187.36 to 592.67) | 195.92 (64.13 to 351.53) | 168.89 (109.51 to 255.48) | 498.97% (335.77% to 714.71%) | 539.54% (296.17% to 885.08%) | 457.91% (276.54% to 736.36%) |
|  |  |  | Age-standardized rate (per 100,000) | 9.13 (5.26 to 13.4) | 8.95 (3.74 to 15.17) | 9.42 (5.68 to 13.72) | 12.3 (6.68 to 19.67) | 12.57 (4.26 to 22.1) | 12.06 (7.82 to 18.02) | 34.85% (0.66% to 76.32%) | 40.39% (-11.28% to 107.47%) | 28.05% (-11.2% to 86.66%) |
|  |  | YLLs | All age number | 2980.82 (2038.83 to 4124.44) | 1044.55 (567.11 to 1591.36) | 1936.28 (1323.72 to 2768.58) | 10251.85 (7345.08 to 14068.92) | 3129.17 (1609.01 to 4688.64) | 7122.68 (5239.96 to 9847.53) | 243.93% (157.05% to 367.71%) | 199.57% (103.21% to 364.31%) | 267.85% (147.7% to 453.71%) |
|  |  |  | Age-standardized rate (per 100,000) | 449.24 (310.18 to 624.87) | 316.23 (173.26 to 478.54) | 573.0 (390.45 to 809.06) | 359.8 (260.51 to 490.26) | 219.57 (120.4 to 323.56) | 494.66 (365.16 to 685.74) | -19.91% (-40.07% to 7.75%) | -30.57% (-52.01% to 5.95%) | -13.67% (-41.33% to 27.13%) |
|  | Environmental/occupational risks | DALYs | All age number | 481.8 (292.48 to 727.35) | 109.96 (60.28 to 184.56) | 371.83 (215.76 to 578.73) | 1946.16 (1267.74 to 2826.09) | 525.59 (292.59 to 864.05) | 1420.57 (910.31 to 2084.58) | 303.94% (174.84% to 523.36%) | 377.96% (158.54% to 696.61%) | 282.05% (138.04% to 540.13%) |
|  |  |  | Age-standardized rate (per 100,000) | 72.06 (44.15 to 108.88) | 34.94 (19.14 to 58.54) | 105.56 (61.19 to 164.46) | 68.52 (44.31 to 100.65) | 39.97 (21.96 to 65.32) | 96.28 (62.04 to 140.89) | -4.91% (-35.62% to 45.05%) | 14.38% (-35.96% to 92.4%) | -8.8% (-43.59% to 51.26%) |
|  |  | Deaths | All age number | 16.37 (9.95 to 24.84) | 3.72 (2.03 to 6.24) | 12.65 (7.33 to 19.78) | 71.98 (46.54 to 106.07) | 19.98 (10.89 to 32.59) | 52.0 (33.32 to 76.6) | 339.83% (197.15% to 579.51%) | 437.66% (200.67% to 803.83%) | 311.09% (152.88% to 581.21%) |
|  |  |  | Age-standardized rate (per 100,000) | 2.9 (1.76 to 4.43) | 1.46 (0.8 to 2.42) | 4.29 (2.51 to 6.74) | 2.98 (1.89 to 4.41) | 1.89 (1.05 to 3.05) | 4.03 (2.57 to 5.88) | 2.71% (-30.84% to 56.84%) | 30.16% (-25.78% to 119.14%) | -6.08% (-41.56% to 53.35%) |
|  |  | YLDs | All age number | 3.98 (2.21 to 6.4) | 0.89 (0.44 to 1.55) | 3.09 (1.67 to 5.21) | 17.82 (10.59 to 27.64) | 4.73 (2.28 to 7.87) | 13.09 (7.78 to 20.2) | 347.68% (193.34% to 597.08%) | 433.34% (193.3% to 805.89%) | 323.15% (154.58% to 610.19%) |
|  |  |  | Age-standardized rate (per 100,000) | 0.64 (0.36 to 1.04) | 0.31 (0.15 to 0.54) | 0.95 (0.52 to 1.6) | 0.67 (0.39 to 1.02) | 0.39 (0.19 to 0.65) | 0.93 (0.54 to 1.47) | 4.13% (-31.62% to 60.62%) | 25.86% (-30.97% to 116.33%) | -1.28% (-40.45% to 64.31%) |
|  |  | YLLs | All age number | 477.82 (289.69 to 722.7) | 109.08 (59.77 to 182.81) | 368.74 (213.89 to 573.6) | 1928.34 (1254.39 to 2801.15) | 520.86 (290.04 to 856.57) | 1407.48 (902.54 to 2063.55) | 303.57% (174.55% to 523.33%) | 377.51% (158.27% to 697.1%) | 281.7% (137.61% to 540.62%) |
|  |  |  | Age-standardized rate (per 100,000) | 71.42 (43.7 to 108.08) | 34.63 (18.97 to 57.99) | 104.62 (60.64 to 163.32) | 67.86 (43.85 to 99.76) | 39.58 (21.71 to 64.72) | 95.34 (61.45 to 139.85) | -4.99% (-35.67% to 45.11%) | 14.28% (-36.1% to 92.22%) | -8.87% (-43.61% to 51.3%) |
|  | Metabolic risks | DALYs | All age number | 567.19 (215.17 to 959.93) | 330.27 (122.35 to 588.32) | 236.93 (89.48 to 410.69) | 4381.88 (1363.88 to 7526.86) | 2391.55 (733.5 to 4238.01) | 1990.33 (609.28 to 3544.91) | 672.56% (427.38% to 973.73%) | 624.13% (363.34% to 1002.14%) | 740.06% (455.95% to 1152.81%) |
|  |  |  | Age-standardized rate (per 100,000) | 86.96 (32.46 to 151.73) | 108.27 (37.1 to 195.7) | 68.3 (23.94 to 119.7) | 159.22 (47.85 to 275.17) | 182.78 (54.76 to 323.31) | 139.35 (40.89 to 251.88) | 83.09% (31.18% to 141.52%) | 68.82% (13.4% to 148.46%) | 104.03% (36.93% to 199.61%) |
|  |  | Deaths | All age number | 19.41 (7.27 to 34.1) | 11.31 (3.94 to 20.54) | 8.11 (2.83 to 14.26) | 162.11 (47.44 to 281.7) | 87.92 (25.63 to 155.19) | 74.19 (21.17 to 134.11) | 734.98% (486.27% to 1001.63%) | 677.43% (426.86% to 1034.31%) | 815.26% (499.13% to 1261.26%) |
|  |  |  | Age-standardized rate (per 100,000) | 3.59 (1.25 to 6.37) | 4.38 (1.47 to 7.97) | 2.85 (0.95 to 5.06) | 6.9 (1.97 to 11.96) | 8.16 (2.37 to 14.34) | 6.0 (1.64 to 11.01) | 92.16% (39.88% to 143.33%) | 86.48% (30.59% to 164.71%) | 110.41% (42.07% to 205.45%) |
|  |  | YLDs | All age number | 15.43 (5.24 to 27.47) | 10.91 (3.64 to 19.69) | 4.52 (1.66 to 8.22) | 204.46 (59.98 to 374.16) | 146.83 (35.95 to 277.08) | 57.63 (20.22 to 104.45) | 1224.67% (827.15% to 1744.43%) | 1245.23% (761.05% to 1861.03%) | 1175.0% (760.68% to 1826.1%) |
|  |  |  | Age-standardized rate (per 100,000) | 2.46 (0.75 to 4.44) | 3.71 (1.08 to 6.83) | 1.39 (0.5 to 2.53) | 7.31 (2.01 to 13.65) | 10.72 (2.48 to 19.86) | 4.08 (1.34 to 7.47) | 196.49% (113.57% to 298.84%) | 188.96% (94.91% to 332.89%) | 193.54% (99.94% to 336.85%) |
|  |  | YLLs | All age number | 551.76 (210.28 to 935.06) | 319.35 (118.6 to 570.86) | 232.41 (87.64 to 403.65) | 4177.43 (1310.48 to 7168.34) | 2244.72 (683.57 to 3954.91) | 1932.71 (590.1 to 3443.19) | 657.11% (414.9% to 945.4%) | 602.9% (351.46% to 975.94%) | 731.6% (448.49% to 1143.85%) |
|  |  |  | Age-standardized rate (per 100,000) | 84.5 (31.7 to 147.77) | 104.56 (36.01 to 189.57) | 66.91 (23.34 to 117.5) | 151.91 (45.98 to 262.6) | 172.06 (51.31 to 302.19) | 135.27 (39.65 to 243.9) | 79.78% (28.14% to 137.39%) | 64.56% (11.04% to 143.12%) | 102.17% (35.47% to 197.15%) |
| Ardebil | All risk factors | DALYs | All age number | 3515.68 (2462.92 to 5379.34) | 1110.19 (676.89 to 1738.68) | 2405.49 (1576.17 to 3757.85) | 9009.18 (6905.2 to 11847.17) | 2673.87 (1747.43 to 3759.41) | 6335.31 (4906.62 to 8136.54) | 156.26% (93.81% to 238.67%) | 140.85% (65.49% to 234.1%) | 163.37% (90.19% to 291.54%) |
|  |  |  | Age-standardized rate (per 100,000) | 633.35 (436.53 to 976.55) | 408.38 (249.9 to 644.52) | 816.21 (532.81 to 1277.9) | 705.93 (539.15 to 929.93) | 396.29 (258.65 to 555.0) | 1052.58 (820.49 to 1360.6) | 11.46% (-15.8% to 47.29%) | -2.96% (-34.13% to 34.49%) | 28.96% (-6.34% to 90.03%) |
|  |  | Deaths | All age number | 119.34 (82.11 to 184.45) | 33.97 (21.03 to 53.83) | 85.37 (56.02 to 133.5) | 342.44 (261.56 to 449.46) | 97.17 (63.28 to 137.89) | 245.27 (190.6 to 317.64) | 186.94% (114.42% to 283.05%) | 186.03% (95.18% to 294.62%) | 187.3% (110.21% to 327.02%) |
|  |  |  | Age-standardized rate (per 100,000) | 25.15 (17.23 to 38.8) | 15.0 (9.08 to 24.09) | 33.68 (22.29 to 52.94) | 29.25 (22.48 to 38.46) | 15.48 (10.06 to 22.09) | 45.28 (35.13 to 58.77) | 16.3% (-11.3% to 53.51%) | 3.19% (-28.93% to 45.85%) | 34.43% (-1.28% to 95.42%) |
|  |  | YLDs | All age number | 48.24 (30.91 to 72.2) | 20.01 (10.87 to 31.79) | 28.23 (16.7 to 44.09) | 201.9 (127.47 to 291.24) | 95.57 (49.66 to 145.95) | 106.34 (71.71 to 151.47) | 318.51% (213.72% to 446.1%) | 377.62% (229.52% to 556.22%) | 276.62% (169.53% to 447.11%) |
|  |  |  | Age-standardized rate (per 100,000) | 8.95 (5.78 to 13.34) | 7.43 (3.99 to 11.97) | 10.07 (6.13 to 15.66) | 15.64 (10.02 to 22.31) | 13.82 (7.19 to 21.0) | 18.08 (12.23 to 25.68) | 74.69% (30.84% to 124.75%) | 85.86% (29.36% to 152.04%) | 79.58% (30.34% to 159.5%) |
|  |  | YLLs | All age number | 3467.44 (2425.71 to 5320.97) | 1090.18 (664.4 to 1711.49) | 2377.26 (1558.17 to 3715.88) | 8807.28 (6775.01 to 11581.82) | 2578.3 (1693.96 to 3605.11) | 6228.98 (4832.89 to 7999.05) | 154.0% (91.88% to 236.51%) | 136.5% (62.25% to 228.26%) | 162.02% (89.09% to 289.67%) |
|  |  |  | Age-standardized rate (per 100,000) | 624.4 (429.97 to 965.61) | 400.95 (245.45 to 634.82) | 806.14 (526.23 to 1263.28) | 690.29 (528.92 to 908.73) | 382.47 (250.68 to 533.02) | 1034.49 (807.28 to 1338.29) | 10.55% (-16.57% to 46.25%) | -4.61% (-35.4% to 32.29%) | 28.33% (-6.85% to 89.11%) |
|  | Behavioral risks | DALYs | All age number | 3100.82 (2083.61 to 4899.1) | 903.63 (475.55 to 1518.85) | 2197.19 (1394.18 to 3492.32) | 7080.08 (5214.07 to 9527.46) | 1698.19 (1028.13 to 2598.06) | 5381.89 (4047.87 to 7100.48) | 128.33% (72.75% to 207.24%) | 87.93% (32.33% to 165.35%) | 144.94% (77.05% to 261.78%) |
|  |  |  | Age-standardized rate (per 100,000) | 560.55 (373.5 to 886.27) | 331.67 (171.49 to 564.23) | 746.83 (481.92 to 1180.01) | 554.5 (408.48 to 746.84) | 248.41 (149.47 to 380.92) | 896.52 (678.91 to 1181.83) | -1.08% (-25.02% to 32.72%) | -25.1% (-47.04% to 4.22%) | 20.04% (-12.97% to 76.75%) |
|  |  | Deaths | All age number | 105.92 (70.73 to 167.59) | 27.53 (14.25 to 47.05) | 78.4 (50.1 to 124.03) | 269.46 (200.3 to 365.72) | 60.06 (35.71 to 92.21) | 209.39 (158.78 to 277.14) | 154.39% (92.53% to 241.42%) | 118.19% (55.38% to 199.97%) | 167.1% (92.45% to 296.37%) |
|  |  |  | Age-standardized rate (per 100,000) | 22.31 (14.56 to 34.73) | 12.1 (6.21 to 20.96) | 30.87 (20.06 to 48.26) | 22.98 (17.2 to 31.21) | 9.48 (5.61 to 14.59) | 38.66 (29.02 to 51.27) | 2.99% (-20.22% to 37.54%) | -21.71% (-43.62% to 9.68%) | 25.22% (-8.62% to 79.4%) |
|  |  | YLDs | All age number | 41.99 (25.49 to 65.14) | 16.12 (7.86 to 26.25) | 25.87 (15.23 to 41.28) | 149.47 (91.55 to 223.91) | 61.29 (26.67 to 104.15) | 88.18 (59.14 to 125.76) | 255.96% (169.85% to 365.47%) | 280.27% (140.93% to 429.66%) | 240.82% (145.49% to 392.78%) |
|  |  |  | Age-standardized rate (per 100,000) | 7.81 (4.76 to 12.19) | 5.93 (2.93 to 9.66) | 9.22 (5.52 to 14.46) | 11.53 (7.19 to 17.12) | 8.65 (3.85 to 14.64) | 15.07 (10.23 to 21.39) | 47.76% (13.09% to 91.07%) | 45.92% (-4.76% to 97.35%) | 63.37% (18.51% to 133.22%) |
|  |  | YLLs | All age number | 3058.83 (2058.08 to 4847.05) | 887.51 (468.45 to 1497.24) | 2171.32 (1376.99 to 3461.0) | 6930.61 (5113.09 to 9342.92) | 1636.9 (993.78 to 2491.7) | 5293.71 (3979.55 to 6986.17) | 126.58% (71.1% to 204.68%) | 84.44% (30.07% to 161.12%) | 143.8% (76.16% to 260.07%) |
|  |  |  | Age-standardized rate (per 100,000) | 552.74 (368.6 to 873.66) | 325.74 (168.58 to 555.24) | 737.6 (475.03 to 1167.7) | 542.97 (400.08 to 732.87) | 239.75 (144.65 to 366.03) | 881.46 (668.39 to 1164.09) | -1.77% (-25.58% to 31.85%) | -26.4% (-47.87% to 2.63%) | 19.5% (-13.39% to 75.89%) |
|  | Environmental/occupational risks | DALYs | All age number | 401.33 (222.76 to 700.85) | 81.3 (37.0 to 150.27) | 320.04 (172.43 to 537.88) | 1203.68 (700.04 to 1893.07) | 291.86 (137.37 to 499.34) | 911.82 (558.43 to 1413.5) | 199.92% (93.98% to 380.8%) | 259.0% (79.91% to 634.28%) | 184.91% (75.27% to 360.57%) |
|  |  |  | Age-standardized rate (per 100,000) | 71.01 (39.2 to 124.99) | 30.84 (13.95 to 56.69) | 104.92 (56.71 to 178.8) | 93.32 (53.12 to 146.65) | 43.72 (20.48 to 75.28) | 147.25 (88.66 to 229.81) | 31.42% (-16.44% to 113.96%) | 41.74% (-28.77% to 191.35%) | 40.35% (-12.74% to 126.39%) |
|  |  | Deaths | All age number | 13.56 (7.46 to 24.01) | 2.6 (1.17 to 4.78) | 10.95 (5.8 to 18.76) | 44.98 (25.03 to 71.09) | 11.26 (5.28 to 19.42) | 33.72 (20.14 to 52.8) | 231.81% (110.19% to 443.0%) | 332.75% (117.77% to 783.42%) | 207.83% (93.61% to 397.99%) |
|  |  |  | Age-standardized rate (per 100,000) | 2.8 (1.54 to 4.89) | 1.2 (0.53 to 2.16) | 4.16 (2.2 to 7.17) | 3.8 (2.08 to 6.08) | 1.81 (0.85 to 3.14) | 6.05 (3.59 to 9.48) | 35.4% (-15.35% to 124.37%) | 50.69% (-24.26% to 211.02%) | 45.23% (-8.6% to 139.02%) |
|  |  | YLDs | All age number | 3.3 (1.62 to 5.93) | 0.65 (0.26 to 1.27) | 2.65 (1.28 to 4.81) | 10.59 (5.31 to 17.56) | 2.53 (1.13 to 4.52) | 8.05 (4.17 to 13.41) | 221.18% (98.94% to 427.46%) | 289.3% (91.29% to 742.78%) | 204.43% (82.26% to 417.29%) |
|  |  |  | Age-standardized rate (per 100,000) | 0.62 (0.31 to 1.15) | 0.27 (0.11 to 0.54) | 0.92 (0.45 to 1.66) | 0.85 (0.42 to 1.42) | 0.39 (0.17 to 0.7) | 1.35 (0.7 to 2.25) | 35.83% (-15.99% to 123.03%) | 44.13% (-31.19% to 223.83%) | 46.44% (-14.07% to 143.8%) |
|  |  | YLLs | All age number | 398.04 (221.13 to 694.27) | 80.65 (36.67 to 148.96) | 317.39 (170.93 to 533.03) | 1193.1 (694.78 to 1876.51) | 289.33 (136.33 to 495.59) | 903.77 (553.09 to 1401.89) | 199.75% (93.7% to 380.64%) | 258.76% (79.71% to 634.1%) | 184.75% (75.32% to 360.75%) |
|  |  |  | Age-standardized rate (per 100,000) | 70.39 (38.91 to 123.94) | 30.57 (13.82 to 56.17) | 104.0 (56.18 to 177.3) | 92.47 (52.7 to 145.35) | 43.33 (20.3 to 74.57) | 145.9 (87.91 to 227.29) | 31.38% (-16.43% to 114.0%) | 41.72% (-28.81% to 191.3%) | 40.29% (-12.86% to 126.6%) |
|  | Metabolic risks | DALYs | All age number | 303.87 (133.61 to 486.03) | 171.47 (80.86 to 277.27) | 132.41 (54.32 to 224.93) | 1816.4 (681.37 to 3012.88) | 895.58 (313.37 to 1518.24) | 920.82 (336.03 to 1568.64) | 497.75% (304.38% to 662.03%) | 422.31% (220.88% to 650.73%) | 595.45% (361.93% to 893.3%) |
|  |  |  | Age-standardized rate (per 100,000) | 53.21 (22.41 to 86.58) | 63.63 (28.78 to 103.64) | 44.13 (17.61 to 75.27) | 143.65 (52.22 to 240.74) | 136.37 (46.83 to 232.6) | 152.63 (53.09 to 261.43) | 169.97% (87.77% to 242.97%) | 114.31% (35.62% to 198.21%) | 245.89% (131.37% to 394.08%) |
|  |  | Deaths | All age number | 9.87 (4.02 to 16.15) | 5.37 (2.41 to 8.8) | 4.5 (1.74 to 7.78) | 69.24 (24.08 to 116.96) | 34.04 (11.24 to 57.91) | 35.2 (11.92 to 60.59) | 601.58% (387.57% to 794.01%) | 533.83% (299.83% to 776.79%) | 682.44% (423.7% to 1017.91%) |
|  |  |  | Age-standardized rate (per 100,000) | 2.05 (0.81 to 3.35) | 2.38 (1.02 to 3.91) | 1.78 (0.66 to 3.07) | 5.97 (2.02 to 10.14) | 5.51 (1.79 to 9.39) | 6.53 (2.18 to 11.4) | 190.61% (110.78% to 268.12%) | 131.5% (51.8% to 215.08%) | 267.75% (152.85% to 419.09%) |
|  |  | YLDs | All age number | 6.14 (2.42 to 10.38) | 4.07 (1.51 to 7.27) | 2.07 (0.82 to 3.78) | 59.96 (21.16 to 103.33) | 38.35 (12.18 to 67.23) | 21.61 (8.86 to 38.81) | 875.9% (617.87% to 1154.65%) | 841.37% (520.0% to 1208.59%) | 943.85% (644.79% to 1380.76%) |
|  |  |  | Age-standardized rate (per 100,000) | 1.12 (0.43 to 1.89) | 1.57 (0.55 to 2.8) | 0.74 (0.29 to 1.31) | 4.72 (1.62 to 8.15) | 5.79 (1.78 to 10.21) | 3.63 (1.44 to 6.6) | 322.16% (213.21% to 432.3%) | 269.02% (145.32% to 405.22%) | 392.59% (256.51% to 597.15%) |
|  |  | YLLs | All age number | 297.73 (131.23 to 475.7) | 167.39 (79.44 to 269.8) | 130.34 (53.45 to 221.21) | 1756.44 (663.0 to 2921.36) | 857.23 (301.56 to 1450.73) | 899.22 (326.54 to 1532.74) | 489.95% (299.19% to 654.63%) | 412.11% (214.66% to 636.96%) | 589.92% (357.63% to 887.9%) |
|  |  |  | Age-standardized rate (per 100,000) | 52.09 (22.0 to 84.56) | 62.06 (28.14 to 100.91) | 43.39 (17.32 to 74.13) | 138.93 (50.76 to 232.86) | 130.58 (45.04 to 223.07) | 149.0 (51.55 to 255.02) | 166.7% (85.29% to 239.25%) | 110.4% (32.03% to 193.97%) | 243.4% (129.11% to 391.48%) |
| Bushehr | All risk factors | DALYs | All age number | 1492.94 (1106.9 to 1945.9) | 540.86 (370.5 to 745.05) | 952.08 (680.42 to 1268.22) | 5054.79 (3996.2 to 6263.58) | 1975.07 (1335.82 to 2640.05) | 3079.72 (2399.64 to 3884.64) | 238.58% (165.24% to 354.31%) | 265.17% (159.15% to 412.17%) | 223.47% (138.0% to 374.73%) |
|  |  |  | Age-standardized rate (per 100,000) | 492.18 (366.14 to 634.84) | 343.11 (238.63 to 465.59) | 633.29 (461.19 to 835.87) | 509.94 (403.78 to 633.43) | 384.67 (261.55 to 513.67) | 641.64 (501.0 to 809.8) | 3.61% (-18.66% to 37.84%) | 12.11% (-18.91% to 56.66%) | 1.32% (-25.04% to 46.14%) |
|  |  | Deaths | All age number | 49.96 (37.33 to 64.46) | 16.4 (11.48 to 22.36) | 33.56 (24.33 to 44.52) | 182.02 (144.35 to 225.62) | 66.09 (45.02 to 87.88) | 115.93 (91.02 to 145.48) | 264.31% (185.54% to 385.63%) | 302.91% (192.38% to 460.59%) | 245.45% (154.7% to 405.91%) |
|  |  |  | Age-standardized rate (per 100,000) | 19.78 (14.88 to 25.42) | 12.64 (8.87 to 17.1) | 26.83 (19.76 to 35.43) | 21.28 (16.71 to 26.42) | 14.84 (10.09 to 19.72) | 28.11 (22.03 to 35.22) | 7.56% (-15.5% to 41.45%) | 17.37% (-13.64% to 62.34%) | 4.75% (-21.92% to 47.9%) |
|  |  | YLDs | All age number | 25.33 (16.33 to 36.79) | 12.01 (6.44 to 18.72) | 13.32 (8.54 to 19.73) | 154.22 (91.86 to 232.29) | 89.39 (43.75 to 145.49) | 64.83 (42.85 to 94.37) | 508.87% (364.1% to 690.76%) | 644.38% (412.93% to 911.83%) | 386.7% (248.59% to 585.78%) |
|  |  |  | Age-standardized rate (per 100,000) | 8.5 (5.54 to 12.24) | 7.65 (4.21 to 11.66) | 9.33 (6.08 to 13.68) | 15.16 (9.36 to 22.39) | 16.77 (8.25 to 27.07) | 13.93 (9.32 to 20.16) | 78.31% (36.46% to 131.84%) | 119.24% (52.24% to 191.92%) | 49.38% (7.5% to 104.31%) |
|  |  | YLLs | All age number | 1467.61 (1088.25 to 1914.37) | 528.85 (362.07 to 729.6) | 938.76 (670.72 to 1251.2) | 4900.57 (3895.53 to 6098.52) | 1885.68 (1284.17 to 2509.56) | 3014.89 (2350.85 to 3797.07) | 233.91% (162.11% to 349.27%) | 256.56% (152.81% to 400.91%) | 221.16% (136.41% to 371.82%) |
|  |  |  | Age-standardized rate (per 100,000) | 483.68 (359.47 to 622.48) | 335.46 (234.51 to 456.16) | 623.96 (453.97 to 821.87) | 494.77 (393.49 to 614.1) | 367.9 (250.66 to 489.02) | 627.71 (490.82 to 791.78) | 2.29% (-19.68% to 36.22%) | 9.67% (-20.6% to 53.5%) | 0.6% (-25.61% to 45.42%) |
|  | Behavioral risks | DALYs | All age number | 1210.76 (893.56 to 1612.49) | 394.97 (247.07 to 563.67) | 815.79 (581.14 to 1112.65) | 3615.15 (2731.32 to 4616.47) | 1163.68 (687.16 to 1661.64) | 2451.47 (1894.53 to 3106.34) | 198.59% (132.38% to 305.78%) | 194.63% (98.32% to 308.84%) | 200.5% (120.58% to 348.86%) |
|  |  |  | Age-standardized rate (per 100,000) | 398.72 (295.73 to 528.14) | 245.99 (155.33 to 350.29) | 542.72 (388.99 to 735.85) | 362.75 (276.52 to 464.77) | 218.85 (131.07 to 311.01) | 512.24 (397.78 to 649.33) | -9.02% (-28.64% to 23.85%) | -11.03% (-38.31% to 21.69%) | -5.62% (-30.49% to 36.54%) |
|  |  | Deaths | All age number | 40.56 (30.15 to 53.52) | 11.69 (7.46 to 16.66) | 28.87 (20.75 to 39.25) | 129.45 (98.8 to 165.56) | 36.93 (22.66 to 52.17) | 92.52 (72.0 to 117.42) | 219.14% (149.78% to 337.26%) | 215.84% (121.36% to 329.13%) | 220.47% (137.07% to 374.57%) |
|  |  |  | Age-standardized rate (per 100,000) | 15.87 (11.93 to 21.04) | 8.79 (5.75 to 12.41) | 22.83 (16.49 to 30.91) | 15.0 (11.52 to 19.16) | 8.04 (5.04 to 11.23) | 22.34 (17.24 to 28.2) | -5.5% (-25.57% to 27.57%) | -8.52% (-32.67% to 22.23%) | -2.16% (-26.97% to 39.05%) |
|  |  | YLDs | All age number | 21.06 (12.93 to 31.14) | 9.32 (4.46 to 15.38) | 11.74 (7.56 to 17.6) | 109.06 (61.41 to 171.46) | 56.88 (20.17 to 106.28) | 52.18 (34.26 to 78.18) | 417.8% (274.81% to 578.94%) | 510.42% (269.75% to 753.92%) | 344.33% (216.01% to 538.46%) |
|  |  |  | Age-standardized rate (per 100,000) | 6.98 (4.37 to 10.23) | 5.71 (2.78 to 9.26) | 8.18 (5.28 to 12.15) | 10.48 (6.08 to 15.98) | 10.05 (3.77 to 18.49) | 11.23 (7.45 to 16.74) | 50.06% (12.49% to 97.01%) | 76.12% (11.14% to 141.01%) | 37.32% (0.91% to 94.05%) |
|  |  | YLLs | All age number | 1189.69 (877.69 to 1584.15) | 385.65 (241.32 to 550.62) | 804.05 (572.15 to 1097.99) | 3506.08 (2661.26 to 4490.65) | 1106.8 (665.67 to 1578.25) | 2399.28 (1853.4 to 3040.4) | 194.7% (130.29% to 300.71%) | 187.0% (93.05% to 296.39%) | 198.4% (118.82% to 346.27%) |
|  |  |  | Age-standardized rate (per 100,000) | 391.74 (291.0 to 519.34) | 240.28 (151.94 to 341.68) | 534.54 (382.73 to 723.98) | 352.27 (269.3 to 450.53) | 208.79 (126.88 to 295.14) | 501.0 (388.95 to 636.37) | -10.07% (-29.66% to 21.94%) | -13.1% (-39.42% to 18.43%) | -6.27% (-30.98% to 35.86%) |
|  | Environmental/occupational risks | DALYs | All age number | 324.42 (208.76 to 470.58) | 81.35 (45.64 to 129.04) | 243.07 (156.69 to 356.24) | 1020.98 (655.91 to 1450.85) | 327.34 (184.79 to 489.04) | 693.64 (459.87 to 988.27) | 214.71% (119.43% to 389.6%) | 302.38% (128.83% to 607.23%) | 185.36% (86.31% to 364.54%) |
|  |  |  | Age-standardized rate (per 100,000) | 110.24 (71.52 to 160.61) | 55.49 (31.01 to 86.99) | 162.29 (102.65 to 237.52) | 104.57 (66.94 to 147.52) | 66.24 (36.86 to 99.93) | 143.54 (93.91 to 204.54) | -5.14% (-34.4% to 47.93%) | 19.37% (-31.79% to 111.45%) | -11.55% (-41.18% to 40.61%) |
|  |  | Deaths | All age number | 11.33 (7.36 to 16.54) | 2.71 (1.51 to 4.26) | 8.61 (5.52 to 12.61) | 38.33 (24.42 to 53.88) | 12.11 (6.69 to 18.18) | 26.22 (17.17 to 37.45) | 238.42% (136.53% to 428.16%) | 346.48% (156.1% to 697.16%) | 204.41% (104.68% to 382.86%) |
|  |  |  | Age-standardized rate (per 100,000) | 4.66 (3.02 to 6.71) | 2.29 (1.29 to 3.54) | 7.0 (4.35 to 10.21) | 4.55 (2.89 to 6.4) | 2.83 (1.55 to 4.23) | 6.34 (4.19 to 9.01) | -2.42% (-32.57% to 50.15%) | 23.67% (-27.9% to 120.84%) | -9.5% (-39.29% to 40.98%) |
|  |  | YLDs | All age number | 2.71 (1.54 to 4.32) | 0.66 (0.33 to 1.1) | 2.05 (1.15 to 3.39) | 9.1 (5.17 to 13.71) | 2.8 (1.46 to 4.64) | 6.3 (3.59 to 9.33) | 235.81% (129.23% to 420.46%) | 326.48% (138.56% to 668.27%) | 206.82% (89.61% to 419.35%) |
|  |  |  | Age-standardized rate (per 100,000) | 1.0 (0.57 to 1.59) | 0.49 (0.25 to 0.83) | 1.48 (0.82 to 2.39) | 0.99 (0.56 to 1.5) | 0.6 (0.31 to 0.98) | 1.38 (0.79 to 2.08) | -0.78% (-31.3% to 53.02%) | 22.47% (-34.31% to 128.35%) | -6.68% (-41.39% to 54.83%) |
|  |  | YLLs | All age number | 321.71 (206.97 to 467.25) | 80.7 (45.24 to 128.11) | 241.02 (155.09 to 353.77) | 1011.88 (650.57 to 1438.44) | 324.54 (183.08 to 485.22) | 687.33 (455.3 to 979.45) | 214.53% (119.49% to 389.31%) | 302.18% (128.53% to 606.32%) | 185.18% (86.07% to 363.85%) |
|  |  |  | Age-standardized rate (per 100,000) | 109.24 (70.71 to 159.3) | 55.0 (30.76 to 86.12) | 160.81 (101.7 to 235.74) | 103.58 (66.39 to 146.19) | 65.64 (36.49 to 99.03) | 142.16 (93.03 to 202.69) | -5.18% (-34.44% to 47.95%) | 19.35% (-31.83% to 111.19%) | -11.6% (-41.27% to 40.39%) |
|  | Metabolic risks | DALYs | All age number | 172.25 (72.8 to 286.44) | 95.94 (39.97 to 154.87) | 76.31 (29.03 to 130.84) | 1207.53 (406.17 to 2054.77) | 654.79 (228.61 to 1109.76) | 552.74 (178.03 to 944.49) | 601.03% (398.32% to 800.83%) | 582.51% (325.41% to 846.21%) | 624.31% (387.19% to 933.98%) |
|  |  |  | Age-standardized rate (per 100,000) | 56.11 (21.34 to 95.49) | 63.41 (23.8 to 105.5) | 49.5 (16.75 to 86.56) | 124.59 (39.42 to 214.54) | 135.02 (44.71 to 233.1) | 114.97 (34.03 to 201.13) | 122.03% (62.19% to 180.69%) | 112.92% (40.05% to 187.8%) | 132.29% (61.28% to 229.48%) |
|  |  | Deaths | All age number | 5.67 (2.16 to 9.66) | 3.07 (1.15 to 5.1) | 2.6 (0.88 to 4.52) | 43.98 (13.53 to 75.81) | 23.34 (7.54 to 40.32) | 20.64 (5.99 to 36.27) | 675.74% (468.84% to 876.27%) | 659.68% (399.84% to 929.49%) | 694.73% (446.42% to 1028.4%) |
|  |  |  | Age-standardized rate (per 100,000) | 2.25 (0.76 to 3.9) | 2.44 (0.87 to 4.17) | 2.09 (0.63 to 3.75) | 5.24 (1.51 to 9.18) | 5.43 (1.65 to 9.48) | 5.06 (1.37 to 9.02) | 132.36% (74.33% to 190.95%) | 123.06% (52.37% to 190.46%) | 141.92% (72.7% to 235.58%) |
|  |  | YLDs | All age number | 3.86 (1.26 to 7.1) | 2.57 (0.81 to 4.73) | 1.29 (0.42 to 2.43) | 49.97 (14.09 to 91.95) | 35.3 (9.18 to 64.81) | 14.66 (4.57 to 26.44) | 1194.02% (831.92% to 1624.6%) | 1275.61% (794.79% to 1919.4%) | 1032.3% (681.66% to 1486.38%) |
|  |  |  | Age-standardized rate (per 100,000) | 1.36 (0.41 to 2.53) | 1.85 (0.52 to 3.39) | 0.92 (0.27 to 1.7) | 5.21 (1.35 to 9.63) | 7.32 (1.76 to 13.41) | 3.15 (0.94 to 5.69) | 283.34% (185.15% to 391.97%) | 296.38% (170.4% to 471.41%) | 243.51% (142.05% to 379.88%) |
|  |  | YLLs | All age number | 168.39 (71.27 to 279.63) | 93.37 (39.0 to 149.89) | 75.02 (28.56 to 128.69) | 1157.56 (388.34 to 1955.11) | 619.48 (219.34 to 1048.06) | 538.08 (173.86 to 918.55) | 587.43% (387.17% to 783.4%) | 563.46% (311.2% to 821.0%) | 617.27% (382.5% to 924.49%) |
|  |  |  | Age-standardized rate (per 100,000) | 54.75 (20.91 to 92.95) | 61.57 (23.13 to 102.59) | 48.58 (16.47 to 85.14) | 119.38 (37.74 to 204.15) | 127.7 (42.72 to 219.44) | 111.82 (33.21 to 195.76) | 118.03% (58.68% to 174.96%) | 107.41% (36.75% to 180.55%) | 130.19% (60.06% to 227.07%) |
| Chahar Mahaal and Bakhtiari | All risk factors | DALYs | All age number | 1573.66 (1182.04 to 2289.01) | 453.66 (303.52 to 655.68) | 1120.0 (795.96 to 1707.3) | 3697.06 (2866.21 to 4819.42) | 1136.61 (768.74 to 1598.65) | 2560.45 (1940.06 to 3378.9) | 134.93% (77.34% to 219.06%) | 150.54% (69.18% to 264.65%) | 128.61% (53.43% to 232.96%) |
|  |  |  | Age-standardized rate (per 100,000) | 477.43 (360.58 to 692.93) | 280.85 (191.19 to 406.17) | 649.72 (467.54 to 980.17) | 395.75 (307.49 to 514.94) | 231.26 (156.13 to 326.16) | 577.09 (439.33 to 755.69) | -17.11% (-37.69% to 11.75%) | -17.66% (-44.66% to 19.19%) | -11.18% (-40.02% to 27.18%) |
|  |  | Deaths | All age number | 53.84 (40.44 to 78.86) | 14.04 (9.51 to 20.39) | 39.81 (28.39 to 60.54) | 137.72 (107.57 to 178.94) | 40.07 (27.23 to 56.98) | 97.65 (74.71 to 127.47) | 155.78% (90.83% to 243.49%) | 185.41% (92.22% to 310.31%) | 145.33% (65.51% to 251.86%) |
|  |  |  | Age-standardized rate (per 100,000) | 18.92 (14.31 to 27.64) | 10.33 (7.1 to 15.16) | 27.03 (19.36 to 40.89) | 16.22 (12.68 to 21.01) | 8.84 (6.0 to 12.57) | 24.79 (19.13 to 32.13) | -14.26% (-36.25% to 12.34%) | -14.46% (-40.52% to 20.52%) | -8.28% (-36.68% to 30.88%) |
|  |  | YLDs | All age number | 24.22 (16.25 to 35.36) | 9.3 (5.32 to 14.55) | 14.93 (9.31 to 22.48) | 106.46 (67.45 to 155.44) | 49.72 (25.84 to 80.92) | 56.74 (37.33 to 81.76) | 339.47% (230.35% to 471.95%) | 434.75% (264.36% to 668.29%) | 280.12% (166.73% to 453.94%) |
|  |  |  | Age-standardized rate (per 100,000) | 7.55 (5.13 to 10.96) | 5.82 (3.29 to 9.12) | 9.07 (5.74 to 13.49) | 11.28 (7.13 to 16.32) | 9.9 (5.12 to 16.09) | 13.08 (8.7 to 18.85) | 49.34% (13.74% to 94.48%) | 70.16% (18.87% to 140.77%) | 44.11% (1.62% to 105.03%) |
|  |  | YLLs | All age number | 1549.43 (1164.46 to 2255.07) | 444.36 (297.14 to 640.67) | 1105.07 (784.54 to 1687.21) | 3590.6 (2789.99 to 4664.17) | 1086.89 (744.61 to 1531.9) | 2503.71 (1897.26 to 3311.36) | 131.74% (74.73% to 215.34%) | 144.6% (63.77% to 256.31%) | 126.57% (52.02% to 230.1%) |
|  |  |  | Age-standardized rate (per 100,000) | 469.88 (354.59 to 682.62) | 275.03 (185.57 to 398.26) | 640.64 (460.18 to 967.03) | 384.47 (299.52 to 499.97) | 221.36 (150.39 to 312.92) | 564.02 (428.38 to 741.67) | -18.18% (-38.56% to 10.47%) | -19.52% (-46.05% to 16.23%) | -11.96% (-40.63% to 26.08%) |
|  | Behavioral risks | DALYs | All age number | 1323.59 (956.01 to 2056.47) | 344.09 (219.28 to 551.41) | 979.5 (676.25 to 1546.11) | 2776.17 (2083.78 to 3699.07) | 696.62 (440.64 to 1079.37) | 2079.55 (1525.19 to 2787.09) | 109.75% (54.87% to 185.62%) | 102.45% (37.55% to 197.85%) | 112.31% (43.82% to 216.69%) |
|  |  |  | Age-standardized rate (per 100,000) | 402.09 (292.17 to 619.96) | 211.66 (134.92 to 342.51) | 568.32 (396.83 to 883.77) | 296.81 (221.25 to 395.8) | 139.53 (88.77 to 216.6) | 469.52 (343.63 to 630.44) | -26.18% (-45.63% to 0.16%) | -34.08% (-53.81% to -2.94%) | -17.39% (-43.16% to 21.45%) |
|  |  | Deaths | All age number | 45.5 (32.73 to 70.85) | 10.52 (6.72 to 17.16) | 34.99 (24.32 to 54.88) | 103.24 (77.37 to 137.09) | 23.67 (15.26 to 36.58) | 79.57 (58.22 to 106.87) | 126.87% (64.86% to 210.02%) | 125.05% (59.64% to 229.73%) | 127.42% (54.69% to 233.77%) |
|  |  |  | Age-standardized rate (per 100,000) | 15.87 (11.49 to 24.52) | 7.65 (4.85 to 12.63) | 23.56 (16.45 to 37.05) | 12.11 (9.1 to 16.26) | 5.15 (3.33 to 7.94) | 20.15 (14.65 to 27.35) | -23.68% (-43.38% to 2.17%) | -32.67% (-51.79% to -2.72%) | -14.47% (-41.18% to 24.25%) |
|  |  | YLDs | All age number | 20.65 (13.71 to 31.03) | 7.4 (4.11 to 12.2) | 13.25 (8.23 to 20.43) | 76.97 (47.25 to 117.06) | 31.95 (13.85 to 55.39) | 45.02 (28.76 to 65.71) | 272.81% (177.84% to 387.88%) | 332.03% (159.06% to 535.3%) | 239.76% (139.69% to 401.46%) |
|  |  |  | Age-standardized rate (per 100,000) | 6.41 (4.26 to 9.65) | 4.53 (2.5 to 7.44) | 8.03 (5.06 to 12.31) | 8.1 (5.02 to 12.18) | 6.17 (2.73 to 10.57) | 10.44 (6.66 to 15.11) | 26.49% (-4.32% to 65.27%) | 36.19% (-14.73% to 97.07%) | 29.96% (-7.18% to 87.05%) |
|  |  | YLLs | All age number | 1302.94 (938.96 to 2025.01) | 336.69 (214.99 to 538.8) | 966.25 (667.26 to 1527.02) | 2699.19 (2021.96 to 3595.91) | 664.67 (424.84 to 1028.6) | 2034.53 (1493.62 to 2730.91) | 107.16% (52.63% to 181.91%) | 97.41% (34.27% to 191.31%) | 110.56% (42.36% to 214.06%) |
|  |  |  | Age-standardized rate (per 100,000) | 395.68 (287.13 to 610.4) | 207.13 (132.11 to 334.99) | 560.29 (390.23 to 870.6) | 288.7 (215.91 to 384.27) | 133.36 (85.7 to 205.94) | 459.08 (334.9 to 615.61) | -27.04% (-46.28% to -1.16%) | -35.61% (-54.86% to -5.7%) | -18.06% (-43.71% to 20.62%) |
|  | Environmental/occupational risks | DALYs | All age number | 299.13 (179.32 to 455.28) | 59.4 (32.69 to 102.14) | 239.73 (141.26 to 371.95) | 676.86 (416.65 to 992.77) | 175.65 (97.32 to 273.13) | 501.21 (308.66 to 755.27) | 126.28% (43.1% to 247.71%) | 195.71% (55.77% to 402.92%) | 109.08% (18.74% to 233.14%) |
|  |  |  | Age-standardized rate (per 100,000) | 91.78 (54.72 to 138.74) | 38.86 (21.06 to 66.72) | 138.89 (82.33 to 215.91) | 72.6 (44.64 to 106.37) | 36.26 (19.81 to 56.62) | 111.9 (68.65 to 168.63) | -20.9% (-49.52% to 21.88%) | -6.69% (-50.15% to 56.78%) | -19.43% (-53.97% to 27.7%) |
|  |  | Deaths | All age number | 10.5 (6.26 to 15.97) | 2.0 (1.08 to 3.43) | 8.5 (5.04 to 13.25) | 25.78 (15.87 to 37.78) | 6.72 (3.63 to 10.53) | 19.06 (11.64 to 28.71) | 145.43% (56.61% to 278.94%) | 236.1% (82.89% to 453.01%) | 124.12% (28.81% to 256.02%) |
|  |  |  | Age-standardized rate (per 100,000) | 3.76 (2.25 to 5.67) | 1.58 (0.85 to 2.66) | 5.85 (3.48 to 9.02) | 3.04 (1.86 to 4.45) | 1.5 (0.81 to 2.34) | 4.8 (2.9 to 7.19) | -19.22% (-47.52% to 24.26%) | -4.52% (-48.1% to 60.36%) | -18.03% (-52.98% to 28.42%) |
|  |  | YLDs | All age number | 2.53 (1.37 to 4.15) | 0.49 (0.25 to 0.85) | 2.04 (1.05 to 3.4) | 6.49 (3.57 to 10.05) | 1.62 (0.82 to 2.62) | 4.87 (2.72 to 7.68) | 156.7% (56.44% to 313.47%) | 231.63% (83.04% to 459.34%) | 138.72% (35.13% to 307.49%) |
|  |  |  | Age-standardized rate (per 100,000) | 0.83 (0.45 to 1.38) | 0.35 (0.18 to 0.6) | 1.27 (0.67 to 2.16) | 0.72 (0.39 to 1.12) | 0.35 (0.17 to 0.56) | 1.13 (0.63 to 1.78) | -13.25% (-47.31% to 37.11%) | -0.78% (-45.18% to 72.66%) | -11.12% (-50.47% to 49.16%) |
|  |  | YLLs | All age number | 296.6 (178.11 to 451.43) | 58.91 (32.38 to 101.44) | 237.69 (140.12 to 368.86) | 670.37 (412.83 to 983.89) | 174.03 (96.57 to 270.75) | 496.34 (305.98 to 748.95) | 126.02% (42.99% to 247.03%) | 195.42% (55.58% to 402.02%) | 108.82% (18.65% to 233.18%) |
|  |  |  | Age-standardized rate (per 100,000) | 90.95 (54.31 to 137.58) | 38.51 (20.85 to 66.13) | 137.61 (81.64 to 214.12) | 71.88 (44.22 to 105.41) | 35.91 (19.63 to 56.05) | 110.77 (68.12 to 167.3) | -20.97% (-49.57% to 21.75%) | -6.74% (-50.2% to 56.59%) | -19.51% (-54.05% to 27.76%) |
|  | Metabolic risks | DALYs | All age number | 153.94 (68.24 to 244.27) | 74.23 (33.16 to 122.87) | 79.71 (32.7 to 136.52) | 791.67 (286.78 to 1303.11) | 361.28 (137.76 to 591.16) | 430.39 (149.48 to 734.56) | 414.27% (256.3% to 596.29%) | 386.71% (201.64% to 651.27%) | 439.94% (252.1% to 699.27%) |
|  |  |  | Age-standardized rate (per 100,000) | 45.5 (18.98 to 72.9) | 46.23 (19.31 to 77.78) | 44.91 (16.9 to 78.35) | 85.63 (29.65 to 142.06) | 75.89 (27.86 to 123.96) | 96.89 (32.23 to 166.52) | 88.18% (34.71% to 149.32%) | 64.17% (5.12% to 146.88%) | 115.75% (43.07% to 215.14%) |
|  |  | Deaths | All age number | 5.04 (2.06 to 8.14) | 2.34 (0.97 to 3.96) | 2.7 (1.0 to 4.76) | 29.6 (9.73 to 49.87) | 13.38 (4.65 to 22.17) | 16.22 (5.2 to 27.87) | 487.38% (315.26% to 674.32%) | 471.31% (259.69% to 764.11%) | 501.33% (295.17% to 783.62%) |
|  |  |  | Age-standardized rate (per 100,000) | 1.76 (0.69 to 2.89) | 1.73 (0.69 to 2.95) | 1.81 (0.61 to 3.23) | 3.53 (1.13 to 6.02) | 3.01 (1.03 to 5.05) | 4.15 (1.29 to 7.17) | 99.95% (48.32% to 157.09%) | 74.42% (14.55% to 155.65%) | 128.71% (53.62% to 220.29%) |
|  |  | YLDs | All age number | 3.21 (1.27 to 5.5) | 1.83 (0.62 to 3.27) | 1.38 (0.53 to 2.5) | 32.87 (13.08 to 55.81) | 19.48 (6.82 to 35.23) | 13.39 (5.9 to 23.1) | 923.06% (648.14% to 1314.49%) | 963.2% (588.55% to 1548.49%) | 869.77% (565.95% to 1364.81%) |
|  |  |  | Age-standardized rate (per 100,000) | 1.01 (0.38 to 1.74) | 1.23 (0.39 to 2.2) | 0.83 (0.31 to 1.53) | 3.56 (1.37 to 6.05) | 4.1 (1.36 to 7.49) | 3.04 (1.28 to 5.26) | 251.86% (165.24% to 375.34%) | 233.4% (118.84% to 396.31%) | 267.0% (155.89% to 443.96%) |
|  |  | YLLs | All age number | 150.73 (67.11 to 239.07) | 72.4 (32.59 to 119.64) | 78.33 (32.16 to 134.13) | 758.8 (271.27 to 1249.97) | 341.79 (129.62 to 556.0) | 417.01 (141.76 to 712.68) | 403.43% (247.41% to 578.44%) | 372.12% (193.48% to 631.99%) | 432.36% (245.51% to 690.31%) |
|  |  |  | Age-standardized rate (per 100,000) | 44.49 (18.6 to 71.27) | 45.0 (18.95 to 75.74) | 44.08 (16.6 to 77.03) | 82.07 (28.18 to 136.41) | 71.79 (26.11 to 116.9) | 93.85 (30.85 to 161.6) | 84.46% (32.31% to 145.06%) | 59.54% (2.22% to 139.58%) | 112.91% (40.13% to 210.19%) |
| East Azarbayejan | All risk factors | DALYs | All age number | 12109.46 (8859.61 to 16908.57) | 4000.81 (2666.1 to 5904.34) | 8108.64 (5581.7 to 11588.51) | 27264.72 (20792.7 to 34739.39) | 9849.26 (6204.71 to 13861.08) | 17415.46 (13030.21 to 22352.98) | 125.15% (70.52% to 191.49%) | 146.18% (69.59% to 264.04%) | 114.78% (53.28% to 206.18%) |
|  |  |  | Age-standardized rate (per 100,000) | 669.68 (492.96 to 930.8) | 451.56 (294.93 to 663.34) | 858.38 (599.11 to 1219.16) | 644.52 (489.55 to 824.29) | 462.0 (291.8 to 650.84) | 830.21 (617.43 to 1062.84) | -3.76% (-26.59% to 23.47%) | 2.31% (-28.91% to 47.85%) | -3.28% (-31.05% to 36.31%) |
|  |  | Deaths | All age number | 411.46 (302.06 to 569.7) | 125.23 (83.13 to 184.03) | 286.23 (197.3 to 404.84) | 1052.7 (798.89 to 1349.84) | 367.84 (231.62 to 520.34) | 684.86 (509.02 to 884.16) | 155.84% (93.72% to 229.76%) | 193.73% (105.78% to 326.65%) | 139.27% (70.43% to 235.98%) |
|  |  |  | Age-standardized rate (per 100,000) | 27.62 (20.46 to 37.9) | 17.84 (11.59 to 25.96) | 36.33 (25.35 to 51.49) | 28.05 (21.25 to 35.92) | 19.6 (12.19 to 27.59) | 36.67 (27.41 to 47.31) | 1.55% (-22.4% to 29.82%) | 9.89% (-22.29% to 55.09%) | 0.93% (-27.02% to 40.54%) |
|  |  | YLDs | All age number | 180.52 (117.02 to 270.17) | 78.52 (39.89 to 128.13) | 102.0 (62.29 to 152.42) | 718.58 (422.69 to 1075.21) | 392.59 (184.34 to 646.82) | 325.99 (217.07 to 456.31) | 298.06% (199.68% to 411.12%) | 399.97% (239.97% to 620.27%) | 219.61% (126.61% to 364.36%) |
|  |  |  | Age-standardized rate (per 100,000) | 10.3 (6.78 to 15.17) | 8.91 (4.57 to 14.23) | 11.42 (7.11 to 16.79) | 16.72 (10.04 to 24.65) | 17.67 (8.43 to 28.54) | 15.88 (10.56 to 22.22) | 62.3% (22.92% to 108.24%) | 98.28% (37.76% to 183.42%) | 39.02% (-0.23% to 95.94%) |
|  |  | YLLs | All age number | 11928.94 (8726.33 to 16704.28) | 3922.29 (2616.29 to 5794.66) | 8006.65 (5510.73 to 11445.83) | 26546.14 (20305.98 to 33889.6) | 9456.67 (5978.37 to 13323.03) | 17089.47 (12772.13 to 21942.84) | 122.54% (68.26% to 188.12%) | 141.1% (65.68% to 255.79%) | 113.44% (52.35% to 204.54%) |
|  |  |  | Age-standardized rate (per 100,000) | 659.37 (485.56 to 919.26) | 442.65 (289.97 to 652.06) | 846.95 (591.71 to 1203.37) | 627.8 (478.69 to 802.78) | 444.32 (281.3 to 626.52) | 814.33 (605.84 to 1045.19) | -4.79% (-27.38% to 22.34%) | 0.38% (-30.09% to 45.23%) | -3.85% (-31.45% to 35.56%) |
|  | Behavioral risks | DALYs | All age number | 10573.36 (7601.0 to 15509.41) | 3226.14 (1959.38 to 4925.23) | 7347.22 (4953.31 to 10763.45) | 20749.06 (15463.19 to 27117.1) | 6267.76 (3582.73 to 9311.11) | 14481.3 (10686.68 to 18684.36) | 96.24% (52.11% to 155.56%) | 94.28% (34.9% to 177.96%) | 97.1% (41.74% to 181.87%) |
|  |  |  | Age-standardized rate (per 100,000) | 585.93 (422.3 to 864.03) | 362.67 (218.87 to 551.71) | 779.4 (525.7 to 1150.66) | 487.97 (364.07 to 639.93) | 289.12 (165.56 to 423.72) | 690.25 (507.58 to 886.09) | -16.72% (-35.15% to 7.21%) | -20.28% (-42.71% to 11.46%) | -11.44% (-35.28% to 25.84%) |
|  |  | Deaths | All age number | 361.02 (260.54 to 529.77) | 100.21 (61.46 to 154.01) | 260.81 (176.39 to 381.77) | 798.74 (594.12 to 1053.91) | 226.42 (130.43 to 327.63) | 572.32 (422.79 to 735.9) | 121.24% (71.89% to 187.29%) | 125.94% (63.55% to 212.56%) | 119.44% (59.46% to 208.84%) |
|  |  |  | Age-standardized rate (per 100,000) | 24.17 (17.67 to 35.24) | 14.2 (8.59 to 21.78) | 33.05 (22.57 to 48.87) | 21.18 (15.74 to 27.93) | 11.95 (6.95 to 17.6) | 30.59 (22.78 to 39.42) | -12.38% (-31.01% to 12.26%) | -15.87% (-38.28% to 14.7%) | -7.43% (-31.87% to 28.45%) |
|  |  | YLDs | All age number | 157.04 (99.76 to 239.19) | 64.02 (31.04 to 109.06) | 93.02 (56.81 to 141.03) | 525.86 (304.87 to 804.04) | 259.06 (98.4 to 458.38) | 266.8 (175.47 to 375.06) | 234.85% (150.02% to 334.91%) | 304.64% (151.83% to 476.79%) | 186.82% (104.03% to 312.53%) |
|  |  |  | Age-standardized rate (per 100,000) | 8.95 (5.76 to 13.53) | 7.16 (3.5 to 12.08) | 10.41 (6.5 to 15.56) | 12.13 (7.25 to 18.21) | 11.38 (4.52 to 19.79) | 13.01 (8.66 to 18.33) | 35.61% (2.09% to 74.79%) | 58.78% (2.8% to 119.35%) | 24.97% (-8.89% to 77.01%) |
|  |  | YLLs | All age number | 10416.32 (7483.04 to 15303.67) | 3162.12 (1927.96 to 4824.92) | 7254.2 (4892.89 to 10635.01) | 20223.2 (15065.95 to 26482.41) | 6008.69 (3460.47 to 8865.28) | 14214.51 (10489.29 to 18373.04) | 94.15% (50.75% to 152.99%) | 90.02% (32.79% to 171.93%) | 95.95% (40.86% to 180.29%) |
|  |  |  | Age-standardized rate (per 100,000) | 576.98 (415.79 to 852.24) | 355.5 (215.43 to 543.14) | 768.98 (517.51 to 1134.66) | 475.83 (355.57 to 625.13) | 277.74 (160.05 to 401.76) | 677.24 (498.84 to 870.19) | -17.53% (-35.86% to 6.25%) | -21.87% (-43.8% to 8.7%) | -11.93% (-35.65% to 25.3%) |
|  | Environmental/occupational risks | DALYs | All age number | 1435.97 (869.36 to 2293.84) | 305.76 (150.67 to 570.83) | 1130.21 (679.87 to 1827.18) | 3461.03 (2127.22 to 5250.95) | 977.42 (512.75 to 1605.83) | 2483.6 (1549.57 to 3702.49) | 141.02% (65.62% to 266.89%) | 219.67% (73.31% to 510.49%) | 119.75% (42.44% to 249.88%) |
|  |  |  | Age-standardized rate (per 100,000) | 77.54 (46.5 to 124.59) | 35.88 (17.81 to 65.36) | 114.37 (68.3 to 183.95) | 80.79 (49.17 to 123.95) | 46.7 (24.27 to 77.58) | 115.1 (70.89 to 173.79) | 4.19% (-28.55% to 57.08%) | 30.17% (-29.07% to 147.83%) | 0.65% (-34.22% to 59.25%) |
|  |  | Deaths | All age number | 48.56 (29.26 to 77.8) | 10.11 (5.0 to 18.52) | 38.45 (22.98 to 61.69) | 131.86 (79.37 to 202.84) | 38.87 (20.52 to 64.23) | 92.98 (56.66 to 142.52) | 171.54% (84.23% to 311.09%) | 284.52% (109.91% to 638.04%) | 141.83% (58.25% to 283.02%) |
|  |  |  | Age-standardized rate (per 100,000) | 3.15 (1.84 to 5.11) | 1.52 (0.76 to 2.83) | 4.61 (2.71 to 7.42) | 3.44 (2.02 to 5.31) | 2.1 (1.12 to 3.46) | 4.8 (2.87 to 7.32) | 9.2% (-25.88% to 62.89%) | 38.35% (-20.72% to 166.57%) | 4.22% (-31.33% to 60.39%) |
|  |  | YLDs | All age number | 11.81 (6.63 to 19.8) | 2.45 (1.18 to 4.39) | 9.36 (5.15 to 16.15) | 31.07 (16.85 to 47.09) | 8.63 (4.16 to 14.49) | 22.44 (12.12 to 34.56) | 163.07% (74.05% to 314.44%) | 252.13% (88.51% to 584.83%) | 139.76% (46.55% to 298.43%) |
|  |  |  | Age-standardized rate (per 100,000) | 0.68 (0.39 to 1.16) | 0.32 (0.15 to 0.57) | 1.01 (0.57 to 1.71) | 0.75 (0.4 to 1.15) | 0.43 (0.21 to 0.72) | 1.08 (0.58 to 1.66) | 10.28% (-27.35% to 72.56%) | 35.45% (-28.12% to 174.21%) | 7.25% (-33.75% to 74.86%) |
|  |  | YLLs | All age number | 1424.16 (860.83 to 2276.86) | 303.31 (149.76 to 566.93) | 1120.85 (672.77 to 1811.3) | 3429.96 (2110.61 to 5208.73) | 968.8 (508.14 to 1592.24) | 2461.16 (1535.65 to 3669.3) | 140.84% (65.42% to 266.92%) | 219.41% (73.13% to 510.07%) | 119.58% (42.4% to 249.93%) |
|  |  |  | Age-standardized rate (per 100,000) | 76.86 (46.02 to 123.5) | 35.56 (17.66 to 64.88) | 113.36 (67.55 to 182.35) | 80.04 (48.71 to 122.91) | 46.27 (24.01 to 76.8) | 114.02 (70.32 to 172.02) | 4.14% (-28.57% to 57.19%) | 30.12% (-29.12% to 147.97%) | 0.59% (-34.26% to 59.29%) |
|  | Metabolic risks | DALYs | All age number | 1215.19 (502.62 to 2016.59) | 660.55 (288.94 to 1070.98) | 554.63 (208.32 to 974.82) | 6395.9 (2104.17 to 10792.8) | 3452.54 (1089.98 to 5966.82) | 2943.36 (993.82 to 5036.55) | 426.33% (255.16% to 590.46%) | 422.67% (215.53% to 635.03%) | 430.68% (256.19% to 662.91%) |
|  |  |  | Age-standardized rate (per 100,000) | 66.54 (26.04 to 110.57) | 75.85 (31.18 to 124.08) | 58.0 (20.28 to 102.74) | 154.32 (49.63 to 261.31) | 167.1 (51.97 to 286.4) | 141.4 (46.16 to 243.6) | 131.92% (64.02% to 196.47%) | 120.3% (40.23% to 206.42%) | 143.8% (67.05% to 245.53%) |
|  |  | Deaths | All age number | 40.41 (15.52 to 67.83) | 21.5 (8.61 to 35.4) | 18.91 (6.43 to 33.48) | 251.94 (77.49 to 431.45) | 136.95 (41.38 to 235.62) | 114.99 (35.54 to 200.59) | 523.4% (332.52% to 698.61%) | 536.94% (303.28% to 779.02%) | 508.01% (316.1% to 758.36%) |
|  |  |  | Age-standardized rate (per 100,000) | 2.74 (0.98 to 4.69) | 3.09 (1.17 to 5.08) | 2.41 (0.77 to 4.34) | 6.83 (2.05 to 11.76) | 7.42 (2.24 to 12.82) | 6.23 (1.85 to 10.97) | 149.37% (84.54% to 215.41%) | 140.43% (60.73% to 224.1%) | 157.91% (81.11% to 258.78%) |
|  |  | YLDs | All age number | 24.43 (8.08 to 44.54) | 15.55 (4.77 to 28.56) | 8.88 (3.08 to 16.31) | 227.2 (71.41 to 403.09) | 153.91 (41.37 to 284.86) | 73.28 (27.85 to 131.2) | 830.0% (552.78% to 1116.33%) | 890.09% (557.6% to 1293.88%) | 724.86% (463.59% to 1105.47%) |
|  |  |  | Age-standardized rate (per 100,000) | 1.41 (0.44 to 2.55) | 1.87 (0.53 to 3.44) | 1.0 (0.34 to 1.82) | 5.42 (1.69 to 9.61) | 7.26 (1.93 to 13.47) | 3.57 (1.33 to 6.4) | 285.62% (176.15% to 398.21%) | 288.83% (167.65% to 432.57%) | 256.63% (146.73% to 413.17%) |
|  |  | YLLs | All age number | 1190.76 (494.27 to 1971.12) | 645.01 (282.94 to 1045.23) | 545.75 (205.1 to 958.17) | 6168.7 (2038.08 to 10411.78) | 3298.62 (1051.78 to 5620.1) | 2870.08 (969.55 to 4923.46) | 418.05% (250.28% to 581.49%) | 411.41% (207.52% to 619.85%) | 425.9% (252.11% to 656.42%) |
|  |  |  | Age-standardized rate (per 100,000) | 65.13 (25.64 to 108.14) | 73.99 (30.5 to 120.64) | 56.99 (19.94 to 100.87) | 148.89 (48.08 to 252.4) | 159.84 (50.2 to 272.3) | 137.82 (45.03 to 238.18) | 128.6% (61.82% to 192.12%) | 116.04% (37.29% to 200.64%) | 141.82% (65.69% to 242.6%) |
| Fars | All risk factors | DALYs | All age number | 8396.17 (6102.77 to 11467.59) | 2503.72 (1587.67 to 3660.87) | 5892.45 (4087.71 to 8244.03) | 24102.44 (17748.61 to 30966.43) | 8517.25 (5043.97 to 12115.65) | 15585.19 (11697.82 to 20176.14) | 187.06% (116.02% to 282.32%) | 240.18% (130.79% to 392.66%) | 164.49% (83.39% to 293.16%) |
|  |  |  | Age-standardized rate (per 100,000) | 473.28 (347.06 to 637.95) | 277.56 (177.75 to 403.42) | 660.1 (462.52 to 914.53) | 471.33 (350.91 to 602.67) | 324.58 (195.11 to 457.34) | 623.31 (470.89 to 803.47) | -0.41% (-24.56% to 31.31%) | 16.94% (-19.82% to 66.59%) | -5.57% (-34.18% to 37.44%) |
|  |  | Deaths | All age number | 281.38 (204.71 to 382.52) | 75.35 (48.32 to 110.22) | 206.03 (142.53 to 285.99) | 880.51 (660.58 to 1124.17) | 292.02 (179.48 to 412.24) | 588.5 (444.07 to 754.66) | 212.92% (136.41% to 315.43%) | 287.54% (165.84% to 450.03%) | 185.63% (97.66% to 319.76%) |
|  |  |  | Age-standardized rate (per 100,000) | 18.78 (13.77 to 25.16) | 10.11 (6.59 to 14.59) | 27.65 (19.49 to 37.88) | 19.28 (14.41 to 24.57) | 12.48 (7.79 to 17.48) | 26.38 (19.92 to 33.86) | 2.69% (-21.74% to 35.83%) | 23.51% (-14.48% to 71.62%) | -4.62% (-31.74% to 35.85%) |
|  |  | YLDs | All age number | 144.42 (92.45 to 213.46) | 61.44 (31.11 to 101.46) | 82.98 (50.59 to 124.02) | 744.68 (438.95 to 1124.51) | 416.56 (172.53 to 705.28) | 328.12 (221.68 to 490.2) | 415.65% (273.69% to 570.41%) | 578.04% (340.85% to 873.9%) | 295.42% (172.75% to 480.8%) |
|  |  |  | Age-standardized rate (per 100,000) | 8.32 (5.4 to 12.17) | 6.86 (3.5 to 11.17) | 9.78 (6.1 to 14.53) | 14.36 (8.71 to 21.45) | 15.43 (6.54 to 25.58) | 13.44 (9.06 to 19.96) | 72.6% (26.56% to 122.37%) | 124.85% (50.45% to 222.12%) | 37.48% (-3.85% to 99.41%) |
|  |  | YLLs | All age number | 8251.75 (6009.19 to 11264.59) | 2442.28 (1552.54 to 3573.71) | 5809.47 (4027.94 to 8112.63) | 23357.76 (17257.35 to 29930.44) | 8100.68 (4868.77 to 11582.93) | 15257.07 (11446.3 to 19764.03) | 183.06% (113.35% to 277.38%) | 231.68% (126.07% to 380.67%) | 162.62% (82.13% to 290.66%) |
|  |  |  | Age-standardized rate (per 100,000) | 464.96 (340.58 to 628.32) | 270.7 (174.04 to 393.92) | 650.32 (455.69 to 901.37) | 456.96 (341.21 to 585.17) | 309.15 (188.32 to 439.31) | 609.87 (460.2 to 787.21) | -1.72% (-25.54% to 29.76%) | 14.2% (-21.62% to 62.57%) | -6.22% (-34.71% to 36.67%) |
|  | Behavioral risks | DALYs | All age number | 7004.6 (5041.58 to 9940.4) | 1815.13 (1106.55 to 2909.9) | 5189.46 (3466.9 to 7359.09) | 17493.21 (12678.31 to 22896.48) | 4826.16 (2591.85 to 7401.99) | 12667.06 (9261.87 to 16612.03) | 149.74% (85.36% to 234.64%) | 165.88% (75.16% to 289.85%) | 144.09% (64.85% to 262.21%) |
|  |  |  | Age-standardized rate (per 100,000) | 397.56 (285.06 to 562.41) | 199.12 (121.19 to 319.09) | 586.64 (393.95 to 825.46) | 342.02 (251.5 to 446.32) | 178.64 (97.88 to 270.75) | 511.08 (374.41 to 670.02) | -13.97% (-35.91% to 14.99%) | -10.29% (-39.27% to 29.23%) | -12.88% (-39.77% to 25.07%) |
|  |  | Deaths | All age number | 237.12 (167.86 to 337.83) | 53.49 (32.57 to 86.33) | 183.64 (122.89 to 260.27) | 644.12 (483.01 to 845.82) | 156.45 (89.17 to 238.06) | 487.66 (358.44 to 635.6) | 171.64% (99.94% to 267.26%) | 192.51% (105.0% to 319.49%) | 165.56% (82.04% to 287.89%) |
|  |  |  | Age-standardized rate (per 100,000) | 15.82 (11.33 to 22.3) | 7.09 (4.35 to 11.12) | 24.75 (17.03 to 34.6) | 14.12 (10.73 to 18.51) | 6.56 (3.8 to 9.84) | 22.0 (16.39 to 28.83) | -10.75% (-33.38% to 19.0%) | -7.49% (-34.17% to 29.32%) | -11.11% (-37.59% to 26.72%) |
|  |  | YLDs | All age number | 121.67 (75.59 to 183.82) | 47.2 (21.38 to 83.95) | 74.47 (44.37 to 111.78) | 531.93 (295.86 to 845.64) | 263.01 (79.36 to 482.86) | 268.92 (175.44 to 405.79) | 337.19% (210.03% to 484.35%) | 457.22% (221.21% to 724.77%) | 261.11% (150.07% to 436.41%) |
|  |  |  | Age-standardized rate (per 100,000) | 6.99 (4.43 to 10.34) | 5.11 (2.33 to 9.16) | 8.81 (5.43 to 13.1) | 10.13 (5.82 to 15.84) | 9.31 (2.89 to 16.84) | 11.11 (7.26 to 16.56) | 44.93% (5.8% to 89.4%) | 82.13% (8.03% to 161.15%) | 26.09% (-11.56% to 83.78%) |
|  |  | YLLs | All age number | 6882.93 (4944.15 to 9798.34) | 1767.93 (1072.03 to 2821.63) | 5114.99 (3404.73 to 7261.97) | 16961.29 (12356.1 to 22231.25) | 4563.15 (2518.15 to 7008.76) | 12398.14 (9080.78 to 16300.32) | 146.43% (83.26% to 230.46%) | 158.11% (72.34% to 279.66%) | 142.39% (63.63% to 259.34%) |
|  |  |  | Age-standardized rate (per 100,000) | 390.57 (279.61 to 554.23) | 194.01 (118.0 to 312.0) | 577.83 (388.26 to 814.58) | 331.89 (245.53 to 433.81) | 169.33 (94.95 to 255.84) | 499.97 (366.87 to 655.93) | -15.03% (-36.89% to 14.02%) | -12.72% (-39.96% to 25.68%) | -13.47% (-40.26% to 24.3%) |
|  | Environmental/occupational risks | DALYs | All age number | 1388.22 (843.47 to 2115.7) | 265.5 (138.11 to 454.7) | 1122.72 (669.59 to 1739.5) | 3608.95 (2248.17 to 5497.78) | 953.45 (523.25 to 1538.68) | 2655.5 (1682.95 to 3981.61) | 159.97% (75.27% to 292.48%) | 259.12% (105.94% to 498.18%) | 136.52% (50.95% to 278.96%) |
|  |  |  | Age-standardized rate (per 100,000) | 77.98 (47.27 to 119.58) | 31.26 (16.44 to 53.18) | 122.05 (73.07 to 191.74) | 70.27 (43.37 to 107.5) | 37.35 (20.44 to 60.43) | 103.75 (65.41 to 156.6) | -9.89% (-39.59% to 35.73%) | 19.51% (-30.57% to 98.2%) | -15.0% (-46.09% to 35.41%) |
|  |  | Deaths | All age number | 47.25 (28.56 to 71.71) | 8.78 (4.63 to 15.04) | 38.47 (22.8 to 60.47) | 132.89 (81.52 to 205.24) | 36.28 (19.77 to 58.28) | 96.61 (60.77 to 147.3) | 181.27% (88.91% to 327.86%) | 313.32% (141.95% to 599.23%) | 151.14% (60.68% to 300.55%) |
|  |  |  | Age-standardized rate (per 100,000) | 3.14 (1.89 to 4.78) | 1.28 (0.68 to 2.17) | 4.99 (2.99 to 7.85) | 2.88 (1.74 to 4.47) | 1.6 (0.87 to 2.59) | 4.21 (2.62 to 6.55) | -8.06% (-37.75% to 37.36%) | 25.33% (-25.3% to 111.36%) | -15.75% (-45.58% to 32.09%) |
|  |  | YLDs | All age number | 11.64 (6.42 to 20.2) | 2.18 (1.11 to 3.76) | 9.46 (4.94 to 16.2) | 32.82 (18.08 to 52.14) | 8.49 (4.3 to 14.34) | 24.33 (13.68 to 39.62) | 181.95% (82.31% to 357.3%) | 288.62% (121.32% to 560.62%) | 157.31% (51.63% to 337.29%) |
|  |  |  | Age-standardized rate (per 100,000) | 0.7 (0.38 to 1.23) | 0.28 (0.14 to 0.5) | 1.1 (0.59 to 1.93) | 0.66 (0.36 to 1.07) | 0.35 (0.17 to 0.6) | 0.99 (0.55 to 1.62) | -4.94% (-38.55% to 52.56%) | 23.31% (-30.88% to 121.35%) | -10.19% (-46.33% to 51.89%) |
|  |  | YLLs | All age number | 1376.58 (836.83 to 2096.17) | 263.31 (136.82 to 450.49) | 1113.27 (663.86 to 1727.39) | 3576.13 (2226.61 to 5446.13) | 944.96 (518.71 to 1525.87) | 2631.16 (1664.8 to 3944.24) | 159.78% (75.11% to 292.25%) | 258.88% (105.89% to 497.77%) | 136.35% (50.84% to 278.57%) |
|  |  |  | Age-standardized rate (per 100,000) | 77.28 (46.86 to 118.32) | 30.97 (16.27 to 52.71) | 120.96 (72.49 to 189.49) | 69.61 (42.96 to 106.32) | 37.01 (20.25 to 59.96) | 102.76 (64.75 to 155.02) | -9.93% (-39.63% to 35.67%) | 19.47% (-30.63% to 98.21%) | -15.04% (-46.16% to 35.13%) |
|  | Metabolic risks | DALYs | All age number | 1008.43 (408.65 to 1611.1) | 534.41 (225.45 to 899.91) | 474.01 (182.21 to 797.96) | 6186.37 (2060.83 to 10339.38) | 3365.21 (991.92 to 5740.24) | 2821.16 (1009.19 to 4715.36) | 513.47% (314.86% to 696.73%) | 529.7% (271.38% to 799.28%) | 495.17% (300.11% to 791.63%) |
|  |  |  | Age-standardized rate (per 100,000) | 54.83 (20.81 to 89.61) | 60.6 (24.12 to 104.02) | 49.65 (17.82 to 84.76) | 121.95 (39.49 to 204.33) | 133.75 (38.31 to 227.86) | 109.97 (38.33 to 184.8) | 122.41% (56.91% to 186.79%) | 120.72% (36.48% to 212.24%) | 121.51% (48.96% to 226.16%) |
|  |  | Deaths | All age number | 32.18 (12.15 to 52.9) | 16.89 (6.71 to 28.85) | 15.29 (5.37 to 25.95) | 223.2 (69.54 to 375.36) | 123.19 (34.45 to 212.46) | 100.01 (34.31 to 171.51) | 593.67% (382.89% to 792.68%) | 629.36% (354.69% to 924.6%) | 554.24% (346.66% to 884.54%) |
|  |  |  | Age-standardized rate (per 100,000) | 2.1 (0.74 to 3.53) | 2.28 (0.85 to 3.93) | 1.94 (0.64 to 3.34) | 4.88 (1.48 to 8.31) | 5.38 (1.49 to 9.33) | 4.38 (1.45 to 7.59) | 132.27% (70.2% to 192.81%) | 136.03% (54.24% to 225.48%) | 126.35% (55.96% to 233.89%) |
|  |  | YLDs | All age number | 22.65 (6.78 to 38.76) | 14.66 (3.9 to 26.97) | 7.99 (2.95 to 14.45) | 243.33 (63.65 to 433.41) | 172.58 (32.89 to 313.03) | 70.74 (26.37 to 128.24) | 974.23% (641.25% to 1327.69%) | 1076.86% (596.06% to 1600.75%) | 785.79% (507.78% to 1235.81%) |
|  |  |  | Age-standardized rate (per 100,000) | 1.33 (0.38 to 2.32) | 1.8 (0.42 to 3.29) | 0.9 (0.33 to 1.63) | 4.87 (1.22 to 8.67) | 6.89 (1.32 to 12.45) | 2.82 (1.03 to 5.08) | 266.64% (157.22% to 376.47%) | 282.92% (137.53% to 442.54%) | 211.64% (115.14% to 365.52%) |
|  |  | YLLs | All age number | 985.78 (402.62 to 1575.65) | 519.75 (221.21 to 876.93) | 466.03 (179.77 to 783.56) | 5943.05 (1983.65 to 9946.95) | 3192.63 (950.45 to 5438.43) | 2750.42 (986.04 to 4606.77) | 502.88% (309.49% to 684.11%) | 514.26% (264.28% to 776.55%) | 490.18% (295.9% to 785.45%) |
|  |  |  | Age-standardized rate (per 100,000) | 53.5 (20.48 to 87.68) | 58.8 (23.66 to 100.91) | 48.74 (17.49 to 83.12) | 117.08 (38.09 to 196.42) | 126.86 (36.68 to 216.12) | 107.15 (37.16 to 180.58) | 118.83% (55.83% to 181.14%) | 115.76% (34.6% to 205.92%) | 119.84% (47.86% to 223.95%) |
| Gilan | All risk factors | DALYs | All age number | 8117.1 (6162.93 to 11217.2) | 2576.49 (1599.88 to 3849.3) | 5540.61 (3940.32 to 7853.13) | 18763.12 (14127.32 to 23972.89) | 6758.38 (4318.9 to 9305.22) | 12004.75 (8970.3 to 15690.47) | 131.16% (76.15% to 199.69%) | 162.31% (85.2% to 269.74%) | 116.67% (52.85% to 210.13%) |
|  |  |  | Age-standardized rate (per 100,000) | 580.63 (442.9 to 797.98) | 365.22 (228.38 to 540.44) | 810.41 (579.87 to 1139.42) | 562.57 (425.15 to 720.09) | 398.87 (256.82 to 548.66) | 732.16 (549.47 to 951.95) | -3.11% (-26.19% to 24.75%) | 9.22% (-22.34% to 51.29%) | -9.66% (-36.72% to 28.45%) |
|  |  | Deaths | All age number | 275.1 (209.43 to 378.79) | 82.71 (52.19 to 123.99) | 192.39 (137.73 to 270.35) | 717.32 (548.66 to 916.75) | 247.04 (160.23 to 339.22) | 470.28 (354.0 to 608.0) | 160.74% (99.64% to 238.01%) | 198.69% (114.13% to 309.03%) | 144.43% (71.29% to 247.33%) |
|  |  |  | Age-standardized rate (per 100,000) | 22.63 (17.25 to 31.0) | 13.54 (8.7 to 20.24) | 33.29 (23.94 to 46.22) | 23.18 (17.79 to 29.56) | 15.78 (10.25 to 21.72) | 30.84 (23.41 to 39.72) | 2.42% (-21.13% to 30.73%) | 16.56% (-15.41% to 58.34%) | -7.34% (-34.6% to 30.02%) |
|  |  | YLDs | All age number | 141.74 (89.79 to 207.88) | 63.28 (33.45 to 103.74) | 78.46 (49.96 to 117.21) | 594.3 (349.54 to 877.1) | 335.56 (156.29 to 542.67) | 258.74 (170.65 to 366.45) | 319.29% (216.88% to 443.87%) | 430.29% (268.32% to 668.22%) | 229.77% (131.4% to 375.97%) |
|  |  |  | Age-standardized rate (per 100,000) | 10.34 (6.65 to 14.91) | 8.97 (4.73 to 14.67) | 11.97 (7.67 to 17.49) | 17.65 (10.45 to 25.9) | 19.34 (9.11 to 31.07) | 15.99 (10.6 to 22.64) | 70.66% (29.6% to 119.13%) | 115.62% (51.08% to 206.91%) | 33.58% (-4.98% to 89.17%) |
|  |  | YLLs | All age number | 7975.36 (6053.71 to 11046.63) | 2513.21 (1565.51 to 3768.69) | 5462.15 (3885.51 to 7750.52) | 18168.82 (13704.89 to 23237.18) | 6422.81 (4172.08 to 8844.72) | 11746.01 (8793.85 to 15361.71) | 127.81% (74.02% to 196.0%) | 155.56% (80.96% to 260.27%) | 115.04% (51.72% to 208.01%) |
|  |  |  | Age-standardized rate (per 100,000) | 570.29 (435.92 to 783.46) | 356.25 (223.04 to 528.47) | 798.44 (571.62 to 1122.22) | 544.93 (412.33 to 697.66) | 379.53 (247.81 to 521.25) | 716.17 (537.23 to 932.33) | -4.45% (-27.07% to 23.32%) | 6.54% (-24.23% to 47.37%) | -10.3% (-37.1% to 27.51%) |
|  | Behavioral risks | DALYs | All age number | 7065.06 (5193.58 to 10100.7) | 2050.42 (1211.16 to 3287.25) | 5014.65 (3538.49 to 7184.86) | 14261.03 (10897.15 to 18883.26) | 4223.31 (2463.61 to 6071.38) | 10037.72 (7363.76 to 13141.0) | 101.85% (55.2% to 165.68%) | 105.97% (40.59% to 189.0%) | 100.17% (41.79% to 185.65%) |
|  |  |  | Age-standardized rate (per 100,000) | 505.18 (375.09 to 724.08) | 289.99 (170.4 to 458.32) | 734.13 (521.37 to 1047.32) | 425.73 (326.91 to 564.33) | 247.52 (145.24 to 355.08) | 610.28 (449.26 to 800.1) | -15.73% (-34.72% to 9.3%) | -14.65% (-40.65% to 19.33%) | -16.87% (-40.87% to 17.44%) |
|  |  | Deaths | All age number | 240.04 (177.73 to 343.12) | 65.05 (39.08 to 102.59) | 174.99 (123.09 to 250.76) | 543.78 (420.69 to 717.92) | 149.21 (90.29 to 216.48) | 394.57 (293.19 to 515.93) | 126.53% (72.78% to 195.6%) | 129.37% (62.03% to 217.5%) | 125.48% (59.49% to 220.17%) |
|  |  |  | Age-standardized rate (per 100,000) | 19.64 (14.69 to 28.09) | 10.61 (6.35 to 16.49) | 30.19 (21.55 to 42.36) | 17.5 (13.49 to 23.13) | 9.47 (5.76 to 13.79) | 25.81 (19.27 to 33.8) | -10.87% (-30.81% to 14.86%) | -10.75% (-35.3% to 22.1%) | -14.5% (-39.25% to 19.5%) |
|  |  | YLDs | All age number | 122.75 (76.16 to 185.12) | 50.82 (24.42 to 88.1) | 71.93 (45.22 to 108.11) | 427.62 (247.07 to 660.68) | 211.86 (77.02 to 378.97) | 215.76 (141.07 to 317.17) | 248.37% (156.97% to 351.78%) | 316.86% (154.61% to 489.72%) | 199.98% (110.84% to 327.06%) |
|  |  |  | Age-standardized rate (per 100,000) | 8.92 (5.58 to 13.2) | 7.13 (3.42 to 12.16) | 10.95 (7.01 to 16.25) | 12.7 (7.4 to 19.51) | 12.2 (4.49 to 21.74) | 13.3 (8.72 to 19.35) | 42.45% (6.44% to 83.24%) | 71.05% (6.38% to 139.33%) | 21.44% (-14.12% to 69.48%) |
|  |  | YLLs | All age number | 6942.32 (5118.64 to 9939.74) | 1999.59 (1181.26 to 3215.4) | 4942.72 (3492.32 to 7090.12) | 13833.41 (10606.09 to 18344.86) | 4011.45 (2352.04 to 5754.18) | 9821.96 (7199.31 to 12801.25) | 99.26% (52.84% to 162.9%) | 100.61% (37.13% to 180.98%) | 98.72% (40.76% to 183.84%) |
|  |  |  | Age-standardized rate (per 100,000) | 496.27 (368.94 to 711.8) | 282.86 (167.34 to 447.14) | 723.17 (514.24 to 1033.62) | 413.03 (316.84 to 548.57) | 235.32 (138.6 to 338.47) | 596.98 (438.74 to 781.04) | -16.77% (-35.67% to 8.29%) | -16.81% (-42.03% to 15.81%) | -17.45% (-41.43% to 16.76%) |
|  | Environmental/occupational risks | DALYs | All age number | 1030.38 (610.53 to 1597.58) | 180.84 (95.72 to 293.99) | 849.54 (500.07 to 1361.69) | 2290.76 (1400.82 to 3409.18) | 531.05 (287.61 to 844.44) | 1759.71 (1073.58 to 2652.97) | 122.32% (38.78% to 227.9%) | 193.66% (67.86% to 392.62%) | 107.14% (22.97% to 226.65%) |
|  |  |  | Age-standardized rate (per 100,000) | 72.6 (42.88 to 112.65) | 26.54 (14.19 to 42.81) | 120.46 (69.48 to 191.29) | 68.26 (41.56 to 101.98) | 31.97 (17.3 to 50.71) | 105.56 (63.9 to 158.52) | -5.97% (-41.01% to 36.74%) | 20.46% (-30.47% to 101.11%) | -12.36% (-47.68% to 38.8%) |
|  |  | Deaths | All age number | 35.17 (20.58 to 55.09) | 6.27 (3.31 to 10.1) | 28.91 (16.95 to 46.29) | 87.33 (52.56 to 130.72) | 21.08 (11.33 to 33.63) | 66.25 (39.93 to 100.2) | 148.27% (54.24% to 263.82%) | 236.41% (95.08% to 467.92%) | 129.17% (35.54% to 259.95%) |
|  |  |  | Age-standardized rate (per 100,000) | 2.82 (1.66 to 4.43) | 1.07 (0.56 to 1.73) | 4.8 (2.76 to 7.79) | 2.78 (1.66 to 4.22) | 1.37 (0.73 to 2.18) | 4.24 (2.55 to 6.48) | -1.27% (-37.89% to 43.55%) | 27.84% (-24.34% to 114.95%) | -11.72% (-47.14% to 40.06%) |
|  |  | YLDs | All age number | 8.9 (4.75 to 14.96) | 1.53 (0.72 to 2.64) | 7.37 (3.71 to 12.67) | 21.89 (11.76 to 35.07) | 4.96 (2.41 to 8.89) | 16.93 (9.07 to 26.31) | 145.96% (61.01% to 280.75%) | 224.14% (81.74% to 463.29%) | 129.74% (37.75% to 289.96%) |
|  |  |  | Age-standardized rate (per 100,000) | 0.66 (0.35 to 1.1) | 0.24 (0.11 to 0.41) | 1.1 (0.58 to 1.89) | 0.66 (0.36 to 1.06) | 0.31 (0.15 to 0.55) | 1.03 (0.54 to 1.6) | 1.33% (-33.97% to 54.84%) | 27.87% (-29.21% to 124.22%) | -6.03% (-42.21% to 57.1%) |
|  |  | YLLs | All age number | 1021.48 (605.0 to 1581.61) | 179.31 (94.94 to 291.17) | 842.17 (495.01 to 1348.17) | 2268.87 (1390.36 to 3375.73) | 526.09 (284.67 to 835.92) | 1742.78 (1063.74 to 2630.26) | 122.12% (38.58% to 227.48%) | 193.4% (67.52% to 392.51%) | 106.94% (22.64% to 226.13%) |
|  |  |  | Age-standardized rate (per 100,000) | 71.94 (42.47 to 111.72) | 26.3 (14.06 to 42.35) | 119.36 (68.85 to 189.78) | 67.6 (41.25 to 100.96) | 31.66 (17.12 to 50.19) | 104.53 (63.33 to 156.94) | -6.04% (-41.08% to 36.65%) | 20.4% (-30.61% to 100.99%) | -12.42% (-47.78% to 38.71%) |
|  | Metabolic risks | DALYs | All age number | 856.04 (344.23 to 1399.75) | 478.71 (195.36 to 802.92) | 377.33 (147.33 to 641.17) | 4595.84 (1520.66 to 7967.5) | 2599.37 (822.63 to 4453.78) | 1996.47 (665.78 to 3470.37) | 436.87% (276.92% to 583.52%) | 442.99% (238.6% to 657.86%) | 429.11% (259.65% to 664.52%) |
|  |  |  | Age-standardized rate (per 100,000) | 61.49 (23.72 to 101.88) | 68.35 (26.4 to 117.6) | 54.57 (20.35 to 92.35) | 139.2 (46.64 to 241.39) | 154.5 (49.77 to 264.11) | 123.54 (40.69 to 214.79) | 126.37% (63.07% to 185.83%) | 126.04% (46.29% to 211.51%) | 126.4% (60.35% to 221.77%) |
|  |  | Deaths | All age number | 28.73 (10.82 to 48.01) | 16.13 (6.04 to 27.91) | 12.59 (4.5 to 21.43) | 178.1 (57.52 to 310.74) | 100.12 (31.09 to 172.22) | 77.98 (24.22 to 139.46) | 519.98% (344.3% to 683.73%) | 520.58% (301.69% to 745.78%) | 519.21% (331.76% to 787.55%) |
|  |  |  | Age-standardized rate (per 100,000) | 2.44 (0.86 to 4.09) | 2.65 (0.95 to 4.62) | 2.19 (0.76 to 3.78) | 5.81 (1.9 to 10.1) | 6.44 (2.03 to 11.15) | 5.19 (1.62 to 9.36) | 138.44% (74.42% to 197.67%) | 142.61% (63.58% to 225.82%) | 136.33% (72.2% to 228.21%) |
|  |  | YLDs | All age number | 20.49 (7.06 to 37.24) | 13.96 (3.97 to 26.06) | 6.54 (2.39 to 12.0) | 200.22 (53.9 to 355.71) | 145.98 (34.26 to 260.71) | 54.24 (18.75 to 99.31) | 876.99% (595.18% to 1218.48%) | 946.02% (560.24% to 1428.2%) | 729.65% (460.88% to 1124.31%) |
|  |  |  | Age-standardized rate (per 100,000) | 1.53 (0.5 to 2.79) | 2.05 (0.57 to 3.78) | 1.01 (0.35 to 1.84) | 5.94 (1.62 to 10.48) | 8.41 (2.0 to 15.14) | 3.39 (1.19 to 6.21) | 287.51% (181.43% to 411.98%) | 309.6% (167.86% to 493.53%) | 236.33% (137.6% to 383.04%) |
|  |  | YLLs | All age number | 835.54 (338.32 to 1364.72) | 464.76 (191.94 to 780.89) | 370.79 (144.94 to 629.76) | 4395.62 (1459.82 to 7557.9) | 2453.4 (788.13 to 4180.12) | 1942.23 (644.42 to 3381.81) | 426.08% (270.42% to 567.99%) | 427.89% (229.28% to 633.61%) | 423.81% (256.05% to 657.63%) |
|  |  |  | Age-standardized rate (per 100,000) | 59.96 (23.19 to 99.12) | 66.3 (25.94 to 114.11) | 53.56 (20.0 to 90.79) | 133.27 (44.81 to 228.79) | 146.09 (47.74 to 248.31) | 120.15 (39.33 to 209.51) | 122.26% (60.02% to 180.79%) | 120.36% (42.27% to 202.68%) | 124.33% (58.93% to 218.86%) |
| Golestan | All risk factors | DALYs | All age number | 4056.68 (2996.55 to 5318.41) | 1368.15 (870.25 to 1966.71) | 2688.53 (1901.5 to 3582.85) | 12085.69 (9190.86 to 15035.69) | 4347.15 (2850.67 to 5832.65) | 7738.55 (5861.55 to 9746.55) | 197.92% (133.77% to 279.0%) | 217.74% (124.5% to 357.01%) | 187.84% (105.84% to 313.39%) |
|  |  |  | Age-standardized rate (per 100,000) | 653.28 (486.48 to 853.78) | 430.41 (272.04 to 618.2) | 861.02 (622.1 to 1142.96) | 678.65 (519.31 to 841.91) | 454.13 (296.01 to 607.14) | 933.36 (712.04 to 1171.49) | 3.88% (-18.64% to 32.24%) | 5.51% (-23.76% to 50.04%) | 8.4% (-21.55% to 51.4%) |
|  |  | Deaths | All age number | 134.03 (99.54 to 175.24) | 40.86 (25.76 to 58.83) | 93.17 (66.68 to 123.87) | 423.38 (324.88 to 524.14) | 142.61 (94.47 to 190.44) | 280.77 (215.14 to 353.19) | 215.89% (149.37% to 302.13%) | 249.05% (153.1% to 398.52%) | 201.35% (117.85% to 322.54%) |
|  |  |  | Age-standardized rate (per 100,000) | 25.46 (18.83 to 33.48) | 15.6 (9.61 to 22.46) | 35.41 (26.11 to 46.84) | 26.91 (20.67 to 33.3) | 16.78 (11.2 to 22.48) | 38.69 (29.96 to 48.5) | 5.7% (-16.34% to 34.06%) | 7.6% (-22.02% to 50.65%) | 9.26% (-19.02% to 49.94%) |
|  |  | YLDs | All age number | 56.97 (37.05 to 82.4) | 25.19 (13.58 to 40.07) | 31.78 (20.34 to 47.58) | 275.37 (169.99 to 401.68) | 153.1 (74.53 to 246.86) | 122.27 (83.96 to 168.43) | 383.33% (271.44% to 519.72%) | 507.77% (321.89% to 761.61%) | 284.7% (177.0% to 437.3%) |
|  |  |  | Age-standardized rate (per 100,000) | 9.4 (6.12 to 13.51) | 7.97 (4.29 to 12.49) | 10.76 (6.99 to 15.82) | 15.23 (9.51 to 22.01) | 15.53 (7.76 to 24.96) | 15.26 (10.47 to 21.23) | 61.94% (25.31% to 104.86%) | 94.79% (39.05% to 169.06%) | 41.78% (3.59% to 93.25%) |
|  |  | YLLs | All age number | 3999.7 (2956.03 to 5240.91) | 1342.96 (855.42 to 1936.26) | 2656.74 (1877.56 to 3541.14) | 11810.32 (9018.01 to 14703.05) | 4194.04 (2773.15 to 5594.95) | 7616.27 (5778.1 to 9608.69) | 195.28% (131.72% to 275.14%) | 212.3% (121.01% to 349.17%) | 186.68% (104.88% to 311.3%) |
|  |  |  | Age-standardized rate (per 100,000) | 643.87 (480.19 to 842.28) | 422.43 (267.3 to 608.84) | 850.26 (613.25 to 1129.1) | 663.43 (508.33 to 823.53) | 438.59 (288.2 to 584.02) | 918.1 (700.02 to 1156.01) | 3.04% (-19.41% to 31.35%) | 3.83% (-25.03% to 47.61%) | 7.98% (-21.92% to 50.98%) |
|  | Behavioral risks | DALYs | All age number | 3565.58 (2555.09 to 4822.92) | 1127.48 (641.04 to 1691.69) | 2438.1 (1700.2 to 3310.58) | 9324.36 (6968.37 to 11920.07) | 2861.48 (1759.57 to 4082.28) | 6462.88 (4815.35 to 8327.22) | 161.51% (103.69% to 234.87%) | 153.79% (76.54% to 261.22%) | 165.08% (90.88% to 282.47%) |
|  |  |  | Age-standardized rate (per 100,000) | 575.36 (415.54 to 779.77) | 352.6 (199.08 to 522.16) | 782.27 (554.8 to 1060.26) | 522.04 (394.04 to 664.48) | 292.62 (180.36 to 414.91) | 781.31 (583.46 to 998.45) | -9.27% (-28.43% to 16.41%) | -17.01% (-40.93% to 16.9%) | -0.12% (-26.89% to 38.92%) |
|  |  | Deaths | All age number | 118.3 (85.49 to 160.62) | 33.36 (18.83 to 49.66) | 84.94 (59.94 to 114.89) | 326.75 (246.61 to 413.65) | 90.84 (56.84 to 128.5) | 235.91 (177.68 to 302.78) | 176.21% (117.0% to 253.46%) | 172.33% (96.54% to 273.81%) | 177.73% (103.03% to 289.77%) |
|  |  |  | Age-standardized rate (per 100,000) | 22.38 (16.04 to 30.41) | 12.64 (6.97 to 18.94) | 32.16 (23.35 to 43.62) | 20.7 (15.81 to 26.15) | 10.5 (6.59 to 14.88) | 32.5 (24.49 to 41.6) | -7.54% (-26.38% to 17.5%) | -16.91% (-38.89% to 14.39%) | 1.07% (-24.11% to 38.43%) |
|  |  | YLDs | All age number | 49.97 (31.2 to 74.48) | 20.95 (10.09 to 34.66) | 29.03 (18.32 to 44.52) | 203.17 (118.36 to 318.34) | 102.23 (42.66 to 178.44) | 100.94 (66.15 to 141.61) | 306.57% (207.92% to 420.23%) | 388.08% (196.58% to 624.31%) | 247.74% (153.13% to 389.65%) |
|  |  |  | Age-standardized rate (per 100,000) | 8.2 (5.15 to 12.24) | 6.48 (3.11 to 10.64) | 9.81 (6.35 to 14.74) | 11.11 (6.7 to 16.81) | 10.0 (4.31 to 17.31) | 12.63 (8.29 to 17.59) | 35.42% (4.5% to 72.56%) | 54.41% (-1.52% to 124.02%) | 28.77% (-6.33% to 81.64%) |
|  |  | YLLs | All age number | 3515.61 (2517.8 to 4762.83) | 1106.54 (631.63 to 1660.1) | 2409.07 (1675.72 to 3261.61) | 9121.19 (6842.26 to 11639.77) | 2759.25 (1718.58 to 3941.41) | 6361.94 (4736.13 to 8209.46) | 159.45% (102.03% to 232.02%) | 149.36% (74.04% to 254.74%) | 164.08% (90.19% to 280.78%) |
|  |  |  | Age-standardized rate (per 100,000) | 567.16 (410.13 to 769.82) | 346.12 (196.2 to 513.71) | 772.46 (547.07 to 1045.86) | 510.93 (386.83 to 649.03) | 282.62 (176.6 to 401.63) | 768.68 (574.83 to 985.42) | -9.91% (-28.97% to 15.59%) | -18.35% (-41.78% to 14.19%) | -0.49% (-27.15% to 38.52%) |
|  | Environmental/occupational risks | DALYs | All age number | 462.23 (275.78 to 714.98) | 91.53 (44.63 to 166.85) | 370.7 (225.73 to 582.48) | 1458.13 (851.23 to 2242.48) | 383.44 (210.97 to 629.3) | 1074.69 (642.45 to 1643.98) | 215.45% (107.81% to 402.13%) | 318.91% (111.99% to 701.61%) | 189.91% (88.16% to 383.19%) |
|  |  |  | Age-standardized rate (per 100,000) | 74.75 (44.22 to 116.89) | 30.35 (14.88 to 54.51) | 116.67 (71.63 to 180.58) | 81.64 (47.34 to 126.71) | 41.24 (22.68 to 67.79) | 125.97 (74.79 to 196.66) | 9.22% (-28.1% to 76.02%) | 35.89% (-30.1% to 158.62%) | 7.97% (-28.99% to 77.04%) |
|  |  | Deaths | All age number | 15.52 (9.18 to 24.21) | 2.94 (1.44 to 5.3) | 12.58 (7.67 to 19.48) | 51.35 (29.69 to 79.71) | 13.58 (7.47 to 22.21) | 37.77 (22.33 to 59.2) | 230.79% (119.29% to 436.14%) | 362.04% (136.57% to 784.27%) | 200.14% (96.89% to 399.66%) |
|  |  |  | Age-standardized rate (per 100,000) | 2.97 (1.75 to 4.63) | 1.2 (0.6 to 2.15) | 4.76 (2.86 to 7.36) | 3.24 (1.88 to 5.01) | 1.65 (0.9 to 2.69) | 5.03 (2.91 to 7.89) | 9.06% (-27.77% to 76.02%) | 37.41% (-28.24% to 161.3%) | 5.77% (-30.59% to 70.95%) |
|  |  | YLDs | All age number | 3.81 (2.12 to 6.51) | 0.73 (0.33 to 1.38) | 3.08 (1.68 to 5.33) | 12.61 (6.76 to 21.13) | 3.18 (1.49 to 5.7) | 9.43 (5.26 to 16.12) | 230.88% (107.96% to 446.3%) | 333.85% (118.57% to 765.55%) | 206.34% (86.31% to 432.81%) |
|  |  |  | Age-standardized rate (per 100,000) | 0.66 (0.37 to 1.12) | 0.27 (0.12 to 0.52) | 1.04 (0.58 to 1.82) | 0.74 (0.4 to 1.24) | 0.36 (0.17 to 0.65) | 1.16 (0.64 to 1.97) | 11.86% (-29.21% to 83.96%) | 33.85% (-32.47% to 169.4%) | 11.62% (-29.86% to 86.97%) |
|  |  | YLLs | All age number | 458.42 (273.35 to 708.75) | 90.8 (44.28 to 165.5) | 367.62 (223.96 to 577.4) | 1445.52 (842.85 to 2222.91) | 380.26 (209.22 to 624.38) | 1065.26 (636.54 to 1627.82) | 215.33% (107.67% to 401.68%) | 318.79% (111.95% to 701.92%) | 189.77% (88.03% to 383.37%) |
|  |  |  | Age-standardized rate (per 100,000) | 74.09 (43.76 to 115.77) | 30.08 (14.74 to 54.03) | 115.63 (70.99 to 178.82) | 80.9 (46.82 to 125.64) | 40.88 (22.48 to 67.23) | 124.81 (74.07 to 194.65) | 9.19% (-28.12% to 75.99%) | 35.9% (-30.03% to 158.88%) | 7.94% (-29.09% to 77.13%) |
|  | Metabolic risks | DALYs | All age number | 376.45 (166.51 to 601.43) | 202.94 (89.39 to 320.07) | 173.51 (70.13 to 294.39) | 2703.35 (922.71 to 4507.72) | 1432.19 (482.19 to 2359.06) | 1271.17 (442.04 to 2149.91) | 618.12% (436.34% to 781.67%) | 605.72% (369.25% to 890.05%) | 632.63% (419.24% to 918.53%) |
|  |  |  | Age-standardized rate (per 100,000) | 59.96 (24.49 to 97.88) | 66.07 (27.36 to 105.48) | 54.51 (20.7 to 93.43) | 154.26 (50.48 to 260.78) | 156.37 (50.91 to 259.07) | 153.05 (51.25 to 261.38) | 157.28% (98.49% to 213.89%) | 136.69% (65.4% to 224.67%) | 180.79% (100.74% to 285.74%) |
|  |  | Deaths | All age number | 12.16 (4.92 to 19.85) | 6.37 (2.64 to 10.21) | 5.79 (2.18 to 10.0) | 95.33 (30.49 to 163.04) | 50.0 (15.77 to 82.96) | 45.33 (14.81 to 77.3) | 683.81% (504.38% to 851.58%) | 684.74% (439.38% to 981.98%) | 682.79% (449.75% to 986.19%) |
|  |  |  | Age-standardized rate (per 100,000) | 2.34 (0.85 to 3.86) | 2.49 (0.96 to 4.05) | 2.21 (0.78 to 3.87) | 6.13 (1.9 to 10.55) | 6.06 (1.86 to 10.09) | 6.27 (1.99 to 10.77) | 162.44% (109.06% to 216.21%) | 143.74% (77.31% to 219.98%) | 183.81% (106.1% to 290.25%) |
|  |  | YLDs | All age number | 7.05 (2.43 to 12.43) | 4.48 (1.4 to 8.01) | 2.56 (0.95 to 4.79) | 83.2 (26.55 to 146.29) | 57.59 (16.82 to 104.89) | 25.61 (9.74 to 45.16) | 1080.86% (747.35% to 1450.3%) | 1184.96% (758.89% to 1784.77%) | 898.88% (596.77% to 1312.25%) |
|  |  |  | Age-standardized rate (per 100,000) | 1.21 (0.38 to 2.14) | 1.59 (0.45 to 2.84) | 0.87 (0.3 to 1.61) | 4.77 (1.48 to 8.39) | 6.28 (1.71 to 11.32) | 3.18 (1.16 to 5.64) | 294.72% (190.21% to 405.84%) | 295.6% (178.2% to 466.03%) | 263.49% (159.01% to 403.66%) |
|  |  | YLLs | All age number | 369.4 (163.65 to 589.8) | 198.46 (88.14 to 312.73) | 170.94 (69.2 to 290.15) | 2620.16 (900.17 to 4362.05) | 1374.6 (463.21 to 2254.66) | 1245.56 (432.57 to 2104.4) | 609.3% (427.81% to 770.47%) | 592.64% (358.59% to 872.11%) | 628.64% (416.16% to 913.34%) |
|  |  |  | Age-standardized rate (per 100,000) | 58.75 (24.07 to 95.91) | 64.48 (26.75 to 102.98) | 53.63 (20.39 to 91.84) | 149.49 (49.35 to 252.2) | 150.09 (48.97 to 248.15) | 149.88 (50.12 to 255.21) | 154.46% (97.32% to 210.29%) | 132.77% (62.67% to 219.34%) | 179.44% (99.49% to 283.88%) |
| Hamadan | All risk factors | DALYs | All age number | 4440.71 (3290.54 to 6186.79) | 1269.21 (809.88 to 1896.74) | 3171.49 (2226.35 to 4428.68) | 9373.61 (7264.47 to 11982.03) | 3056.64 (1893.55 to 4226.0) | 6316.96 (4868.52 to 8120.88) | 111.08% (54.58% to 181.87%) | 140.83% (65.18% to 238.73%) | 99.18% (36.54% to 191.83%) |
|  |  |  | Age-standardized rate (per 100,000) | 493.92 (363.85 to 688.16) | 287.52 (184.15 to 429.35) | 679.38 (477.44 to 950.21) | 488.7 (378.07 to 623.11) | 318.58 (198.88 to 438.72) | 662.38 (509.09 to 849.75) | -1.06% (-27.8% to 32.14%) | 10.81% (-22.44% to 55.26%) | -2.5% (-33.15% to 40.6%) |
|  |  | Deaths | All age number | 151.84 (112.38 to 210.33) | 39.64 (25.42 to 59.02) | 112.2 (78.96 to 155.51) | 350.43 (270.42 to 449.75) | 112.81 (71.42 to 154.46) | 237.62 (183.06 to 305.67) | 130.8% (68.48% to 207.01%) | 184.62% (99.4% to 295.87%) | 111.79% (46.11% to 205.89%) |
|  |  |  | Age-standardized rate (per 100,000) | 19.08 (14.07 to 26.74) | 10.58 (6.85 to 15.39) | 26.81 (19.35 to 38.07) | 19.09 (14.76 to 24.48) | 12.44 (7.91 to 17.04) | 25.93 (19.88 to 33.41) | 0.02% (-26.83% to 34.12%) | 17.54% (-15.97% to 62.7%) | -3.26% (-33.1% to 38.88%) |
|  |  | YLDs | All age number | 67.37 (42.51 to 97.21) | 26.62 (13.06 to 41.67) | 40.75 (25.93 to 60.49) | 236.66 (149.06 to 341.4) | 120.71 (56.54 to 194.66) | 115.95 (77.58 to 162.65) | 251.3% (160.24% to 353.23%) | 353.5% (203.86% to 519.82%) | 184.54% (94.98% to 306.6%) |
|  |  |  | Age-standardized rate (per 100,000) | 7.7 (4.9 to 11.01) | 6.11 (3.01 to 9.48) | 9.08 (5.86 to 13.21) | 12.3 (7.78 to 17.62) | 12.35 (5.84 to 19.75) | 12.34 (8.23 to 17.27) | 59.69% (19.6% to 104.48%) | 102.05% (35.93% to 173.86%) | 35.94% (-6.87% to 93.01%) |
|  |  | YLLs | All age number | 4373.34 (3237.95 to 6103.84) | 1242.59 (793.43 to 1858.44) | 3130.74 (2194.33 to 4381.09) | 9136.94 (7057.51 to 11673.16) | 2935.93 (1831.46 to 4026.55) | 6201.02 (4768.16 to 7975.39) | 108.92% (53.11% to 179.96%) | 136.27% (62.68% to 231.87%) | 98.07% (35.74% to 189.9%) |
|  |  |  | Age-standardized rate (per 100,000) | 486.22 (358.99 to 679.17) | 281.4 (180.73 to 420.7) | 670.3 (471.53 to 939.99) | 476.41 (369.39 to 607.49) | 306.23 (192.03 to 419.54) | 650.04 (498.85 to 834.25) | -2.02% (-28.43% to 31.22%) | 8.82% (-23.55% to 52.45%) | -3.02% (-33.53% to 40.01%) |
|  | Behavioral risks | DALYs | All age number | 3801.72 (2725.9 to 5526.11) | 970.38 (598.56 to 1498.0) | 2831.35 (1943.75 to 4046.39) | 7005.21 (5322.38 to 9041.18) | 1817.37 (1058.12 to 2654.06) | 5187.85 (3900.58 to 6834.65) | 84.26% (36.09% to 151.08%) | 87.29% (29.26% to 170.27%) | 83.23% (25.69% to 167.49%) |
|  |  |  | Age-standardized rate (per 100,000) | 424.59 (306.97 to 616.47) | 219.54 (134.81 to 336.89) | 608.86 (421.44 to 881.9) | 363.96 (277.54 to 467.11) | 186.56 (110.07 to 271.16) | 544.82 (409.35 to 717.81) | -14.28% (-36.59% to 15.81%) | -15.02% (-40.05% to 21.47%) | -10.52% (-38.25% to 30.26%) |
|  |  | Deaths | All age number | 130.91 (94.83 to 188.9) | 30.02 (18.49 to 46.44) | 100.89 (69.75 to 145.15) | 260.87 (197.11 to 336.32) | 64.47 (38.56 to 95.07) | 196.4 (147.42 to 260.48) | 99.28% (45.36% to 169.77%) | 114.74% (56.38% to 201.64%) | 94.68% (33.32% to 180.23%) |
|  |  |  | Age-standardized rate (per 100,000) | 16.42 (11.94 to 23.51) | 7.99 (4.94 to 12.43) | 24.09 (16.83 to 35.14) | 14.17 (10.68 to 18.35) | 7.04 (4.25 to 10.34) | 21.47 (16.1 to 28.36) | -13.73% (-36.42% to 14.78%) | -11.95% (-34.42% to 22.04%) | -10.87% (-38.64% to 26.07%) |
|  |  | YLDs | All age number | 57.98 (36.28 to 86.03) | 21.02 (9.81 to 34.6) | 36.96 (23.59 to 55.15) | 170.66 (103.74 to 254.03) | 75.91 (28.09 to 135.7) | 94.75 (62.49 to 135.38) | 194.33% (114.2% to 284.1%) | 261.05% (125.33% to 413.37%) | 156.38% (75.81% to 267.79%) |
|  |  |  | Age-standardized rate (per 100,000) | 6.62 (4.13 to 9.59) | 4.76 (2.25 to 7.75) | 8.23 (5.33 to 12.09) | 8.8 (5.49 to 13.04) | 7.58 (2.88 to 13.45) | 10.11 (6.67 to 14.33) | 32.99% (-2.27% to 71.36%) | 59.31% (0.9% to 125.4%) | 22.85% (-16.18% to 76.06%) |
|  |  | YLLs | All age number | 3743.74 (2689.62 to 5453.27) | 949.35 (587.73 to 1470.28) | 2794.39 (1920.38 to 3999.82) | 6834.56 (5219.7 to 8812.71) | 1741.46 (1028.79 to 2549.05) | 5093.09 (3825.26 to 6721.2) | 82.56% (34.5% to 149.34%) | 83.44% (27.08% to 163.62%) | 82.26% (24.8% to 166.33%) |
|  |  |  | Age-standardized rate (per 100,000) | 417.97 (302.9 to 607.73) | 214.78 (132.16 to 331.68) | 600.62 (415.89 to 871.42) | 355.16 (271.62 to 455.42) | 178.98 (106.56 to 262.25) | 534.71 (401.38 to 705.44) | -15.03% (-37.18% to 14.95%) | -16.67% (-40.8% to 19.23%) | -10.97% (-38.65% to 29.72%) |
|  | Environmental/occupational risks | DALYs | All age number | 676.9 (396.8 to 1048.6) | 108.85 (55.06 to 185.28) | 568.06 (331.13 to 884.51) | 1426.95 (860.1 to 2218.56) | 319.62 (171.71 to 555.11) | 1107.33 (674.53 to 1692.85) | 110.81% (35.36% to 236.25%) | 193.64% (67.2% to 413.97%) | 94.93% (19.91% to 232.85%) |
|  |  |  | Age-standardized rate (per 100,000) | 74.2 (43.24 to 115.46) | 25.47 (13.04 to 42.36) | 118.51 (68.36 to 184.71) | 74.16 (44.29 to 115.25) | 33.74 (18.03 to 58.5) | 114.93 (68.98 to 176.71) | -0.04% (-35.48% to 58.85%) | 32.48% (-23.17% to 130.36%) | -3.02% (-40.21% to 64.88%) |
|  |  | Deaths | All age number | 23.12 (13.38 to 35.97) | 3.62 (1.85 to 6.01) | 19.5 (11.22 to 30.47) | 52.93 (30.6 to 82.96) | 12.78 (6.69 to 22.13) | 40.15 (23.63 to 61.77) | 128.93% (46.88% to 265.14%) | 252.92% (103.97% to 510.39%) | 105.9% (26.7% to 249.1%) |
|  |  |  | Age-standardized rate (per 100,000) | 2.84 (1.64 to 4.44) | 1.02 (0.52 to 1.68) | 4.51 (2.55 to 7.09) | 2.87 (1.64 to 4.53) | 1.43 (0.74 to 2.46) | 4.34 (2.55 to 6.7) | 0.91% (-35.17% to 60.04%) | 40.55% (-18.28% to 145.43%) | -3.81% (-41.4% to 61.19%) |
|  |  | YLDs | All age number | 5.7 (3.2 to 9.67) | 0.9 (0.41 to 1.59) | 4.8 (2.6 to 8.3) | 12.94 (7.37 to 20.94) | 2.87 (1.41 to 5.22) | 10.07 (5.53 to 16.12) | 127.0% (43.37% to 279.47%) | 219.91% (77.31% to 457.67%) | 109.63% (24.89% to 272.39%) |
|  |  |  | Age-standardized rate (per 100,000) | 0.66 (0.36 to 1.11) | 0.23 (0.11 to 0.41) | 1.05 (0.56 to 1.81) | 0.69 (0.39 to 1.12) | 0.31 (0.15 to 0.56) | 1.06 (0.58 to 1.72) | 4.13% (-33.65% to 72.83%) | 35.26% (-26.89% to 138.68%) | 1.6% (-39.36% to 79.4%) |
|  |  | YLLs | All age number | 671.2 (394.19 to 1040.86) | 107.95 (54.59 to 183.82) | 563.25 (328.37 to 877.24) | 1414.01 (852.93 to 2197.24) | 316.75 (170.21 to 549.23) | 1097.26 (668.08 to 1676.94) | 110.67% (35.23% to 235.94%) | 193.42% (67.12% to 414.08%) | 94.81% (19.84% to 232.62%) |
|  |  |  | Age-standardized rate (per 100,000) | 73.54 (42.87 to 114.38) | 25.24 (12.91 to 41.97) | 117.47 (67.76 to 183.2) | 73.48 (43.92 to 114.09) | 33.43 (17.88 to 57.87) | 113.87 (68.39 to 175.22) | -0.08% (-35.55% to 58.74%) | 32.46% (-23.23% to 130.17%) | -3.06% (-40.24% to 64.93%) |
|  | Metabolic risks | DALYs | All age number | 455.22 (196.1 to 722.02) | 253.83 (110.55 to 419.71) | 201.39 (75.18 to 338.07) | 2188.32 (745.45 to 3597.21) | 1168.4 (390.29 to 1950.33) | 1019.92 (345.93 to 1685.01) | 380.72% (224.74% to 526.29%) | 360.31% (176.08% to 528.02%) | 406.44% (233.71% to 627.27%) |
|  |  |  | Age-standardized rate (per 100,000) | 49.59 (20.09 to 79.68) | 57.79 (23.37 to 96.6) | 42.01 (14.96 to 70.71) | 115.71 (38.8 to 190.62) | 124.81 (40.84 to 209.18) | 106.87 (35.31 to 176.01) | 133.32% (62.24% to 205.83%) | 115.95% (38.87% to 192.51%) | 154.41% (73.97% to 262.09%) |
|  |  | Deaths | All age number | 14.97 (5.77 to 24.32) | 8.19 (3.25 to 13.85) | 6.78 (2.29 to 11.62) | 83.4 (26.3 to 138.92) | 45.51 (14.23 to 77.3) | 37.89 (11.52 to 63.84) | 457.0% (286.78% to 615.07%) | 455.68% (244.65% to 649.72%) | 458.61% (273.12% to 698.16%) |
|  |  |  | Age-standardized rate (per 100,000) | 1.89 (0.68 to 3.13) | 2.18 (0.84 to 3.71) | 1.62 (0.52 to 2.79) | 4.59 (1.43 to 7.66) | 5.09 (1.58 to 8.7) | 4.13 (1.23 to 6.99) | 142.84% (76.39% to 210.15%) | 133.1% (52.36% to 208.28%) | 155.15% (77.03% to 255.85%) |
|  |  | YLDs | All age number | 9.04 (2.93 to 16.01) | 5.93 (1.82 to 10.9) | 3.11 (1.08 to 5.43) | 74.15 (22.32 to 129.04) | 50.25 (13.36 to 88.87) | 23.9 (9.06 to 40.89) | 720.0% (477.49% to 989.45%) | 746.98% (442.68% to 1044.98%) | 668.54% (420.55% to 1027.21%) |
|  |  |  | Age-standardized rate (per 100,000) | 1.04 (0.31 to 1.87) | 1.43 (0.41 to 2.64) | 0.69 (0.23 to 1.21) | 3.94 (1.17 to 6.86) | 5.36 (1.38 to 9.56) | 2.52 (0.94 to 4.34) | 277.64% (172.68% to 397.13%) | 273.73% (148.0% to 404.73%) | 264.48% (148.07% to 436.71%) |
|  |  | YLLs | All age number | 446.18 (193.08 to 709.67) | 247.89 (108.16 to 407.61) | 198.28 (74.08 to 331.73) | 2114.17 (719.9 to 3469.98) | 1118.15 (375.44 to 1866.56) | 996.02 (337.07 to 1651.48) | 373.84% (219.16% to 513.97%) | 351.06% (171.13% to 517.61%) | 402.32% (230.65% to 623.06%) |
|  |  |  | Age-standardized rate (per 100,000) | 48.55 (19.78 to 78.2) | 56.36 (23.06 to 93.8) | 41.31 (14.74 to 69.59) | 111.77 (37.48 to 184.03) | 119.45 (39.31 to 200.17) | 104.35 (34.41 to 172.31) | 130.22% (59.91% to 201.59%) | 111.94% (36.35% to 187.91%) | 152.56% (72.48% to 260.51%) |
| Hormozgan | All risk factors | DALYs | All age number | 1821.0 (1386.61 to 2386.01) | 656.05 (440.51 to 899.83) | 1164.95 (839.4 to 1620.96) | 5096.92 (3856.43 to 6448.25) | 2039.13 (1306.05 to 2855.14) | 3057.79 (2324.02 to 4048.61) | 179.9% (101.47% to 272.48%) | 210.82% (111.71% to 346.05%) | 162.48% (75.09% to 284.85%) |
|  |  |  | Age-standardized rate (per 100,000) | 414.82 (316.59 to 543.47) | 308.84 (209.54 to 423.04) | 503.49 (368.21 to 694.75) | 358.2 (274.55 to 453.2) | 274.65 (176.26 to 382.67) | 446.26 (341.24 to 591.57) | -13.65% (-37.82% to 13.47%) | -11.07% (-39.75% to 26.54%) | -11.37% (-40.73% to 29.02%) |
|  |  | Deaths | All age number | 60.5 (46.17 to 80.24) | 19.69 (13.29 to 27.12) | 40.82 (29.66 to 57.05) | 172.39 (132.63 to 218.79) | 64.85 (42.5 to 90.6) | 107.53 (82.69 to 141.42) | 184.92% (106.35% to 274.16%) | 229.43% (124.91% to 363.28%) | 163.45% (77.52% to 285.16%) |
|  |  |  | Age-standardized rate (per 100,000) | 15.56 (11.91 to 20.64) | 10.65 (7.33 to 14.65) | 20.02 (14.77 to 27.72) | 14.09 (10.86 to 17.81) | 10.21 (6.69 to 14.07) | 18.18 (14.08 to 23.74) | -9.44% (-33.97% to 18.07%) | -4.16% (-32.47% to 33.5%) | -9.18% (-38.06% to 29.89%) |
|  |  | YLDs | All age number | 28.22 (18.89 to 39.83) | 13.08 (7.29 to 21.63) | 15.14 (9.44 to 22.66) | 142.92 (82.93 to 221.83) | 86.5 (40.24 to 147.1) | 56.42 (36.92 to 83.09) | 406.5% (246.83% to 575.57%) | 561.23% (334.16% to 843.47%) | 272.78% (154.7% to 455.74%) |
|  |  |  | Age-standardized rate (per 100,000) | 6.59 (4.44 to 9.31) | 6.26 (3.49 to 10.23) | 6.85 (4.32 to 10.01) | 9.87 (5.9 to 15.08) | 11.28 (5.38 to 18.73) | 8.58 (5.59 to 12.49) | 49.73% (5.88% to 97.53%) | 80.1% (18.3% to 149.27%) | 25.17% (-14.7% to 82.55%) |
|  |  | YLLs | All age number | 1792.78 (1360.83 to 2356.05) | 642.97 (431.09 to 882.34) | 1149.81 (827.72 to 1602.23) | 4954.0 (3781.39 to 6295.43) | 1952.63 (1256.25 to 2733.35) | 3001.37 (2283.62 to 3967.6) | 176.33% (99.15% to 268.37%) | 203.69% (107.63% to 333.41%) | 161.03% (73.98% to 283.15%) |
|  |  |  | Age-standardized rate (per 100,000) | 408.23 (311.12 to 534.98) | 302.58 (205.49 to 414.91) | 496.63 (363.28 to 686.26) | 348.33 (266.64 to 441.42) | 263.37 (171.11 to 367.91) | 437.68 (334.42 to 579.93) | -14.67% (-38.5% to 11.72%) | -12.96% (-41.28% to 24.08%) | -11.87% (-41.1% to 28.32%) |
|  | Behavioral risks | DALYs | All age number | 1559.86 (1158.63 to 2136.78) | 535.71 (337.44 to 766.36) | 1024.15 (720.42 to 1475.4) | 3801.43 (2804.16 to 5027.07) | 1361.13 (821.7 to 1994.08) | 2440.3 (1828.96 to 3277.38) | 143.7% (80.52% to 224.31%) | 154.08% (70.77% to 262.3%) | 138.28% (61.96% to 248.14%) |
|  |  |  | Age-standardized rate (per 100,000) | 356.08 (265.23 to 485.25) | 250.24 (161.74 to 359.55) | 444.17 (314.53 to 639.84) | 266.15 (199.36 to 349.32) | 175.81 (107.79 to 253.19) | 361.4 (271.63 to 479.43) | -25.26% (-44.02% to -1.37%) | -29.74% (-51.93% to -0.71%) | -18.63% (-44.78% to 18.6%) |
|  |  | Deaths | All age number | 51.93 (38.4 to 71.7) | 15.79 (10.25 to 22.42) | 36.14 (25.51 to 52.12) | 128.07 (96.97 to 167.15) | 41.18 (25.71 to 59.21) | 86.89 (65.13 to 115.15) | 146.61% (81.73% to 228.3%) | 160.72% (80.96% to 263.58%) | 140.44% (64.94% to 249.57%) |
|  |  |  | Age-standardized rate (per 100,000) | 13.3 (9.96 to 18.56) | 8.47 (5.49 to 12.03) | 17.66 (12.49 to 25.2) | 10.4 (7.88 to 13.54) | 6.26 (4.03 to 9.0) | 14.76 (11.12 to 19.52) | -21.8% (-41.76% to 3.0%) | -26.09% (-47.48% to 1.35%) | -16.41% (-41.62% to 18.4%) |
|  |  | YLDs | All age number | 24.24 (15.92 to 35.42) | 10.74 (5.63 to 18.61) | 13.49 (8.49 to 20.08) | 104.33 (54.87 to 167.33) | 59.74 (22.38 to 110.28) | 44.59 (29.11 to 66.09) | 330.43% (194.98% to 492.84%) | 456.02% (229.58% to 727.26%) | 230.43% (125.02% to 394.14%) |
|  |  |  | Age-standardized rate (per 100,000) | 5.63 (3.73 to 8.09) | 5.02 (2.65 to 8.78) | 6.11 (3.86 to 9.07) | 6.98 (3.88 to 10.89) | 7.22 (2.87 to 13.17) | 6.9 (4.49 to 10.21) | 23.97% (-10.02% to 66.95%) | 43.75% (-11.14% to 106.05%) | 12.92% (-22.41% to 66.09%) |
|  |  | YLLs | All age number | 1535.62 (1141.78 to 2105.97) | 524.96 (331.31 to 749.8) | 1010.65 (711.3 to 1457.73) | 3697.1 (2739.46 to 4887.51) | 1301.39 (794.36 to 1874.7) | 2395.71 (1793.28 to 3226.19) | 140.76% (78.19% to 220.75%) | 147.9% (67.68% to 251.78%) | 137.05% (60.99% to 247.02%) |
|  |  |  | Age-standardized rate (per 100,000) | 350.46 (260.98 to 478.61) | 245.22 (158.42 to 351.67) | 438.05 (309.66 to 631.75) | 259.17 (194.99 to 339.98) | 168.59 (104.46 to 240.87) | 354.5 (266.01 to 471.58) | -26.05% (-44.68% to -2.21%) | -31.25% (-52.75% to -2.95%) | -19.07% (-45.11% to 17.94%) |
|  | Environmental/occupational risks | DALYs | All age number | 300.54 (184.8 to 453.67) | 52.32 (26.98 to 90.84) | 248.22 (149.67 to 382.77) | 832.85 (505.88 to 1209.39) | 207.17 (118.16 to 322.44) | 625.68 (384.79 to 922.69) | 177.12% (72.83% to 339.14%) | 295.97% (100.93% to 665.25%) | 152.07% (52.24% to 320.77%) |
|  |  |  | Age-standardized rate (per 100,000) | 68.97 (42.37 to 104.1) | 26.12 (13.42 to 44.96) | 105.52 (63.59 to 162.22) | 58.77 (35.61 to 86.28) | 29.31 (16.68 to 45.74) | 88.86 (53.57 to 131.8) | -14.79% (-47.19% to 35.13%) | 12.21% (-42.74% to 120.41%) | -15.79% (-48.68% to 38.51%) |
|  |  | Deaths | All age number | 10.3 (6.31 to 15.6) | 1.75 (0.9 to 3.01) | 8.55 (5.16 to 13.17) | 28.76 (17.54 to 42.19) | 7.34 (4.18 to 11.49) | 21.42 (12.99 to 31.69) | 179.09% (72.16% to 345.17%) | 318.75% (114.26% to 718.98%) | 150.45% (51.64% to 312.21%) |
|  |  |  | Age-standardized rate (per 100,000) | 2.66 (1.63 to 4.05) | 1.02 (0.51 to 1.75) | 4.13 (2.49 to 6.48) | 2.35 (1.44 to 3.45) | 1.22 (0.69 to 1.92) | 3.52 (2.11 to 5.19) | -11.57% (-45.07% to 40.89%) | 19.9% (-38.58% to 136.45%) | -14.82% (-47.93% to 37.85%) |
|  |  | YLDs | All age number | 2.53 (1.38 to 4.3) | 0.44 (0.21 to 0.75) | 2.09 (1.08 to 3.62) | 7.32 (4.29 to 11.44) | 1.8 (0.93 to 3.1) | 5.52 (3.18 to 8.72) | 190.04% (79.21% to 392.99%) | 313.01% (112.33% to 690.83%) | 164.38% (55.44% to 398.52%) |
|  |  |  | Age-standardized rate (per 100,000) | 0.61 (0.34 to 1.03) | 0.24 (0.11 to 0.41) | 0.93 (0.51 to 1.63) | 0.55 (0.32 to 0.87) | 0.27 (0.14 to 0.47) | 0.83 (0.47 to 1.33) | -10.04% (-44.04% to 53.33%) | 15.81% (-40.84% to 127.73%) | -11.14% (-46.86% to 54.8%) |
|  |  | YLLs | All age number | 298.01 (182.88 to 449.89) | 51.88 (26.78 to 89.97) | 246.13 (148.25 to 379.88) | 825.53 (501.15 to 1199.52) | 205.36 (116.98 to 320.04) | 620.16 (381.41 to 914.1) | 177.01% (72.76% to 338.76%) | 295.82% (100.81% to 665.03%) | 151.97% (52.15% to 320.61%) |
|  |  |  | Age-standardized rate (per 100,000) | 68.36 (41.9 to 103.3) | 25.88 (13.3 to 44.56) | 104.58 (63.05 to 160.95) | 58.22 (35.26 to 85.48) | 29.04 (16.51 to 45.26) | 88.03 (53.09 to 130.45) | -14.83% (-47.23% to 34.99%) | 12.18% (-42.76% to 120.43%) | -15.83% (-48.71% to 38.53%) |
|  | Metabolic risks | DALYs | All age number | 174.78 (84.37 to 275.29) | 93.84 (45.2 to 153.54) | 80.94 (35.61 to 135.62) | 1127.88 (396.25 to 1837.21) | 605.83 (200.39 to 1019.92) | 522.05 (190.33 to 878.59) | 545.33% (341.16% to 744.08%) | 545.63% (288.57% to 819.55%) | 544.98% (347.22% to 839.16%) |
|  |  |  | Age-standardized rate (per 100,000) | 39.4 (17.16 to 62.29) | 45.93 (20.43 to 76.18) | 34.06 (14.07 to 57.78) | 82.2 (26.61 to 136.9) | 89.95 (27.55 to 155.39) | 74.1 (24.44 to 125.76) | 108.63% (38.82% to 167.35%) | 95.83% (20.77% to 175.92%) | 117.55% (52.21% to 210.78%) |
|  |  | Deaths | All age number | 5.75 (2.39 to 9.15) | 3.04 (1.32 to 5.06) | 2.71 (1.07 to 4.64) | 39.17 (12.64 to 65.18) | 21.25 (6.6 to 36.87) | 17.91 (5.78 to 30.46) | 581.11% (368.06% to 776.25%) | 599.94% (346.33% to 879.94%) | 560.04% (360.15% to 847.7%) |
|  |  |  | Age-standardized rate (per 100,000) | 1.49 (0.59 to 2.42) | 1.69 (0.68 to 2.82) | 1.33 (0.49 to 2.32) | 3.3 (1.0 to 5.55) | 3.57 (1.03 to 6.25) | 3.02 (0.92 to 5.2) | 120.91% (54.32% to 179.48%) | 111.31% (35.87% to 191.1%) | 126.29% (59.62% to 210.28%) |
|  |  | YLDs | All age number | 3.71 (1.32 to 6.34) | 2.38 (0.75 to 4.3) | 1.33 (0.54 to 2.39) | 42.38 (12.21 to 72.1) | 29.5 (7.05 to 53.23) | 12.89 (5.51 to 22.96) | 1042.01% (688.53% to 1426.95%) | 1141.32% (673.08% to 1737.82%) | 865.23% (570.1% to 1332.23%) |
|  |  |  | Age-standardized rate (per 100,000) | 0.9 (0.3 to 1.55) | 1.27 (0.35 to 2.32) | 0.6 (0.24 to 1.05) | 3.22 (0.83 to 5.58) | 4.52 (0.99 to 8.13) | 1.87 (0.74 to 3.37) | 258.07% (143.05% to 367.08%) | 256.53% (123.34% to 415.6%) | 211.36% (118.33% to 351.58%) |
|  |  | YLLs | All age number | 171.07 (82.7 to 269.81) | 91.46 (44.53 to 149.42) | 79.61 (35.13 to 133.26) | 1085.5 (383.96 to 1761.75) | 576.33 (194.93 to 979.13) | 509.17 (186.02 to 860.65) | 534.55% (334.84% to 730.95%) | 530.15% (279.86% to 800.87%) | 539.61% (342.86% to 831.92%) |
|  |  |  | Age-standardized rate (per 100,000) | 38.5 (16.87 to 60.67) | 44.67 (20.01 to 74.09) | 33.46 (13.86 to 56.69) | 78.98 (25.8 to 131.39) | 85.44 (26.59 to 148.73) | 72.22 (23.73 to 123.27) | 105.14% (36.65% to 163.22%) | 91.27% (18.47% to 170.86%) | 115.86% (50.7% to 208.06%) |
| Ilam | All risk factors | DALYs | All age number | 1101.33 (793.48 to 1580.62) | 301.33 (194.48 to 467.1) | 800.0 (555.16 to 1163.99) | 3253.56 (2501.9 to 4170.36) | 1152.03 (748.68 to 1626.28) | 2101.53 (1612.74 to 2677.53) | 195.42% (123.93% to 304.25%) | 282.31% (169.69% to 473.86%) | 162.69% (89.05% to 286.11%) |
|  |  |  | Age-standardized rate (per 100,000) | 604.52 (438.66 to 871.48) | 352.79 (225.75 to 541.55) | 792.9 (556.76 to 1152.96) | 588.66 (452.87 to 750.45) | 391.03 (254.64 to 549.76) | 804.95 (619.12 to 1019.14) | -2.62% (-25.52% to 33.04%) | 10.84% (-21.39% to 66.38%) | 1.52% (-26.61% to 46.89%) |
|  |  | Deaths | All age number | 38.52 (27.99 to 55.34) | 9.44 (6.07 to 14.5) | 29.08 (20.31 to 42.68) | 120.62 (93.46 to 153.75) | 39.79 (26.24 to 55.36) | 80.83 (62.04 to 101.86) | 213.13% (139.43% to 325.07%) | 321.6% (201.51% to 524.32%) | 177.93% (103.35% to 296.9%) |
|  |  |  | Age-standardized rate (per 100,000) | 24.27 (17.71 to 34.96) | 13.15 (8.37 to 20.47) | 33.05 (23.36 to 48.1) | 24.9 (19.3 to 31.78) | 15.27 (10.09 to 21.23) | 35.58 (27.37 to 44.76) | 2.59% (-20.87% to 38.07%) | 16.11% (-16.67% to 65.54%) | 7.65% (-20.36% to 50.67%) |
|  |  | YLDs | All age number | 15.99 (10.56 to 23.7) | 5.7 (3.1 to 8.84) | 10.3 (6.68 to 15.7) | 83.26 (52.74 to 119.65) | 43.18 (21.91 to 71.11) | 40.08 (27.46 to 57.43) | 420.58% (291.59% to 583.28%) | 657.88% (416.58% to 1033.65%) | 289.26% (182.73% to 462.08%) |
|  |  |  | Age-standardized rate (per 100,000) | 8.99 (6.0 to 13.23) | 6.69 (3.52 to 10.43) | 10.65 (6.99 to 16.1) | 14.83 (9.53 to 21.33) | 14.18 (7.42 to 22.91) | 15.76 (10.94 to 22.21) | 64.96% (25.37% to 114.9%) | 111.86% (47.44% to 211.46%) | 47.94% (7.19% to 111.64%) |
|  |  | YLLs | All age number | 1085.33 (780.67 to 1559.97) | 295.63 (190.75 to 458.89) | 789.7 (546.9 to 1151.32) | 3170.3 (2444.15 to 4071.61) | 1108.84 (727.48 to 1551.68) | 2061.46 (1579.53 to 2615.48) | 192.1% (121.31% to 300.08%) | 275.07% (164.85% to 462.87%) | 161.04% (87.65% to 283.84%) |
|  |  |  | Age-standardized rate (per 100,000) | 595.53 (432.2 to 859.7) | 346.1 (221.73 to 531.65) | 782.24 (548.75 to 1140.91) | 573.83 (443.0 to 733.04) | 376.85 (247.57 to 525.98) | 789.19 (604.69 to 998.92) | -3.64% (-26.35% to 31.93%) | 8.88% (-22.85% to 63.44%) | 0.89% (-27.03% to 46.03%) |
|  | Behavioral risks | DALYs | All age number | 955.99 (667.41 to 1428.48) | 241.87 (144.14 to 399.22) | 714.12 (480.22 to 1077.25) | 2430.8 (1843.81 to 3319.17) | 717.3 (443.86 to 1102.37) | 1713.49 (1282.22 to 2258.05) | 154.27% (98.11% to 247.7%) | 196.57% (109.76% to 335.05%) | 139.94% (72.62% to 252.89%) |
|  |  |  | Age-standardized rate (per 100,000) | 525.94 (367.53 to 781.29) | 281.12 (164.49 to 461.68) | 708.56 (484.21 to 1073.83) | 439.38 (334.41 to 602.41) | 238.24 (150.06 to 370.72) | 659.77 (496.56 to 868.89) | -16.46% (-35.02% to 12.75%) | -15.25% (-38.58% to 23.12%) | -6.89% (-31.9% to 35.29%) |
|  |  | Deaths | All age number | 33.53 (23.35 to 49.89) | 7.5 (4.37 to 12.4) | 26.03 (17.69 to 39.47) | 90.3 (68.92 to 124.96) | 23.96 (15.23 to 37.24) | 66.34 (50.13 to 87.11) | 169.3% (110.58% to 260.79%) | 219.57% (135.14% to 357.51%) | 154.83% (88.48% to 267.1%) |
|  |  |  | Age-standardized rate (per 100,000) | 21.05 (14.65 to 31.48) | 10.36 (5.96 to 17.16) | 29.45 (20.24 to 44.34) | 18.59 (14.17 to 25.67) | 9.05 (5.76 to 14.0) | 29.18 (22.32 to 38.21) | -11.7% (-30.49% to 16.53%) | -12.68% (-35.12% to 22.39%) | -0.92% (-26.47% to 38.69%) |
|  |  | YLDs | All age number | 13.99 (8.81 to 21.4) | 4.7 (2.42 to 7.67) | 9.29 (5.93 to 14.56) | 60.34 (36.66 to 90.42) | 28.15 (11.36 to 49.27) | 32.2 (21.33 to 47.51) | 331.31% (224.5% to 461.92%) | 498.72% (271.03% to 768.77%) | 246.59% (147.88% to 398.79%) |
|  |  |  | Age-standardized rate (per 100,000) | 7.83 (4.98 to 11.73) | 5.37 (2.79 to 8.71) | 9.59 (6.15 to 15.03) | 10.61 (6.7 to 15.75) | 8.81 (3.61 to 15.18) | 12.76 (8.58 to 18.73) | 35.51% (2.95% to 72.8%) | 64.19% (6.45% to 133.73%) | 32.99% (-3.76% to 88.07%) |
|  |  | YLLs | All age number | 942.0 (658.49 to 1406.69) | 237.17 (141.85 to 392.6) | 704.83 (472.85 to 1063.67) | 2370.45 (1796.73 to 3251.66) | 689.16 (433.61 to 1062.56) | 1681.29 (1258.44 to 2213.52) | 151.64% (96.13% to 243.93%) | 190.58% (105.25% to 324.74%) | 138.54% (71.63% to 251.07%) |
|  |  |  | Age-standardized rate (per 100,000) | 518.11 (361.74 to 768.77) | 275.75 (161.6 to 455.48) | 698.97 (476.92 to 1060.83) | 428.78 (325.88 to 589.26) | 229.43 (145.94 to 356.32) | 647.01 (487.47 to 849.66) | -17.24% (-35.66% to 11.72%) | -16.8% (-39.34% to 20.39%) | -7.43% (-32.32% to 34.49%) |
|  | Environmental/occupational risks | DALYs | All age number | 174.35 (114.29 to 258.17) | 29.26 (16.0 to 47.52) | 145.09 (92.53 to 223.16) | 482.79 (320.77 to 692.36) | 133.68 (73.2 to 215.01) | 349.1 (231.25 to 493.54) | 176.91% (76.84% to 341.07%) | 356.95% (146.73% to 728.06%) | 140.61% (46.67% to 295.66%) |
|  |  |  | Age-standardized rate (per 100,000) | 97.03 (64.16 to 143.26) | 36.27 (19.69 to 58.38) | 143.64 (92.22 to 219.71) | 88.49 (59.26 to 126.37) | 46.34 (25.24 to 74.37) | 134.01 (89.76 to 189.3) | -8.8% (-41.96% to 43.13%) | 27.77% (-30.52% to 139.11%) | -6.71% (-42.52% to 52.55%) |
|  |  | Deaths | All age number | 6.29 (4.17 to 9.29) | 1.0 (0.54 to 1.6) | 5.29 (3.4 to 8.07) | 18.64 (12.54 to 26.44) | 4.97 (2.73 to 7.95) | 13.67 (9.1 to 19.32) | 196.51% (88.46% to 364.04%) | 399.13% (173.65% to 834.15%) | 158.36% (60.58% to 317.35%) |
|  |  |  | Age-standardized rate (per 100,000) | 4.04 (2.7 to 6.01) | 1.48 (0.79 to 2.35) | 6.08 (3.93 to 9.29) | 3.91 (2.63 to 5.53) | 1.96 (1.08 to 3.16) | 6.05 (4.05 to 8.47) | -3.11% (-37.59% to 51.19%) | 32.53% (-27.49% to 149.05%) | -0.41% (-37.26% to 59.27%) |
|  |  | YLDs | All age number | 1.54 (0.87 to 2.59) | 0.24 (0.12 to 0.42) | 1.29 (0.73 to 2.21) | 4.62 (2.66 to 7.01) | 1.18 (0.57 to 2.09) | 3.44 (2.01 to 5.31) | 200.58% (85.05% to 393.75%) | 386.25% (145.18% to 810.78%) | 165.76% (61.27% to 357.02%) |
|  |  |  | Age-standardized rate (per 100,000) | 0.91 (0.53 to 1.53) | 0.33 (0.15 to 0.57) | 1.36 (0.8 to 2.31) | 0.89 (0.52 to 1.35) | 0.43 (0.2 to 0.77) | 1.39 (0.81 to 2.15) | -1.95% (-39.75% to 59.91%) | 31.2% (-34.62% to 149.9%) | 2.18% (-36.84% to 71.47%) |
|  |  | YLLs | All age number | 172.81 (113.46 to 255.6) | 29.01 (15.85 to 47.09) | 143.8 (91.85 to 220.77) | 478.17 (318.2 to 685.5) | 132.5 (72.65 to 212.94) | 345.66 (229.01 to 489.31) | 176.7% (76.6% to 341.03%) | 356.7% (146.59% to 727.69%) | 140.38% (46.48% to 295.79%) |
|  |  |  | Age-standardized rate (per 100,000) | 96.11 (63.61 to 141.58) | 35.94 (19.52 to 57.84) | 142.28 (91.33 to 217.38) | 87.6 (58.76 to 125.05) | 45.9 (25.05 to 73.61) | 132.61 (88.8 to 187.82) | -8.86% (-42.0% to 43.12%) | 27.73% (-30.43% to 139.0%) | -6.79% (-42.58% to 52.56%) |
|  | Metabolic risks | DALYs | All age number | 89.78 (38.99 to 144.3) | 43.54 (20.3 to 70.88) | 46.24 (17.58 to 79.48) | 759.04 (271.52 to 1252.26) | 397.77 (136.84 to 668.62) | 361.27 (137.57 to 598.64) | 745.45% (516.85% to 991.99%) | 813.65% (455.68% to 1211.57%) | 681.25% (434.08% to 1037.43%) |
|  |  |  | Age-standardized rate (per 100,000) | 47.69 (19.04 to 77.21) | 52.47 (22.42 to 87.41) | 44.09 (15.39 to 75.53) | 138.41 (47.41 to 231.29) | 140.91 (46.08 to 239.28) | 135.57 (49.36 to 228.51) | 190.2% (116.68% to 264.91%) | 168.58% (70.43% to 273.24%) | 207.46% (112.03% to 337.62%) |
|  |  | Deaths | All age number | 3.0 (1.17 to 4.91) | 1.4 (0.6 to 2.32) | 1.59 (0.54 to 2.73) | 27.82 (9.22 to 46.5) | 14.43 (4.66 to 24.54) | 13.39 (4.7 to 22.83) | 828.29% (588.5% to 1057.68%) | 928.34% (554.09% to 1331.33%) | 740.16% (484.07% to 1090.93%) |
|  |  |  | Age-standardized rate (per 100,000) | 1.86 (0.67 to 3.04) | 1.98 (0.77 to 3.27) | 1.78 (0.56 to 3.07) | 5.76 (1.84 to 9.64) | 5.67 (1.78 to 9.77) | 5.85 (1.94 to 10.09) | 209.77% (138.06% to 280.13%) | 186.54% (91.99% to 288.08%) | 229.61% (131.89% to 353.92%) |
|  |  | YLDs | All age number | 1.75 (0.61 to 3.03) | 0.99 (0.34 to 1.77) | 0.76 (0.29 to 1.39) | 25.96 (9.14 to 44.68) | 16.94 (5.04 to 30.95) | 9.02 (3.82 to 15.46) | 1386.48% (1003.38% to 1845.46%) | 1620.03% (990.29% to 2396.89%) | 1084.32% (713.75% to 1665.7%) |
|  |  |  | Age-standardized rate (per 100,000) | 1.0 (0.32 to 1.75) | 1.31 (0.39 to 2.39) | 0.78 (0.28 to 1.42) | 4.8 (1.59 to 8.36) | 6.07 (1.68 to 11.03) | 3.45 (1.41 to 5.96) | 379.92% (267.59% to 518.59%) | 362.02% (201.41% to 568.38%) | 344.02% (210.72% to 542.11%) |
|  |  | YLLs | All age number | 88.03 (38.43 to 141.05) | 42.55 (19.9 to 68.92) | 45.48 (17.32 to 78.14) | 733.08 (263.24 to 1202.07) | 380.82 (131.25 to 640.91) | 352.25 (134.2 to 585.12) | 732.74% (504.24% to 977.42%) | 794.98% (442.86% to 1182.65%) | 674.5% (428.63% to 1029.8%) |
|  |  |  | Age-standardized rate (per 100,000) | 46.69 (18.68 to 75.49) | 51.15 (22.06 to 84.63) | 43.31 (15.14 to 74.15) | 133.6 (45.95 to 222.72) | 134.84 (44.26 to 228.37) | 132.12 (48.25 to 223.69) | 186.13% (113.12% to 259.45%) | 163.61% (68.13% to 266.75%) | 205.01% (109.97% to 335.06%) |
| Isfahan | All risk factors | DALYs | All age number | 8486.32 (6349.56 to 11489.57) | 3108.6 (1984.11 to 4494.16) | 5377.72 (3704.39 to 7539.51) | 27436.39 (20579.96 to 35028.42) | 10750.26 (6668.3 to 15156.22) | 16686.13 (12197.87 to 21949.29) | 223.3% (147.19% to 333.21%) | 245.82% (137.16% to 402.7%) | 210.28% (120.3% to 360.84%) |
|  |  |  | Age-standardized rate (per 100,000) | 423.94 (315.83 to 568.88) | 312.08 (200.93 to 447.16) | 535.95 (373.59 to 743.14) | 474.86 (357.41 to 604.82) | 375.2 (234.19 to 531.69) | 572.97 (417.91 to 749.08) | 12.01% (-14.77% to 48.43%) | 20.23% (-16.08% to 72.62%) | 6.91% (-24.55% to 56.53%) |
|  |  | Deaths | All age number | 290.53 (216.37 to 388.63) | 100.43 (63.99 to 144.84) | 190.1 (131.39 to 266.78) | 1040.67 (787.13 to 1322.07) | 393.97 (249.61 to 557.73) | 646.7 (476.57 to 839.84) | 258.2% (167.24% to 380.93%) | 292.3% (177.38% to 456.55%) | 240.18% (140.12% to 403.01%) |
|  |  |  | Age-standardized rate (per 100,000) | 16.98 (12.68 to 22.65) | 11.98 (7.61 to 17.2) | 22.28 (15.72 to 31.33) | 19.67 (14.9 to 24.87) | 15.28 (9.7 to 21.5) | 24.0 (17.8 to 31.02) | 15.87% (-11.72% to 52.63%) | 27.59% (-7.85% to 78.54%) | 7.73% (-23.75% to 55.92%) |
|  |  | YLDs | All age number | 158.94 (98.03 to 238.35) | 80.2 (39.64 to 134.02) | 78.74 (49.87 to 121.57) | 867.58 (502.07 to 1354.04) | 515.19 (231.05 to 887.03) | 352.39 (237.83 to 496.58) | 445.86% (300.93% to 626.7%) | 542.4% (346.02% to 823.74%) | 347.53% (213.83% to 546.18%) |
|  |  |  | Age-standardized rate (per 100,000) | 8.08 (5.06 to 12.06) | 8.02 (3.97 to 13.23) | 8.26 (5.28 to 12.53) | 14.8 (8.63 to 22.85) | 17.37 (7.73 to 29.83) | 12.32 (8.34 to 17.36) | 83.12% (34.92% to 141.59%) | 116.49% (50.24% to 206.42%) | 49.22% (4.63% to 114.62%) |
|  |  | YLLs | All age number | 8327.38 (6228.97 to 11275.48) | 3028.4 (1923.09 to 4396.35) | 5298.98 (3645.35 to 7422.29) | 26568.81 (19857.32 to 33861.65) | 10235.07 (6399.17 to 14232.43) | 16333.74 (11952.11 to 21541.41) | 219.05% (143.0% to 328.04%) | 237.97% (133.48% to 392.69%) | 208.24% (118.82% to 357.16%) |
|  |  |  | Age-standardized rate (per 100,000) | 415.86 (308.96 to 558.08) | 304.06 (194.73 to 437.41) | 527.7 (367.41 to 731.96) | 460.06 (347.63 to 585.27) | 357.83 (225.18 to 499.29) | 560.65 (409.27 to 734.19) | 10.63% (-15.99% to 46.96%) | 17.69% (-17.66% to 68.59%) | 6.24% (-25.01% to 55.62%) |
|  | Behavioral risks | DALYs | All age number | 6981.73 (5008.47 to 9762.15) | 2318.04 (1384.23 to 3463.33) | 4663.69 (3191.9 to 6685.63) | 19440.31 (14304.5 to 25488.14) | 6268.96 (3524.32 to 9416.66) | 13171.35 (9804.89 to 17525.58) | 178.45% (107.18% to 272.62%) | 170.44% (86.9% to 295.9%) | 182.42% (102.9% to 317.33%) |
|  |  |  | Age-standardized rate (per 100,000) | 349.51 (251.93 to 486.69) | 231.01 (138.18 to 340.23) | 467.92 (323.91 to 668.47) | 333.89 (245.15 to 439.3) | 213.41 (122.45 to 316.87) | 451.8 (337.48 to 604.0) | -4.47% (-28.02% to 27.55%) | -7.62% (-35.74% to 33.45%) | -3.45% (-31.6% to 41.06%) |
|  |  | Deaths | All age number | 239.92 (173.29 to 335.03) | 73.39 (44.58 to 107.2) | 166.53 (113.4 to 238.65) | 730.78 (550.2 to 961.13) | 218.3 (130.71 to 316.72) | 512.49 (381.77 to 682.95) | 204.6% (129.91% to 307.62%) | 197.46% (109.22% to 322.84%) | 207.75% (119.22% to 353.42%) |
|  |  |  | Age-standardized rate (per 100,000) | 13.93 (10.16 to 19.45) | 8.68 (5.29 to 12.74) | 19.5 (13.67 to 27.79) | 13.73 (10.41 to 18.1) | 8.32 (5.05 to 12.0) | 19.0 (14.1 to 25.03) | -1.5% (-24.55% to 29.12%) | -4.16% (-31.22% to 34.13%) | -2.59% (-30.74% to 41.0%) |
|  |  | YLDs | All age number | 132.34 (79.18 to 205.62) | 62.53 (28.03 to 109.92) | 69.81 (43.73 to 109.0) | 604.23 (330.59 to 964.99) | 324.74 (112.36 to 616.99) | 279.49 (187.44 to 398.38) | 356.56% (236.09% to 523.62%) | 419.33% (215.21% to 657.76%) | 300.35% (176.26% to 472.99%) |
|  |  |  | Age-standardized rate (per 100,000) | 6.68 (4.05 to 10.19) | 6.12 (2.77 to 10.61) | 7.33 (4.67 to 11.26) | 10.17 (5.71 to 15.94) | 10.59 (3.75 to 20.05) | 9.78 (6.59 to 13.84) | 52.34% (13.19% to 104.96%) | 72.93% (8.45% to 148.12%) | 33.49% (-6.98% to 89.23%) |
|  |  | YLLs | All age number | 6849.39 (4919.45 to 9567.08) | 2255.51 (1351.72 to 3356.48) | 4593.88 (3137.24 to 6597.03) | 18836.09 (13899.07 to 24651.88) | 5944.23 (3433.63 to 8866.37) | 12891.86 (9583.68 to 17217.95) | 175.0% (105.6% to 268.82%) | 163.54% (81.85% to 284.74%) | 180.63% (101.4% to 314.55%) |
|  |  |  | Age-standardized rate (per 100,000) | 342.84 (247.21 to 478.1) | 224.89 (135.49 to 330.6) | 460.59 (317.51 to 657.61) | 323.72 (240.14 to 425.21) | 202.82 (119.36 to 297.74) | 442.01 (329.78 to 592.62) | -5.58% (-28.55% to 26.49%) | -9.81% (-37.08% to 30.43%) | -4.03% (-32.1% to 40.27%) |
|  | Environmental/occupational risks | DALYs | All age number | 1385.11 (898.5 to 2150.67) | 333.19 (181.32 to 525.19) | 1051.92 (645.53 to 1699.48) | 4340.01 (2745.99 to 6283.46) | 1266.7 (744.71 to 1957.97) | 3073.31 (1952.39 to 4540.48) | 213.33% (115.56% to 381.0%) | 280.17% (128.41% to 543.11%) | 192.16% (90.3% to 380.36%) |
|  |  |  | Age-standardized rate (per 100,000) | 68.73 (44.35 to 106.82) | 34.84 (19.4 to 54.37) | 101.69 (62.13 to 162.81) | 75.02 (47.28 to 108.67) | 45.37 (26.82 to 70.06) | 104.04 (66.06 to 152.66) | 9.15% (-24.37% to 67.24%) | 30.24% (-20.85% to 119.72%) | 2.31% (-33.2% to 68.75%) |
|  |  | Deaths | All age number | 47.97 (30.65 to 73.92) | 11.72 (6.5 to 18.29) | 36.25 (21.95 to 58.47) | 166.47 (104.92 to 238.7) | 50.99 (30.46 to 78.6) | 115.48 (72.47 to 169.07) | 247.04% (138.23% to 430.0%) | 335.12% (163.22% to 628.94%) | 218.57% (106.14% to 425.26%) |
|  |  |  | Age-standardized rate (per 100,000) | 2.77 (1.75 to 4.32) | 1.47 (0.81 to 2.28) | 4.11 (2.46 to 6.48) | 3.13 (1.97 to 4.51) | 2.03 (1.21 to 3.15) | 4.21 (2.62 to 6.18) | 12.96% (-21.55% to 70.52%) | 37.91% (-15.71% to 132.57%) | 2.57% (-32.98% to 67.96%) |
|  |  | YLDs | All age number | 11.85 (6.38 to 18.99) | 2.82 (1.39 to 4.65) | 9.03 (4.68 to 14.91) | 40.13 (23.13 to 60.51) | 11.52 (5.9 to 19.69) | 28.6 (16.49 to 43.22) | 238.66% (121.07% to 437.85%) | 309.0% (145.3% to 613.33%) | 216.72% (92.02% to 461.76%) |
|  |  |  | Age-standardized rate (per 100,000) | 0.63 (0.33 to 1.0) | 0.32 (0.16 to 0.53) | 0.93 (0.48 to 1.52) | 0.72 (0.41 to 1.07) | 0.43 (0.22 to 0.74) | 1.0 (0.56 to 1.51) | 14.51% (-24.18% to 77.58%) | 35.19% (-19.33% to 136.82%) | 7.06% (-35.3% to 82.01%) |
|  |  | YLLs | All age number | 1373.27 (889.69 to 2135.25) | 330.38 (179.52 to 520.86) | 1042.89 (641.11 to 1685.16) | 4299.88 (2720.64 to 6219.1) | 1255.18 (739.09 to 1938.53) | 3044.7 (1932.55 to 4499.32) | 213.11% (115.52% to 380.73%) | 279.92% (128.38% to 542.32%) | 191.95% (90.09% to 379.9%) |
|  |  |  | Age-standardized rate (per 100,000) | 68.11 (43.9 to 105.92) | 34.52 (19.21 to 53.84) | 100.76 (61.59 to 161.45) | 74.31 (46.8 to 107.53) | 44.94 (26.61 to 69.37) | 103.05 (65.38 to 151.24) | 9.1% (-24.53% to 67.16%) | 30.19% (-20.94% to 119.63%) | 2.27% (-33.19% to 68.75%) |
|  | Metabolic risks | DALYs | All age number | 1133.13 (432.39 to 1850.16) | 635.14 (231.92 to 1123.25) | 497.99 (197.1 to 831.77) | 7584.97 (2260.31 to 12717.77) | 4164.51 (1163.97 to 7160.55) | 3420.45 (1084.64 to 5681.84) | 569.38% (367.39% to 770.48%) | 555.68% (321.7% to 842.24%) | 586.85% (354.42% to 903.73%) |
|  |  |  | Age-standardized rate (per 100,000) | 56.44 (19.48 to 93.72) | 65.17 (22.76 to 115.88) | 47.99 (17.91 to 81.48) | 134.06 (38.81 to 225.56) | 150.55 (41.17 to 260.13) | 118.45 (36.62 to 198.56) | 137.53% (71.71% to 205.63%) | 131.02% (54.75% to 226.54%) | 146.81% (66.13% to 255.39%) |
|  |  | Deaths | All age number | 38.47 (12.97 to 64.1) | 21.72 (7.34 to 38.78) | 16.76 (6.11 to 28.75) | 295.02 (81.73 to 500.75) | 162.46 (43.14 to 282.49) | 132.56 (38.46 to 225.01) | 666.8% (453.42% to 870.09%) | 648.14% (405.94% to 941.92%) | 690.97% (432.69% to 1036.14%) |
|  |  |  | Age-standardized rate (per 100,000) | 2.29 (0.73 to 3.86) | 2.62 (0.85 to 4.63) | 1.95 (0.64 to 3.35) | 5.66 (1.55 to 9.64) | 6.43 (1.7 to 11.21) | 4.96 (1.41 to 8.42) | 146.99% (85.47% to 210.92%) | 145.52% (70.56% to 239.11%) | 153.85% (72.38% to 258.84%) |
|  |  | YLDs | All age number | 27.47 (7.48 to 50.4) | 18.77 (4.51 to 35.55) | 8.69 (3.03 to 15.99) | 304.68 (86.51 to 543.78) | 216.74 (49.29 to 393.49) | 87.94 (31.75 to 155.59) | 1009.28% (726.17% to 1398.1%) | 1054.5% (663.64% to 1579.82%) | 911.62% (596.04% to 1401.79%) |
|  |  |  | Age-standardized rate (per 100,000) | 1.45 (0.38 to 2.67) | 2.01 (0.46 to 3.81) | 0.91 (0.31 to 1.65) | 5.36 (1.46 to 9.6) | 7.72 (1.7 to 14.02) | 3.08 (1.09 to 5.49) | 269.81% (179.04% to 395.45%) | 283.05% (162.38% to 456.61%) | 238.65% (134.79% to 386.48%) |
|  |  | YLLs | All age number | 1105.66 (427.06 to 1803.02) | 616.37 (226.53 to 1083.67) | 489.3 (193.74 to 815.2) | 7280.28 (2163.41 to 12226.71) | 3947.77 (1117.3 to 6806.29) | 3332.51 (1047.23 to 5529.79) | 558.45% (357.72% to 754.13%) | 540.49% (311.93% to 820.42%) | 581.08% (349.46% to 895.18%) |
|  |  |  | Age-standardized rate (per 100,000) | 54.99 (19.14 to 91.19) | 63.15 (22.21 to 112.25) | 47.08 (17.56 to 79.89) | 128.69 (37.19 to 216.45) | 142.84 (39.53 to 246.89) | 115.37 (35.52 to 193.08) | 134.04% (68.52% to 200.83%) | 126.17% (51.21% to 219.41%) | 145.03% (64.76% to 253.15%) |
| Kerman | All risk factors | DALYs | All age number | 5725.63 (4384.19 to 7627.7) | 1558.13 (988.98 to 2189.29) | 4167.5 (3086.77 to 5712.76) | 15131.29 (11541.77 to 19214.55) | 5054.76 (3317.01 to 6831.82) | 10076.53 (7723.49 to 12974.83) | 164.27% (99.38% to 258.91%) | 224.41% (118.5% to 368.41%) | 141.79% (64.55% to 261.32%) |
|  |  |  | Age-standardized rate (per 100,000) | 609.37 (471.88 to 808.73) | 338.74 (215.48 to 473.33) | 859.66 (638.15 to 1159.75) | 530.97 (404.79 to 672.11) | 349.98 (229.42 to 469.63) | 714.44 (552.27 to 915.49) | -12.87% (-34.17% to 17.33%) | 3.32% (-29.84% to 47.87%) | -16.89% (-42.99% to 21.91%) |
|  |  | Deaths | All age number | 196.43 (151.16 to 260.74) | 48.92 (31.08 to 68.29) | 147.51 (109.88 to 199.61) | 546.07 (416.13 to 689.96) | 176.06 (115.57 to 236.3) | 370.02 (286.95 to 474.62) | 178.0% (109.84% to 278.3%) | 259.89% (145.2% to 414.04%) | 150.84% (73.45% to 265.77%) |
|  |  |  | Age-standardized rate (per 100,000) | 23.74 (18.58 to 31.59) | 12.49 (8.1 to 17.28) | 34.78 (26.09 to 46.34) | 21.91 (16.62 to 27.58) | 14.11 (9.23 to 18.93) | 29.87 (23.11 to 37.9) | -7.71% (-29.69% to 23.53%) | 12.99% (-21.13% to 60.03%) | -14.1% (-39.58% to 22.15%) |
|  |  | YLDs | All age number | 84.02 (56.45 to 119.37) | 31.01 (16.03 to 48.89) | 53.01 (35.2 to 77.05) | 376.11 (226.64 to 567.43) | 194.14 (94.56 to 315.57) | 181.97 (118.55 to 264.01) | 347.62% (225.74% to 483.98%) | 525.98% (313.2% to 798.0%) | 243.27% (135.96% to 392.83%) |
|  |  |  | Age-standardized rate (per 100,000) | 9.17 (6.21 to 12.91) | 6.83 (3.57 to 10.67) | 11.35 (7.58 to 16.31) | 13.05 (7.98 to 19.49) | 12.96 (6.43 to 20.63) | 13.29 (8.68 to 19.4) | 42.43% (5.25% to 84.45%) | 89.87% (27.19% to 163.74%) | 17.09% (-18.5% to 66.12%) |
|  |  | YLLs | All age number | 5641.61 (4316.18 to 7512.72) | 1527.12 (967.11 to 2144.63) | 4114.49 (3044.46 to 5648.6) | 14755.18 (11339.85 to 18701.49) | 4860.62 (3218.94 to 6605.4) | 9894.56 (7581.58 to 12715.4) | 161.54% (97.2% to 255.46%) | 218.29% (113.73% to 359.43%) | 140.48% (63.4% to 259.25%) |
|  |  |  | Age-standardized rate (per 100,000) | 600.21 (464.41 to 796.77) | 331.92 (210.18 to 464.28) | 848.31 (629.38 to 1146.8) | 517.91 (396.7 to 656.27) | 337.02 (222.63 to 454.18) | 701.15 (541.51 to 899.26) | -13.71% (-34.68% to 16.11%) | 1.54% (-30.97% to 45.54%) | -17.35% (-43.35% to 21.42%) |
|  | Behavioral risks | DALYs | All age number | 4780.78 (3546.38 to 6537.09) | 1132.48 (721.11 to 1680.4) | 3648.3 (2612.34 to 5107.9) | 11221.19 (8577.98 to 14254.56) | 2989.48 (1894.41 to 4525.18) | 8231.71 (6220.41 to 10714.1) | 134.71% (76.48% to 224.76%) | 163.98% (81.19% to 285.45%) | 125.63% (51.18% to 231.17%) |
|  |  |  | Age-standardized rate (per 100,000) | 508.26 (379.54 to 693.53) | 243.92 (158.05 to 359.21) | 752.36 (548.49 to 1042.5) | 391.63 (302.12 to 500.23) | 200.34 (128.22 to 301.17) | 585.64 (444.62 to 766.05) | -22.95% (-41.39% to 5.85%) | -17.87% (-42.17% to 18.55%) | -22.16% (-46.81% to 12.45%) |
|  |  | Deaths | All age number | 164.53 (122.44 to 224.94) | 34.81 (22.62 to 52.01) | 129.72 (94.46 to 180.87) | 402.25 (310.04 to 513.11) | 98.88 (63.72 to 146.01) | 303.38 (228.8 to 400.59) | 144.48% (84.94% to 238.01%) | 184.03% (99.6% to 308.42%) | 133.87% (60.77% to 240.64%) |
|  |  |  | Age-standardized rate (per 100,000) | 19.67 (14.82 to 26.79) | 8.76 (5.66 to 13.09) | 30.35 (22.32 to 42.14) | 15.98 (12.33 to 20.57) | 7.71 (4.98 to 11.33) | 24.44 (18.45 to 32.2) | -18.73% (-38.34% to 10.39%) | -11.9% (-37.29% to 24.78%) | -19.49% (-42.77% to 12.81%) |
|  |  | YLDs | All age number | 71.03 (46.09 to 103.3) | 23.69 (11.76 to 39.6) | 47.34 (30.57 to 69.89) | 272.67 (159.31 to 427.2) | 123.5 (47.77 to 227.2) | 149.17 (95.27 to 224.95) | 283.88% (182.47% to 408.95%) | 421.27% (213.63% to 679.91%) | 215.11% (115.64% to 347.72%) |
|  |  |  | Age-standardized rate (per 100,000) | 7.7 (5.06 to 11.09) | 5.1 (2.54 to 8.44) | 10.11 (6.59 to 14.84) | 9.29 (5.61 to 14.23) | 7.81 (3.13 to 13.93) | 10.93 (7.11 to 16.4) | 20.71% (-9.66% to 57.98%) | 52.97% (-5.74% to 120.91%) | 8.15% (-24.99% to 53.23%) |
|  |  | YLLs | All age number | 4709.75 (3494.15 to 6446.14) | 1108.79 (708.32 to 1649.09) | 3600.96 (2580.58 to 5048.74) | 10948.52 (8408.85 to 13894.01) | 2865.98 (1832.49 to 4339.51) | 8082.54 (6102.53 to 10533.73) | 132.46% (75.02% to 220.78%) | 158.48% (77.47% to 276.74%) | 124.46% (50.37% to 229.62%) |
|  |  |  | Age-standardized rate (per 100,000) | 500.56 (374.57 to 683.43) | 238.82 (155.11 to 352.9) | 742.25 (540.99 to 1031.07) | 382.34 (295.34 to 488.9) | 192.53 (124.28 to 287.0) | 574.71 (435.89 to 752.85) | -23.62% (-41.89% to 4.99%) | -19.38% (-43.13% to 15.92%) | -22.57% (-47.07% to 11.84%) |
|  | Environmental/occupational risks | DALYs | All age number | 1232.09 (762.19 to 1833.29) | 233.6 (121.0 to 394.29) | 998.49 (598.82 to 1517.19) | 2988.72 (1910.32 to 4378.67) | 764.23 (417.82 to 1176.04) | 2224.49 (1414.32 to 3252.42) | 142.57% (60.05% to 281.84%) | 227.16% (69.74% to 510.11%) | 122.78% (38.46% to 256.34%) |
|  |  |  | Age-standardized rate (per 100,000) | 131.66 (80.69 to 195.3) | 53.58 (27.81 to 90.5) | 203.84 (123.1 to 309.2) | 104.79 (66.71 to 153.84) | 54.72 (29.58 to 84.11) | 154.86 (96.64 to 227.42) | -20.41% (-47.27% to 23.4%) | 2.12% (-46.33% to 89.84%) | -24.03% (-52.49% to 20.85%) |
|  |  | Deaths | All age number | 43.08 (26.54 to 63.68) | 8.03 (4.15 to 13.47) | 35.06 (20.91 to 52.89) | 109.2 (69.44 to 160.44) | 29.14 (15.7 to 44.87) | 80.06 (49.85 to 116.49) | 153.45% (68.26% to 292.43%) | 263.06% (90.7% to 570.38%) | 128.36% (42.68% to 265.68%) |
|  |  |  | Age-standardized rate (per 100,000) | 5.24 (3.28 to 7.74) | 2.21 (1.12 to 3.66) | 8.19 (5.02 to 12.4) | 4.37 (2.76 to 6.41) | 2.43 (1.3 to 3.77) | 6.34 (3.93 to 9.3) | -16.66% (-44.08% to 25.98%) | 9.96% (-40.0% to 104.27%) | -22.66% (-51.28% to 20.75%) |
|  |  | YLDs | All age number | 10.19 (5.56 to 16.59) | 1.88 (0.87 to 3.38) | 8.31 (4.42 to 14.02) | 26.06 (14.49 to 39.65) | 6.52 (3.25 to 10.59) | 19.53 (10.61 to 30.83) | 155.68% (61.38% to 302.48%) | 246.33% (78.2% to 570.61%) | 135.14% (40.47% to 282.17%) |
|  |  |  | Age-standardized rate (per 100,000) | 1.14 (0.63 to 1.84) | 0.47 (0.22 to 0.84) | 1.78 (0.95 to 2.92) | 0.96 (0.54 to 1.47) | 0.5 (0.24 to 0.8) | 1.42 (0.78 to 2.23) | -16.1% (-46.3% to 29.53%) | 6.23% (-45.09% to 107.28%) | -19.84% (-52.0% to 29.15%) |
|  |  | YLLs | All age number | 1221.9 (756.45 to 1818.57) | 231.72 (120.06 to 391.03) | 990.18 (595.26 to 1504.98) | 2962.66 (1896.73 to 4343.92) | 757.71 (414.69 to 1167.13) | 2204.95 (1403.08 to 3223.36) | 142.46% (60.14% to 281.73%) | 227.0% (69.6% to 509.04%) | 122.68% (38.38% to 256.2%) |
|  |  |  | Age-standardized rate (per 100,000) | 130.52 (79.99 to 193.66) | 53.11 (27.57 to 89.68) | 202.06 (122.2 to 306.91) | 103.83 (66.11 to 152.55) | 54.22 (29.35 to 83.43) | 153.44 (95.88 to 225.2) | -20.45% (-47.29% to 23.38%) | 2.08% (-46.35% to 89.8%) | -24.06% (-52.52% to 20.85%) |
|  | Metabolic risks | DALYs | All age number | 548.09 (214.2 to 860.75) | 285.26 (116.37 to 461.65) | 262.83 (93.05 to 452.23) | 3239.43 (1064.47 to 5504.66) | 1727.48 (571.67 to 2880.28) | 1511.94 (518.78 to 2666.01) | 491.04% (316.9% to 673.35%) | 505.59% (267.08% to 762.74%) | 475.24% (281.38% to 759.03%) |
|  |  |  | Age-standardized rate (per 100,000) | 57.93 (21.77 to 91.89) | 62.64 (23.44 to 101.93) | 53.66 (18.0 to 92.75) | 116.87 (36.2 to 202.59) | 126.22 (39.53 to 215.18) | 107.43 (35.05 to 192.73) | 101.73% (43.99% to 159.31%) | 101.5% (26.59% to 181.59%) | 100.21% (32.53% to 195.42%) |
|  |  | Deaths | All age number | 18.29 (6.67 to 29.34) | 9.31 (3.4 to 15.24) | 8.98 (2.88 to 15.56) | 120.02 (36.1 to 209.08) | 64.44 (19.38 to 109.67) | 55.59 (17.49 to 100.52) | 556.18% (365.53% to 739.76%) | 592.06% (334.76% to 859.78%) | 518.98% (311.69% to 808.8%) |
|  |  |  | Age-standardized rate (per 100,000) | 2.26 (0.77 to 3.72) | 2.38 (0.82 to 3.84) | 2.16 (0.64 to 3.79) | 4.95 (1.42 to 8.62) | 5.33 (1.54 to 9.04) | 4.57 (1.4 to 8.45) | 118.92% (60.24% to 172.82%) | 124.29% (46.7% to 207.82%) | 111.81% (45.9% to 204.66%) |
|  |  | YLDs | All age number | 11.11 (3.43 to 19.31) | 6.94 (2.07 to 12.42) | 4.16 (1.44 to 7.58) | 112.23 (34.1 to 203.77) | 76.52 (20.88 to 139.09) | 35.71 (12.41 to 64.32) | 910.48% (627.13% to 1254.9%) | 1002.23% (622.16% to 1544.99%) | 757.52% (477.96% to 1231.92%) |
|  |  |  | Age-standardized rate (per 100,000) | 1.24 (0.36 to 2.19) | 1.62 (0.45 to 2.93) | 0.9 (0.32 to 1.6) | 4.1 (1.17 to 7.46) | 5.6 (1.46 to 10.29) | 2.6 (0.85 to 4.75) | 231.39% (140.66% to 332.84%) | 246.34% (134.9% to 398.42%) | 189.24% (98.81% to 336.03%) |
|  |  | YLLs | All age number | 536.99 (210.17 to 844.09) | 278.31 (114.54 to 450.43) | 258.67 (91.5 to 444.18) | 3127.19 (1030.91 to 5317.73) | 1650.96 (546.19 to 2747.13) | 1476.23 (505.28 to 2593.22) | 482.36% (311.48% to 661.19%) | 493.2% (258.72% to 744.27%) | 470.7% (278.17% to 750.75%) |
|  |  |  | Age-standardized rate (per 100,000) | 56.69 (21.34 to 89.79) | 61.02 (23.07 to 99.47) | 52.76 (17.67 to 91.39) | 112.76 (35.07 to 195.44) | 120.62 (37.78 to 205.08) | 104.83 (34.14 to 187.58) | 98.9% (42.42% to 155.88%) | 97.67% (24.08% to 176.65%) | 98.69% (31.39% to 193.26%) |
| Kermanshah | All risk factors | DALYs | All age number | 5693.76 (4292.58 to 7649.24) | 1537.11 (991.41 to 2278.08) | 4156.65 (2985.5 to 5736.62) | 11678.14 (8713.39 to 14619.06) | 4130.95 (2612.44 to 5857.01) | 7547.19 (5618.95 to 9552.22) | 105.1% (55.21% to 174.2%) | 168.75% (79.58% to 308.07%) | 81.57% (28.05% to 159.24%) |
|  |  |  | Age-standardized rate (per 100,000) | 690.22 (513.0 to 928.51) | 388.01 (250.41 to 569.84) | 931.06 (668.62 to 1281.23) | 547.4 (409.49 to 686.14) | 376.85 (238.98 to 530.81) | 730.04 (542.67 to 925.89) | -20.69% (-39.6% to 6.09%) | -2.88% (-34.51% to 44.24%) | -21.59% (-44.38% to 11.02%) |
|  |  | Deaths | All age number | 193.66 (143.56 to 260.97) | 46.84 (30.23 to 68.7) | 146.82 (105.14 to 202.34) | 434.74 (323.97 to 546.7) | 148.52 (95.72 to 207.35) | 286.22 (214.25 to 362.99) | 124.48% (69.54% to 199.27%) | 217.06% (113.1% to 371.47%) | 94.94% (39.14% to 175.67%) |
|  |  |  | Age-standardized rate (per 100,000) | 27.12 (20.23 to 36.27) | 14.38 (9.2 to 20.85) | 37.55 (27.16 to 51.62) | 22.15 (16.51 to 27.86) | 14.94 (9.64 to 20.78) | 29.85 (22.47 to 37.96) | -18.33% (-37.47% to 8.14%) | 3.85% (-29.52% to 50.82%) | -20.51% (-42.71% to 9.88%) |
|  |  | YLDs | All age number | 82.06 (54.5 to 118.73) | 29.72 (15.26 to 47.36) | 52.34 (33.74 to 75.78) | 300.43 (184.78 to 455.68) | 159.23 (76.47 to 267.95) | 141.2 (91.58 to 205.09) | 266.11% (164.6% to 392.81%) | 435.77% (256.92% to 690.9%) | 169.77% (95.76% to 282.69%) |
|  |  |  | Age-standardized rate (per 100,000) | 10.19 (6.72 to 14.5) | 7.56 (3.89 to 11.75) | 12.18 (8.0 to 17.56) | 13.96 (8.65 to 20.89) | 14.14 (6.78 to 23.67) | 13.9 (9.08 to 19.99) | 37.09% (0.05% to 83.12%) | 87.08% (26.41% to 169.94%) | 14.13% (-16.31% to 61.0%) |
|  |  | YLLs | All age number | 5611.7 (4224.12 to 7546.89) | 1507.39 (977.55 to 2237.21) | 4104.31 (2946.21 to 5670.34) | 11377.72 (8539.9 to 14274.88) | 3971.73 (2549.23 to 5587.51) | 7405.99 (5513.39 to 9358.22) | 102.75% (53.26% to 171.14%) | 163.48% (75.58% to 300.04%) | 80.44% (27.04% to 157.91%) |
|  |  |  | Age-standardized rate (per 100,000) | 680.04 (505.53 to 914.57) | 380.45 (245.74 to 559.22) | 918.88 (659.26 to 1265.47) | 533.43 (398.61 to 668.28) | 362.72 (233.05 to 508.51) | 716.14 (533.81 to 906.76) | -21.56% (-40.33% to 5.14%) | -4.66% (-35.86% to 41.89%) | -22.06% (-44.79% to 10.5%) |
|  | Behavioral risks | DALYs | All age number | 4900.8 (3537.77 to 6782.22) | 1192.82 (706.98 to 1837.45) | 3707.99 (2575.67 to 5190.24) | 8575.79 (6397.36 to 11278.66) | 2474.36 (1442.09 to 3656.11) | 6101.43 (4555.69 to 7903.72) | 74.99% (31.84% to 132.01%) | 107.44% (42.25% to 202.48%) | 64.55% (15.38% to 135.68%) |
|  |  |  | Age-standardized rate (per 100,000) | 595.67 (431.24 to 821.56) | 298.76 (176.92 to 461.86) | 832.16 (583.39 to 1163.51) | 400.33 (299.7 to 522.55) | 221.83 (130.36 to 324.52) | 591.38 (441.74 to 762.72) | -32.79% (-49.1% to -11.08%) | -25.75% (-48.19% to 6.24%) | -28.93% (-50.03% to 1.49%) |
|  |  | Deaths | All age number | 167.54 (120.87 to 232.09) | 35.9 (21.23 to 55.54) | 131.64 (91.74 to 185.15) | 317.97 (241.36 to 410.1) | 85.09 (51.21 to 123.05) | 232.87 (173.41 to 300.7) | 89.78% (42.13% to 152.34%) | 137.0% (66.52% to 236.85%) | 76.91% (24.76% to 149.94%) |
|  |  |  | Age-standardized rate (per 100,000) | 23.37 (16.89 to 32.36) | 10.91 (6.37 to 16.91) | 33.55 (23.65 to 46.94) | 16.13 (12.33 to 20.87) | 8.44 (5.08 to 12.15) | 24.29 (18.14 to 31.42) | -30.95% (-47.51% to -10.01%) | -22.63% (-44.5% to 9.12%) | -27.59% (-48.23% to 1.04%) |
|  |  | YLDs | All age number | 71.51 (45.6 to 104.91) | 24.05 (11.48 to 40.45) | 47.46 (30.05 to 69.73) | 217.34 (131.26 to 343.71) | 103.06 (39.48 to 186.59) | 114.28 (74.01 to 167.39) | 203.93% (116.0% to 311.75%) | 328.53% (176.0% to 525.21%) | 140.79% (74.95% to 238.79%) |
|  |  |  | Age-standardized rate (per 100,000) | 8.84 (5.67 to 12.68) | 5.96 (2.86 to 9.86) | 11.03 (7.22 to 15.96) | 10.01 (6.23 to 15.6) | 8.92 (3.5 to 15.99) | 11.29 (7.32 to 16.64) | 13.23% (-18.53% to 50.72%) | 49.78% (-1.91% to 114.07%) | 2.34% (-24.95% to 43.04%) |
|  |  | YLLs | All age number | 4829.29 (3489.02 to 6689.36) | 1168.77 (694.47 to 1802.19) | 3660.53 (2543.72 to 5132.59) | 8358.45 (6223.59 to 10949.12) | 2371.3 (1398.23 to 3476.8) | 5987.15 (4474.27 to 7749.69) | 73.08% (30.42% to 129.65%) | 102.89% (39.17% to 195.88%) | 63.56% (14.55% to 134.68%) |
|  |  |  | Age-standardized rate (per 100,000) | 586.83 (423.99 to 808.67) | 292.8 (173.8 to 453.16) | 821.13 (576.55 to 1149.0) | 390.32 (292.03 to 507.6) | 212.91 (126.31 to 309.73) | 580.09 (433.9 to 746.97) | -33.49% (-49.63% to -11.68%) | -27.29% (-49.17% to 3.73%) | -29.35% (-50.27% to 0.99%) |
|  | Environmental/occupational risks | DALYs | All age number | 921.68 (553.76 to 1441.67) | 151.62 (78.68 to 254.63) | 770.06 (451.09 to 1201.65) | 1836.83 (1121.34 to 2759.97) | 494.67 (272.07 to 828.58) | 1342.17 (822.77 to 2031.27) | 99.29% (21.8% to 216.18%) | 226.26% (77.89% to 504.52%) | 74.29% (1.34% to 191.95%) |
|  |  |  | Age-standardized rate (per 100,000) | 111.58 (66.75 to 172.58) | 40.34 (21.26 to 66.24) | 169.49 (99.02 to 262.28) | 85.88 (52.15 to 129.38) | 45.94 (25.3 to 77.13) | 128.18 (77.44 to 193.96) | -23.04% (-52.86% to 21.77%) | 13.86% (-36.14% to 112.08%) | -24.37% (-55.19% to 26.38%) |
|  |  | Deaths | All age number | 31.84 (18.94 to 49.35) | 4.96 (2.59 to 8.15) | 26.87 (15.69 to 41.69) | 68.58 (41.69 to 103.3) | 19.17 (10.54 to 32.18) | 49.4 (29.81 to 75.48) | 115.4% (31.59% to 244.59%) | 286.31% (117.94% to 620.29%) | 83.84% (7.82% to 209.43%) |
|  |  |  | Age-standardized rate (per 100,000) | 4.44 (2.64 to 6.8) | 1.64 (0.85 to 2.66) | 6.74 (3.93 to 10.5) | 3.47 (2.1 to 5.28) | 1.96 (1.08 to 3.29) | 5.08 (3.05 to 7.78) | -21.78% (-51.27% to 22.3%) | 19.89% (-32.23% to 121.62%) | -24.71% (-54.62% to 26.08%) |
|  |  | YLDs | All age number | 7.59 (4.22 to 12.69) | 1.2 (0.58 to 2.08) | 6.39 (3.43 to 10.77) | 16.31 (8.66 to 26.6) | 4.27 (2.01 to 7.4) | 12.04 (6.41 to 19.72) | 114.74% (30.79% to 245.24%) | 254.46% (83.79% to 583.4%) | 88.42% (8.76% to 210.95%) |
|  |  |  | Age-standardized rate (per 100,000) | 0.97 (0.54 to 1.63) | 0.35 (0.17 to 0.61) | 1.48 (0.79 to 2.49) | 0.79 (0.41 to 1.28) | 0.41 (0.19 to 0.73) | 1.18 (0.63 to 1.93) | -19.25% (-50.17% to 26.7%) | 16.71% (-38.71% to 118.52%) | -20.08% (-52.62% to 30.88%) |
|  |  | YLLs | All age number | 914.08 (548.99 to 1431.36) | 150.41 (77.96 to 252.62) | 763.67 (446.76 to 1191.46) | 1820.53 (1112.67 to 2737.46) | 490.4 (269.92 to 822.16) | 1330.13 (816.32 to 2013.59) | 99.16% (21.63% to 216.08%) | 226.03% (77.61% to 503.92%) | 74.18% (1.22% to 191.98%) |
|  |  |  | Age-standardized rate (per 100,000) | 110.61 (66.14 to 171.27) | 39.99 (21.05 to 65.81) | 168.01 (97.96 to 259.95) | 85.09 (51.64 to 128.29) | 45.52 (25.12 to 76.48) | 127.0 (76.78 to 192.22) | -23.07% (-52.89% to 21.84%) | 13.83% (-36.1% to 112.08%) | -24.41% (-55.2% to 26.34%) |
|  | Metabolic risks | DALYs | All age number | 542.98 (220.31 to 876.58) | 270.93 (118.01 to 462.07) | 272.05 (100.38 to 460.57) | 2855.4 (908.61 to 4785.8) | 1506.54 (459.07 to 2581.89) | 1348.86 (448.38 to 2273.84) | 425.88% (260.71% to 595.29%) | 456.06% (227.1% to 704.81%) | 395.82% (239.43% to 622.16%) |
|  |  |  | Age-standardized rate (per 100,000) | 64.63 (24.23 to 105.81) | 70.37 (27.81 to 120.38) | 59.82 (20.35 to 103.49) | 135.84 (41.8 to 230.03) | 141.25 (41.82 to 241.95) | 130.26 (41.92 to 219.97) | 110.19% (51.82% to 172.83%) | 100.72% (24.34% to 183.17%) | 117.77% (53.04% to 209.62%) |
|  |  | Deaths | All age number | 17.88 (6.6 to 29.42) | 8.63 (3.36 to 14.68) | 9.25 (3.06 to 16.19) | 108.16 (30.83 to 184.96) | 57.62 (16.2 to 100.13) | 50.54 (15.06 to 86.09) | 504.84% (332.07% to 685.5%) | 567.81% (308.06% to 838.38%) | 446.12% (279.68% to 676.37%) |
|  |  |  | Age-standardized rate (per 100,000) | 2.52 (0.85 to 4.14) | 2.68 (0.97 to 4.59) | 2.38 (0.75 to 4.19) | 5.57 (1.54 to 9.58) | 5.89 (1.61 to 10.31) | 5.28 (1.52 to 9.08) | 121.26% (64.58% to 180.06%) | 119.37% (39.31% to 200.01%) | 121.77% (58.78% to 209.11%) |
|  |  | YLDs | All age number | 9.86 (3.14 to 17.68) | 5.78 (1.81 to 10.78) | 4.09 (1.37 to 7.3) | 93.87 (27.25 to 168.13) | 62.88 (14.67 to 114.32) | 30.99 (11.05 to 55.36) | 851.52% (553.61% to 1210.29%) | 988.38% (573.21% to 1548.93%) | 658.09% (416.9% to 1039.27%) |
|  |  |  | Age-standardized rate (per 100,000) | 1.25 (0.37 to 2.25) | 1.63 (0.44 to 3.14) | 0.95 (0.3 to 1.7) | 4.48 (1.28 to 7.98) | 5.84 (1.32 to 10.62) | 3.03 (1.05 to 5.48) | 257.81% (152.86% to 379.16%) | 258.04% (129.67% to 423.13%) | 217.49% (121.15% to 369.78%) |
|  |  | YLLs | All age number | 533.11 (217.28 to 861.46) | 265.15 (116.05 to 451.63) | 267.96 (98.77 to 453.46) | 2761.53 (880.06 to 4603.05) | 1443.66 (443.22 to 2480.15) | 1317.87 (439.19 to 2222.7) | 418.0% (256.12% to 584.3%) | 444.47% (220.88% to 687.32%) | 391.82% (236.22% to 615.86%) |
|  |  |  | Age-standardized rate (per 100,000) | 63.38 (23.88 to 103.84) | 68.74 (27.42 to 117.26) | 58.86 (19.98 to 101.98) | 131.36 (40.5 to 220.51) | 135.42 (40.43 to 232.59) | 127.23 (40.96 to 214.51) | 107.27% (50.1% to 168.99%) | 96.99% (21.97% to 178.51%) | 116.15% (51.85% to 207.58%) |
| Khorasan-e-Razavi | All risk factors | DALYs | All age number | 16320.38 (12034.59 to 22033.02) | 4807.55 (3105.79 to 7100.97) | 11512.83 (8243.37 to 16240.4) | 36063.23 (26547.55 to 47124.04) | 13122.61 (8437.95 to 18607.43) | 22940.63 (17024.78 to 29783.08) | 120.97% (64.22% to 189.43%) | 172.96% (78.9% to 286.44%) | 99.26% (37.68% to 178.07%) |
|  |  |  | Age-standardized rate (per 100,000) | 678.82 (499.95 to 916.8) | 407.53 (261.64 to 604.02) | 921.79 (664.68 to 1282.57) | 601.74 (446.09 to 787.5) | 424.31 (271.04 to 600.16) | 785.82 (585.32 to 1015.54) | -11.36% (-33.76% to 14.92%) | 4.12% (-30.14% to 46.27%) | -14.75% (-40.84% to 18.98%) |
|  |  | Deaths | All age number | 561.02 (411.61 to 762.48) | 151.91 (98.25 to 226.02) | 409.11 (292.43 to 574.89) | 1350.22 (998.08 to 1770.71) | 471.62 (301.94 to 665.06) | 878.6 (659.33 to 1134.66) | 140.67% (79.17% to 213.19%) | 210.46% (110.47% to 333.88%) | 114.76% (50.12% to 198.49%) |
|  |  |  | Age-standardized rate (per 100,000) | 27.23 (20.12 to 37.06) | 15.54 (9.98 to 23.48) | 38.04 (27.38 to 53.01) | 24.96 (18.32 to 32.71) | 16.98 (10.91 to 23.95) | 33.26 (24.85 to 42.98) | -8.35% (-31.23% to 18.83%) | 9.22% (-24.94% to 51.01%) | -12.57% (-37.68% to 20.67%) |
|  |  | YLDs | All age number | 223.65 (139.17 to 324.68) | 84.67 (46.86 to 129.69) | 138.98 (86.69 to 206.41) | 865.7 (516.2 to 1287.41) | 465.41 (229.23 to 745.21) | 400.29 (259.17 to 554.91) | 287.08% (178.78% to 411.17%) | 449.67% (249.94% to 670.46%) | 188.02% (104.19% to 307.77%) |
|  |  |  | Age-standardized rate (per 100,000) | 9.62 (6.02 to 14.01) | 7.3 (4.02 to 11.09) | 11.68 (7.35 to 17.29) | 14.27 (8.52 to 20.99) | 14.56 (7.3 to 23.14) | 14.07 (9.22 to 19.41) | 48.41% (8.7% to 95.54%) | 99.58% (29.75% to 175.83%) | 20.46% (-13.15% to 65.31%) |
|  |  | YLLs | All age number | 16096.73 (11830.67 to 21746.59) | 4722.88 (3061.26 to 6986.23) | 11373.85 (8142.5 to 16040.23) | 35197.53 (26047.73 to 46036.96) | 12657.19 (8206.96 to 17744.08) | 22540.34 (16759.64 to 29305.92) | 118.66% (62.41% to 186.24%) | 168.0% (75.92% to 277.65%) | 98.18% (36.92% to 176.69%) |
|  |  |  | Age-standardized rate (per 100,000) | 669.21 (492.92 to 905.11) | 400.23 (257.67 to 593.73) | 910.12 (655.89 to 1267.3) | 587.47 (437.57 to 769.67) | 409.75 (263.47 to 576.09) | 771.75 (576.17 to 997.23) | -12.21% (-34.5% to 13.75%) | 2.38% (-31.35% to 43.56%) | -15.2% (-41.12% to 18.37%) |
|  | Behavioral risks | DALYs | All age number | 14047.45 (10190.9 to 19629.81) | 3671.03 (2172.25 to 5762.62) | 10376.42 (7310.29 to 14890.73) | 26852.71 (20056.14 to 36735.46) | 7893.76 (4662.76 to 11747.09) | 18958.94 (13946.23 to 25048.43) | 91.16% (44.9% to 152.14%) | 115.03% (45.84% to 205.07%) | 82.71% (26.46% to 155.28%) |
|  |  |  | Age-standardized rate (per 100,000) | 584.67 (421.22 to 818.2) | 309.46 (183.24 to 492.25) | 831.39 (592.0 to 1197.61) | 446.99 (334.57 to 610.58) | 249.49 (148.86 to 371.37) | 651.88 (480.72 to 860.74) | -23.55% (-41.61% to -0.82%) | -19.38% (-44.27% to 13.55%) | -21.59% (-45.42% to 9.0%) |
|  |  | Deaths | All age number | 485.12 (351.48 to 681.99) | 114.83 (67.61 to 183.13) | 370.29 (262.22 to 532.9) | 1003.84 (753.99 to 1357.29) | 273.39 (164.28 to 405.08) | 730.46 (539.53 to 963.53) | 106.93% (57.39% to 169.94%) | 138.08% (65.24% to 235.84%) | 97.27% (39.21% to 175.96%) |
|  |  |  | Age-standardized rate (per 100,000) | 23.41 (16.98 to 32.97) | 11.64 (6.97 to 18.62) | 34.31 (24.55 to 49.4) | 18.5 (13.85 to 25.02) | 9.68 (5.84 to 14.32) | 27.68 (20.33 to 36.46) | -20.99% (-39.56% to 2.13%) | -16.81% (-41.83% to 15.75%) | -19.34% (-42.88% to 11.08%) |
|  |  | YLDs | All age number | 191.49 (117.54 to 285.19) | 65.53 (33.2 to 106.58) | 125.96 (77.4 to 190.25) | 626.76 (373.64 to 974.06) | 297.41 (117.15 to 536.4) | 329.35 (212.72 to 485.3) | 227.31% (133.7% to 341.5%) | 353.85% (172.89% to 563.93%) | 161.48% (85.87% to 266.87%) |
|  |  |  | Age-standardized rate (per 100,000) | 8.2 (5.08 to 12.31) | 5.55 (2.83 to 8.97) | 10.56 (6.56 to 15.74) | 10.2 (6.27 to 15.54) | 8.92 (3.56 to 15.76) | 11.61 (7.6 to 17.02) | 24.29% (-8.8% to 65.24%) | 60.54% (-1.12% to 127.93%) | 9.97% (-20.87% to 52.27%) |
|  |  | YLLs | All age number | 13855.97 (10052.02 to 19419.11) | 3605.5 (2140.5 to 5664.7) | 10250.46 (7203.29 to 14723.0) | 26225.95 (19594.2 to 35817.95) | 7596.35 (4533.23 to 11302.48) | 18629.59 (13682.24 to 24645.24) | 89.28% (43.6% to 149.46%) | 110.69% (42.64% to 197.63%) | 81.74% (25.53% to 153.91%) |
|  |  |  | Age-standardized rate (per 100,000) | 576.46 (415.34 to 808.69) | 303.9 (180.29 to 483.01) | 820.83 (585.11 to 1184.95) | 436.79 (327.17 to 593.34) | 240.57 (144.84 to 358.86) | 640.26 (470.19 to 846.1) | -24.23% (-42.04% to -1.7%) | -20.84% (-45.35% to 11.51%) | -22.0% (-45.71% to 8.49%) |
|  | Environmental/occupational risks | DALYs | All age number | 2164.18 (1350.38 to 3316.05) | 496.4 (260.69 to 852.69) | 1667.79 (1002.62 to 2587.14) | 5354.93 (3399.4 to 7719.65) | 1743.75 (978.8 to 2633.11) | 3611.18 (2258.13 to 5283.32) | 147.43% (64.59% to 270.77%) | 251.28% (92.28% to 527.91%) | 116.53% (31.99% to 234.47%) |
|  |  |  | Age-standardized rate (per 100,000) | 88.71 (55.29 to 135.82) | 43.83 (22.68 to 75.14) | 128.96 (77.13 to 198.63) | 88.53 (56.15 to 128.35) | 57.66 (32.31 to 87.54) | 120.22 (73.71 to 175.77) | -0.2% (-32.86% to 49.81%) | 31.56% (-27.16% to 133.93%) | -6.77% (-42.71% to 45.58%) |
|  |  | Deaths | All age number | 74.03 (46.29 to 113.87) | 16.62 (8.58 to 28.4) | 57.41 (34.27 to 90.05) | 198.56 (124.8 to 289.32) | 67.32 (37.6 to 103.14) | 131.24 (80.17 to 191.63) | 168.22% (79.34% to 306.46%) | 304.96% (124.86% to 624.8%) | 128.62% (39.54% to 260.27%) |
|  |  |  | Age-standardized rate (per 100,000) | 3.52 (2.2 to 5.34) | 1.8 (0.93 to 3.13) | 5.09 (3.08 to 7.81) | 3.63 (2.25 to 5.35) | 2.48 (1.38 to 3.8) | 4.82 (2.91 to 7.04) | 3.03% (-30.62% to 56.69%) | 37.33% (-22.06% to 143.95%) | -5.24% (-41.44% to 47.65%) |
|  |  | YLDs | All age number | 17.89 (9.57 to 29.81) | 3.94 (1.87 to 7.24) | 13.95 (7.34 to 23.58) | 47.5 (26.48 to 71.0) | 14.93 (7.31 to 23.23) | 32.57 (16.93 to 50.18) | 165.52% (71.2% to 317.6%) | 279.35% (106.26% to 619.73%) | 133.41% (38.1% to 303.03%) |
|  |  |  | Age-standardized rate (per 100,000) | 0.78 (0.42 to 1.29) | 0.38 (0.18 to 0.71) | 1.13 (0.6 to 1.91) | 0.82 (0.45 to 1.22) | 0.52 (0.25 to 0.81) | 1.13 (0.58 to 1.74) | 5.27% (-31.51% to 62.95%) | 36.13% (-27.06% to 164.72%) | -0.57% (-41.46% to 66.23%) |
|  |  | YLLs | All age number | 2146.29 (1339.87 to 3285.91) | 492.46 (258.79 to 847.14) | 1653.83 (995.68 to 2565.61) | 5307.43 (3365.7 to 7657.54) | 1728.82 (969.78 to 2610.01) | 3578.61 (2236.41 to 5231.95) | 147.28% (64.53% to 270.4%) | 251.06% (92.13% to 526.8%) | 116.38% (31.93% to 233.85%) |
|  |  |  | Age-standardized rate (per 100,000) | 87.93 (54.84 to 134.58) | 43.45 (22.47 to 74.62) | 127.83 (76.39 to 196.84) | 87.71 (55.63 to 127.27) | 57.14 (31.99 to 86.77) | 119.1 (72.93 to 174.18) | -0.25% (-33.01% to 49.83%) | 31.52% (-27.2% to 133.93%) | -6.83% (-42.75% to 45.4%) |
|  | Metabolic risks | DALYs | All age number | 1730.97 (655.71 to 2791.66) | 892.71 (364.58 to 1492.65) | 838.26 (299.46 to 1473.4) | 8396.8 (2723.13 to 13874.73) | 4603.22 (1501.92 to 7800.38) | 3793.58 (1191.53 to 6475.09) | 385.09% (247.59% to 520.31%) | 415.65% (218.75% to 617.93%) | 352.55% (217.69% to 522.68%) |
|  |  |  | Age-standardized rate (per 100,000) | 72.15 (25.83 to 119.18) | 76.78 (29.98 to 130.56) | 67.78 (22.03 to 120.63) | 142.56 (45.25 to 239.38) | 154.9 (49.17 to 262.48) | 129.8 (39.87 to 221.71) | 97.58% (46.09% to 147.59%) | 101.75% (30.05% to 173.79%) | 91.5% (33.51% to 160.65%) |
|  |  | Deaths | All age number | 58.64 (20.47 to 97.48) | 29.14 (11.13 to 50.44) | 29.51 (9.08 to 53.39) | 318.67 (97.53 to 540.89) | 174.24 (52.98 to 298.1) | 144.43 (43.05 to 249.26) | 443.4% (295.47% to 583.7%) | 497.96% (288.56% to 716.26%) | 389.52% (240.01% to 570.42%) |
|  |  |  | Age-standardized rate (per 100,000) | 2.93 (0.97 to 4.96) | 3.02 (1.1 to 5.27) | 2.85 (0.84 to 5.16) | 5.96 (1.75 to 10.17) | 6.42 (1.9 to 11.07) | 5.5 (1.58 to 9.56) | 103.35% (55.26% to 148.84%) | 112.4% (43.87% to 183.17%) | 92.71% (38.3% to 165.11%) |
|  |  | YLDs | All age number | 31.99 (10.84 to 55.36) | 19.37 (6.28 to 35.08) | 12.62 (4.5 to 22.98) | 270.02 (78.06 to 472.84) | 185.96 (48.17 to 337.35) | 84.05 (30.1 to 146.73) | 744.17% (515.48% to 1007.47%) | 860.28% (532.4% to 1244.16%) | 566.01% (367.96% to 881.14%) |
|  |  |  | Age-standardized rate (per 100,000) | 1.4 (0.45 to 2.42) | 1.75 (0.54 to 3.2) | 1.09 (0.37 to 2.03) | 4.63 (1.31 to 8.21) | 6.26 (1.61 to 11.53) | 2.94 (1.02 to 5.14) | 229.81% (146.24% to 324.08%) | 257.01% (141.55% to 393.6%) | 169.7% (90.74% to 293.96%) |
|  |  | YLLs | All age number | 1698.98 (643.5 to 2742.83) | 873.34 (358.21 to 1461.64) | 825.64 (295.03 to 1453.39) | 8126.78 (2634.36 to 13422.47) | 4417.26 (1437.62 to 7463.15) | 3709.53 (1162.94 to 6326.02) | 378.33% (242.97% to 512.52%) | 405.79% (213.73% to 605.52%) | 349.29% (215.94% to 517.91%) |
|  |  |  | Age-standardized rate (per 100,000) | 70.75 (25.31 to 116.88) | 75.03 (29.45 to 128.32) | 66.69 (21.63 to 118.81) | 137.93 (43.77 to 232.26) | 148.64 (47.11 to 252.01) | 126.86 (38.94 to 216.81) | 94.96% (43.55% to 144.69%) | 98.12% (28.03% to 169.26%) | 90.22% (32.7% to 158.53%) |
| Khuzestan | All risk factors | DALYs | All age number | 6910.0 (5064.69 to 9142.89) | 2553.2 (1618.2 to 3582.78) | 4356.8 (3003.46 to 6005.48) | 21671.29 (16554.51 to 27490.83) | 8475.35 (5395.56 to 12042.17) | 13195.94 (10325.46 to 17026.82) | 213.62% (138.71% to 302.47%) | 231.95% (129.54% to 373.11%) | 202.88% (116.43% to 331.51%) |
|  |  |  | Age-standardized rate (per 100,000) | 491.79 (362.87 to 650.44) | 357.07 (227.28 to 498.11) | 619.59 (430.53 to 849.91) | 523.76 (399.5 to 662.99) | 398.32 (250.45 to 559.64) | 653.67 (504.36 to 848.31) | 6.5% (-18.84% to 36.21%) | 11.55% (-22.09% to 57.84%) | 5.5% (-23.83% to 50.71%) |
|  |  | Deaths | All age number | 235.97 (173.68 to 312.85) | 80.4 (51.2 to 112.4) | 155.57 (107.32 to 213.96) | 763.47 (582.68 to 964.95) | 283.23 (178.33 to 390.15) | 480.25 (372.41 to 621.98) | 223.55% (146.68% to 313.25%) | 252.27% (148.53% to 391.28%) | 208.7% (122.17% to 340.47%) |
|  |  |  | Age-standardized rate (per 100,000) | 18.93 (13.96 to 25.12) | 12.84 (8.4 to 17.75) | 25.09 (17.61 to 34.2) | 20.73 (15.68 to 26.18) | 15.05 (9.51 to 20.71) | 26.67 (20.4 to 34.59) | 9.48% (-15.74% to 39.14%) | 17.21% (-17.32% to 62.94%) | 6.27% (-21.82% to 49.68%) |
|  |  | YLDs | All age number | 118.71 (74.29 to 175.07) | 59.11 (29.13 to 95.01) | 59.61 (37.76 to 89.61) | 630.65 (360.49 to 964.48) | 376.52 (168.87 to 641.29) | 254.13 (169.91 to 362.2) | 431.24% (294.63% to 577.94%) | 537.03% (333.08% to 826.79%) | 326.35% (206.63% to 500.75%) |
|  |  |  | Age-standardized rate (per 100,000) | 8.55 (5.35 to 12.49) | 8.27 (4.2 to 13.19) | 8.87 (5.67 to 13.13) | 14.95 (8.63 to 22.41) | 17.06 (7.94 to 28.6) | 12.99 (8.63 to 18.64) | 74.74% (31.91% to 121.92%) | 106.24% (42.4% to 191.47%) | 46.38% (5.96% to 104.64%) |
|  |  | YLLs | All age number | 6791.29 (4986.75 to 9018.67) | 2494.09 (1583.71 to 3503.25) | 4297.19 (2964.31 to 5922.88) | 21040.64 (16196.17 to 26561.98) | 8098.83 (5210.01 to 11387.89) | 12941.81 (10129.09 to 16654.83) | 209.82% (135.68% to 297.85%) | 224.72% (125.52% to 361.83%) | 201.17% (115.06% to 329.34%) |
|  |  |  | Age-standardized rate (per 100,000) | 483.23 (357.14 to 639.35) | 348.8 (222.17 to 487.96) | 610.72 (424.43 to 838.48) | 508.81 (389.14 to 643.69) | 381.26 (241.33 to 530.37) | 640.68 (494.43 to 830.61) | 5.29% (-19.83% to 34.87%) | 9.31% (-23.53% to 54.17%) | 4.91% (-24.24% to 49.94%) |
|  | Behavioral risks | DALYs | All age number | 5661.88 (4032.95 to 7740.49) | 1931.25 (1119.53 to 2860.46) | 3730.63 (2484.31 to 5254.1) | 15555.16 (11884.17 to 20378.92) | 5157.67 (2911.79 to 7708.48) | 10397.49 (8026.48 to 13417.67) | 174.73% (113.26% to 257.8%) | 167.06% (85.35% to 276.16%) | 178.71% (97.21% to 297.94%) |
|  |  |  | Age-standardized rate (per 100,000) | 403.96 (287.84 to 552.89) | 266.6 (153.75 to 396.24) | 533.57 (358.03 to 751.38) | 373.02 (287.22 to 485.48) | 233.26 (136.13 to 346.49) | 517.14 (397.21 to 668.68) | -7.66% (-28.26% to 18.97%) | -12.51% (-38.04% to 21.36%) | -3.08% (-30.47% to 38.31%) |
|  |  | Deaths | All age number | 193.25 (138.05 to 264.51) | 59.21 (34.42 to 87.19) | 134.03 (89.6 to 189.49) | 542.79 (424.04 to 708.08) | 162.63 (97.29 to 237.78) | 380.15 (292.17 to 494.03) | 180.88% (117.08% to 265.7%) | 174.65% (98.57% to 275.34%) | 183.63% (102.85% to 304.21%) |
|  |  |  | Age-standardized rate (per 100,000) | 15.44 (11.02 to 21.15) | 9.33 (5.5 to 13.63) | 21.58 (14.9 to 30.28) | 14.61 (11.35 to 18.94) | 8.38 (5.1 to 12.16) | 21.11 (16.14 to 27.32) | -5.38% (-26.35% to 22.76%) | -10.24% (-33.83% to 21.24%) | -2.21% (-29.02% to 39.39%) |
|  |  | YLDs | All age number | 98.39 (58.46 to 149.12) | 46.2 (20.73 to 78.79) | 52.19 (32.9 to 80.33) | 449.05 (239.24 to 701.86) | 247.47 (83.72 to 460.84) | 201.58 (136.72 to 285.43) | 356.39% (236.47% to 483.94%) | 435.66% (205.25% to 683.4%) | 286.23% (179.01% to 448.85%) |
|  |  |  | Age-standardized rate (per 100,000) | 7.03 (4.28 to 10.61) | 6.27 (2.86 to 10.58) | 7.77 (4.92 to 11.74) | 10.39 (5.9 to 15.67) | 10.57 (3.72 to 19.3) | 10.35 (6.97 to 14.64) | 47.68% (11.34% to 86.09%) | 68.49% (-0.56% to 141.7%) | 33.13% (-3.28% to 86.87%) |
|  |  | YLLs | All age number | 5563.49 (3965.34 to 7602.87) | 1885.05 (1095.65 to 2795.84) | 3678.44 (2450.67 to 5179.0) | 15106.11 (11552.6 to 19705.31) | 4910.2 (2821.54 to 7284.12) | 10195.91 (7847.45 to 13200.34) | 171.52% (110.42% to 253.54%) | 160.48% (82.83% to 264.93%) | 177.18% (96.22% to 296.23%) |
|  |  |  | Age-standardized rate (per 100,000) | 396.93 (283.64 to 543.02) | 260.33 (150.51 to 387.52) | 525.79 (352.17 to 741.08) | 362.63 (280.74 to 472.34) | 222.68 (132.03 to 327.08) | 506.79 (389.08 to 655.11) | -8.64% (-29.25% to 17.92%) | -14.46% (-38.96% to 17.45%) | -3.61% (-30.92% to 37.48%) |
|  | Environmental/occupational risks | DALYs | All age number | 1285.6 (843.92 to 1885.84) | 271.86 (152.95 to 449.42) | 1013.74 (620.8 to 1517.35) | 3686.65 (2479.03 to 5115.13) | 1010.23 (597.16 to 1551.33) | 2676.42 (1832.22 to 3649.11) | 186.76% (103.23% to 331.89%) | 271.59% (112.64% to 565.21%) | 164.02% (75.23% to 320.4%) |
|  |  |  | Age-standardized rate (per 100,000) | 91.83 (59.66 to 134.79) | 39.59 (22.36 to 65.34) | 140.76 (85.3 to 212.61) | 89.09 (59.7 to 124.6) | 48.83 (28.95 to 75.79) | 129.85 (87.34 to 179.52) | -2.99% (-32.0% to 46.19%) | 23.33% (-29.27% to 122.82%) | -7.75% (-38.3% to 44.89%) |
|  |  | Deaths | All age number | 44.77 (28.89 to 65.77) | 9.37 (5.28 to 15.35) | 35.4 (21.31 to 54.16) | 131.85 (87.2 to 184.22) | 36.91 (21.64 to 57.33) | 94.94 (63.05 to 131.4) | 194.51% (105.56% to 346.24%) | 293.86% (126.72% to 616.41%) | 168.2% (79.69% to 325.77%) |
|  |  |  | Age-standardized rate (per 100,000) | 3.58 (2.33 to 5.29) | 1.56 (0.89 to 2.53) | 5.57 (3.33 to 8.46) | 3.57 (2.32 to 5.01) | 2.02 (1.18 to 3.14) | 5.15 (3.37 to 7.19) | -0.49% (-30.79% to 51.81%) | 29.25% (-24.5% to 135.55%) | -7.46% (-38.46% to 44.73%) |
|  |  | YLDs | All age number | 10.68 (5.97 to 17.97) | 2.24 (1.16 to 3.87) | 8.44 (4.58 to 14.92) | 31.88 (18.35 to 46.84) | 8.5 (4.36 to 14.27) | 23.38 (13.78 to 34.17) | 198.42% (99.43% to 367.36%) | 279.22% (109.86% to 584.54%) | 176.96% (73.33% to 365.78%) |
|  |  |  | Age-standardized rate (per 100,000) | 0.8 (0.45 to 1.34) | 0.35 (0.18 to 0.6) | 1.24 (0.67 to 2.15) | 0.81 (0.46 to 1.2) | 0.43 (0.22 to 0.73) | 1.19 (0.68 to 1.74) | 0.25% (-33.21% to 57.7%) | 23.95% (-32.81% to 133.28%) | -4.26% (-39.65% to 60.74%) |
|  |  | YLLs | All age number | 1274.92 (837.23 to 1868.86) | 269.62 (151.61 to 445.73) | 1005.29 (616.05 to 1504.07) | 3654.77 (2458.61 to 5070.37) | 1001.73 (592.41 to 1537.28) | 2653.04 (1816.21 to 3624.13) | 186.67% (103.12% to 332.17%) | 271.53% (112.5% to 564.7%) | 163.91% (75.15% to 320.61%) |
|  |  |  | Age-standardized rate (per 100,000) | 91.03 (59.21 to 133.69) | 39.25 (22.13 to 64.67) | 139.52 (84.59 to 210.84) | 88.28 (59.15 to 123.45) | 48.4 (28.73 to 75.05) | 128.66 (86.57 to 178.04) | -3.02% (-32.01% to 46.19%) | 23.32% (-29.23% to 122.63%) | -7.78% (-38.29% to 44.76%) |
|  | Metabolic risks | DALYs | All age number | 868.57 (348.52 to 1441.96) | 489.13 (201.76 to 833.17) | 379.45 (142.37 to 649.84) | 5569.84 (1708.85 to 9486.78) | 2999.86 (812.91 to 5138.18) | 2569.98 (781.84 to 4583.79) | 541.26% (344.68% to 708.2%) | 513.31% (274.22% to 765.47%) | 577.3% (373.51% to 824.02%) |
|  |  |  | Age-standardized rate (per 100,000) | 62.11 (22.58 to 105.34) | 72.21 (26.73 to 124.63) | 53.09 (18.02 to 92.72) | 139.15 (40.6 to 239.37) | 151.0 (38.98 to 259.75) | 127.61 (35.92 to 229.34) | 124.04% (58.24% to 178.7%) | 109.11% (32.34% to 193.19%) | 140.35% (69.75% to 222.52%) |
|  |  | Deaths | All age number | 30.1 (10.6 to 51.56) | 16.77 (6.2 to 29.29) | 13.33 (4.29 to 23.5) | 202.86 (58.0 to 351.16) | 109.2 (28.04 to 186.35) | 93.65 (25.24 to 169.48) | 573.99% (380.48% to 739.5%) | 551.07% (320.91% to 808.44%) | 602.83% (394.56% to 846.67%) |
|  |  |  | Age-standardized rate (per 100,000) | 2.46 (0.81 to 4.27) | 2.78 (0.96 to 4.9) | 2.17 (0.65 to 3.85) | 5.64 (1.56 to 9.86) | 6.06 (1.53 to 10.39) | 5.24 (1.35 to 9.55) | 129.44% (65.98% to 185.7%) | 118.05% (46.43% to 199.07%) | 141.69% (76.32% to 223.4%) |
|  |  | YLDs | All age number | 19.78 (6.26 to 35.06) | 13.34 (3.92 to 24.83) | 6.44 (2.21 to 11.81) | 205.59 (56.26 to 365.69) | 144.44 (35.12 to 263.43) | 61.15 (20.59 to 108.12) | 939.48% (611.15% to 1229.74%) | 982.81% (566.12% to 1467.83%) | 849.72% (580.54% to 1228.19%) |
|  |  |  | Age-standardized rate (per 100,000) | 1.49 (0.44 to 2.67) | 2.08 (0.55 to 3.84) | 0.96 (0.31 to 1.73) | 5.2 (1.34 to 9.33) | 7.3 (1.63 to 13.38) | 3.11 (1.0 to 5.58) | 248.74% (148.47% to 342.89%) | 250.64% (132.28% to 400.2%) | 223.52% (136.14% to 349.21%) |
|  |  | YLLs | All age number | 848.8 (341.96 to 1411.96) | 475.79 (197.65 to 810.4) | 373.01 (139.55 to 641.26) | 5364.25 (1648.2 to 9106.61) | 2855.42 (776.47 to 4878.07) | 2508.84 (759.3 to 4484.9) | 531.98% (337.69% to 694.96%) | 500.14% (264.5% to 749.18%) | 572.6% (368.36% to 817.37%) |
|  |  |  | Age-standardized rate (per 100,000) | 60.62 (22.19 to 102.89) | 70.13 (26.16 to 121.25) | 52.13 (17.71 to 91.3) | 133.96 (39.2 to 230.53) | 143.71 (37.31 to 244.52) | 124.5 (34.86 to 224.22) | 120.98% (55.51% to 174.47%) | 104.91% (30.13% to 188.09%) | 138.82% (68.2% to 220.38%) |
| Kohgiluyeh and Boyer-Ahmad | All risk factors | DALYs | All age number | 824.32 (582.88 to 1260.41) | 250.53 (164.39 to 399.27) | 573.79 (377.98 to 893.11) | 2503.81 (1904.77 to 3407.12) | 882.29 (594.71 to 1272.11) | 1621.52 (1226.03 to 2300.76) | 203.74% (127.19% to 328.48%) | 252.17% (146.1% to 431.01%) | 182.6% (97.26% to 331.56%) |
|  |  |  | Age-standardized rate (per 100,000) | 416.4 (295.04 to 643.38) | 259.87 (169.17 to 418.27) | 548.53 (359.57 to 856.06) | 395.0 (299.91 to 540.58) | 273.64 (179.55 to 395.24) | 512.29 (388.29 to 726.26) | -5.14% (-29.66% to 33.98%) | 5.3% (-27.52% to 58.44%) | -6.61% (-34.77% to 41.92%) |
|  |  | Deaths | All age number | 26.69 (18.84 to 41.42) | 7.46 (4.87 to 12.13) | 19.23 (12.48 to 30.09) | 88.33 (66.08 to 121.46) | 28.71 (19.02 to 41.56) | 59.62 (45.23 to 84.49) | 230.92% (144.91% to 367.18%) | 284.84% (165.25% to 480.06%) | 210.0% (116.02% to 369.87%) |
|  |  |  | Age-standardized rate (per 100,000) | 16.18 (11.44 to 25.23) | 9.58 (6.24 to 15.68) | 22.07 (14.17 to 35.03) | 15.65 (11.78 to 21.64) | 10.37 (6.76 to 15.05) | 20.61 (15.61 to 29.27) | -3.25% (-27.93% to 34.87%) | 8.22% (-24.86% to 63.19%) | -6.61% (-33.93% to 40.16%) |
|  |  | YLDs | All age number | 11.75 (7.39 to 17.63) | 4.9 (2.72 to 7.79) | 6.86 (4.05 to 10.61) | 64.55 (40.06 to 96.95) | 34.8 (17.04 to 55.58) | 29.75 (19.47 to 42.82) | 449.19% (305.74% to 646.98%) | 610.66% (374.51% to 939.62%) | 333.9% (202.04% to 531.04%) |
|  |  |  | Age-standardized rate (per 100,000) | 6.16 (3.92 to 9.13) | 5.16 (2.89 to 8.08) | 7.02 (4.32 to 10.9) | 9.99 (6.17 to 14.77) | 10.34 (5.21 to 16.47) | 9.64 (6.31 to 13.93) | 62.08% (18.52% to 120.55%) | 100.28% (36.95% to 189.22%) | 37.33% (-2.74% to 97.01%) |
|  |  | YLLs | All age number | 812.57 (573.98 to 1242.97) | 245.63 (161.69 to 393.28) | 566.94 (372.92 to 884.28) | 2439.26 (1851.39 to 3320.34) | 847.49 (572.41 to 1218.89) | 1591.77 (1203.34 to 2265.59) | 200.19% (124.96% to 323.17%) | 245.03% (140.81% to 419.42%) | 180.77% (95.82% to 329.9%) |
|  |  |  | Age-standardized rate (per 100,000) | 410.24 (290.34 to 635.88) | 254.71 (166.17 to 411.07) | 541.51 (354.98 to 845.78) | 385.01 (292.77 to 527.17) | 263.3 (173.88 to 380.57) | 502.64 (380.26 to 714.84) | -6.15% (-30.71% to 32.69%) | 3.37% (-28.72% to 55.48%) | -7.18% (-35.13% to 41.18%) |
|  | Behavioral risks | DALYs | All age number | 683.33 (462.91 to 1101.97) | 187.44 (109.75 to 331.34) | 495.89 (315.6 to 803.54) | 1806.08 (1333.44 to 2615.71) | 533.65 (328.49 to 850.76) | 1272.43 (933.94 to 1833.84) | 164.3% (98.51% to 269.46%) | 184.7% (95.71% to 333.89%) | 156.6% (81.86% to 289.34%) |
|  |  |  | Age-standardized rate (per 100,000) | 347.53 (236.42 to 563.08) | 193.39 (114.71 to 344.29) | 477.15 (303.38 to 781.85) | 287.33 (212.27 to 416.06) | 160.9 (102.28 to 257.04) | 409.46 (299.08 to 585.72) | -17.32% (-37.81% to 15.21%) | -16.8% (-41.74% to 24.99%) | -14.19% (-38.89% to 30.58%) |
|  |  | Deaths | All age number | 22.32 (15.02 to 36.33) | 5.52 (3.26 to 9.99) | 16.8 (10.66 to 27.52) | 64.2 (47.1 to 94.0) | 16.63 (10.61 to 27.25) | 47.57 (34.53 to 68.53) | 187.65% (114.61% to 299.41%) | 201.23% (110.17% to 352.39%) | 183.18% (98.24% to 327.65%) |
|  |  |  | Age-standardized rate (per 100,000) | 13.44 (9.16 to 22.32) | 6.99 (4.05 to 12.61) | 19.16 (12.3 to 31.85) | 11.41 (8.35 to 16.77) | 5.85 (3.75 to 9.57) | 16.6 (12.02 to 23.98) | -15.09% (-36.24% to 15.29%) | -16.31% (-40.5% to 25.38%) | -13.38% (-38.65% to 29.85%) |
|  |  | YLDs | All age number | 9.94 (6.11 to 15.23) | 3.86 (1.94 to 6.41) | 6.09 (3.49 to 9.55) | 46.8 (26.97 to 72.95) | 23.4 (9.39 to 42.68) | 23.4 (15.18 to 34.72) | 370.76% (236.56% to 548.46%) | 506.8% (254.94% to 821.78%) | 284.55% (172.32% to 464.47%) |
|  |  |  | Age-standardized rate (per 100,000) | 5.19 (3.18 to 7.89) | 3.96 (2.01 to 6.52) | 6.23 (3.7 to 9.67) | 7.14 (4.24 to 10.82) | 6.52 (2.76 to 11.53) | 7.75 (5.04 to 11.43) | 37.7% (3.19% to 83.91%) | 64.72% (4.3% to 143.71%) | 24.47% (-11.51% to 82.47%) |
|  |  | YLLs | All age number | 673.39 (455.77 to 1087.87) | 183.59 (107.65 to 325.39) | 489.8 (311.22 to 794.16) | 1759.28 (1293.99 to 2562.62) | 510.24 (318.84 to 817.77) | 1249.03 (916.58 to 1803.78) | 161.26% (95.83% to 265.08%) | 177.93% (93.0% to 324.53%) | 155.01% (80.49% to 287.39%) |
|  |  |  | Age-standardized rate (per 100,000) | 342.34 (231.99 to 555.3) | 189.44 (112.3 to 337.7) | 470.92 (299.32 to 771.81) | 280.19 (206.51 to 408.4) | 154.38 (99.42 to 248.79) | 401.71 (293.56 to 576.19) | -18.16% (-38.41% to 14.04%) | -18.51% (-42.75% to 22.5%) | -14.7% (-39.26% to 30.05%) |
|  | Environmental/occupational risks | DALYs | All age number | 176.56 (112.23 to 263.04) | 37.6 (20.52 to 61.72) | 138.97 (87.87 to 218.52) | 491.19 (320.03 to 734.88) | 154.14 (87.66 to 240.42) | 337.05 (224.45 to 508.56) | 178.19% (72.9% to 338.8%) | 309.97% (120.68% to 664.14%) | 142.54% (45.0% to 314.73%) |
|  |  |  | Age-standardized rate (per 100,000) | 90.06 (58.04 to 132.67) | 41.38 (23.36 to 67.44) | 131.33 (84.11 to 203.02) | 77.56 (50.53 to 115.43) | 48.94 (28.4 to 76.83) | 105.24 (70.77 to 158.32) | -13.88% (-47.07% to 35.07%) | 18.26% (-35.23% to 123.62%) | -19.87% (-52.35% to 34.78%) |
|  |  | Deaths | All age number | 5.83 (3.75 to 8.63) | 1.21 (0.68 to 1.96) | 4.62 (2.92 to 7.27) | 17.81 (11.56 to 26.39) | 5.45 (3.15 to 8.41) | 12.36 (8.3 to 18.31) | 205.65% (87.79% to 378.31%) | 351.26% (149.36% to 750.22%) | 167.59% (59.41% to 353.91%) |
|  |  |  | Age-standardized rate (per 100,000) | 3.61 (2.35 to 5.34) | 1.68 (0.96 to 2.7) | 5.34 (3.4 to 8.08) | 3.17 (2.04 to 4.67) | 2.03 (1.18 to 3.11) | 4.24 (2.83 to 6.26) | -12.35% (-46.64% to 38.29%) | 20.84% (-33.27% to 126.91%) | -20.59% (-53.1% to 32.59%) |
|  |  | YLDs | All age number | 1.43 (0.83 to 2.4) | 0.3 (0.15 to 0.54) | 1.13 (0.63 to 2.0) | 4.41 (2.61 to 6.84) | 1.33 (0.7 to 2.15) | 3.08 (1.84 to 4.82) | 208.1% (94.9% to 400.02%) | 344.63% (145.79% to 710.48%) | 172.06% (60.79% to 398.31%) |
|  |  |  | Age-standardized rate (per 100,000) | 0.8 (0.48 to 1.33) | 0.37 (0.19 to 0.65) | 1.17 (0.66 to 2.06) | 0.74 (0.43 to 1.14) | 0.46 (0.24 to 0.74) | 1.01 (0.6 to 1.58) | -7.65% (-42.5% to 52.06%) | 23.79% (-31.95% to 128.35%) | -14.04% (-49.25% to 55.87%) |
|  |  | YLLs | All age number | 175.13 (111.24 to 260.41) | 37.3 (20.32 to 61.26) | 137.83 (87.15 to 216.95) | 486.78 (317.19 to 728.4) | 152.81 (86.84 to 238.26) | 333.97 (222.68 to 504.3) | 177.95% (72.84% to 338.21%) | 309.69% (120.49% to 664.03%) | 142.3% (44.81% to 314.58%) |
|  |  |  | Age-standardized rate (per 100,000) | 89.26 (57.52 to 131.41) | 41.01 (23.09 to 66.9) | 130.16 (83.25 to 201.37) | 76.82 (49.99 to 114.22) | 48.48 (28.14 to 76.09) | 104.23 (70.12 to 156.94) | -13.94% (-47.07% to 35.01%) | 18.21% (-35.29% to 123.69%) | -19.92% (-52.39% to 34.78%) |
|  | Metabolic risks | DALYs | All age number | 74.31 (34.86 to 117.58) | 38.07 (19.39 to 61.99) | 36.24 (15.82 to 60.87) | 548.3 (223.46 to 915.26) | 261.7 (99.16 to 453.54) | 286.6 (112.58 to 477.24) | 637.86% (403.77% to 882.7%) | 587.47% (323.25% to 922.91%) | 690.8% (427.47% to 1069.01%) |
|  |  |  | Age-standardized rate (per 100,000) | 35.5 (15.97 to 57.62) | 39.53 (18.71 to 66.22) | 32.27 (13.18 to 55.37) | 86.53 (33.04 to 147.79) | 86.56 (29.93 to 151.86) | 86.51 (31.72 to 147.94) | 143.73% (71.53% to 224.7%) | 119.0% (37.09% to 219.95%) | 168.11% (83.18% to 289.2%) |
|  |  | Deaths | All age number | 2.26 (1.02 to 3.69) | 1.15 (0.55 to 1.94) | 1.11 (0.45 to 1.93) | 19.03 (7.02 to 32.32) | 9.07 (3.08 to 15.87) | 9.96 (3.4 to 17.36) | 740.46% (483.27% to 1019.21%) | 686.87% (388.42% to 1045.08%) | 796.01% (503.7% to 1208.49%) |
|  |  |  | Age-standardized rate (per 100,000) | 1.35 (0.57 to 2.24) | 1.48 (0.65 to 2.52) | 1.24 (0.47 to 2.15) | 3.38 (1.19 to 5.8) | 3.41 (1.1 to 6.02) | 3.36 (1.07 to 5.86) | 150.4% (79.66% to 233.12%) | 130.83% (51.18% to 228.49%) | 170.61% (86.11% to 283.08%) |
|  |  | YLDs | All age number | 1.46 (0.57 to 2.52) | 0.95 (0.37 to 1.68) | 0.51 (0.21 to 0.96) | 18.64 (7.25 to 32.23) | 11.97 (4.2 to 21.03) | 6.67 (2.87 to 11.35) | 1178.11% (832.88% to 1610.88%) | 1166.26% (753.62% to 1767.33%) | 1199.95% (819.46% to 1807.14%) |
|  |  |  | Age-standardized rate (per 100,000) | 0.77 (0.26 to 1.36) | 1.09 (0.36 to 1.97) | 0.51 (0.2 to 0.95) | 3.03 (1.06 to 5.35) | 4.06 (1.29 to 7.43) | 2.02 (0.8 to 3.51) | 292.49% (193.13% to 419.06%) | 272.09% (156.54% to 437.1%) | 293.05% (180.26% to 460.0%) |
|  |  | YLLs | All age number | 72.85 (34.16 to 115.34) | 37.12 (18.98 to 60.22) | 35.73 (15.58 to 60.06) | 529.66 (214.89 to 888.13) | 249.73 (94.8 to 434.01) | 279.93 (110.42 to 465.19) | 627.05% (393.18% to 870.63%) | 572.72% (313.18% to 905.83%) | 683.5% (421.38% to 1060.81%) |
|  |  |  | Age-standardized rate (per 100,000) | 34.73 (15.62 to 56.38) | 38.44 (18.27 to 64.24) | 31.75 (12.98 to 54.73) | 83.5 (31.72 to 142.93) | 82.51 (28.69 to 143.86) | 84.49 (30.87 to 144.49) | 140.42% (68.83% to 220.75%) | 114.65% (35.26% to 214.98%) | 166.08% (81.24% to 287.23%) |
| Kurdistan | All risk factors | DALYs | All age number | 4440.61 (3168.35 to 6632.65) | 1149.11 (768.15 to 1740.41) | 3291.5 (2270.19 to 5051.97) | 9178.01 (6977.65 to 11901.58) | 2952.5 (1971.85 to 4186.12) | 6225.52 (4704.56 to 8194.37) | 106.68% (52.77% to 185.99%) | 156.94% (82.76% to 263.36%) | 89.14% (28.49% to 180.12%) |
|  |  |  | Age-standardized rate (per 100,000) | 732.08 (525.92 to 1093.39) | 388.43 (257.5 to 589.15) | 1007.92 (699.96 to 1548.75) | 554.76 (420.16 to 720.61) | 346.27 (230.32 to 493.45) | 772.87 (584.1 to 1022.7) | -24.22% (-44.56% to 3.77%) | -10.85% (-36.18% to 26.04%) | -23.32% (-48.07% to 12.9%) |
|  |  | Deaths | All age number | 155.05 (110.32 to 231.66) | 35.62 (23.59 to 54.65) | 119.43 (83.24 to 183.31) | 347.75 (264.59 to 451.52) | 107.12 (72.24 to 152.86) | 240.62 (180.59 to 318.57) | 124.28% (64.23% to 206.22%) | 200.76% (115.05% to 328.14%) | 101.47% (36.69% to 197.28%) |
|  |  |  | Age-standardized rate (per 100,000) | 29.37 (21.12 to 44.14) | 14.36 (9.41 to 22.2) | 41.64 (29.16 to 63.96) | 22.77 (17.17 to 29.62) | 13.77 (9.25 to 19.58) | 32.16 (23.96 to 42.46) | -22.45% (-43.36% to 4.12%) | -4.09% (-31.84% to 36.59%) | -22.77% (-47.74% to 13.06%) |
|  |  | YLDs | All age number | 60.34 (39.32 to 90.18) | 20.4 (11.48 to 31.83) | 39.94 (24.96 to 62.82) | 222.41 (139.62 to 335.93) | 108.18 (54.07 to 172.87) | 114.23 (76.83 to 167.09) | 268.59% (170.48% to 389.94%) | 430.17% (262.16% to 656.13%) | 186.04% (95.58% to 319.62%) |
|  |  |  | Age-standardized rate (per 100,000) | 10.23 (6.66 to 15.19) | 7.0 (4.0 to 10.82) | 12.74 (8.11 to 19.83) | 13.34 (8.53 to 20.04) | 12.36 (6.26 to 19.57) | 14.47 (9.72 to 21.17) | 30.36% (-3.2% to 73.78%) | 76.63% (23.04% to 147.51%) | 13.63% (-21.51% to 66.94%) |
|  |  | YLLs | All age number | 4380.27 (3122.62 to 6542.58) | 1128.71 (755.27 to 1713.89) | 3251.56 (2241.66 to 4996.74) | 8955.6 (6834.19 to 11598.3) | 2844.32 (1906.88 to 4042.82) | 6111.28 (4608.52 to 8043.16) | 104.45% (51.32% to 182.56%) | 152.0% (79.3% to 258.43%) | 87.95% (27.51% to 178.05%) |
|  |  |  | Age-standardized rate (per 100,000) | 721.85 (516.77 to 1078.13) | 381.43 (252.93 to 579.07) | 995.18 (690.82 to 1528.14) | 541.43 (411.55 to 702.21) | 333.91 (223.67 to 476.79) | 758.39 (571.99 to 1003.44) | -24.99% (-45.06% to 2.73%) | -12.46% (-37.23% to 24.13%) | -23.79% (-48.43% to 12.15%) |
|  | Behavioral risks | DALYs | All age number | 3936.93 (2726.29 to 6087.91) | 932.89 (594.87 to 1511.58) | 3004.04 (2069.15 to 4730.78) | 7184.59 (5387.21 to 9759.21) | 1913.9 (1181.82 to 2866.52) | 5270.7 (3883.53 to 7159.39) | 82.49% (35.38% to 151.63%) | 105.16% (45.05% to 193.96%) | 75.45% (18.95% to 159.97%) |
|  |  |  | Age-standardized rate (per 100,000) | 650.38 (455.73 to 1010.44) | 314.26 (196.9 to 511.71) | 920.09 (636.99 to 1441.74) | 434.12 (325.66 to 591.29) | 221.04 (138.0 to 331.75) | 657.05 (486.09 to 891.09) | -33.25% (-50.77% to -7.86%) | -29.66% (-49.92% to 0.85%) | -28.59% (-51.64% to 5.95%) |
|  |  | Deaths | All age number | 138.04 (96.9 to 215.09) | 28.73 (18.04 to 46.93) | 109.31 (75.5 to 172.46) | 272.04 (203.81 to 367.93) | 67.3 (42.49 to 100.85) | 204.74 (150.99 to 277.88) | 97.08% (44.75% to 172.84%) | 134.23% (69.47% to 231.05%) | 87.31% (25.83% to 175.96%) |
|  |  |  | Age-standardized rate (per 100,000) | 26.07 (18.32 to 40.87) | 11.52 (7.03 to 19.21) | 37.95 (26.44 to 59.81) | 17.79 (13.34 to 24.15) | 8.56 (5.41 to 12.81) | 27.4 (20.24 to 37.21) | -31.78% (-50.21% to -7.01%) | -25.74% (-45.83% to 3.39%) | -27.79% (-51.09% to 5.28%) |
|  |  | YLDs | All age number | 53.57 (34.03 to 82.43) | 16.8 (8.68 to 27.86) | 36.77 (22.99 to 58.33) | 169.25 (101.63 to 266.24) | 73.15 (29.56 to 127.08) | 96.1 (63.07 to 144.41) | 215.93% (125.63% to 328.12%) | 335.35% (166.38% to 540.81%) | 161.36% (76.77% to 281.16%) |
|  |  |  | Age-standardized rate (per 100,000) | 9.07 (5.76 to 13.87) | 5.66 (2.98 to 9.3) | 11.71 (7.39 to 18.59) | 10.06 (6.16 to 15.62) | 8.09 (3.34 to 13.94) | 12.22 (8.05 to 18.26) | 10.94% (-18.91% to 50.24%) | 42.87% (-8.8% to 106.25%) | 4.43% (-28.88% to 52.46%) |
|  |  | YLLs | All age number | 3883.36 (2689.2 to 6000.97) | 916.09 (584.54 to 1490.42) | 2967.27 (2045.7 to 4667.7) | 7015.34 (5268.66 to 9548.69) | 1840.74 (1150.93 to 2752.86) | 5174.6 (3810.55 to 7025.89) | 80.65% (33.77% to 149.34%) | 100.94% (41.85% to 187.65%) | 74.39% (18.01% to 158.58%) |
|  |  |  | Age-standardized rate (per 100,000) | 641.31 (450.1 to 995.61) | 308.6 (193.96 to 503.15) | 908.39 (629.57 to 1424.48) | 424.06 (318.92 to 580.1) | 212.95 (134.31 to 318.97) | 644.83 (476.28 to 876.59) | -33.88% (-51.31% to -8.97%) | -30.99% (-51.07% to -1.25%) | -29.01% (-51.98% to 5.33%) |
|  | Environmental/occupational risks | DALYs | All age number | 630.79 (367.22 to 1026.55) | 104.17 (51.3 to 181.72) | 526.61 (310.08 to 874.55) | 1357.06 (830.6 to 2146.26) | 359.64 (199.1 to 616.19) | 997.42 (608.99 to 1549.99) | 115.14% (35.34% to 250.12%) | 245.23% (83.43% to 536.8%) | 89.4% (11.03% to 214.17%) |
|  |  |  | Age-standardized rate (per 100,000) | 103.89 (60.87 to 171.14) | 36.56 (18.38 to 64.62) | 159.29 (92.12 to 265.9) | 81.58 (49.22 to 129.93) | 42.82 (23.47 to 73.48) | 121.77 (73.73 to 191.27) | -21.48% (-50.43% to 29.22%) | 17.14% (-36.8% to 121.34%) | -23.56% (-55.2% to 28.7%) |
|  |  | Deaths | All age number | 22.23 (13.17 to 36.31) | 3.42 (1.71 to 6.04) | 18.81 (10.83 to 31.37) | 51.29 (30.58 to 81.82) | 14.0 (7.47 to 23.67) | 37.29 (22.14 to 59.25) | 130.79% (45.98% to 275.42%) | 309.41% (121.28% to 673.42%) | 98.31% (18.12% to 234.83%) |
|  |  |  | Age-standardized rate (per 100,000) | 4.21 (2.45 to 6.98) | 1.45 (0.72 to 2.57) | 6.5 (3.63 to 10.9) | 3.34 (1.96 to 5.37) | 1.83 (0.96 to 3.08) | 4.9 (2.85 to 7.83) | -20.73% (-49.89% to 27.98%) | 26.4% (-29.54% to 135.12%) | -24.55% (-54.85% to 28.84%) |
|  |  | YLDs | All age number | 5.26 (2.81 to 8.75) | 0.83 (0.37 to 1.58) | 4.43 (2.39 to 7.42) | 12.17 (6.63 to 19.42) | 3.12 (1.49 to 5.69) | 9.04 (4.75 to 14.77) | 131.37% (42.53% to 278.29%) | 275.04% (96.27% to 611.82%) | 104.34% (14.58% to 256.41%) |
|  |  |  | Age-standardized rate (per 100,000) | 0.91 (0.48 to 1.54) | 0.32 (0.14 to 0.59) | 1.41 (0.75 to 2.41) | 0.75 (0.41 to 1.23) | 0.39 (0.18 to 0.7) | 1.14 (0.6 to 1.87) | -17.49% (-48.1% to 38.56%) | 21.7% (-35.81% to 131.6%) | -19.16% (-53.49% to 44.67%) |
|  |  | YLLs | All age number | 625.53 (364.19 to 1020.52) | 103.34 (50.91 to 180.02) | 522.19 (307.3 to 867.18) | 1344.89 (822.37 to 2127.21) | 356.51 (197.29 to 610.85) | 988.38 (603.1 to 1534.56) | 115.0% (35.3% to 250.09%) | 244.99% (83.14% to 536.68%) | 89.28% (11.03% to 214.09%) |
|  |  |  | Age-standardized rate (per 100,000) | 102.98 (60.38 to 169.86) | 36.24 (18.19 to 63.95) | 157.89 (91.3 to 264.26) | 80.83 (48.73 to 128.66) | 42.43 (23.26 to 72.76) | 120.63 (72.97 to 189.67) | -21.51% (-50.49% to 29.2%) | 17.1% (-36.91% to 121.36%) | -23.6% (-55.29% to 28.69%) |
|  | Metabolic risks | DALYs | All age number | 326.52 (138.15 to 532.93) | 163.73 (76.41 to 272.19) | 162.79 (59.93 to 285.28) | 1786.95 (557.39 to 2990.79) | 916.86 (292.73 to 1558.76) | 870.09 (267.99 to 1519.1) | 447.27% (280.61% to 619.18%) | 459.99% (236.23% to 697.58%) | 434.47% (262.2% to 678.56%) |
|  |  |  | Age-standardized rate (per 100,000) | 52.66 (20.38 to 87.06) | 56.17 (23.59 to 94.84) | 49.43 (16.85 to 86.32) | 109.29 (32.46 to 184.22) | 111.38 (34.15 to 190.69) | 107.13 (31.07 to 188.2) | 107.52% (48.84% to 165.22%) | 98.29% (22.73% to 174.49%) | 116.72% (48.01% to 211.49%) |
|  |  | Deaths | All age number | 10.97 (4.12 to 18.32) | 5.21 (2.15 to 8.82) | 5.76 (1.9 to 10.12) | 68.38 (19.08 to 115.93) | 35.11 (10.36 to 60.59) | 33.27 (8.82 to 58.33) | 523.3% (340.99% to 699.21%) | 573.66% (316.37% to 827.38%) | 477.73% (300.53% to 725.61%) |
|  |  |  | Age-standardized rate (per 100,000) | 2.07 (0.73 to 3.51) | 2.11 (0.83 to 3.59) | 2.04 (0.62 to 3.56) | 4.52 (1.23 to 7.69) | 4.6 (1.33 to 7.92) | 4.44 (1.12 to 7.85) | 118.08% (60.08% to 175.15%) | 117.94% (38.74% to 193.86%) | 118.12% (53.99% to 207.65%) |
|  |  | YLDs | All age number | 6.19 (2.1 to 11.02) | 3.63 (1.24 to 6.59) | 2.57 (0.92 to 4.51) | 60.56 (20.1 to 107.47) | 39.29 (12.55 to 72.44) | 21.27 (7.45 to 37.43) | 877.61% (599.33% to 1171.19%) | 982.81% (604.36% to 1468.04%) | 728.87% (465.85% to 1138.96%) |
|  |  |  | Age-standardized rate (per 100,000) | 1.06 (0.33 to 1.89) | 1.34 (0.43 to 2.45) | 0.82 (0.28 to 1.45) | 3.75 (1.19 to 6.68) | 4.79 (1.48 to 8.83) | 2.67 (0.91 to 4.76) | 255.43% (159.32% to 353.8%) | 257.75% (139.37% to 408.37%) | 223.52% (125.42% to 376.53%) |
|  |  | YLLs | All age number | 320.33 (135.76 to 522.5) | 160.1 (74.99 to 265.87) | 160.23 (59.05 to 280.28) | 1726.39 (536.79 to 2895.69) | 877.57 (280.19 to 1490.51) | 848.81 (259.13 to 1482.7) | 438.95% (274.63% to 608.82%) | 448.15% (227.45% to 678.86%) | 429.76% (255.39% to 672.56%) |
|  |  |  | Age-standardized rate (per 100,000) | 51.61 (20.02 to 85.38) | 54.83 (23.15 to 92.44) | 48.61 (16.52 to 84.73) | 105.53 (31.26 to 178.45) | 106.58 (32.72 to 182.81) | 104.46 (30.02 to 183.64) | 104.5% (46.24% to 161.73%) | 94.39% (19.56% to 169.6%) | 114.91% (46.78% to 209.08%) |
| Lorestan | All risk factors | DALYs | All age number | 3937.98 (2820.04 to 5834.89) | 1155.81 (714.97 to 1797.3) | 2782.17 (1910.54 to 4061.82) | 9107.07 (6957.42 to 11764.83) | 2884.5 (1780.14 to 4103.38) | 6222.57 (4810.67 to 7973.15) | 131.26% (76.11% to 203.34%) | 149.56% (68.4% to 267.62%) | 123.66% (60.15% to 212.76%) |
|  |  |  | Age-standardized rate (per 100,000) | 564.36 (405.89 to 827.34) | 346.04 (209.72 to 528.54) | 740.09 (513.98 to 1056.81) | 548.97 (415.24 to 708.53) | 321.83 (197.7 to 460.58) | 817.61 (634.33 to 1047.1) | -2.73% (-25.81% to 26.02%) | -7.0% (-37.34% to 36.16%) | 10.48% (-20.15% to 51.49%) |
|  |  | Deaths | All age number | 136.52 (98.04 to 197.85) | 36.35 (22.14 to 56.14) | 100.17 (68.94 to 144.01) | 334.34 (252.23 to 434.83) | 100.62 (62.04 to 145.14) | 233.72 (180.89 to 299.64) | 144.9% (85.52% to 216.7%) | 176.82% (87.1% to 300.99%) | 133.32% (69.27% to 219.65%) |
|  |  |  | Age-standardized rate (per 100,000) | 22.52 (16.23 to 32.33) | 12.92 (7.89 to 19.98) | 30.55 (21.53 to 43.58) | 22.44 (17.02 to 29.19) | 12.36 (7.58 to 17.77) | 34.85 (27.16 to 44.7) | -0.35% (-23.36% to 27.84%) | -4.38% (-35.35% to 36.91%) | 14.06% (-16.21% to 52.81%) |
|  |  | YLDs | All age number | 58.87 (38.69 to 87.27) | 22.99 (11.9 to 36.85) | 35.88 (23.41 to 54.38) | 247.97 (151.45 to 371.4) | 125.95 (59.53 to 212.04) | 122.01 (81.51 to 170.85) | 321.19% (214.73% to 455.21%) | 447.84% (264.1% to 694.79%) | 240.03% (143.7% to 378.24%) |
|  |  |  | Age-standardized rate (per 100,000) | 8.64 (5.72 to 12.67) | 6.93 (3.67 to 10.89) | 9.95 (6.61 to 14.94) | 14.69 (9.16 to 21.82) | 13.63 (6.51 to 22.83) | 16.41 (11.06 to 22.82) | 70.06% (28.16% to 123.61%) | 96.69% (31.56% to 181.2%) | 64.91% (20.24% to 127.68%) |
|  |  | YLLs | All age number | 3879.11 (2774.09 to 5755.98) | 1132.82 (698.84 to 1756.65) | 2746.28 (1886.02 to 4008.81) | 8859.1 (6767.53 to 11440.0) | 2758.55 (1709.93 to 3921.75) | 6100.55 (4703.63 to 7825.37) | 128.38% (73.75% to 199.57%) | 143.51% (64.95% to 258.99%) | 122.14% (59.08% to 210.85%) |
|  |  |  | Age-standardized rate (per 100,000) | 555.72 (399.03 to 816.21) | 339.11 (205.91 to 518.48) | 730.14 (506.95 to 1043.6) | 534.28 (404.63 to 687.46) | 308.2 (190.14 to 443.22) | 801.2 (620.29 to 1026.36) | -3.86% (-26.69% to 24.3%) | -9.12% (-38.87% to 32.98%) | 9.73% (-20.75% to 50.49%) |
|  | Behavioral risks | DALYs | All age number | 3391.42 (2362.37 to 5146.53) | 902.71 (514.67 to 1521.98) | 2488.71 (1674.2 to 3662.19) | 6850.83 (5225.22 to 9245.47) | 1755.24 (967.83 to 2683.52) | 5095.59 (3936.8 to 6732.82) | 102.0% (54.47% to 165.64%) | 94.44% (34.58% to 180.41%) | 104.75% (47.25% to 185.92%) |
|  |  |  | Age-standardized rate (per 100,000) | 487.8 (339.86 to 732.02) | 269.48 (154.44 to 444.28) | 663.36 (450.87 to 956.8) | 412.15 (314.1 to 555.33) | 192.31 (106.65 to 295.8) | 671.25 (522.28 to 878.79) | -15.51% (-34.79% to 9.71%) | -28.64% (-50.16% to 2.13%) | 1.19% (-26.68% to 39.29%) |
|  |  | Deaths | All age number | 118.2 (82.33 to 177.1) | 28.18 (16.16 to 46.65) | 90.02 (60.61 to 129.89) | 251.06 (191.32 to 338.15) | 58.99 (33.62 to 91.99) | 192.07 (149.34 to 251.72) | 112.4% (64.55% to 174.82%) | 109.29% (48.35% to 197.44%) | 113.37% (54.98% to 192.43%) |
|  |  |  | Age-standardized rate (per 100,000) | 19.46 (13.56 to 28.91) | 9.99 (5.57 to 16.44) | 27.39 (18.57 to 39.47) | 16.78 (12.67 to 22.66) | 7.15 (4.09 to 11.14) | 28.61 (22.11 to 37.39) | -13.79% (-33.19% to 9.92%) | -28.41% (-48.66% to -0.83%) | 4.48% (-24.09% to 42.14%) |
|  |  | YLDs | All age number | 50.33 (31.52 to 76.45) | 17.95 (8.53 to 31.2) | 32.39 (21.04 to 49.89) | 178.02 (100.89 to 276.28) | 80.03 (30.04 to 145.34) | 97.99 (65.07 to 139.06) | 253.69% (167.56% to 365.41%) | 345.94% (172.94% to 544.04%) | 202.57% (115.24% to 324.7%) |
|  |  |  | Age-standardized rate (per 100,000) | 7.39 (4.68 to 11.13) | 5.33 (2.59 to 9.12) | 8.97 (5.84 to 13.8) | 10.46 (6.2 to 15.96) | 8.38 (3.25 to 15.06) | 13.27 (8.82 to 18.56) | 41.61% (9.25% to 83.98%) | 57.03% (-1.97% to 124.16%) | 47.85% (7.22% to 104.77%) |
|  |  | YLLs | All age number | 3341.08 (2321.74 to 5078.27) | 884.76 (505.51 to 1489.7) | 2456.32 (1652.44 to 3617.26) | 6672.81 (5111.05 to 9017.51) | 1675.21 (936.24 to 2573.26) | 4997.6 (3870.06 to 6626.31) | 99.72% (52.52% to 162.94%) | 89.34% (31.36% to 171.22%) | 103.46% (46.34% to 184.2%) |
|  |  |  | Age-standardized rate (per 100,000) | 480.41 (333.75 to 722.64) | 264.15 (151.83 to 434.92) | 654.38 (445.14 to 944.72) | 401.69 (307.2 to 541.81) | 183.93 (103.39 to 284.23) | 657.98 (513.15 to 864.37) | -16.39% (-35.65% to 8.7%) | -30.37% (-50.72% to -0.5%) | 0.55% (-27.2% to 38.5%) |
|  | Environmental/occupational risks | DALYs | All age number | 543.04 (336.29 to 812.69) | 104.2 (52.1 to 178.49) | 438.84 (264.14 to 668.88) | 1229.23 (747.71 to 1816.69) | 323.68 (175.77 to 538.46) | 905.55 (554.76 to 1320.85) | 126.36% (49.44% to 250.68%) | 210.64% (69.15% to 483.47%) | 106.35% (27.03% to 231.68%) |
|  |  |  | Age-standardized rate (per 100,000) | 77.06 (47.19 to 113.68) | 31.97 (16.02 to 53.67) | 114.01 (68.36 to 174.36) | 73.35 (44.32 to 108.88) | 36.47 (19.61 to 60.7) | 115.59 (71.53 to 169.69) | -4.81% (-36.91% to 46.22%) | 14.09% (-37.2% to 114.06%) | 1.39% (-36.85% to 64.74%) |
|  |  | Deaths | All age number | 18.86 (11.55 to 27.93) | 3.43 (1.71 to 5.74) | 15.44 (9.14 to 23.59) | 44.9 (26.92 to 66.94) | 12.04 (6.4 to 19.87) | 32.86 (20.18 to 48.97) | 138.0% (55.76% to 266.69%) | 251.12% (93.88% to 569.23%) | 112.87% (32.06% to 246.23%) |
|  |  |  | Age-standardized rate (per 100,000) | 3.07 (1.84 to 4.65) | 1.26 (0.64 to 2.08) | 4.59 (2.67 to 7.0) | 2.97 (1.76 to 4.44) | 1.49 (0.79 to 2.46) | 4.74 (2.92 to 7.12) | -3.3% (-37.38% to 49.56%) | 18.9% (-32.94% to 124.05%) | 3.26% (-36.44% to 63.24%) |
|  |  | YLDs | All age number | 4.58 (2.58 to 7.53) | 0.84 (0.4 to 1.54) | 3.74 (2.09 to 6.27) | 11.11 (6.21 to 17.63) | 2.84 (1.44 to 4.81) | 8.28 (4.64 to 13.3) | 142.64% (56.29% to 282.41%) | 236.9% (84.96% to 534.89%) | 121.42% (30.49% to 261.19%) |
|  |  |  | Age-standardized rate (per 100,000) | 0.69 (0.39 to 1.12) | 0.28 (0.13 to 0.51) | 1.03 (0.59 to 1.7) | 0.69 (0.38 to 1.11) | 0.33 (0.17 to 0.57) | 1.11 (0.62 to 1.79) | 0.04% (-35.46% to 57.43%) | 18.61% (-34.99% to 124.99%) | 7.59% (-35.5% to 73.68%) |
|  |  | YLLs | All age number | 538.46 (333.69 to 806.09) | 103.36 (51.71 to 177.04) | 435.1 (261.55 to 664.36) | 1218.11 (741.3 to 1799.37) | 320.85 (174.44 to 533.53) | 897.27 (549.11 to 1310.88) | 126.22% (49.31% to 250.61%) | 210.43% (69.06% to 482.93%) | 106.22% (26.87% to 231.37%) |
|  |  |  | Age-standardized rate (per 100,000) | 76.37 (46.87 to 112.57) | 31.69 (15.89 to 53.22) | 112.98 (67.63 to 172.94) | 72.66 (43.85 to 107.76) | 36.14 (19.45 to 60.12) | 114.49 (70.74 to 168.22) | -4.85% (-36.97% to 46.11%) | 14.05% (-37.2% to 113.81%) | 1.33% (-36.98% to 64.7%) |
|  | Metabolic risks | DALYs | All age number | 398.38 (166.83 to 642.58) | 207.75 (94.77 to 334.88) | 190.63 (72.19 to 328.45) | 2159.56 (686.12 to 3654.43) | 1045.8 (312.25 to 1830.48) | 1113.76 (358.82 to 1903.52) | 442.09% (278.41% to 600.15%) | 403.39% (190.03% to 659.81%) | 484.26% (307.58% to 719.22%) |
|  |  |  | Age-standardized rate (per 100,000) | 56.02 (22.21 to 91.37) | 63.38 (27.42 to 103.89) | 49.94 (17.63 to 86.8) | 132.16 (40.41 to 225.58) | 120.85 (34.91 to 212.91) | 146.56 (44.79 to 251.65) | 135.91% (67.6% to 198.38%) | 90.69% (10.91% to 179.28%) | 193.48% (106.23% to 306.63%) |
|  |  | Deaths | All age number | 13.41 (5.21 to 21.87) | 6.74 (2.89 to 11.06) | 6.67 (2.28 to 11.72) | 80.31 (23.82 to 137.99) | 38.57 (10.69 to 68.6) | 41.74 (12.55 to 72.36) | 498.7% (321.94% to 657.43%) | 471.96% (231.63% to 738.3%) | 525.74% (340.96% to 764.78%) |
|  |  |  | Age-standardized rate (per 100,000) | 2.21 (0.81 to 3.67) | 2.42 (0.97 to 4.04) | 2.05 (0.66 to 3.58) | 5.47 (1.58 to 9.37) | 4.84 (1.31 to 8.63) | 6.29 (1.81 to 10.99) | 147.58% (76.78% to 204.57%) | 100.31% (20.02% to 182.08%) | 207.44% (121.26% to 311.39%) |
|  |  | YLDs | All age number | 8.4 (3.13 to 14.15) | 5.29 (1.95 to 9.29) | 3.11 (1.1 to 5.59) | 80.29 (26.7 to 143.42) | 51.78 (16.69 to 94.79) | 28.52 (10.44 to 52.3) | 856.2% (588.63% to 1135.58%) | 878.89% (496.02% to 1295.23%) | 817.59% (556.0% to 1224.16%) |
|  |  |  | Age-standardized rate (per 100,000) | 1.23 (0.43 to 2.09) | 1.68 (0.56 to 2.99) | 0.86 (0.29 to 1.53) | 4.89 (1.56 to 8.77) | 5.95 (1.78 to 11.04) | 3.77 (1.3 to 6.92) | 298.32% (186.72% to 406.88%) | 253.82% (117.73% to 398.78%) | 338.33% (219.82% to 521.59%) |
|  |  | YLLs | All age number | 389.98 (163.17 to 628.84) | 202.46 (92.62 to 324.72) | 187.52 (70.85 to 322.99) | 2079.27 (662.16 to 3529.27) | 994.03 (298.77 to 1730.01) | 1085.24 (350.36 to 1848.88) | 433.17% (269.35% to 589.35%) | 390.97% (182.15% to 644.59%) | 478.73% (302.73% to 710.97%) |
|  |  |  | Age-standardized rate (per 100,000) | 54.79 (21.76 to 89.51) | 61.7 (26.78 to 101.05) | 49.08 (17.3 to 85.28) | 127.27 (38.93 to 217.87) | 114.9 (33.27 to 201.58) | 142.79 (43.61 to 245.35) | 132.28% (64.43% to 194.71%) | 86.24% (8.13% to 173.12%) | 190.93% (103.97% to 302.71%) |
| Markazi | All risk factors | DALYs | All age number | 3978.47 (2871.48 to 5350.77) | 1287.84 (855.12 to 1901.7) | 2690.63 (1880.2 to 3821.47) | 7522.91 (5628.78 to 9596.06) | 2896.2 (1803.17 to 4068.46) | 4626.71 (3535.02 to 5899.2) | 89.09% (42.57% to 153.94%) | 124.89% (59.87% to 221.28%) | 71.96% (23.5% to 151.7%) |
|  |  |  | Age-standardized rate (per 100,000) | 588.92 (422.58 to 794.4) | 383.05 (254.87 to 559.65) | 779.59 (544.72 to 1108.21) | 465.83 (347.81 to 594.67) | 353.41 (220.17 to 496.2) | 583.97 (445.38 to 748.88) | -20.9% (-40.14% to 5.48%) | -7.74% (-34.41% to 30.51%) | -25.09% (-46.59% to 8.56%) |
|  |  | Deaths | All age number | 135.35 (97.64 to 183.16) | 40.25 (26.86 to 58.81) | 95.1 (66.36 to 135.44) | 292.66 (217.1 to 375.72) | 111.68 (69.7 to 155.71) | 180.99 (136.94 to 234.73) | 116.22% (64.19% to 190.54%) | 177.47% (98.68% to 290.34%) | 90.3% (36.27% to 174.87%) |
|  |  |  | Age-standardized rate (per 100,000) | 23.04 (16.63 to 31.32) | 14.31 (9.48 to 20.9) | 31.22 (21.7 to 44.26) | 18.68 (13.85 to 23.98) | 14.03 (8.77 to 19.57) | 23.67 (17.81 to 30.83) | -18.93% (-38.0% to 7.26%) | -2.0% (-29.57% to 36.35%) | -24.18% (-45.64% to 8.09%) |
|  |  | YLDs | All age number | 62.25 (40.08 to 89.84) | 26.68 (14.78 to 41.68) | 35.57 (23.27 to 53.04) | 215.23 (124.8 to 317.41) | 122.12 (57.75 to 196.29) | 93.11 (59.75 to 133.56) | 245.77% (153.95% to 352.92%) | 357.73% (219.89% to 546.58%) | 161.78% (86.92% to 275.12%) |
|  |  |  | Age-standardized rate (per 100,000) | 9.45 (6.07 to 13.65) | 7.99 (4.36 to 12.32) | 10.71 (7.11 to 15.76) | 13.24 (7.75 to 19.47) | 14.69 (7.02 to 23.56) | 11.93 (7.69 to 17.11) | 40.12% (3.96% to 82.35%) | 83.88% (27.9% to 158.12%) | 11.37% (-21.66% to 56.12%) |
|  |  | YLLs | All age number | 3916.22 (2827.32 to 5280.48) | 1261.16 (839.51 to 1863.32) | 2655.06 (1853.72 to 3771.03) | 7307.68 (5476.87 to 9282.58) | 2774.08 (1745.25 to 3875.69) | 4533.6 (3472.72 to 5781.46) | 86.6% (40.93% to 150.74%) | 119.96% (56.91% to 213.08%) | 70.75% (22.74% to 149.56%) |
|  |  |  | Age-standardized rate (per 100,000) | 579.47 (415.83 to 783.81) | 375.06 (249.35 to 548.6) | 768.88 (537.5 to 1092.64) | 452.59 (338.18 to 576.9) | 338.72 (214.08 to 474.63) | 572.04 (436.41 to 733.78) | -21.9% (-40.8% to 4.41%) | -9.69% (-35.67% to 27.71%) | -25.6% (-46.95% to 7.75%) |
|  | Behavioral risks | DALYs | All age number | 3410.65 (2388.25 to 4811.7) | 1004.76 (642.66 to 1565.67) | 2405.89 (1604.44 to 3517.47) | 5512.58 (4055.52 to 7187.59) | 1751.94 (1041.21 to 2620.8) | 3760.64 (2837.81 to 4883.14) | 61.63% (22.51% to 115.82%) | 74.36% (21.09% to 151.19%) | 56.31% (9.58% to 127.92%) |
|  |  |  | Age-standardized rate (per 100,000) | 505.67 (356.04 to 718.33) | 297.64 (190.11 to 467.45) | 698.57 (465.21 to 1017.89) | 339.79 (250.82 to 443.41) | 210.77 (126.93 to 316.2) | 475.21 (359.43 to 618.65) | -32.8% (-48.75% to -11.07%) | -29.19% (-49.75% to 0.31%) | -31.97% (-52.16% to -2.47%) |
|  |  | Deaths | All age number | 116.59 (82.18 to 166.26) | 31.04 (19.88 to 48.34) | 85.55 (57.0 to 124.84) | 212.51 (158.24 to 279.18) | 64.72 (40.3 to 97.42) | 147.79 (110.6 to 193.95) | 82.26% (37.6% to 141.15%) | 108.5% (50.41% to 188.25%) | 72.74% (21.05% to 149.27%) |
|  |  |  | Age-standardized rate (per 100,000) | 19.78 (13.95 to 28.27) | 10.97 (7.0 to 17.17) | 28.03 (18.93 to 40.8) | 13.53 (10.06 to 17.8) | 8.06 (5.04 to 12.19) | 19.36 (14.49 to 25.31) | -31.6% (-47.18% to -10.19%) | -26.53% (-46.85% to 0.53%) | -30.95% (-51.05% to -2.55%) |
|  |  | YLDs | All age number | 53.46 (34.37 to 78.63) | 21.21 (10.94 to 34.49) | 32.24 (20.87 to 48.67) | 153.69 (83.2 to 234.9) | 78.16 (29.27 to 139.33) | 75.53 (47.93 to 108.35) | 187.5% (106.22% to 277.44%) | 268.42% (124.2% to 444.61%) | 134.25% (66.39% to 235.06%) |
|  |  |  | Age-standardized rate (per 100,000) | 8.1 (5.23 to 11.81) | 6.28 (3.22 to 10.06) | 9.7 (6.33 to 14.68) | 9.36 (5.2 to 14.16) | 9.18 (3.5 to 16.16) | 9.7 (6.16 to 13.89) | 15.48% (-16.43% to 52.65%) | 46.21% (-10.1% to 110.34%) | 0.03% (-30.32% to 40.57%) |
|  |  | YLLs | All age number | 3357.19 (2352.25 to 4749.5) | 983.54 (630.13 to 1531.47) | 2373.65 (1582.83 to 3471.31) | 5358.9 (3962.98 to 6953.41) | 1673.78 (1006.38 to 2507.47) | 3685.12 (2789.8 to 4781.13) | 59.62% (21.12% to 113.51%) | 70.18% (19.82% to 144.52%) | 55.25% (8.86% to 126.78%) |
|  |  |  | Age-standardized rate (per 100,000) | 497.57 (349.96 to 707.85) | 291.36 (186.82 to 457.17) | 688.87 (457.92 to 1003.45) | 330.43 (244.95 to 429.2) | 201.59 (122.66 to 302.75) | 465.51 (352.71 to 606.29) | -33.59% (-49.4% to -12.19%) | -30.81% (-50.48% to -2.22%) | -32.42% (-52.42% to -2.93%) |
|  | Environmental/occupational risks | DALYs | All age number | 524.05 (314.31 to 819.99) | 102.6 (51.73 to 179.21) | 421.45 (251.11 to 679.0) | 973.83 (618.65 to 1436.19) | 276.04 (157.01 to 454.55) | 697.79 (443.32 to 1056.01) | 85.83% (20.61% to 184.77%) | 169.03% (49.97% to 385.42%) | 65.57% (2.05% to 171.07%) |
|  |  |  | Age-standardized rate (per 100,000) | 76.38 (45.27 to 120.23) | 31.86 (16.05 to 55.65) | 118.4 (69.34 to 188.87) | 60.15 (38.15 to 89.35) | 33.97 (19.3 to 56.03) | 86.87 (55.16 to 132.48) | -21.24% (-48.18% to 21.33%) | 6.62% (-39.99% to 90.28%) | -26.63% (-54.93% to 19.12%) |
|  |  | Deaths | All age number | 17.9 (10.73 to 28.0) | 3.43 (1.72 to 6.05) | 14.47 (8.49 to 23.3) | 37.41 (23.2 to 56.53) | 11.55 (6.47 to 18.79) | 25.85 (16.19 to 39.13) | 108.92% (36.56% to 220.41%) | 236.55% (86.59% to 499.17%) | 78.65% (10.83% to 190.34%) |
|  |  |  | Age-standardized rate (per 100,000) | 2.99 (1.72 to 4.85) | 1.3 (0.63 to 2.25) | 4.57 (2.62 to 7.34) | 2.38 (1.48 to 3.62) | 1.46 (0.81 to 2.38) | 3.34 (2.08 to 5.12) | -20.24% (-47.74% to 21.59%) | 12.01% (-35.34% to 98.57%) | -26.8% (-54.67% to 18.22%) |
|  |  | YLDs | All age number | 4.42 (2.42 to 7.2) | 0.83 (0.4 to 1.48) | 3.59 (1.94 to 5.9) | 9.06 (4.82 to 14.13) | 2.54 (1.25 to 4.51) | 6.52 (3.47 to 10.18) | 104.8% (29.69% to 223.68%) | 204.88% (68.42% to 461.67%) | 81.6% (6.84% to 212.05%) |
|  |  |  | Age-standardized rate (per 100,000) | 0.68 (0.37 to 1.11) | 0.28 (0.13 to 0.5) | 1.05 (0.56 to 1.76) | 0.57 (0.3 to 0.88) | 0.32 (0.15 to 0.57) | 0.82 (0.43 to 1.29) | -16.4% (-46.77% to 29.77%) | 12.67% (-37.47% to 105.19%) | -21.63% (-54.25% to 34.6%) |
|  |  | YLLs | All age number | 519.63 (311.53 to 813.0) | 101.77 (51.3 to 178.09) | 417.86 (248.8 to 672.92) | 964.77 (613.89 to 1425.27) | 273.5 (155.42 to 450.04) | 691.27 (438.78 to 1046.86) | 85.66% (20.58% to 184.69%) | 168.74% (49.64% to 385.26%) | 65.43% (1.93% to 170.94%) |
|  |  |  | Age-standardized rate (per 100,000) | 75.7 (44.91 to 119.06) | 31.58 (15.89 to 55.13) | 117.34 (68.68 to 187.13) | 59.58 (37.85 to 88.57) | 33.65 (19.13 to 55.4) | 86.05 (54.59 to 131.3) | -21.29% (-48.2% to 21.31%) | 6.57% (-40.07% to 90.23%) | -26.67% (-54.98% to 19.27%) |
|  | Metabolic risks | DALYs | All age number | 443.79 (162.66 to 731.18) | 243.5 (90.93 to 414.39) | 200.29 (73.12 to 337.67) | 1970.25 (606.19 to 3313.01) | 1107.09 (338.03 to 1905.16) | 863.16 (279.02 to 1490.43) | 343.96% (221.14% to 478.49%) | 354.66% (201.87% to 534.31%) | 330.95% (187.25% to 515.59%) |
|  |  |  | Age-standardized rate (per 100,000) | 65.15 (22.69 to 108.87) | 73.09 (25.73 to 124.09) | 57.43 (20.48 to 97.62) | 124.02 (37.76 to 209.74) | 138.44 (41.52 to 238.49) | 109.24 (34.54 to 188.87) | 90.36% (40.52% to 143.42%) | 89.41% (29.57% to 160.85%) | 90.19% (28.37% to 165.16%) |
|  |  | Deaths | All age number | 14.78 (4.96 to 24.79) | 7.94 (2.73 to 13.57) | 6.84 (2.32 to 11.81) | 79.22 (22.98 to 133.55) | 45.39 (12.92 to 78.83) | 33.83 (10.08 to 58.47) | 436.05% (291.93% to 587.13%) | 471.66% (292.01% to 687.84%) | 394.71% (237.44% to 595.85%) |
|  |  |  | Age-standardized rate (per 100,000) | 2.54 (0.81 to 4.27) | 2.83 (0.92 to 4.93) | 2.26 (0.74 to 3.92) | 5.1 (1.47 to 8.65) | 5.78 (1.63 to 10.08) | 4.42 (1.28 to 7.68) | 100.71% (50.23% to 152.13%) | 103.96% (39.87% to 178.8%) | 95.25% (35.04% to 170.07%) |
|  |  | YLDs | All age number | 9.0 (2.43 to 15.77) | 5.84 (1.5 to 10.52) | 3.16 (1.12 to 5.75) | 71.5 (21.15 to 131.08) | 50.2 (12.93 to 92.36) | 21.3 (7.38 to 38.96) | 694.05% (493.48% to 942.11%) | 759.34% (499.03% to 1139.61%) | 573.46% (362.39% to 875.76%) |
|  |  |  | Age-standardized rate (per 100,000) | 1.37 (0.36 to 2.41) | 1.81 (0.45 to 3.23) | 0.96 (0.33 to 1.74) | 4.53 (1.33 to 8.32) | 6.3 (1.63 to 11.6) | 2.72 (0.9 to 4.98) | 229.3% (146.82% to 329.13%) | 247.27% (148.56% to 394.74%) | 182.41% (98.4% to 295.62%) |
|  |  | YLLs | All age number | 434.79 (160.33 to 716.79) | 237.66 (89.81 to 404.53) | 197.13 (71.92 to 332.77) | 1898.75 (584.76 to 3193.67) | 1056.89 (323.83 to 1807.24) | 841.86 (271.62 to 1450.84) | 336.71% (215.97% to 470.4%) | 344.71% (194.98% to 519.96%) | 327.06% (184.02% to 510.35%) |
|  |  |  | Age-standardized rate (per 100,000) | 63.78 (22.36 to 106.87) | 71.28 (25.37 to 120.42) | 56.47 (20.15 to 95.98) | 119.5 (36.37 to 202.24) | 132.14 (39.84 to 226.87) | 106.52 (33.62 to 183.82) | 87.37% (38.4% to 139.41%) | 85.4% (26.88% to 154.94%) | 88.62% (27.32% to 162.39%) |
| Mazandaran | All risk factors | DALYs | All age number | 6779.27 (4902.11 to 9525.25) | 2505.26 (1578.36 to 3697.54) | 4274.0 (2935.31 to 6177.03) | 21131.0 (15325.4 to 28143.93) | 8050.32 (4899.92 to 11384.96) | 13080.68 (9951.7 to 17646.87) | 211.7% (135.0% to 316.42%) | 221.34% (115.76% to 367.54%) | 206.05% (112.01% to 352.58%) |
|  |  |  | Age-standardized rate (per 100,000) | 509.84 (372.16 to 721.87) | 367.87 (233.17 to 543.27) | 650.98 (449.24 to 939.16) | 515.52 (377.83 to 689.68) | 383.24 (233.95 to 540.3) | 652.22 (495.93 to 875.86) | 1.11% (-23.12% to 35.22%) | 4.18% (-29.66% to 49.35%) | 0.19% (-30.05% to 46.09%) |
|  |  | Deaths | All age number | 229.52 (167.19 to 326.36) | 79.21 (50.73 to 116.65) | 150.31 (103.61 to 218.38) | 792.38 (592.7 to 1052.2) | 284.88 (176.81 to 399.92) | 507.5 (386.18 to 678.38) | 245.24% (161.37% to 361.88%) | 259.65% (143.77% to 409.44%) | 237.64% (135.19% to 393.47%) |
|  |  |  | Age-standardized rate (per 100,000) | 20.28 (14.73 to 28.89) | 13.84 (8.79 to 20.44) | 27.08 (18.85 to 38.64) | 20.97 (15.71 to 27.74) | 14.69 (9.2 to 20.53) | 27.43 (20.7 to 36.72) | 3.4% (-21.31% to 37.64%) | 6.13% (-25.71% to 50.09%) | 1.3% (-27.15% to 45.12%) |
|  |  | YLDs | All age number | 132.57 (81.02 to 195.0) | 67.77 (33.19 to 111.38) | 64.81 (41.89 to 95.69) | 719.11 (410.62 to 1087.34) | 427.17 (193.26 to 695.53) | 291.95 (191.56 to 413.8) | 442.42% (290.71% to 613.23%) | 530.36% (318.27% to 812.08%) | 350.47% (215.57% to 558.68%) |
|  |  |  | Age-standardized rate (per 100,000) | 10.06 (6.23 to 14.56) | 9.83 (4.94 to 15.99) | 10.33 (6.62 to 15.04) | 17.27 (9.95 to 26.07) | 19.8 (8.95 to 32.26) | 14.78 (9.73 to 20.86) | 71.73% (27.12% to 124.72%) | 101.32% (37.0% to 189.08%) | 43.14% (1.67% to 107.15%) |
|  |  | YLLs | All age number | 6646.69 (4806.84 to 9384.63) | 2437.5 (1539.2 to 3601.71) | 4209.19 (2885.57 to 6099.05) | 20411.89 (14953.32 to 27240.01) | 7623.15 (4659.87 to 10815.58) | 12788.73 (9736.3 to 17257.78) | 207.1% (132.22% to 310.4%) | 212.75% (108.91% to 358.59%) | 203.83% (110.55% to 349.07%) |
|  |  |  | Age-standardized rate (per 100,000) | 499.78 (364.0 to 709.01) | 358.04 (227.88 to 528.7) | 640.65 (441.34 to 924.6) | 498.25 (369.36 to 668.08) | 363.45 (222.82 to 515.54) | 637.44 (485.27 to 856.28) | -0.31% (-24.13% to 33.38%) | 1.51% (-31.44% to 46.26%) | -0.5% (-30.64% to 45.07%) |
|  | Behavioral risks | DALYs | All age number | 5780.83 (4031.03 to 8605.86) | 1968.88 (1106.4 to 3028.57) | 3811.96 (2557.61 to 5587.07) | 15732.93 (11486.22 to 21346.35) | 4993.9 (2749.17 to 7679.82) | 10739.03 (7991.54 to 14493.32) | 172.16% (105.48% to 269.68%) | 153.64% (75.12% to 263.42%) | 181.72% (93.55% to 316.54%) |
|  |  |  | Age-standardized rate (per 100,000) | 435.83 (302.05 to 646.93) | 286.77 (161.07 to 436.7) | 583.56 (394.65 to 853.82) | 382.1 (280.67 to 522.53) | 234.34 (129.62 to 362.02) | 534.9 (396.81 to 724.06) | -12.33% (-33.63% to 18.56%) | -18.28% (-42.63% to 16.1%) | -8.34% (-36.33% to 33.44%) |
|  |  | Deaths | All age number | 196.4 (135.62 to 292.61) | 61.29 (34.61 to 93.82) | 135.11 (90.95 to 199.03) | 589.23 (435.32 to 807.99) | 169.97 (96.64 to 258.64) | 419.26 (309.98 to 567.06) | 200.01% (126.79% to 309.75%) | 177.31% (95.97% to 295.53%) | 210.31% (117.78% to 354.72%) |
|  |  |  | Age-standardized rate (per 100,000) | 17.3 (12.05 to 25.44) | 10.63 (6.1 to 16.43) | 24.32 (16.41 to 35.79) | 15.55 (11.57 to 21.33) | 8.66 (5.05 to 13.06) | 22.65 (16.77 to 30.83) | -10.1% (-31.19% to 20.86%) | -18.49% (-41.11% to 14.26%) | -6.9% (-32.93% to 34.62%) |
|  |  | YLDs | All age number | 112.45 (67.51 to 169.66) | 53.96 (22.91 to 91.65) | 58.49 (37.08 to 87.86) | 510.04 (274.47 to 807.87) | 272.21 (89.32 to 501.27) | 237.83 (153.37 to 344.06) | 353.55% (235.29% to 505.84%) | 404.45% (214.79% to 644.44%) | 306.59% (183.94% to 495.45%) |
|  |  |  | Age-standardized rate (per 100,000) | 8.48 (5.16 to 12.75) | 7.65 (3.3 to 12.74) | 9.33 (6.0 to 13.84) | 12.18 (6.73 to 19.11) | 12.42 (4.19 to 22.65) | 12.05 (7.86 to 17.35) | 43.63% (6.82% to 90.86%) | 62.38% (3.53% to 136.01%) | 29.18% (-8.85% to 84.31%) |
|  |  | YLLs | All age number | 5668.38 (3931.32 to 8478.07) | 1914.91 (1082.35 to 2937.19) | 3753.46 (2516.84 to 5509.53) | 15222.89 (11142.42 to 20839.43) | 4721.69 (2637.93 to 7214.4) | 10501.2 (7813.66 to 14165.18) | 168.56% (101.76% to 266.03%) | 146.57% (70.6% to 254.45%) | 179.77% (92.32% to 314.54%) |
|  |  |  | Age-standardized rate (per 100,000) | 427.35 (295.42 to 636.27) | 279.12 (157.19 to 425.64) | 574.23 (388.11 to 841.0) | 369.92 (272.62 to 508.79) | 221.92 (124.51 to 340.61) | 522.85 (388.37 to 707.41) | -13.44% (-34.54% to 17.29%) | -20.49% (-44.12% to 12.78%) | -8.95% (-36.72% to 32.68%) |
|  | Environmental/occupational risks | DALYs | All age number | 804.57 (460.71 to 1296.81) | 176.44 (84.42 to 327.15) | 628.13 (355.22 to 1028.54) | 2532.31 (1508.64 to 3962.98) | 682.73 (331.73 to 1169.6) | 1849.58 (1098.04 to 2783.32) | 214.74% (113.86% to 383.24%) | 286.95% (110.37% to 605.7%) | 194.46% (87.57% to 373.76%) |
|  |  |  | Age-standardized rate (per 100,000) | 59.63 (33.17 to 97.52) | 26.9 (12.73 to 50.07) | 91.92 (51.24 to 150.36) | 61.53 (36.15 to 96.14) | 33.37 (16.29 to 57.37) | 90.35 (52.62 to 138.47) | 3.18% (-29.57% to 60.15%) | 24.07% (-32.06% to 121.97%) | -1.71% (-36.93% to 55.7%) |
|  |  | Deaths | All age number | 27.23 (15.24 to 44.46) | 5.98 (2.82 to 11.14) | 21.26 (12.01 to 34.98) | 95.36 (55.08 to 149.64) | 26.57 (13.04 to 46.02) | 68.79 (39.53 to 106.41) | 250.17% (136.2% to 444.49%) | 344.62% (146.1% to 690.96%) | 223.62% (105.47% to 420.66%) |
|  |  |  | Age-standardized rate (per 100,000) | 2.37 (1.25 to 3.99) | 1.1 (0.52 to 2.07) | 3.68 (1.99 to 6.14) | 2.5 (1.43 to 3.94) | 1.41 (0.69 to 2.44) | 3.62 (2.07 to 5.7) | 5.71% (-28.73% to 62.93%) | 27.92% (-29.35% to 127.89%) | -1.47% (-36.64% to 54.41%) |
|  |  | YLDs | All age number | 7.03 (3.67 to 11.51) | 1.47 (0.66 to 2.94) | 5.56 (2.77 to 9.4) | 24.34 (13.13 to 39.85) | 6.3 (3.04 to 11.38) | 18.04 (9.73 to 28.94) | 246.08% (129.06% to 440.88%) | 328.09% (133.62% to 656.05%) | 224.38% (105.2% to 455.66%) |
|  |  |  | Age-standardized rate (per 100,000) | 0.55 (0.28 to 0.89) | 0.24 (0.11 to 0.48) | 0.86 (0.42 to 1.46) | 0.61 (0.33 to 1.01) | 0.32 (0.15 to 0.58) | 0.9 (0.49 to 1.45) | 9.41% (-26.54% to 74.53%) | 29.72% (-29.82% to 130.15%) | 4.42% (-32.1% to 76.85%) |
|  |  | YLLs | All age number | 797.53 (455.99 to 1287.0) | 174.97 (83.65 to 324.05) | 622.57 (352.5 to 1018.73) | 2507.97 (1492.72 to 3920.96) | 676.43 (328.03 to 1158.91) | 1831.55 (1087.12 to 2761.58) | 214.47% (113.53% to 382.67%) | 286.6% (110.15% to 605.37%) | 194.19% (87.23% to 372.95%) |
|  |  |  | Age-standardized rate (per 100,000) | 59.08 (32.81 to 96.56) | 26.66 (12.59 to 49.6) | 91.06 (50.83 to 148.63) | 60.92 (35.76 to 95.09) | 33.06 (16.1 to 56.82) | 89.45 (52.14 to 137.25) | 3.13% (-29.57% to 59.97%) | 24.02% (-32.07% to 121.92%) | -1.77% (-37.02% to 55.81%) |
|  | Metabolic risks | DALYs | All age number | 836.1 (322.85 to 1333.41) | 490.86 (196.78 to 822.5) | 345.24 (136.21 to 571.37) | 5494.93 (1641.9 to 9151.3) | 3076.43 (851.09 to 5180.49) | 2418.49 (802.65 to 4108.65) | 557.21% (362.35% to 756.4%) | 526.74% (300.75% to 796.67%) | 600.52% (375.87% to 942.2%) |
|  |  |  | Age-standardized rate (per 100,000) | 62.87 (22.71 to 101.4) | 74.92 (28.24 to 126.71) | 51.22 (18.52 to 86.13) | 135.64 (40.73 to 227.1) | 149.41 (41.7 to 251.49) | 121.37 (39.52 to 206.52) | 115.74% (58.12% to 177.08%) | 99.41% (31.98% to 180.09%) | 136.97% (63.33% to 249.08%) |
|  |  | Deaths | All age number | 28.23 (10.06 to 45.91) | 16.56 (6.22 to 27.88) | 11.67 (4.18 to 19.72) | 207.6 (60.67 to 351.15) | 115.19 (31.61 to 194.59) | 92.41 (28.75 to 158.07) | 635.3% (430.68% to 838.33%) | 595.57% (361.11% to 855.51%) | 691.66% (440.11% to 1063.56%) |
|  |  |  | Age-standardized rate (per 100,000) | 2.53 (0.86 to 4.14) | 2.96 (1.06 to 5.15) | 2.1 (0.71 to 3.54) | 5.53 (1.62 to 9.38) | 6.02 (1.66 to 10.12) | 5.02 (1.54 to 8.62) | 118.04% (60.58% to 174.79%) | 103.15% (40.56% to 181.24%) | 139.03% (70.95% to 247.74%) |
|  |  | YLDs | All age number | 22.12 (6.92 to 40.03) | 15.59 (4.1 to 30.05) | 6.53 (2.37 to 11.59) | 249.31 (63.89 to 448.24) | 181.74 (34.11 to 331.24) | 67.57 (26.31 to 122.69) | 1027.26% (678.31% to 1411.26%) | 1066.09% (651.14% to 1643.11%) | 934.62% (623.48% to 1440.25%) |
|  |  |  | Age-standardized rate (per 100,000) | 1.74 (0.52 to 3.19) | 2.48 (0.62 to 4.8) | 1.04 (0.36 to 1.88) | 6.08 (1.56 to 10.99) | 8.64 (1.62 to 15.72) | 3.43 (1.32 to 6.2) | 248.92% (152.0% to 359.67%) | 249.18% (133.04% to 418.45%) | 229.33% (133.94% to 390.12%) |
|  |  | YLLs | All age number | 813.98 (316.05 to 1303.19) | 475.27 (192.49 to 795.92) | 338.71 (133.32 to 558.09) | 5245.62 (1586.94 to 8748.96) | 2894.69 (812.44 to 4866.35) | 2350.93 (773.77 to 3992.52) | 544.44% (353.79% to 739.33%) | 509.06% (289.58% to 773.16%) | 594.08% (371.07% to 932.05%) |
|  |  |  | Age-standardized rate (per 100,000) | 61.13 (22.21 to 98.88) | 72.45 (27.68 to 122.06) | 50.18 (18.1 to 84.58) | 129.57 (39.37 to 216.92) | 140.76 (39.75 to 237.45) | 117.94 (38.12 to 200.66) | 111.95% (55.61% to 172.47%) | 94.3% (29.13% to 173.97%) | 135.05% (62.08% to 246.25%) |
| North Khorasan | All risk factors | DALYs | All age number | 2258.54 (1656.72 to 3229.05) | 681.97 (440.81 to 983.92) | 1576.57 (1107.57 to 2284.34) | 4746.13 (3567.5 to 6146.69) | 1716.73 (1108.73 to 2450.1) | 3029.39 (2290.52 to 3934.83) | 110.14% (61.01% to 180.07%) | 151.73% (72.25% to 260.33%) | 92.15% (35.62% to 171.99%) |
|  |  |  | Age-standardized rate (per 100,000) | 716.83 (527.16 to 1027.64) | 435.6 (281.04 to 627.78) | 959.19 (674.08 to 1399.68) | 606.08 (455.02 to 787.61) | 425.22 (271.65 to 602.84) | 792.81 (601.13 to 1030.65) | -15.45% (-35.62% to 12.48%) | -2.38% (-32.9% to 36.43%) | -17.35% (-40.81% to 15.82%) |
|  |  | Deaths | All age number | 75.07 (54.92 to 107.83) | 20.56 (13.32 to 29.79) | 54.51 (38.3 to 79.51) | 176.42 (131.7 to 228.34) | 60.08 (38.18 to 84.24) | 116.34 (88.45 to 150.88) | 135.02% (79.19% to 214.53%) | 192.25% (101.63% to 316.45%) | 113.43% (51.54% to 201.1%) |
|  |  |  | Age-standardized rate (per 100,000) | 28.07 (20.46 to 40.55) | 16.09 (10.58 to 23.72) | 38.77 (27.57 to 55.93) | 24.55 (18.29 to 31.72) | 16.54 (10.53 to 23.06) | 32.59 (24.74 to 42.35) | -12.54% (-33.89% to 15.33%) | 2.79% (-28.28% to 42.73%) | -15.93% (-38.57% to 16.8%) |
|  |  | YLDs | All age number | 31.05 (19.8 to 44.46) | 12.03 (6.72 to 18.26) | 19.01 (12.24 to 28.81) | 113.95 (70.48 to 165.84) | 60.68 (31.02 to 98.49) | 53.27 (35.72 to 73.66) | 267.06% (166.65% to 383.6%) | 404.25% (246.21% to 616.61%) | 180.22% (100.22% to 289.16%) |
|  |  |  | Age-standardized rate (per 100,000) | 10.19 (6.54 to 14.59) | 7.81 (4.32 to 11.75) | 12.19 (7.88 to 18.44) | 14.41 (9.16 to 20.88) | 14.6 (7.53 to 23.21) | 14.22 (9.5 to 19.51) | 41.42% (4.39% to 84.89%) | 87.05% (29.67% to 161.05%) | 16.67% (-17.13% to 58.33%) |
|  |  | YLLs | All age number | 2227.49 (1629.55 to 3187.76) | 669.94 (433.85 to 969.24) | 1557.56 (1092.49 to 2259.24) | 4632.17 (3495.14 to 6023.06) | 1656.05 (1077.49 to 2360.11) | 2976.12 (2255.68 to 3872.82) | 107.95% (59.07% to 178.17%) | 147.19% (69.07% to 254.58%) | 91.08% (34.98% to 170.45%) |
|  |  |  | Age-standardized rate (per 100,000) | 706.64 (519.49 to 1014.94) | 427.79 (275.39 to 617.9) | 947.01 (664.85 to 1383.84) | 591.67 (446.56 to 770.87) | 410.61 (263.68 to 580.07) | 778.59 (590.11 to 1012.67) | -16.27% (-36.46% to 11.91%) | -4.02% (-34.18% to 34.42%) | -17.78% (-41.09% to 15.3%) |
|  | Behavioral risks | DALYs | All age number | 2006.57 (1424.99 to 2961.79) | 560.98 (340.44 to 850.71) | 1445.59 (994.34 to 2123.34) | 3615.64 (2685.25 to 4801.53) | 1080.21 (639.07 to 1604.34) | 2535.43 (1899.37 to 3337.57) | 80.19% (37.76% to 138.39%) | 92.56% (36.14% to 173.47%) | 75.39% (25.13% to 143.99%) |
|  |  |  | Age-standardized rate (per 100,000) | 636.82 (451.71 to 945.84) | 355.29 (214.13 to 541.99) | 879.41 (607.4 to 1303.46) | 460.67 (343.37 to 611.3) | 262.3 (156.66 to 389.05) | 665.33 (501.61 to 877.24) | -27.66% (-44.62% to -3.8%) | -26.17% (-46.72% to 2.97%) | -24.34% (-45.61% to 4.98%) |
|  |  | Deaths | All age number | 66.86 (47.54 to 99.56) | 16.74 (10.14 to 25.57) | 50.13 (34.46 to 73.88) | 134.27 (100.1 to 178.3) | 36.55 (22.25 to 53.45) | 97.72 (73.87 to 128.6) | 100.81% (53.73% to 166.09%) | 118.39% (57.63% to 201.24%) | 94.95% (39.92% to 170.63%) |
|  |  |  | Age-standardized rate (per 100,000) | 24.86 (17.67 to 36.93) | 12.96 (7.81 to 20.05) | 35.48 (24.78 to 52.74) | 18.64 (13.99 to 24.75) | 9.9 (6.08 to 14.33) | 27.41 (20.61 to 36.25) | -25.02% (-42.45% to -1.58%) | -23.59% (-44.37% to 5.24%) | -22.75% (-43.73% to 6.39%) |
|  |  | YLDs | All age number | 27.1 (16.93 to 40.31) | 9.64 (4.91 to 15.4) | 17.46 (11.16 to 27.0) | 82.33 (47.36 to 121.75) | 38.01 (15.66 to 66.68) | 44.32 (29.3 to 61.2) | 203.76% (117.38% to 299.02%) | 294.09% (148.07% to 476.41%) | 153.86% (81.76% to 254.49%) |
|  |  |  | Age-standardized rate (per 100,000) | 8.87 (5.56 to 13.14) | 6.14 (3.13 to 9.82) | 11.16 (7.16 to 17.09) | 10.33 (6.1 to 15.11) | 8.86 (3.78 to 15.23) | 11.87 (7.84 to 16.36) | 16.52% (-15.38% to 51.38%) | 44.3% (-7.2% to 109.14%) | 6.32% (-23.81% to 43.91%) |
|  |  | YLLs | All age number | 1979.47 (1403.65 to 2922.45) | 551.34 (334.86 to 836.78) | 1428.13 (982.68 to 2099.15) | 3533.31 (2637.45 to 4689.43) | 1042.2 (623.68 to 1539.42) | 2491.1 (1866.2 to 3283.68) | 78.5% (36.47% to 137.07%) | 89.03% (33.67% to 167.93%) | 74.43% (24.44% to 142.94%) |
|  |  |  | Age-standardized rate (per 100,000) | 627.95 (445.45 to 933.07) | 349.15 (210.36 to 533.66) | 868.25 (600.4 to 1288.86) | 450.34 (337.03 to 599.24) | 253.44 (152.91 to 371.92) | 653.46 (491.91 to 862.41) | -28.28% (-45.08% to -4.42%) | -27.41% (-47.83% to 0.8%) | -24.74% (-45.89% to 4.48%) |
|  | Environmental/occupational risks | DALYs | All age number | 236.39 (135.02 to 382.77) | 39.44 (19.03 to 71.55) | 196.94 (112.74 to 326.51) | 585.86 (356.49 to 867.12) | 161.64 (82.6 to 269.05) | 424.22 (267.29 to 635.2) | 147.84% (57.55% to 291.18%) | 309.81% (109.7% to 691.38%) | 115.4% (33.25% to 256.48%) |
|  |  |  | Age-standardized rate (per 100,000) | 74.58 (42.78 to 122.6) | 26.45 (12.62 to 47.68) | 117.12 (66.84 to 195.1) | 74.58 (45.41 to 110.66) | 40.98 (20.81 to 67.83) | 109.18 (68.3 to 163.66) | -0.0% (-37.67% to 58.38%) | 54.95% (-18.91% to 206.34%) | -6.78% (-43.05% to 53.21%) |
|  |  | Deaths | All age number | 7.92 (4.54 to 13.06) | 1.26 (0.6 to 2.29) | 6.66 (3.78 to 11.08) | 21.89 (13.23 to 32.35) | 6.06 (3.1 to 10.07) | 15.83 (9.92 to 23.66) | 176.4% (72.79% to 336.11%) | 381.12% (149.21% to 850.5%) | 137.66% (46.57% to 292.53%) |
|  |  |  | Age-standardized rate (per 100,000) | 2.96 (1.7 to 4.93) | 1.06 (0.51 to 1.94) | 4.67 (2.68 to 7.86) | 3.03 (1.82 to 4.53) | 1.71 (0.87 to 2.83) | 4.36 (2.72 to 6.53) | 2.65% (-36.01% to 61.35%) | 61.33% (-14.08% to 225.37%) | -6.56% (-43.16% to 54.72%) |
|  |  | YLDs | All age number | 2.04 (1.12 to 3.41) | 0.32 (0.15 to 0.59) | 1.72 (0.92 to 2.87) | 5.48 (2.92 to 8.68) | 1.43 (0.63 to 2.45) | 4.05 (2.26 to 6.41) | 168.36% (70.75% to 335.52%) | 343.61% (117.54% to 771.57%) | 135.55% (44.41% to 290.56%) |
|  |  |  | Age-standardized rate (per 100,000) | 0.69 (0.38 to 1.16) | 0.24 (0.11 to 0.44) | 1.09 (0.59 to 1.82) | 0.72 (0.38 to 1.15) | 0.38 (0.16 to 0.65) | 1.07 (0.59 to 1.7) | 4.54% (-34.16% to 64.17%) | 57.48% (-22.58% to 211.09%) | -1.79% (-39.03% to 61.4%) |
|  |  | YLLs | All age number | 234.35 (134.0 to 379.33) | 39.12 (18.88 to 71.02) | 195.22 (111.54 to 323.93) | 580.38 (353.75 to 857.89) | 160.21 (81.89 to 266.7) | 420.17 (264.55 to 629.99) | 147.66% (57.39% to 290.9%) | 309.53% (109.7% to 690.43%) | 115.23% (32.94% to 256.12%) |
|  |  |  | Age-standardized rate (per 100,000) | 73.89 (42.42 to 121.4) | 26.21 (12.53 to 47.23) | 116.03 (66.05 to 193.44) | 73.86 (45.06 to 109.55) | 40.61 (20.63 to 67.2) | 108.11 (67.57 to 162.17) | -0.05% (-37.72% to 58.37%) | 54.92% (-18.96% to 206.27%) | -6.82% (-43.1% to 53.18%) |
|  | Metabolic risks | DALYs | All age number | 204.28 (87.38 to 338.22) | 109.97 (48.62 to 177.9) | 94.31 (36.12 to 167.07) | 1075.45 (347.84 to 1772.46) | 605.89 (195.92 to 996.5) | 469.57 (150.14 to 788.88) | 426.46% (270.32% to 574.09%) | 450.95% (240.08% to 681.35%) | 397.9% (245.43% to 598.27%) |
|  |  |  | Age-standardized rate (per 100,000) | 65.52 (26.38 to 109.68) | 73.68 (30.74 to 122.2) | 58.03 (20.63 to 104.24) | 139.23 (43.49 to 231.18) | 155.63 (49.13 to 257.21) | 122.57 (37.95 to 208.49) | 112.49% (55.15% to 170.54%) | 111.23% (39.97% to 192.43%) | 111.21% (49.88% to 196.89%) |
|  |  | Deaths | All age number | 6.76 (2.68 to 11.4) | 3.51 (1.45 to 5.79) | 3.25 (1.12 to 5.87) | 40.33 (12.07 to 67.96) | 22.42 (6.87 to 37.33) | 17.9 (5.26 to 30.81) | 496.23% (334.14% to 656.6%) | 538.34% (313.24% to 787.59%) | 450.74% (280.3% to 670.07%) |
|  |  |  | Age-standardized rate (per 100,000) | 2.63 (0.92 to 4.48) | 2.87 (1.12 to 4.82) | 2.4 (0.74 to 4.39) | 5.66 (1.66 to 9.6) | 6.32 (1.93 to 10.53) | 5.02 (1.42 to 8.65) | 115.63% (61.71% to 170.27%) | 120.51% (52.35% to 198.1%) | 108.81% (50.77% to 189.33%) |
|  |  | YLDs | All age number | 4.02 (1.46 to 6.87) | 2.57 (0.94 to 4.45) | 1.45 (0.53 to 2.64) | 35.64 (11.84 to 60.97) | 25.22 (8.12 to 43.6) | 10.41 (3.9 to 18.48) | 787.32% (534.02% to 1120.54%) | 881.95% (537.18% to 1333.42%) | 619.4% (396.43% to 923.62%) |
|  |  |  | Age-standardized rate (per 100,000) | 1.35 (0.45 to 2.33) | 1.8 (0.61 to 3.18) | 0.96 (0.34 to 1.73) | 4.62 (1.47 to 8.0) | 6.41 (1.99 to 11.18) | 2.76 (0.98 to 4.9) | 241.74% (150.33% to 359.22%) | 255.53% (138.86% to 404.8%) | 188.55% (103.8% to 314.38%) |
|  |  | YLLs | All age number | 200.27 (85.64 to 331.0) | 107.4 (47.63 to 173.82) | 92.86 (35.5 to 164.29) | 1039.82 (334.56 to 1716.78) | 580.66 (185.93 to 960.35) | 459.15 (147.11 to 770.61) | 419.22% (267.19% to 565.97%) | 440.64% (234.79% to 667.28%) | 394.45% (242.28% to 595.38%) |
|  |  |  | Age-standardized rate (per 100,000) | 64.17 (25.92 to 107.21) | 71.87 (30.11 to 118.68) | 57.07 (20.19 to 102.51) | 134.61 (41.86 to 223.68) | 149.22 (47.03 to 248.0) | 119.81 (37.19 to 202.97) | 109.77% (53.21% to 167.17%) | 107.61% (36.94% to 187.66%) | 109.91% (49.02% to 194.87%) |
| Qazvin | All risk factors | DALYs | All age number | 2395.97 (1764.23 to 3339.45) | 756.34 (473.06 to 1107.06) | 1639.63 (1165.8 to 2418.82) | 7056.5 (5336.34 to 8997.16) | 2215.97 (1365.81 to 3141.31) | 4840.52 (3713.67 to 5982.93) | 194.52% (123.66% to 288.33%) | 192.99% (101.63% to 323.71%) | 195.22% (109.81% to 315.89%) |
|  |  |  | Age-standardized rate (per 100,000) | 511.84 (378.67 to 714.77) | 323.99 (202.99 to 476.26) | 689.13 (491.16 to 1012.45) | 552.5 (418.29 to 709.91) | 329.85 (200.26 to 470.1) | 789.12 (612.77 to 981.65) | 7.94% (-17.38% to 42.49%) | 1.81% (-29.3% to 48.43%) | 14.51% (-18.62% to 61.75%) |
|  |  | Deaths | All age number | 83.18 (61.4 to 116.19) | 24.16 (15.24 to 35.34) | 59.02 (42.05 to 87.08) | 262.61 (201.74 to 338.29) | 77.23 (46.46 to 109.35) | 185.37 (144.61 to 231.54) | 215.73% (140.76% to 313.7%) | 219.72% (123.08% to 361.1%) | 214.1% (122.69% to 343.01%) |
|  |  |  | Age-standardized rate (per 100,000) | 20.27 (14.88 to 28.49) | 11.97 (7.49 to 17.43) | 28.72 (20.28 to 41.83) | 23.02 (17.57 to 29.88) | 12.79 (7.72 to 18.14) | 34.23 (26.79 to 43.51) | 13.57% (-13.48% to 48.36%) | 6.82% (-24.52% to 53.58%) | 19.17% (-13.76% to 65.93%) |
|  |  | YLDs | All age number | 39.52 (24.65 to 56.3) | 16.83 (8.43 to 27.67) | 22.68 (14.41 to 33.64) | 205.66 (121.73 to 305.87) | 105.18 (48.77 to 179.66) | 100.48 (67.89 to 144.79) | 420.45% (287.15% to 565.3%) | 524.87% (321.94% to 772.31%) | 342.96% (213.26% to 533.6%) |
|  |  |  | Age-standardized rate (per 100,000) | 8.62 (5.44 to 12.09) | 7.26 (3.69 to 11.73) | 9.95 (6.5 to 14.64) | 15.8 (9.44 to 23.4) | 15.14 (7.08 to 25.7) | 16.84 (11.44 to 23.98) | 83.23% (36.03% to 135.04%) | 108.45% (42.47% to 189.38%) | 69.28% (21.02% to 138.38%) |
|  |  | YLLs | All age number | 2356.45 (1732.73 to 3290.04) | 739.51 (463.05 to 1083.83) | 1616.94 (1149.29 to 2389.62) | 6850.84 (5192.43 to 8685.25) | 2110.8 (1309.0 to 2976.84) | 4740.04 (3634.4 to 5864.92) | 190.73% (121.13% to 284.01%) | 185.43% (97.5% to 314.17%) | 193.15% (107.83% to 312.85%) |
|  |  |  | Age-standardized rate (per 100,000) | 503.22 (372.66 to 703.2) | 316.73 (199.26 to 464.88) | 679.18 (483.76 to 997.48) | 536.7 (410.3 to 688.89) | 314.72 (191.96 to 446.47) | 772.27 (598.91 to 961.65) | 6.65% (-18.29% to 41.02%) | -0.64% (-30.87% to 45.17%) | 13.71% (-19.16% to 60.79%) |
|  | Behavioral risks | DALYs | All age number | 2058.05 (1462.66 to 2985.13) | 590.57 (332.52 to 931.47) | 1467.48 (1020.77 to 2169.82) | 5376.85 (3995.32 to 7029.87) | 1371.74 (758.61 to 2078.87) | 4005.11 (3036.07 to 5174.66) | 161.26% (97.59% to 245.38%) | 132.27% (59.1% to 239.89%) | 172.92% (93.08% to 282.3%) |
|  |  |  | Age-standardized rate (per 100,000) | 441.03 (318.32 to 636.54) | 252.65 (143.02 to 396.1) | 618.22 (433.42 to 919.81) | 419.53 (314.38 to 554.16) | 198.45 (112.66 to 300.73) | 653.98 (496.77 to 843.45) | -4.87% (-27.96% to 25.01%) | -21.45% (-45.01% to 15.22%) | 5.78% (-24.13% to 47.89%) |
|  |  | Deaths | All age number | 71.77 (51.85 to 104.54) | 18.68 (10.71 to 29.24) | 53.1 (37.02 to 79.06) | 199.54 (151.27 to 264.08) | 45.7 (25.96 to 69.25) | 153.84 (117.57 to 200.41) | 178.02% (110.59% to 264.83%) | 144.69% (72.31% to 262.3%) | 189.74% (109.4% to 303.38%) |
|  |  |  | Age-standardized rate (per 100,000) | 17.46 (12.65 to 25.49) | 9.24 (5.27 to 14.42) | 25.78 (17.88 to 38.47) | 17.42 (13.19 to 23.29) | 7.41 (4.25 to 11.15) | 28.37 (21.38 to 37.22) | -0.2% (-22.63% to 29.75%) | -19.8% (-42.3% to 14.73%) | 10.06% (-19.56% to 52.27%) |
|  |  | YLDs | All age number | 33.87 (21.0 to 49.27) | 13.29 (5.84 to 22.77) | 20.58 (12.95 to 31.1) | 152.15 (87.14 to 234.03) | 69.16 (24.13 to 128.25) | 83.0 (54.39 to 117.49) | 349.28% (229.46% to 480.06%) | 420.54% (219.24% to 631.04%) | 303.28% (185.72% to 467.16%) |
|  |  |  | Age-standardized rate (per 100,000) | 7.38 (4.57 to 10.62) | 5.66 (2.48 to 9.7) | 9.02 (5.83 to 13.57) | 11.54 (6.92 to 17.63) | 9.52 (3.42 to 17.46) | 13.94 (9.19 to 19.28) | 56.38% (18.13% to 99.63%) | 68.26% (6.87% to 134.29%) | 54.59% (11.54% to 115.91%) |
|  |  | YLLs | All age number | 2024.18 (1441.13 to 2938.47) | 577.28 (326.6 to 910.51) | 1446.9 (1007.43 to 2141.87) | 5224.7 (3891.0 to 6842.95) | 1302.58 (728.88 to 1955.93) | 3922.11 (2964.52 to 5069.51) | 158.11% (95.59% to 241.45%) | 125.64% (54.72% to 231.85%) | 171.07% (91.71% to 280.03%) |
|  |  |  | Age-standardized rate (per 100,000) | 433.65 (313.2 to 626.76) | 246.99 (140.67 to 386.44) | 609.2 (425.6 to 907.79) | 408.0 (306.35 to 540.01) | 188.93 (107.88 to 286.18) | 640.04 (484.39 to 823.75) | -5.92% (-28.75% to 23.26%) | -23.51% (-46.39% to 12.75%) | 5.06% (-24.67% to 46.88%) |
|  | Environmental/occupational risks | DALYs | All age number | 308.52 (189.5 to 460.05) | 52.43 (26.46 to 92.08) | 256.09 (157.0 to 385.88) | 922.79 (624.28 to 1333.75) | 185.85 (109.63 to 293.15) | 736.94 (489.94 to 1044.68) | 199.1% (100.93% to 363.82%) | 254.44% (104.4% to 548.37%) | 187.77% (80.76% to 358.51%) |
|  |  |  | Age-standardized rate (per 100,000) | 65.16 (39.74 to 97.53) | 23.13 (11.66 to 40.31) | 104.75 (64.94 to 159.44) | 71.94 (48.42 to 104.46) | 28.26 (16.65 to 44.85) | 117.42 (78.5 to 168.04) | 10.4% (-25.84% to 69.9%) | 22.21% (-28.29% to 122.55%) | 12.1% (-28.37% to 75.33%) |
|  |  | Deaths | All age number | 10.79 (6.59 to 16.04) | 1.79 (0.9 to 3.12) | 9.01 (5.54 to 13.7) | 34.49 (22.94 to 50.32) | 7.01 (4.15 to 11.17) | 27.48 (18.3 to 39.69) | 219.59% (114.8% to 382.89%) | 292.19% (130.23% to 605.42%) | 205.18% (94.47% to 381.06%) |
|  |  |  | Age-standardized rate (per 100,000) | 2.58 (1.57 to 3.86) | 0.93 (0.47 to 1.59) | 4.24 (2.63 to 6.5) | 3.0 (1.99 to 4.41) | 1.19 (0.7 to 1.9) | 4.95 (3.26 to 7.2) | 16.06% (-21.82% to 76.87%) | 28.19% (-25.83% to 125.28%) | 16.66% (-25.61% to 77.05%) |
|  |  | YLDs | All age number | 2.69 (1.57 to 4.25) | 0.44 (0.2 to 0.8) | 2.25 (1.26 to 3.62) | 8.7 (5.14 to 13.13) | 1.67 (0.87 to 2.83) | 7.03 (4.08 to 10.88) | 222.96% (115.23% to 433.44%) | 278.26% (120.15% to 569.0%) | 212.09% (97.8% to 434.84%) |
|  |  |  | Age-standardized rate (per 100,000) | 0.6 (0.34 to 0.93) | 0.21 (0.09 to 0.38) | 0.97 (0.56 to 1.53) | 0.71 (0.42 to 1.08) | 0.27 (0.14 to 0.46) | 1.17 (0.68 to 1.77) | 17.67% (-21.84% to 93.06%) | 26.17% (-27.02% to 123.71%) | 19.87% (-23.13% to 97.74%) |
|  |  | YLLs | All age number | 305.83 (187.88 to 456.07) | 51.99 (26.24 to 91.38) | 253.84 (155.56 to 382.62) | 914.09 (618.37 to 1321.65) | 184.17 (108.95 to 290.59) | 729.91 (486.14 to 1034.72) | 198.89% (100.66% to 363.4%) | 254.23% (104.21% to 548.06%) | 187.55% (80.67% to 358.44%) |
|  |  |  | Age-standardized rate (per 100,000) | 64.56 (39.41 to 96.59) | 22.91 (11.55 to 39.91) | 103.77 (64.19 to 158.12) | 71.24 (48.0 to 103.38) | 27.99 (16.52 to 44.44) | 116.25 (77.81 to 166.58) | 10.34% (-25.9% to 69.85%) | 22.18% (-28.37% to 122.55%) | 12.03% (-28.39% to 75.37%) |
|  | Metabolic risks | DALYs | All age number | 268.59 (108.91 to 437.94) | 152.02 (59.47 to 249.75) | 116.57 (42.07 to 198.07) | 1666.37 (527.58 to 2744.78) | 839.3 (262.83 to 1430.09) | 827.07 (282.38 to 1401.17) | 520.41% (336.57% to 712.02%) | 452.11% (252.34% to 692.84%) | 609.48% (366.56% to 918.8%) |
|  |  |  | Age-standardized rate (per 100,000) | 56.5 (22.04 to 93.21) | 65.64 (24.28 to 109.55) | 48.17 (16.59 to 82.41) | 132.99 (40.67 to 221.19) | 131.47 (40.05 to 226.52) | 135.16 (42.55 to 230.37) | 135.39% (67.74% to 205.01%) | 100.29% (30.89% to 187.95%) | 180.59% (87.47% to 296.66%) |
|  |  | Deaths | All age number | 9.1 (3.43 to 15.18) | 5.05 (1.84 to 8.48) | 4.06 (1.34 to 7.09) | 62.97 (18.73 to 105.71) | 31.44 (9.54 to 54.23) | 31.53 (9.44 to 54.22) | 591.63% (391.08% to 786.91%) | 523.2% (308.08% to 802.92%) | 676.67% (423.38% to 1010.46%) |
|  |  |  | Age-standardized rate (per 100,000) | 2.23 (0.82 to 3.75) | 2.5 (0.91 to 4.24) | 1.98 (0.64 to 3.44) | 5.61 (1.63 to 9.51) | 5.37 (1.61 to 9.26) | 5.88 (1.7 to 10.24) | 151.61% (80.25% to 220.12%) | 115.32% (44.43% to 202.29%) | 196.17% (104.38% to 305.22%) |
|  |  | YLDs | All age number | 5.87 (1.87 to 10.28) | 3.91 (1.13 to 7.3) | 1.95 (0.72 to 3.53) | 62.82 (17.18 to 112.57) | 41.44 (9.25 to 77.14) | 21.37 (8.01 to 37.91) | 970.71% (658.13% to 1364.55%) | 958.72% (578.43% to 1547.0%) | 994.74% (655.54% to 1443.9%) |
|  |  |  | Age-standardized rate (per 100,000) | 1.29 (0.39 to 2.26) | 1.77 (0.47 to 3.3) | 0.86 (0.3 to 1.56) | 5.04 (1.39 to 9.13) | 6.49 (1.44 to 12.12) | 3.58 (1.3 to 6.41) | 290.15% (180.4% to 418.5%) | 266.65% (140.72% to 461.93%) | 316.86% (186.31% to 482.45%) |
|  |  | YLLs | All age number | 262.73 (107.07 to 428.63) | 148.1 (58.57 to 244.3) | 114.62 (41.3 to 195.03) | 1603.55 (504.83 to 2640.51) | 797.85 (251.87 to 1367.62) | 805.7 (274.07 to 1367.7) | 510.35% (329.05% to 697.39%) | 438.71% (244.04% to 676.55%) | 602.92% (361.83% to 910.04%) |
|  |  |  | Age-standardized rate (per 100,000) | 55.21 (21.64 to 91.02) | 63.87 (23.78 to 106.52) | 47.31 (16.29 to 81.01) | 127.96 (38.95 to 213.29) | 124.99 (38.61 to 216.03) | 131.58 (41.25 to 224.77) | 131.77% (64.99% to 199.96%) | 95.68% (27.67% to 181.32%) | 178.12% (85.53% to 293.69%) |
| Qom | All risk factors | DALYs | All age number | 2331.56 (1733.19 to 3127.58) | 816.01 (517.13 to 1137.86) | 1515.55 (1111.02 to 2135.51) | 6823.95 (5062.82 to 8864.49) | 2662.96 (1587.09 to 3792.1) | 4160.98 (3208.56 to 5292.65) | 192.68% (122.72% to 287.14%) | 226.34% (129.55% to 358.42%) | 174.55% (87.16% to 295.65%) |
|  |  |  | Age-standardized rate (per 100,000) | 693.37 (514.54 to 927.4) | 495.77 (309.86 to 678.06) | 877.97 (646.39 to 1227.12) | 563.82 (421.43 to 729.63) | 452.02 (269.15 to 640.49) | 676.59 (520.86 to 864.23) | -18.68% (-38.13% to 8.13%) | -8.83% (-35.29% to 26.76%) | -22.94% (-47.09% to 11.92%) |
|  |  | Deaths | All age number | 79.89 (59.45 to 107.18) | 26.32 (16.52 to 35.99) | 53.57 (39.62 to 75.06) | 252.56 (188.17 to 325.87) | 96.22 (57.66 to 134.94) | 156.34 (119.65 to 200.7) | 216.14% (140.15% to 318.63%) | 265.59% (162.31% to 408.33%) | 191.84% (101.95% to 323.42%) |
|  |  |  | Age-standardized rate (per 100,000) | 28.09 (21.07 to 37.53) | 19.7 (12.42 to 26.69) | 36.2 (26.81 to 50.17) | 23.77 (17.61 to 30.65) | 19.33 (11.46 to 27.02) | 28.49 (21.8 to 36.59) | -15.38% (-35.23% to 9.65%) | -1.9% (-28.87% to 31.1%) | -21.3% (-44.88% to 12.38%) |
|  |  | YLDs | All age number | 38.86 (23.75 to 55.98) | 18.53 (9.25 to 30.83) | 20.33 (13.68 to 29.38) | 221.88 (128.04 to 346.36) | 132.93 (58.02 to 221.52) | 88.95 (59.65 to 127.82) | 470.95% (336.27% to 642.84%) | 617.46% (390.02% to 931.06%) | 337.46% (201.1% to 537.96%) |
|  |  |  | Age-standardized rate (per 100,000) | 11.78 (7.28 to 16.71) | 11.2 (5.58 to 17.89) | 12.38 (8.37 to 17.87) | 17.98 (10.59 to 27.36) | 21.34 (9.4 to 35.29) | 14.89 (10.03 to 21.26) | 52.56% (16.55% to 98.25%) | 90.53% (32.34% to 165.85%) | 20.23% (-17.08% to 73.4%) |
|  |  | YLLs | All age number | 2292.7 (1709.9 to 3084.42) | 797.49 (508.07 to 1107.94) | 1495.22 (1095.03 to 2111.42) | 6602.07 (4923.59 to 8536.08) | 2530.04 (1531.73 to 3567.99) | 4072.03 (3126.13 to 5186.55) | 187.96% (119.65% to 280.29%) | 217.25% (125.17% to 346.55%) | 172.34% (85.63% to 292.76%) |
|  |  |  | Age-standardized rate (per 100,000) | 681.58 (507.26 to 912.45) | 484.57 (305.16 to 663.36) | 865.59 (636.82 to 1211.36) | 545.84 (409.21 to 706.57) | 430.67 (260.04 to 604.58) | 661.7 (508.55 to 843.14) | -19.92% (-39.14% to 6.3%) | -11.12% (-36.75% to 23.97%) | -23.56% (-47.53% to 10.9%) |
|  | Behavioral risks | DALYs | All age number | 1957.02 (1428.0 to 2705.49) | 633.25 (371.24 to 914.55) | 1323.77 (954.33 to 1897.96) | 4904.84 (3585.27 to 6538.16) | 1623.86 (913.33 to 2468.59) | 3280.98 (2440.14 to 4360.68) | 150.63% (90.26% to 230.4%) | 156.43% (84.74% to 257.42%) | 147.85% (67.53% to 262.78%) |
|  |  |  | Age-standardized rate (per 100,000) | 583.36 (426.69 to 809.66) | 381.54 (225.07 to 547.52) | 771.44 (557.32 to 1106.35) | 402.13 (296.83 to 533.65) | 266.28 (152.18 to 396.94) | 535.62 (397.94 to 711.77) | -31.07% (-47.33% to -9.25%) | -30.21% (-48.31% to -4.48%) | -30.57% (-52.66% to 1.16%) |
|  |  | Deaths | All age number | 67.22 (49.11 to 94.26) | 20.08 (11.97 to 28.81) | 47.14 (34.23 to 67.6) | 179.83 (134.54 to 238.42) | 55.91 (32.42 to 82.91) | 123.93 (91.55 to 164.63) | 167.54% (102.65% to 254.07%) | 178.41% (108.71% to 276.37%) | 162.91% (79.81% to 283.19%) |
|  |  |  | Age-standardized rate (per 100,000) | 23.53 (17.05 to 32.69) | 14.93 (9.05 to 21.28) | 31.82 (22.94 to 45.86) | 16.8 (12.62 to 22.32) | 10.98 (6.36 to 15.97) | 22.59 (16.59 to 30.02) | -28.61% (-45.3% to -6.05%) | -26.43% (-43.31% to -2.55%) | -29.01% (-51.03% to 1.99%) |
|  |  | YLDs | All age number | 32.54 (19.38 to 48.93) | 14.55 (6.39 to 24.55) | 17.99 (11.92 to 26.09) | 156.19 (81.82 to 251.57) | 86.0 (28.38 to 159.79) | 70.18 (46.49 to 102.09) | 380.03% (248.15% to 538.93%) | 491.25% (261.25% to 744.97%) | 290.11% (165.02% to 475.09%) |
|  |  |  | Age-standardized rate (per 100,000) | 9.81 (6.0 to 14.55) | 8.61 (3.86 to 14.4) | 10.97 (7.31 to 15.84) | 12.38 (6.86 to 19.56) | 13.07 (4.54 to 23.68) | 11.82 (7.91 to 16.83) | 26.21% (-7.13% to 65.67%) | 51.82% (-3.62% to 111.91%) | 7.74% (-26.48% to 58.54%) |
|  |  | YLLs | All age number | 1924.48 (1404.01 to 2665.49) | 618.7 (365.73 to 893.71) | 1305.78 (941.97 to 1874.92) | 4748.65 (3484.02 to 6372.6) | 1537.86 (876.57 to 2326.18) | 3210.79 (2388.25 to 4274.25) | 146.75% (87.91% to 225.45%) | 148.56% (80.49% to 248.06%) | 145.89% (66.28% to 259.79%) |
|  |  |  | Age-standardized rate (per 100,000) | 573.55 (419.63 to 798.33) | 372.93 (221.57 to 534.52) | 760.48 (550.41 to 1092.48) | 389.75 (289.85 to 519.24) | 253.21 (147.15 to 378.01) | 523.81 (388.97 to 697.11) | -32.05% (-47.93% to -10.48%) | -32.1% (-49.4% to -7.69%) | -31.12% (-52.96% to 0.28%) |
|  | Environmental/occupational risks | DALYs | All age number | 317.22 (204.03 to 459.63) | 66.22 (35.53 to 110.68) | 251.0 (156.96 to 371.84) | 921.84 (576.17 to 1353.32) | 259.0 (138.86 to 410.76) | 662.84 (427.68 to 990.74) | 190.6% (87.83% to 322.87%) | 291.12% (121.71% to 607.4%) | 164.08% (58.63% to 316.62%) |
|  |  |  | Age-standardized rate (per 100,000) | 93.07 (60.28 to 136.56) | 41.8 (22.45 to 69.69) | 140.37 (88.38 to 209.58) | 75.78 (47.1 to 111.39) | 45.56 (24.1 to 72.19) | 105.29 (67.57 to 156.67) | -18.58% (-47.55% to 19.03%) | 8.98% (-36.96% to 93.67%) | -24.99% (-54.67% to 16.66%) |
|  |  | Deaths | All age number | 10.89 (7.04 to 15.89) | 2.28 (1.21 to 3.84) | 8.61 (5.42 to 12.78) | 34.45 (21.38 to 50.89) | 10.31 (5.4 to 16.35) | 24.15 (15.51 to 35.8) | 216.35% (102.59% to 364.17%) | 351.39% (158.5% to 702.57%) | 180.54% (71.35% to 335.85%) |
|  |  |  | Age-standardized rate (per 100,000) | 3.76 (2.41 to 5.54) | 1.8 (0.96 to 2.97) | 5.61 (3.58 to 8.45) | 3.22 (1.99 to 4.73) | 2.15 (1.14 to 3.4) | 4.3 (2.74 to 6.39) | -14.29% (-43.58% to 24.37%) | 19.22% (-30.88% to 110.78%) | -23.38% (-52.34% to 19.82%) |
|  |  | YLDs | All age number | 2.68 (1.59 to 4.23) | 0.54 (0.25 to 0.96) | 2.14 (1.27 to 3.47) | 8.58 (4.76 to 12.73) | 2.3 (1.19 to 3.7) | 6.28 (3.56 to 9.38) | 220.3% (102.93% to 384.34%) | 328.18% (136.17% to 719.41%) | 193.27% (73.16% to 379.1%) |
|  |  |  | Age-standardized rate (per 100,000) | 0.84 (0.51 to 1.32) | 0.38 (0.17 to 0.66) | 1.28 (0.75 to 2.04) | 0.74 (0.41 to 1.12) | 0.43 (0.22 to 0.71) | 1.05 (0.59 to 1.57) | -11.76% (-44.07% to 32.24%) | 14.77% (-36.98% to 120.97%) | -17.88% (-52.62% to 33.9%) |
|  |  | YLLs | All age number | 314.54 (202.39 to 455.96) | 65.68 (35.22 to 109.84) | 248.86 (155.89 to 369.03) | 913.25 (571.72 to 1341.81) | 256.7 (137.82 to 406.63) | 656.55 (423.76 to 980.09) | 190.34% (87.69% to 322.89%) | 290.82% (121.59% to 606.56%) | 163.82% (58.44% to 315.61%) |
|  |  |  | Age-standardized rate (per 100,000) | 92.23 (59.67 to 135.39) | 41.42 (22.23 to 69.12) | 139.1 (87.59 to 207.61) | 75.04 (46.69 to 110.35) | 45.12 (23.9 to 71.44) | 104.24 (67.02 to 155.25) | -18.64% (-47.58% to 18.94%) | 8.93% (-37.07% to 93.37%) | -25.06% (-54.74% to 16.63%) |
|  | Metabolic risks | DALYs | All age number | 298.7 (120.26 to 495.99) | 159.36 (65.8 to 270.75) | 139.34 (50.76 to 244.16) | 1855.9 (564.48 to 3158.16) | 999.72 (270.43 to 1751.25) | 856.18 (296.06 to 1453.99) | 521.32% (338.11% to 702.98%) | 527.32% (280.71% to 788.71%) | 514.46% (322.19% to 792.77%) |
|  |  |  | Age-standardized rate (per 100,000) | 89.19 (32.41 to 151.02) | 100.58 (38.81 to 170.19) | 79.05 (26.83 to 141.05) | 157.89 (45.66 to 270.09) | 179.92 (46.99 to 316.48) | 139.09 (45.19 to 240.35) | 77.04% (28.56% to 123.97%) | 78.88% (13.5% to 144.42%) | 75.97% (24.24% to 156.39%) |
|  |  | Deaths | All age number | 10.29 (3.59 to 17.53) | 5.5 (2.12 to 9.44) | 4.79 (1.59 to 8.66) | 70.7 (20.05 to 120.22) | 38.69 (10.04 to 68.06) | 32.01 (10.07 to 56.04) | 587.21% (391.97% to 768.9%) | 603.89% (342.82% to 868.51%) | 568.08% (370.78% to 856.88%) |
|  |  |  | Age-standardized rate (per 100,000) | 3.69 (1.22 to 6.42) | 4.19 (1.54 to 7.27) | 3.26 (0.99 to 5.85) | 6.78 (1.88 to 11.64) | 8.01 (2.06 to 14.22) | 5.87 (1.77 to 10.31) | 83.75% (36.75% to 130.57%) | 91.34% (26.8% to 155.94%) | 79.93% (31.08% to 154.57%) |
|  |  | YLDs | All age number | 6.55 (2.08 to 11.65) | 4.31 (1.3 to 7.77) | 2.25 (0.79 to 4.19) | 76.17 (21.35 to 139.28) | 53.81 (10.27 to 101.67) | 22.35 (8.85 to 39.58) | 1062.19% (696.39% to 1383.46%) | 1149.67% (717.65% to 1697.87%) | 894.58% (610.86% to 1435.2%) |
|  |  |  | Age-standardized rate (per 100,000) | 2.05 (0.6 to 3.65) | 2.81 (0.79 to 5.18) | 1.37 (0.45 to 2.56) | 6.52 (1.73 to 12.02) | 9.52 (1.84 to 17.99) | 3.7 (1.38 to 6.62) | 218.02% (125.21% to 304.38%) | 238.92% (130.88% to 374.48%) | 170.52% (95.1% to 309.61%) |
|  |  | YLLs | All age number | 292.15 (118.31 to 485.1) | 155.06 (64.36 to 263.31) | 137.09 (49.85 to 240.44) | 1779.73 (544.02 to 3028.58) | 945.91 (256.71 to 1647.04) | 833.82 (286.23 to 1414.94) | 509.19% (327.87% to 686.03%) | 510.04% (270.71% to 762.89%) | 508.23% (317.07% to 785.01%) |
|  |  |  | Age-standardized rate (per 100,000) | 87.13 (31.79 to 147.49) | 97.77 (37.98 to 165.72) | 77.68 (26.3 to 138.6) | 151.37 (44.01 to 257.81) | 170.4 (44.76 to 297.57) | 135.4 (43.77 to 234.03) | 73.72% (25.85% to 119.93%) | 74.29% (10.31% to 138.97%) | 74.3% (22.71% to 153.09%) |
| Semnan | All risk factors | DALYs | All age number | 1809.79 (1339.32 to 2428.3) | 579.5 (375.9 to 830.7) | 1230.3 (895.11 to 1732.56) | 3826.74 (2759.42 to 4926.74) | 1394.17 (854.12 to 2011.17) | 2432.57 (1813.56 to 3180.57) | 111.45% (60.77% to 171.75%) | 140.58% (65.44% to 243.06%) | 97.72% (41.15% to 178.67%) |
|  |  |  | Age-standardized rate (per 100,000) | 621.99 (460.78 to 831.99) | 397.86 (257.52 to 569.0) | 850.77 (621.08 to 1198.28) | 511.12 (370.4 to 659.01) | 359.93 (219.01 to 519.6) | 669.06 (503.8 to 871.62) | -17.82% (-36.99% to 5.58%) | -9.53% (-37.08% to 28.73%) | -21.36% (-43.44% to 10.16%) |
|  |  | Deaths | All age number | 64.67 (48.12 to 87.07) | 19.32 (12.58 to 27.46) | 45.35 (32.92 to 63.0) | 147.31 (107.32 to 189.54) | 50.82 (31.08 to 72.95) | 96.49 (73.2 to 125.43) | 127.79% (74.93% to 190.52%) | 163.04% (86.89% to 272.98%) | 112.78% (53.9% to 194.59%) |
|  |  |  | Age-standardized rate (per 100,000) | 24.85 (18.72 to 33.35) | 14.89 (9.68 to 21.11) | 35.73 (26.29 to 49.38) | 21.49 (15.67 to 27.58) | 14.19 (8.68 to 20.35) | 29.35 (22.13 to 38.15) | -13.51% (-32.99% to 9.84%) | -4.69% (-31.87% to 33.96%) | -17.85% (-40.5% to 11.84%) |
|  |  | YLDs | All age number | 29.76 (18.63 to 42.91) | 13.03 (6.52 to 21.06) | 16.73 (10.87 to 24.01) | 114.44 (64.24 to 173.6) | 64.39 (29.21 to 108.24) | 50.04 (32.27 to 73.43) | 284.55% (183.55% to 393.06%) | 394.17% (239.34% to 599.47%) | 199.17% (114.26% to 331.66%) |
|  |  |  | Age-standardized rate (per 100,000) | 10.41 (6.57 to 14.92) | 8.97 (4.5 to 14.4) | 11.96 (7.84 to 17.07) | 15.05 (8.55 to 22.66) | 16.27 (7.27 to 26.75) | 14.04 (9.1 to 20.37) | 44.65% (8.62% to 83.77%) | 81.27% (24.57% to 153.7%) | 17.41% (-14.33% to 67.15%) |
|  |  | YLLs | All age number | 1780.04 (1318.28 to 2388.87) | 566.47 (368.29 to 811.91) | 1213.57 (882.02 to 1708.89) | 3712.31 (2698.88 to 4792.66) | 1329.78 (821.08 to 1901.94) | 2382.53 (1779.55 to 3117.72) | 108.55% (58.43% to 168.35%) | 134.75% (60.26% to 235.13%) | 96.32% (40.08% to 176.56%) |
|  |  |  | Age-standardized rate (per 100,000) | 611.58 (453.87 to 819.77) | 388.89 (252.26 to 555.55) | 838.81 (611.12 to 1182.52) | 496.07 (362.39 to 639.71) | 343.66 (211.17 to 492.09) | 655.02 (494.49 to 853.64) | -18.89% (-37.7% to 4.48%) | -11.63% (-38.6% to 25.7%) | -21.91% (-43.79% to 9.21%) |
|  | Behavioral risks | DALYs | All age number | 1543.68 (1115.55 to 2130.24) | 446.19 (268.79 to 674.15) | 1097.49 (773.18 to 1558.75) | 2778.05 (2017.84 to 3605.3) | 836.31 (468.17 to 1237.82) | 1941.74 (1440.29 to 2597.41) | 79.96% (38.6% to 135.84%) | 87.44% (29.7% to 169.58%) | 76.92% (26.62% to 151.22%) |
|  |  |  | Age-standardized rate (per 100,000) | 530.33 (383.32 to 733.78) | 305.44 (182.65 to 456.74) | 759.27 (535.87 to 1067.87) | 369.97 (269.3 to 481.36) | 211.27 (120.82 to 311.27) | 535.27 (398.51 to 712.53) | -30.24% (-45.71% to -8.96%) | -30.83% (-51.49% to -2.49%) | -29.5% (-49.13% to -1.28%) |
|  |  | Deaths | All age number | 55.23 (40.18 to 77.16) | 14.61 (8.76 to 21.6) | 40.61 (28.77 to 56.91) | 106.39 (77.53 to 136.42) | 29.06 (17.41 to 42.24) | 77.32 (57.81 to 101.41) | 92.64% (49.25% to 150.47%) | 98.9% (41.93% to 176.66%) | 90.39% (37.79% to 165.11%) |
|  |  |  | Age-standardized rate (per 100,000) | 21.1 (15.28 to 29.43) | 11.21 (6.67 to 16.47) | 31.86 (22.69 to 44.72) | 15.47 (11.28 to 19.91) | 7.99 (4.79 to 11.66) | 23.5 (17.64 to 30.68) | -26.71% (-42.88% to -6.36%) | -28.69% (-48.21% to -1.51%) | -26.25% (-46.09% to 0.9%) |
|  |  | YLDs | All age number | 25.27 (15.72 to 37.4) | 10.2 (4.68 to 17.41) | 15.07 (9.64 to 22.02) | 81.49 (42.02 to 126.71) | 41.56 (13.83 to 73.06) | 39.93 (25.45 to 58.63) | 222.48% (134.11% to 314.04%) | 307.61% (155.16% to 483.62%) | 164.89% (90.68% to 274.49%) |
|  |  |  | Age-standardized rate (per 100,000) | 8.8 (5.52 to 13.12) | 6.96 (3.21 to 11.82) | 10.75 (6.96 to 15.62) | 10.57 (5.66 to 16.21) | 10.12 (3.44 to 17.58) | 11.23 (7.2 to 16.35) | 20.11% (-10.72% to 52.15%) | 45.47% (-7.84% to 104.49%) | 4.45% (-23.71% to 45.01%) |
|  |  | YLLs | All age number | 1518.41 (1098.67 to 2100.17) | 435.99 (263.38 to 655.43) | 1082.42 (762.19 to 1540.06) | 2696.57 (1970.11 to 3506.82) | 794.75 (451.84 to 1178.99) | 1901.81 (1408.83 to 2544.83) | 77.59% (36.47% to 133.32%) | 82.29% (27.59% to 162.01%) | 75.7% (25.66% to 149.77%) |
|  |  |  | Age-standardized rate (per 100,000) | 521.53 (377.7 to 723.49) | 298.48 (178.97 to 446.58) | 748.52 (527.88 to 1054.08) | 359.4 (263.16 to 467.21) | 201.15 (117.0 to 296.81) | 524.04 (389.38 to 694.45) | -31.09% (-46.48% to -9.71%) | -32.61% (-52.32% to -4.86%) | -29.99% (-49.46% to -1.77%) |
|  | Environmental/occupational risks | DALYs | All age number | 236.43 (151.41 to 355.44) | 46.26 (24.33 to 78.93) | 190.17 (118.84 to 287.13) | 516.19 (332.07 to 719.2) | 134.92 (77.41 to 205.88) | 381.27 (243.91 to 534.99) | 118.32% (47.66% to 224.09%) | 191.66% (63.89% to 396.76%) | 100.48% (28.01% to 214.95%) |
|  |  |  | Age-standardized rate (per 100,000) | 81.0 (51.5 to 122.21) | 32.47 (17.57 to 54.94) | 130.34 (81.72 to 198.64) | 69.0 (44.38 to 96.23) | 35.41 (20.22 to 54.46) | 103.44 (66.03 to 146.04) | -14.82% (-41.83% to 25.92%) | 9.06% (-38.03% to 83.87%) | -20.63% (-49.63% to 22.98%) |
|  |  | Deaths | All age number | 8.58 (5.46 to 12.89) | 1.66 (0.91 to 2.77) | 6.92 (4.29 to 10.43) | 20.22 (12.94 to 28.31) | 5.37 (3.04 to 8.44) | 14.85 (9.48 to 20.84) | 135.59% (61.34% to 249.99%) | 224.1% (88.1% to 445.93%) | 114.41% (35.59% to 232.35%) |
|  |  |  | Age-standardized rate (per 100,000) | 3.29 (2.09 to 4.98) | 1.32 (0.73 to 2.19) | 5.44 (3.41 to 8.39) | 2.94 (1.87 to 4.14) | 1.53 (0.87 to 2.43) | 4.45 (2.81 to 6.2) | -10.59% (-37.95% to 31.32%) | 15.39% (-32.54% to 94.35%) | -18.17% (-47.61% to 24.12%) |
|  |  | YLDs | All age number | 2.04 (1.12 to 3.35) | 0.39 (0.19 to 0.73) | 1.65 (0.88 to 2.73) | 4.77 (2.75 to 7.38) | 1.22 (0.64 to 2.03) | 3.56 (2.09 to 5.55) | 133.74% (54.78% to 253.94%) | 210.57% (72.85% to 447.45%) | 115.5% (29.97% to 254.72%) |
|  |  |  | Age-standardized rate (per 100,000) | 0.73 (0.41 to 1.19) | 0.29 (0.14 to 0.54) | 1.19 (0.64 to 1.92) | 0.66 (0.38 to 1.02) | 0.33 (0.17 to 0.56) | 1.0 (0.58 to 1.55) | -9.44% (-39.55% to 37.27%) | 14.4% (-36.91% to 101.82%) | -15.63% (-48.81% to 38.06%) |
|  |  | YLLs | All age number | 234.39 (150.11 to 351.9) | 45.87 (24.14 to 78.23) | 188.52 (117.8 to 284.45) | 511.41 (328.55 to 712.45) | 133.7 (76.54 to 204.2) | 377.71 (241.23 to 529.85) | 118.19% (47.51% to 224.12%) | 191.5% (63.9% to 396.78%) | 100.35% (27.93% to 215.36%) |
|  |  |  | Age-standardized rate (per 100,000) | 80.27 (51.1 to 120.83) | 32.18 (17.4 to 54.35) | 129.16 (80.98 to 196.65) | 68.34 (43.89 to 95.29) | 35.08 (20.02 to 54.0) | 102.44 (65.44 to 144.61) | -14.87% (-41.85% to 25.95%) | 9.01% (-38.04% to 84.07%) | -20.68% (-49.7% to 23.14%) |
|  | Metabolic risks | DALYs | All age number | 226.85 (76.93 to 374.7) | 120.67 (41.15 to 208.79) | 106.17 (37.42 to 182.34) | 1053.66 (338.1 to 1800.05) | 547.06 (161.75 to 942.62) | 506.59 (164.78 to 856.94) | 364.48% (237.09% to 488.32%) | 353.35% (196.18% to 541.03%) | 377.13% (215.64% to 577.32%) |
|  |  |  | Age-standardized rate (per 100,000) | 78.18 (25.33 to 130.71) | 83.8 (27.75 to 146.16) | 72.67 (24.85 to 124.2) | 142.67 (44.65 to 245.26) | 146.53 (41.81 to 253.43) | 139.07 (43.85 to 235.78) | 82.48% (34.26% to 129.19%) | 74.85% (17.36% to 144.65%) | 91.38% (31.52% to 167.9%) |
|  |  | Deaths | All age number | 8.1 (2.55 to 13.62) | 4.28 (1.36 to 7.52) | 3.82 (1.24 to 6.55) | 41.24 (12.58 to 71.75) | 21.31 (5.97 to 36.79) | 19.93 (6.05 to 33.96) | 408.87% (285.21% to 538.36%) | 397.32% (246.04% to 589.08%) | 421.84% (260.72% to 634.48%) |
|  |  |  | Age-standardized rate (per 100,000) | 3.18 (0.96 to 5.39) | 3.33 (1.02 to 5.89) | 3.03 (0.95 to 5.2) | 6.08 (1.82 to 10.65) | 6.07 (1.69 to 10.56) | 6.09 (1.8 to 10.5) | 91.33% (45.54% to 139.71%) | 82.48% (27.39% to 149.79%) | 101.32% (45.44% to 174.4%) |
|  |  | YLDs | All age number | 4.92 (1.31 to 8.88) | 3.15 (0.66 to 6.08) | 1.76 (0.61 to 3.24) | 39.31 (10.33 to 71.43) | 26.61 (5.74 to 49.9) | 12.7 (4.48 to 22.36) | 699.71% (485.13% to 949.86%) | 744.45% (454.7% to 1151.96%) | 619.83% (392.05% to 957.65%) |
|  |  |  | Age-standardized rate (per 100,000) | 1.75 (0.44 to 3.2) | 2.24 (0.47 to 4.35) | 1.27 (0.43 to 2.29) | 5.36 (1.37 to 9.8) | 7.18 (1.51 to 13.47) | 3.55 (1.21 to 6.26) | 206.35% (129.95% to 302.15%) | 220.24% (116.14% to 366.73%) | 180.13% (97.03% to 301.61%) |
|  |  | YLLs | All age number | 221.93 (75.62 to 366.53) | 117.52 (40.54 to 202.81) | 104.41 (36.75 to 179.14) | 1014.35 (327.2 to 1730.15) | 520.46 (154.72 to 894.26) | 493.89 (160.27 to 835.13) | 357.06% (234.91% to 480.03%) | 342.87% (190.7% to 526.83%) | 373.03% (212.94% to 569.6%) |
|  |  |  | Age-standardized rate (per 100,000) | 76.43 (24.89 to 127.74) | 81.56 (27.12 to 142.51) | 71.4 (24.38 to 121.75) | 137.31 (43.07 to 235.64) | 139.35 (40.04 to 239.51) | 135.52 (42.55 to 229.64) | 79.64% (32.44% to 125.58%) | 70.86% (15.15% to 138.71%) | 89.8% (30.68% to 165.88%) |
| Sistan and Baluchistan | All risk factors | DALYs | All age number | 2757.54 (1999.72 to 3905.13) | 743.83 (479.45 to 1161.72) | 2013.72 (1420.32 to 2933.28) | 7421.51 (5661.43 to 9790.39) | 2802.0 (1854.88 to 3933.71) | 4619.51 (3512.36 to 6144.6) | 169.13% (101.66% to 263.86%) | 276.7% (148.71% to 473.47%) | 129.4% (56.4% to 239.97%) |
|  |  |  | Age-standardized rate (per 100,000) | 432.4 (312.42 to 609.27) | 247.53 (160.77 to 384.1) | 575.87 (406.45 to 832.16) | 427.85 (327.3 to 557.4) | 304.25 (201.37 to 429.39) | 553.09 (421.03 to 733.55) | -1.05% (-25.87% to 35.41%) | 22.91% (-18.49% to 84.13%) | -3.96% (-33.86% to 41.37%) |
|  |  | Deaths | All age number | 91.68 (66.07 to 129.32) | 22.35 (14.48 to 34.8) | 69.33 (49.06 to 100.1) | 247.25 (189.28 to 321.96) | 86.58 (56.9 to 121.82) | 160.67 (121.82 to 212.62) | 169.7% (101.62% to 271.13%) | 287.42% (156.74% to 476.04%) | 131.76% (59.06% to 246.25%) |
|  |  |  | Age-standardized rate (per 100,000) | 16.35 (11.72 to 22.71) | 8.84 (5.77 to 13.68) | 22.48 (16.07 to 32.16) | 16.23 (12.39 to 21.0) | 10.92 (7.18 to 15.38) | 21.52 (16.31 to 28.65) | -0.73% (-24.61% to 34.96%) | 23.57% (-17.48% to 80.99%) | -4.25% (-33.71% to 40.25%) |
|  |  | YLDs | All age number | 35.44 (22.84 to 50.77) | 12.4 (6.85 to 19.83) | 23.04 (14.17 to 34.03) | 139.48 (82.58 to 214.51) | 75.99 (37.03 to 126.23) | 63.49 (41.02 to 91.31) | 293.57% (188.39% to 434.45%) | 512.84% (303.42% to 827.87%) | 175.56% (87.19% to 307.69%) |
|  |  |  | Age-standardized rate (per 100,000) | 5.78 (3.73 to 8.3) | 4.29 (2.35 to 6.85) | 6.92 (4.3 to 10.13) | 7.98 (4.87 to 11.86) | 8.04 (4.05 to 13.17) | 7.92 (5.15 to 11.44) | 37.98% (3.84% to 85.71%) | 87.43% (25.19% to 175.88%) | 14.43% (-21.07% to 66.56%) |
|  |  | YLLs | All age number | 2722.1 (1974.44 to 3861.13) | 731.43 (472.02 to 1145.05) | 1990.68 (1403.85 to 2904.51) | 7282.03 (5561.49 to 9587.7) | 2726.01 (1802.22 to 3832.23) | 4556.02 (3467.77 to 6071.82) | 167.51% (100.78% to 262.37%) | 272.7% (146.15% to 467.27%) | 128.87% (55.96% to 239.35%) |
|  |  |  | Age-standardized rate (per 100,000) | 426.61 (308.32 to 600.47) | 243.24 (158.32 to 378.03) | 568.94 (401.45 to 822.86) | 419.87 (321.2 to 547.84) | 296.2 (196.32 to 418.27) | 545.16 (415.08 to 723.82) | -1.58% (-26.3% to 34.9%) | 21.78% (-19.27% to 82.45%) | -4.18% (-34.03% to 41.07%) |
|  | Behavioral risks | DALYs | All age number | 2361.07 (1668.46 to 3439.7) | 576.03 (340.18 to 958.65) | 1785.04 (1232.5 to 2631.46) | 5362.04 (3864.39 to 7290.47) | 1739.43 (1041.38 to 2654.21) | 3622.62 (2625.14 to 5017.23) | 127.1% (70.54% to 206.22%) | 201.97% (97.34% to 356.1%) | 102.94% (41.3% to 202.04%) |
|  |  |  | Age-standardized rate (per 100,000) | 371.39 (258.07 to 537.72) | 190.73 (112.66 to 315.07) | 511.31 (354.9 to 745.05) | 311.09 (225.27 to 419.26) | 182.63 (111.67 to 269.49) | 441.23 (321.71 to 603.59) | -16.24% (-36.97% to 13.97%) | -4.25% (-34.96% to 40.87%) | -13.71% (-40.02% to 27.41%) |
|  |  | Deaths | All age number | 78.93 (54.85 to 114.02) | 17.13 (10.05 to 28.42) | 61.8 (42.82 to 90.62) | 179.15 (130.46 to 243.1) | 51.66 (31.34 to 76.87) | 127.49 (93.08 to 173.78) | 126.98% (70.82% to 209.71%) | 201.58% (105.98% to 341.52%) | 106.3% (44.01% to 202.95%) |
|  |  |  | Age-standardized rate (per 100,000) | 14.01 (9.68 to 20.2) | 6.72 (3.99 to 10.98) | 19.95 (13.86 to 28.73) | 11.78 (8.59 to 16.02) | 6.32 (3.81 to 9.59) | 17.22 (12.63 to 23.6) | -15.96% (-36.45% to 14.3%) | -5.9% (-34.31% to 35.04%) | -13.68% (-39.32% to 25.24%) |
|  |  | YLDs | All age number | 30.81 (19.65 to 44.78) | 10.02 (5.27 to 16.64) | 20.79 (12.75 to 31.07) | 101.68 (55.32 to 162.81) | 51.34 (20.29 to 94.96) | 50.34 (31.68 to 75.42) | 230.02% (130.15% to 349.48%) | 412.56% (195.52% to 692.5%) | 142.09% (64.88% to 251.34%) |
|  |  |  | Age-standardized rate (per 100,000) | 5.01 (3.2 to 7.33) | 3.39 (1.72 to 5.64) | 6.24 (3.86 to 9.25) | 5.71 (3.26 to 8.81) | 5.07 (2.09 to 8.98) | 6.38 (4.03 to 9.47) | 14.16% (-17.11% to 52.14%) | 49.52% (-7.22% to 123.34%) | 2.31% (-29.16% to 47.35%) |
|  |  | YLLs | All age number | 2330.26 (1643.99 to 3396.54) | 566.02 (334.87 to 941.76) | 1764.24 (1217.5 to 2602.41) | 5260.36 (3792.42 to 7148.25) | 1688.08 (1018.14 to 2566.87) | 3572.28 (2589.6 to 4954.15) | 125.74% (69.63% to 204.88%) | 198.24% (97.28% to 348.54%) | 102.48% (40.97% to 201.34%) |
|  |  |  | Age-standardized rate (per 100,000) | 366.39 (254.54 to 530.84) | 187.34 (110.16 to 309.81) | 505.07 (350.49 to 736.65) | 305.37 (221.32 to 412.19) | 177.56 (109.35 to 261.36) | 434.85 (317.31 to 594.57) | -16.65% (-37.25% to 13.54%) | -5.22% (-35.19% to 39.31%) | -13.9% (-40.12% to 27.12%) |
|  | Environmental/occupational risks | DALYs | All age number | 522.23 (331.09 to 760.55) | 103.67 (58.21 to 169.09) | 418.57 (261.65 to 627.35) | 1440.46 (944.61 to 2028.05) | 455.52 (271.74 to 729.56) | 984.94 (655.42 to 1412.61) | 175.83% (83.89% to 339.54%) | 339.41% (140.55% to 720.47%) | 135.31% (49.74% to 299.56%) |
|  |  |  | Age-standardized rate (per 100,000) | 82.01 (52.31 to 119.67) | 35.96 (20.17 to 57.94) | 118.47 (75.04 to 175.5) | 83.17 (53.95 to 117.01) | 50.78 (30.09 to 81.58) | 115.98 (77.06 to 166.37) | 1.41% (-32.51% to 62.72%) | 41.19% (-20.48% to 162.37%) | -2.1% (-37.06% to 65.77%) |
|  |  | Deaths | All age number | 17.5 (11.1 to 25.89) | 3.32 (1.85 to 5.31) | 14.18 (8.7 to 21.26) | 48.72 (31.55 to 68.69) | 15.02 (8.89 to 24.1) | 33.7 (22.41 to 48.05) | 178.37% (87.67% to 342.4%) | 352.52% (153.1% to 743.78%) | 137.62% (53.54% to 302.94%) |
|  |  |  | Age-standardized rate (per 100,000) | 3.13 (2.0 to 4.55) | 1.38 (0.77 to 2.19) | 4.57 (2.88 to 6.8) | 3.21 (2.06 to 4.55) | 1.96 (1.16 to 3.11) | 4.46 (2.94 to 6.37) | 2.36% (-31.92% to 62.31%) | 41.45% (-18.43% to 160.82%) | -2.41% (-36.48% to 65.01%) |
|  |  | YLDs | All age number | 4.31 (2.41 to 7.05) | 0.84 (0.44 to 1.36) | 3.47 (1.88 to 5.92) | 11.8 (6.76 to 17.98) | 3.58 (1.83 to 5.95) | 8.22 (4.65 to 12.9) | 173.91% (78.86% to 351.46%) | 327.98% (139.18% to 707.6%) | 136.78% (45.33% to 334.96%) |
|  |  |  | Age-standardized rate (per 100,000) | 0.72 (0.4 to 1.18) | 0.32 (0.17 to 0.53) | 1.04 (0.57 to 1.76) | 0.73 (0.41 to 1.11) | 0.43 (0.22 to 0.73) | 1.03 (0.57 to 1.65) | 1.48% (-33.64% to 67.29%) | 35.06% (-26.3% to 151.76%) | -0.92% (-39.19% to 77.2%) |
|  |  | YLLs | All age number | 517.93 (328.05 to 754.85) | 102.83 (57.74 to 167.86) | 415.1 (259.23 to 620.56) | 1428.66 (936.65 to 2012.38) | 451.94 (269.77 to 723.71) | 976.72 (649.61 to 1400.19) | 175.84% (83.83% to 339.92%) | 339.5% (140.63% to 720.59%) | 135.3% (50.03% to 299.01%) |
|  |  |  | Age-standardized rate (per 100,000) | 81.3 (51.79 to 118.64) | 35.64 (20.03 to 57.48) | 117.43 (74.28 to 173.74) | 82.44 (53.48 to 116.15) | 50.35 (29.86 to 81.01) | 114.96 (76.31 to 164.75) | 1.41% (-32.53% to 62.72%) | 41.25% (-20.43% to 162.36%) | -2.11% (-37.07% to 65.71%) |
|  | Metabolic risks | DALYs | All age number | 210.54 (101.3 to 337.27) | 99.52 (47.15 to 161.9) | 111.02 (44.05 to 190.83) | 1538.95 (585.43 to 2551.07) | 794.32 (308.54 to 1358.91) | 744.63 (279.59 to 1256.73) | 630.96% (424.95% to 889.39%) | 698.16% (400.99% to 1085.13%) | 570.73% (336.46% to 891.81%) |
|  |  |  | Age-standardized rate (per 100,000) | 32.17 (14.37 to 52.11) | 33.57 (14.3 to 56.11) | 31.03 (11.65 to 54.36) | 89.94 (30.74 to 153.49) | 93.69 (33.41 to 161.96) | 86.08 (28.58 to 149.13) | 179.58% (99.58% to 274.78%) | 179.06% (75.7% to 310.16%) | 177.36% (83.15% to 309.95%) |
|  |  | Deaths | All age number | 6.74 (2.94 to 11.01) | 3.07 (1.32 to 5.15) | 3.67 (1.35 to 6.48) | 51.64 (17.36 to 87.85) | 26.4 (9.42 to 45.83) | 25.24 (8.1 to 43.68) | 666.35% (452.62% to 916.76%) | 760.78% (437.45% to 1161.65%) | 587.47% (362.21% to 916.49%) |
|  |  |  | Age-standardized rate (per 100,000) | 1.2 (0.48 to 1.99) | 1.22 (0.5 to 2.07) | 1.19 (0.42 to 2.09) | 3.43 (1.07 to 5.94) | 3.52 (1.19 to 6.14) | 3.33 (0.98 to 5.87) | 185.29% (106.07% to 278.54%) | 189.66% (83.81% to 318.45%) | 178.97% (88.21% to 312.29%) |
|  |  | YLDs | All age number | 3.51 (1.25 to 6.35) | 2.05 (0.69 to 3.81) | 1.46 (0.54 to 2.77) | 37.16 (14.5 to 63.68) | 24.65 (8.87 to 42.78) | 12.51 (5.18 to 21.78) | 958.9% (632.02% to 1417.05%) | 1100.01% (625.56% to 1835.34%) | 759.77% (480.35% to 1233.48%) |
|  |  |  | Age-standardized rate (per 100,000) | 0.59 (0.2 to 1.08) | 0.78 (0.25 to 1.46) | 0.44 (0.16 to 0.84) | 2.26 (0.77 to 3.99) | 3.02 (0.94 to 5.46) | 1.49 (0.57 to 2.68) | 284.33% (173.85% to 440.28%) | 287.22% (146.63% to 500.33%) | 237.23% (135.64% to 410.16%) |
|  |  | YLLs | All age number | 207.03 (99.84 to 331.53) | 97.46 (46.4 to 158.1) | 109.56 (43.47 to 188.55) | 1501.79 (570.83 to 2493.31) | 769.67 (299.89 to 1312.6) | 732.11 (274.22 to 1236.62) | 625.41% (418.28% to 882.31%) | 689.7% (392.66% to 1073.52%) | 568.21% (334.65% to 888.05%) |
|  |  |  | Age-standardized rate (per 100,000) | 31.58 (14.15 to 51.3) | 32.79 (14.08 to 54.66) | 30.59 (11.47 to 53.58) | 87.68 (29.82 to 149.73) | 90.67 (32.41 to 156.59) | 84.59 (27.95 to 146.7) | 177.62% (97.48% to 272.99%) | 176.49% (71.84% to 307.29%) | 176.49% (82.21% to 309.11%) |
| South Khorasan | All risk factors | DALYs | All age number | 2037.09 (1552.38 to 2794.72) | 610.73 (398.05 to 882.9) | 1426.37 (1036.12 to 2098.28) | 3668.32 (2794.42 to 4653.82) | 1399.22 (898.03 to 1927.18) | 2269.09 (1748.28 to 2915.33) | 80.08% (34.85% to 142.49%) | 129.11% (59.89% to 214.02%) | 59.08% (9.05% to 128.92%) |
|  |  |  | Age-standardized rate (per 100,000) | 528.34 (406.12 to 726.15) | 332.64 (216.63 to 476.86) | 700.62 (510.77 to 1021.91) | 468.97 (358.8 to 595.25) | 343.45 (221.18 to 473.08) | 603.49 (463.01 to 774.46) | -11.24% (-33.38% to 17.91%) | 3.25% (-26.88% to 41.53%) | -13.86% (-40.51% to 23.19%) |
|  |  | Deaths | All age number | 71.88 (54.99 to 98.97) | 20.12 (13.05 to 28.71) | 51.75 (37.83 to 75.24) | 144.87 (110.71 to 184.53) | 52.57 (34.06 to 72.84) | 92.3 (70.53 to 119.14) | 101.55% (50.92% to 170.79%) | 161.2% (87.13% to 256.99%) | 78.35% (22.93% to 155.69%) |
|  |  |  | Age-standardized rate (per 100,000) | 21.19 (16.08 to 29.32) | 12.63 (8.25 to 18.07) | 28.96 (21.19 to 41.67) | 19.26 (14.78 to 24.6) | 13.44 (8.73 to 18.65) | 25.53 (19.42 to 32.99) | -9.11% (-31.1% to 20.21%) | 6.46% (-23.37% to 43.98%) | -11.84% (-38.45% to 24.24%) |
|  |  | YLDs | All age number | 30.22 (19.45 to 43.77) | 11.82 (6.52 to 18.56) | 18.4 (11.97 to 27.14) | 97.03 (58.22 to 146.06) | 53.61 (25.41 to 89.07) | 43.42 (29.35 to 60.78) | 221.06% (137.7% to 320.26%) | 353.57% (207.06% to 519.98%) | 135.94% (66.66% to 235.96%) |
|  |  |  | Age-standardized rate (per 100,000) | 8.06 (5.23 to 11.59) | 6.52 (3.57 to 10.12) | 9.4 (6.23 to 13.8) | 12.3 (7.43 to 18.42) | 12.94 (6.19 to 21.41) | 11.68 (7.88 to 16.31) | 52.51% (14.73% to 99.36%) | 98.46% (33.91% to 169.74%) | 24.29% (-10.62% to 74.19%) |
|  |  | YLLs | All age number | 2006.87 (1528.88 to 2759.56) | 598.91 (390.47 to 867.81) | 1407.97 (1023.86 to 2075.24) | 3571.29 (2740.0 to 4526.45) | 1345.61 (870.8 to 1846.33) | 2225.67 (1717.59 to 2857.57) | 77.95% (33.08% to 139.33%) | 124.68% (56.61% to 208.28%) | 58.08% (8.3% to 127.63%) |
|  |  |  | Age-standardized rate (per 100,000) | 520.27 (399.52 to 716.28) | 326.13 (212.56 to 467.28) | 691.22 (504.09 to 1008.83) | 456.68 (351.5 to 580.02) | 330.52 (215.05 to 453.92) | 591.8 (454.17 to 760.19) | -12.22% (-34.06% to 16.48%) | 1.35% (-28.42% to 38.76%) | -14.38% (-40.96% to 22.74%) |
|  | Behavioral risks | DALYs | All age number | 1746.2 (1302.15 to 2492.26) | 470.44 (284.37 to 722.47) | 1275.76 (914.72 to 1891.66) | 2657.81 (1987.21 to 3542.13) | 837.27 (482.79 to 1230.63) | 1820.54 (1359.44 to 2415.41) | 52.21% (14.3% to 100.49%) | 77.98% (22.23% to 143.1%) | 42.7% (-1.98% to 106.09%) |
|  |  |  | Age-standardized rate (per 100,000) | 451.77 (336.33 to 645.19) | 255.04 (151.86 to 390.67) | 624.76 (445.2 to 916.22) | 339.21 (255.25 to 451.49) | 201.89 (117.5 to 295.83) | 486.47 (363.04 to 644.74) | -24.92% (-43.17% to -1.49%) | -20.84% (-44.7% to 7.03%) | -22.14% (-45.78% to 12.62%) |
|  |  | Deaths | All age number | 61.6 (45.72 to 87.24) | 15.27 (9.11 to 23.31) | 46.32 (33.12 to 68.75) | 104.37 (78.25 to 138.93) | 30.1 (17.9 to 43.35) | 74.27 (55.16 to 99.21) | 69.44% (27.73% to 126.13%) | 97.11% (39.97% to 163.19%) | 60.32% (12.37% to 128.65%) |
|  |  |  | Age-standardized rate (per 100,000) | 18.03 (13.35 to 25.74) | 9.51 (5.7 to 14.51) | 25.74 (18.21 to 37.93) | 13.86 (10.41 to 18.54) | 7.6 (4.56 to 10.99) | 20.58 (15.25 to 27.46) | -23.12% (-41.19% to -0.34%) | -20.08% (-42.46% to 7.99%) | -20.02% (-43.67% to 13.37%) |
|  |  | YLDs | All age number | 25.74 (16.05 to 37.57) | 9.12 (4.5 to 14.79) | 16.62 (10.7 to 24.66) | 69.34 (40.11 to 105.43) | 34.36 (13.32 to 60.74) | 34.97 (23.53 to 48.78) | 169.39% (93.53% to 261.42%) | 276.98% (131.49% to 435.37%) | 110.39% (49.64% to 204.33%) |
|  |  |  | Age-standardized rate (per 100,000) | 6.85 (4.3 to 9.91) | 4.99 (2.49 to 7.99) | 8.45 (5.5 to 12.48) | 8.68 (5.11 to 13.12) | 8.03 (3.15 to 14.12) | 9.46 (6.34 to 13.18) | 26.81% (-6.95% to 67.62%) | 60.81% (0.57% to 126.84%) | 11.99% (-19.08% to 60.52%) |
|  |  | YLLs | All age number | 1720.46 (1283.98 to 2459.33) | 461.32 (279.33 to 708.93) | 1259.14 (903.68 to 1863.61) | 2588.47 (1941.11 to 3441.81) | 802.91 (469.23 to 1173.16) | 1785.57 (1332.37 to 2372.7) | 50.45% (12.69% to 98.1%) | 74.04% (20.58% to 136.99%) | 41.81% (-2.57% to 104.95%) |
|  |  |  | Age-standardized rate (per 100,000) | 444.92 (330.97 to 636.53) | 250.04 (149.15 to 383.14) | 616.31 (439.19 to 905.4) | 330.52 (249.61 to 438.98) | 193.86 (114.27 to 282.15) | 477.0 (355.04 to 633.88) | -25.71% (-43.93% to -2.47%) | -22.47% (-45.71% to 4.96%) | -22.6% (-46.1% to 12.03%) |
|  | Environmental/occupational risks | DALYs | All age number | 312.96 (194.09 to 467.31) | 59.27 (30.42 to 100.81) | 253.69 (155.54 to 391.08) | 614.27 (405.93 to 851.07) | 195.49 (114.32 to 290.06) | 418.78 (290.19 to 583.85) | 96.28% (23.09% to 202.05%) | 229.83% (86.04% to 492.17%) | 65.07% (-4.91% to 166.41%) |
|  |  |  | Age-standardized rate (per 100,000) | 80.59 (49.92 to 119.5) | 33.08 (17.06 to 55.08) | 122.91 (75.98 to 187.34) | 78.57 (51.73 to 108.87) | 48.53 (28.4 to 72.8) | 110.69 (76.0 to 155.19) | -2.5% (-38.83% to 49.15%) | 46.71% (-17.02% to 163.58%) | -9.94% (-47.61% to 44.73%) |
|  |  | Deaths | All age number | 11.12 (6.85 to 16.55) | 2.06 (1.06 to 3.47) | 9.06 (5.51 to 13.94) | 24.55 (15.96 to 33.98) | 7.91 (4.63 to 11.86) | 16.65 (11.29 to 23.18) | 120.81% (38.12% to 240.3%) | 283.49% (116.22% to 595.29%) | 83.78% (7.35% to 198.9%) |
|  |  |  | Age-standardized rate (per 100,000) | 3.25 (2.01 to 4.84) | 1.34 (0.69 to 2.26) | 4.99 (3.07 to 7.46) | 3.27 (2.11 to 4.53) | 2.04 (1.19 to 3.08) | 4.59 (3.11 to 6.43) | 0.36% (-37.88% to 54.06%) | 52.6% (-13.86% to 175.29%) | -8.06% (-45.94% to 47.3%) |
|  |  | YLDs | All age number | 2.72 (1.54 to 4.37) | 0.5 (0.24 to 0.88) | 2.21 (1.22 to 3.64) | 5.8 (3.33 to 8.6) | 1.78 (0.94 to 2.88) | 4.03 (2.4 to 5.96) | 113.71% (33.14% to 240.98%) | 253.23% (94.78% to 527.5%) | 82.01% (4.36% to 205.04%) |
|  |  |  | Age-standardized rate (per 100,000) | 0.74 (0.42 to 1.17) | 0.3 (0.14 to 0.52) | 1.13 (0.65 to 1.83) | 0.76 (0.43 to 1.13) | 0.45 (0.24 to 0.73) | 1.09 (0.65 to 1.62) | 2.94% (-34.24% to 64.03%) | 51.07% (-18.57% to 178.7%) | -3.74% (-45.67% to 59.89%) |
|  |  | YLLs | All age number | 310.24 (192.58 to 463.53) | 58.77 (30.18 to 100.05) | 251.48 (154.01 to 386.99) | 608.46 (402.51 to 843.8) | 193.71 (113.49 to 287.62) | 414.75 (287.05 to 577.9) | 96.12% (22.97% to 201.95%) | 229.63% (86.02% to 491.84%) | 64.93% (-4.97% to 166.48%) |
|  |  |  | Age-standardized rate (per 100,000) | 79.85 (49.52 to 118.44) | 32.78 (16.9 to 54.63) | 121.78 (75.18 to 185.8) | 77.82 (51.26 to 107.93) | 48.08 (28.18 to 72.18) | 109.61 (75.16 to 153.51) | -2.55% (-38.87% to 49.15%) | 46.67% (-17.0% to 163.44%) | -10.0% (-47.62% to 44.69%) |
|  | Metabolic risks | DALYs | All age number | 215.8 (79.06 to 363.91) | 117.18 (47.31 to 204.3) | 98.63 (32.55 to 170.61) | 887.64 (294.26 to 1519.82) | 488.73 (145.42 to 837.11) | 398.9 (129.96 to 672.74) | 311.32% (199.92% to 430.54%) | 317.09% (168.21% to 486.33%) | 304.45% (180.91% to 481.48%) |
|  |  |  | Age-standardized rate (per 100,000) | 56.69 (19.83 to 96.06) | 64.77 (25.36 to 113.26) | 49.53 (15.88 to 87.17) | 115.04 (37.16 to 199.03) | 124.02 (36.16 to 215.71) | 105.21 (32.83 to 178.46) | 102.92% (49.52% to 161.49%) | 91.47% (24.96% to 166.14%) | 112.42% (48.6% to 202.48%) |
|  |  | Deaths | All age number | 7.7 (2.58 to 13.19) | 4.08 (1.55 to 7.17) | 3.62 (1.1 to 6.38) | 35.66 (11.1 to 61.97) | 19.51 (5.56 to 33.86) | 16.15 (4.7 to 27.82) | 362.86% (242.45% to 494.6%) | 377.56% (213.71% to 546.28%) | 346.27% (217.87% to 531.49%) |
|  |  |  | Age-standardized rate (per 100,000) | 2.34 (0.74 to 4.05) | 2.6 (0.96 to 4.56) | 2.12 (0.61 to 3.8) | 4.78 (1.47 to 8.32) | 5.09 (1.44 to 8.84) | 4.45 (1.26 to 7.72) | 103.74% (54.24% to 157.95%) | 95.26% (29.7% to 157.11%) | 110.47% (53.61% to 199.0%) |
|  |  | YLDs | All age number | 4.45 (1.41 to 8.21) | 2.87 (0.83 to 5.39) | 1.58 (0.53 to 2.83) | 30.91 (9.15 to 54.68) | 21.34 (5.61 to 39.49) | 9.57 (3.33 to 16.46) | 595.02% (412.48% to 822.06%) | 644.42% (402.07% to 946.61%) | 505.45% (330.06% to 776.92%) |
|  |  |  | Age-standardized rate (per 100,000) | 1.2 (0.37 to 2.22) | 1.61 (0.44 to 3.03) | 0.84 (0.28 to 1.49) | 4.06 (1.18 to 7.3) | 5.46 (1.41 to 10.12) | 2.54 (0.85 to 4.39) | 238.81% (150.55% to 347.85%) | 238.77% (130.75% to 372.87%) | 203.41% (117.61% to 342.69%) |
|  |  | YLLs | All age number | 211.36 (77.81 to 356.1) | 114.31 (46.48 to 200.07) | 97.05 (32.1 to 168.28) | 856.73 (284.1 to 1467.06) | 467.4 (140.16 to 802.75) | 389.33 (126.41 to 657.37) | 305.35% (195.71% to 422.75%) | 308.88% (161.78% to 474.16%) | 301.18% (178.06% to 476.44%) |
|  |  |  | Age-standardized rate (per 100,000) | 55.49 (19.51 to 93.94) | 63.16 (24.91 to 110.61) | 48.69 (15.64 to 85.85) | 110.98 (35.88 to 192.46) | 118.56 (34.86 to 206.63) | 102.67 (31.92 to 174.06) | 99.99% (47.73% to 157.81%) | 87.71% (21.38% to 160.2%) | 110.85% (47.23% to 200.28%) |
| Tehran | All risk factors | DALYs | All age number | 28711.52 (20847.0 to 38759.07) | 11579.93 (6963.35 to 17204.17) | 17131.59 (11848.75 to 23335.2) | 88928.61 (62922.67 to 117536.19) | 37003.28 (20611.14 to 53475.82) | 51925.34 (38364.61 to 70631.81) | 209.73% (131.0% to 316.56%) | 219.55% (112.1% to 362.16%) | 203.1% (108.98% to 344.21%) |
|  |  |  | Age-standardized rate (per 100,000) | 638.6 (462.61 to 865.42) | 512.35 (312.18 to 758.21) | 764.8 (532.74 to 1041.44) | 570.33 (405.14 to 755.4) | 474.04 (265.28 to 686.13) | 662.28 (490.15 to 904.02) | -10.69% (-33.06% to 19.62%) | -7.48% (-37.53% to 33.05%) | -13.4% (-39.52% to 24.61%) |
|  |  | Deaths | All age number | 992.63 (720.82 to 1344.98) | 377.65 (231.96 to 562.8) | 614.98 (429.87 to 835.73) | 3580.69 (2578.12 to 4782.8) | 1366.43 (781.81 to 1963.79) | 2214.25 (1652.9 to 2983.69) | 260.73% (170.31% to 380.43%) | 261.82% (144.01% to 414.99%) | 260.05% (153.76% to 422.41%) |
|  |  |  | Age-standardized rate (per 100,000) | 26.09 (19.02 to 35.41) | 19.72 (12.13 to 29.39) | 32.75 (22.71 to 44.78) | 24.57 (17.61 to 32.91) | 19.26 (11.08 to 27.57) | 29.49 (22.04 to 39.74) | -5.82% (-28.88% to 21.93%) | -2.36% (-33.43% to 37.82%) | -9.96% (-36.09% to 27.76%) |
|  |  | YLDs | All age number | 649.53 (387.43 to 1037.53) | 369.91 (162.76 to 646.07) | 279.61 (176.09 to 433.33) | 3601.6 (1950.15 to 5602.76) | 2261.1 (961.73 to 3762.83) | 1340.5 (855.91 to 2033.37) | 454.49% (302.81% to 655.37%) | 511.25% (297.3% to 789.64%) | 379.41% (231.04% to 611.85%) |
|  |  |  | Age-standardized rate (per 100,000) | 14.47 (8.75 to 22.63) | 16.1 (7.11 to 27.63) | 13.16 (8.49 to 20.28) | 22.74 (12.52 to 35.36) | 28.14 (11.87 to 46.5) | 17.32 (11.03 to 26.22) | 57.13% (14.26% to 111.56%) | 74.79% (13.85% to 151.29%) | 31.53% (-8.44% to 92.63%) |
|  |  | YLLs | All age number | 28061.99 (20462.81 to 37899.61) | 11210.01 (6810.81 to 16602.41) | 16851.98 (11642.15 to 22944.67) | 85327.02 (60658.41 to 112925.05) | 34742.18 (19551.66 to 50196.99) | 50584.84 (37347.47 to 68988.89) | 204.07% (126.83% to 309.82%) | 209.92% (106.23% to 348.66%) | 200.17% (106.75% to 339.87%) |
|  |  |  | Age-standardized rate (per 100,000) | 624.13 (454.26 to 846.28) | 496.24 (304.23 to 737.69) | 751.63 (524.39 to 1022.99) | 547.59 (390.68 to 727.41) | 445.9 (252.67 to 643.03) | 644.96 (476.36 to 880.22) | -12.26% (-34.24% to 17.81%) | -10.14% (-39.16% to 29.31%) | -14.19% (-40.1% to 23.52%) |
|  | Behavioral risks | DALYs | All age number | 22824.31 (15822.58 to 31717.73) | 8133.79 (4393.89 to 13051.91) | 14690.52 (9847.09 to 20426.35) | 60329.61 (43391.79 to 81560.05) | 20353.54 (10710.28 to 30682.73) | 39976.07 (29847.94 to 54011.14) | 164.32% (102.16% to 251.92%) | 150.23% (67.91% to 262.27%) | 172.12% (89.8% to 298.33%) |
|  |  |  | Age-standardized rate (per 100,000) | 506.96 (354.85 to 697.69) | 352.76 (195.54 to 558.76) | 659.03 (451.73 to 922.91) | 384.45 (279.45 to 519.71) | 254.19 (135.08 to 381.23) | 509.43 (379.74 to 689.34) | -24.17% (-42.09% to -0.03%) | -27.94% (-50.79% to 3.38%) | -22.7% (-45.94% to 11.28%) |
|  |  | Deaths | All age number | 787.17 (553.63 to 1083.76) | 256.83 (143.97 to 405.17) | 530.34 (363.01 to 740.4) | 2412.59 (1794.49 to 3234.88) | 712.23 (385.67 to 1061.76) | 1700.36 (1266.71 to 2316.15) | 206.49% (134.73% to 304.34%) | 177.32% (95.08% to 298.56%) | 220.62% (124.57% to 358.56%) |
|  |  |  | Age-standardized rate (per 100,000) | 20.57 (14.66 to 28.02) | 13.19 (7.38 to 20.55) | 28.19 (19.43 to 39.64) | 16.49 (12.28 to 22.15) | 9.87 (5.41 to 14.66) | 22.63 (16.86 to 30.8) | -19.81% (-38.4% to 4.09%) | -25.21% (-46.85% to 6.14%) | -19.72% (-43.53% to 11.11%) |
|  |  | YLDs | All age number | 505.4 (286.11 to 821.72) | 262.46 (101.84 to 485.53) | 242.94 (150.16 to 369.18) | 2366.16 (1177.43 to 3807.31) | 1329.46 (442.51 to 2417.12) | 1036.7 (664.53 to 1563.28) | 368.18% (248.94% to 521.63%) | 406.53% (220.3% to 646.15%) | 326.74% (196.02% to 526.51%) |
|  |  |  | Age-standardized rate (per 100,000) | 11.14 (6.52 to 17.55) | 11.03 (4.29 to 20.22) | 11.45 (7.21 to 17.23) | 14.75 (7.53 to 23.54) | 16.0 (5.37 to 28.6) | 13.41 (8.61 to 20.09) | 32.4% (-1.33% to 76.35%) | 45.14% (-5.23% to 112.73%) | 17.15% (-17.97% to 67.83%) |
|  |  | YLLs | All age number | 22318.92 (15500.95 to 30860.59) | 7871.33 (4275.13 to 12582.36) | 14447.58 (9685.62 to 20147.22) | 57963.45 (42123.73 to 78211.52) | 19024.09 (10264.25 to 28329.99) | 38939.37 (28981.21 to 52683.44) | 159.71% (98.66% to 244.52%) | 141.69% (62.18% to 249.72%) | 169.52% (87.51% to 294.44%) |
|  |  |  | Age-standardized rate (per 100,000) | 495.82 (347.75 to 680.87) | 341.73 (191.31 to 541.14) | 647.58 (443.64 to 906.7) | 369.71 (270.92 to 497.26) | 238.18 (129.41 to 353.88) | 496.02 (369.28 to 672.14) | -25.44% (-43.29% to -1.51%) | -30.3% (-52.46% to 0.43%) | -23.4% (-46.44% to 10.31%) |
|  | Environmental/occupational risks | DALYs | All age number | 3615.63 (2170.85 to 5469.07) | 757.4 (412.92 to 1272.21) | 2858.22 (1736.32 to 4499.38) | 10701.58 (7105.68 to 15697.67) | 2865.4 (1706.5 to 4716.2) | 7836.18 (5077.69 to 11780.2) | 195.98% (104.47% to 334.96%) | 278.32% (126.21% to 514.65%) | 174.16% (77.07% to 321.48%) |
|  |  |  | Age-standardized rate (per 100,000) | 79.82 (47.65 to 122.16) | 34.71 (19.12 to 59.04) | 122.81 (73.47 to 196.82) | 68.77 (45.28 to 100.91) | 37.71 (22.39 to 62.78) | 99.05 (63.87 to 149.08) | -13.84% (-40.4% to 26.19%) | 8.65% (-34.28% to 74.09%) | -19.35% (-48.57% to 25.62%) |
|  |  | Deaths | All age number | 126.15 (75.43 to 193.79) | 26.61 (14.74 to 45.24) | 99.54 (59.94 to 159.25) | 441.23 (284.6 to 654.62) | 118.2 (70.42 to 194.64) | 323.03 (207.09 to 485.51) | 249.76% (143.83% to 411.14%) | 344.14% (167.89% to 607.38%) | 224.52% (107.4% to 399.31%) |
|  |  |  | Age-standardized rate (per 100,000) | 3.26 (1.92 to 5.16) | 1.46 (0.8 to 2.48) | 5.06 (3.01 to 8.17) | 3.02 (1.94 to 4.5) | 1.71 (1.01 to 2.79) | 4.26 (2.74 to 6.42) | -7.35% (-34.44% to 35.61%) | 17.25% (-29.3% to 85.31%) | -15.69% (-45.25% to 28.45%) |
|  |  | YLDs | All age number | 30.93 (17.04 to 53.32) | 6.3 (3.0 to 11.45) | 24.63 (13.36 to 43.36) | 105.13 (61.98 to 169.19) | 26.89 (14.08 to 46.34) | 78.24 (45.12 to 127.0) | 239.85% (127.07% to 408.11%) | 326.51% (153.27% to 602.75%) | 217.66% (95.3% to 416.53%) |
|  |  |  | Age-standardized rate (per 100,000) | 0.73 (0.39 to 1.27) | 0.31 (0.15 to 0.58) | 1.13 (0.6 to 2.04) | 0.69 (0.41 to 1.12) | 0.37 (0.19 to 0.64) | 1.01 (0.58 to 1.65) | -5.01% (-37.2% to 42.85%) | 17.45% (-32.99% to 95.1%) | -11.02% (-45.87% to 45.35%) |
|  |  | YLLs | All age number | 3584.69 (2153.72 to 5415.73) | 751.1 (409.54 to 1264.15) | 2833.59 (1721.77 to 4453.07) | 10596.46 (7026.52 to 15573.74) | 2838.51 (1690.99 to 4674.34) | 7757.94 (5018.49 to 11669.43) | 195.6% (104.13% to 334.47%) | 277.91% (125.82% to 513.84%) | 173.78% (76.7% to 320.72%) |
|  |  |  | Age-standardized rate (per 100,000) | 79.09 (47.13 to 121.19) | 34.4 (18.97 to 58.51) | 121.68 (72.65 to 194.77) | 68.08 (44.82 to 100.09) | 37.35 (22.19 to 62.2) | 98.04 (63.12 to 147.7) | -13.92% (-40.46% to 26.0%) | 8.57% (-34.35% to 73.87%) | -19.42% (-48.61% to 25.39%) |
|  | Metabolic risks | DALYs | All age number | 5593.2 (1809.3 to 9541.42) | 3441.33 (1160.34 to 5943.48) | 2151.88 (769.61 to 3880.02) | 29833.77 (8744.41 to 52214.77) | 17116.56 (4615.61 to 28935.54) | 12717.21 (3964.23 to 23211.69) | 433.39% (281.85% to 629.43%) | 397.38% (205.13% to 626.92%) | 490.98% (282.27% to 764.03%) |
|  |  |  | Age-standardized rate (per 100,000) | 127.3 (37.73 to 219.61) | 161.1 (49.47 to 284.46) | 95.88 (31.24 to 175.32) | 194.28 (56.29 to 341.17) | 226.26 (60.89 to 384.84) | 163.13 (49.93 to 297.74) | 52.61% (12.29% to 105.61%) | 40.45% (-9.52% to 103.87%) | 70.15% (12.43% to 143.28%) |
|  |  | Deaths | All age number | 198.62 (57.61 to 342.02) | 121.75 (37.24 to 215.47) | 76.86 (24.58 to 140.83) | 1217.76 (349.87 to 2159.44) | 669.39 (182.28 to 1139.21) | 548.37 (159.87 to 1016.61) | 513.12% (353.63% to 722.47%) | 449.79% (257.26% to 698.63%) | 613.44% (369.02% to 928.44%) |
|  |  |  | Age-standardized rate (per 100,000) | 5.36 (1.49 to 9.36) | 6.59 (1.98 to 11.85) | 4.17 (1.23 to 7.63) | 8.42 (2.4 to 15.01) | 9.59 (2.61 to 16.4) | 7.34 (2.11 to 13.62) | 57.27% (18.6% to 110.01%) | 45.66% (-0.27% to 109.38%) | 75.8% (18.06% to 150.06%) |
|  |  | YLDs | All age number | 163.37 (53.47 to 298.44) | 121.36 (38.19 to 232.13) | 42.01 (14.47 to 79.58) | 1466.77 (369.19 to 2729.64) | 1084.52 (226.52 to 2031.58) | 382.25 (127.14 to 731.3) | 797.84% (528.84% to 1153.96%) | 793.63% (447.44% to 1233.9%) | 809.98% (522.66% to 1217.48%) |
|  |  |  | Age-standardized rate (per 100,000) | 3.81 (1.11 to 7.14) | 5.77 (1.62 to 11.22) | 2.0 (0.65 to 3.76) | 9.52 (2.4 to 17.63) | 14.16 (2.97 to 26.69) | 4.94 (1.62 to 9.46) | 149.7% (80.76% to 239.45%) | 145.28% (59.65% to 264.68%) | 147.46% (66.1% to 251.63%) |
|  |  | YLLs | All age number | 5429.84 (1753.46 to 9256.81) | 3319.97 (1116.89 to 5717.76) | 2109.87 (751.87 to 3802.85) | 28367.0 (8370.36 to 49847.13) | 16032.05 (4379.31 to 27082.5) | 12334.95 (3825.67 to 22430.38) | 422.43% (272.26% to 615.82%) | 382.9% (197.7% to 608.65%) | 484.63% (278.23% to 755.37%) |
|  |  |  | Age-standardized rate (per 100,000) | 123.49 (36.56 to 212.43) | 155.33 (47.79 to 274.08) | 93.88 (30.44 to 171.61) | 184.76 (53.92 to 325.28) | 212.11 (57.85 to 359.83) | 158.19 (48.15 to 288.13) | 49.62% (10.34% to 102.45%) | 36.55% (-11.83% to 99.57%) | 68.51% (10.83% to 141.01%) |
| West Azarbayejan | All risk factors | DALYs | All age number | 8800.93 (6494.76 to 12548.81) | 2351.98 (1437.55 to 3819.32) | 6448.95 (4555.58 to 9317.48) | 20427.64 (15653.57 to 26367.27) | 6485.04 (4256.4 to 9216.51) | 13942.6 (10766.62 to 18203.83) | 132.11% (76.34% to 214.87%) | 175.73% (94.68% to 294.66%) | 116.2% (51.12% to 226.37%) |
|  |  |  | Age-standardized rate (per 100,000) | 800.45 (593.55 to 1133.07) | 426.21 (253.63 to 693.2) | 1131.42 (810.22 to 1622.26) | 672.04 (515.53 to 867.65) | 411.77 (270.2 to 581.44) | 947.43 (726.13 to 1232.7) | -16.04% (-35.01% to 13.01%) | -3.39% (-31.18% to 37.25%) | -16.26% (-40.63% to 22.51%) |
|  |  | Deaths | All age number | 300.16 (222.48 to 426.25) | 72.62 (43.72 to 117.56) | 227.54 (161.87 to 325.51) | 767.4 (591.43 to 989.16) | 233.57 (152.25 to 331.44) | 533.83 (409.94 to 693.21) | 155.66% (95.59% to 244.92%) | 221.63% (125.87% to 356.82%) | 134.61% (65.04% to 245.32%) |
|  |  |  | Age-standardized rate (per 100,000) | 33.44 (24.6 to 47.2) | 16.79 (10.08 to 27.24) | 48.71 (35.21 to 69.12) | 29.43 (22.59 to 37.79) | 17.37 (11.31 to 24.43) | 42.43 (32.38 to 54.84) | -11.99% (-32.06% to 16.13%) | 3.42% (-25.74% to 45.47%) | -12.89% (-36.95% to 26.06%) |
|  |  | YLDs | All age number | 116.78 (74.74 to 168.53) | 42.43 (22.2 to 70.55) | 74.34 (47.75 to 112.69) | 463.42 (285.17 to 681.91) | 233.45 (114.47 to 374.72) | 229.98 (153.39 to 332.26) | 296.85% (194.48% to 422.53%) | 450.16% (287.04% to 672.9%) | 209.35% (113.59% to 361.11%) |
|  |  |  | Age-standardized rate (per 100,000) | 11.01 (7.22 to 15.71) | 7.77 (4.09 to 12.76) | 13.85 (8.94 to 20.8) | 14.99 (9.4 to 21.54) | 14.12 (7.0 to 22.65) | 16.13 (10.83 to 23.26) | 36.11% (1.53% to 80.03%) | 81.64% (29.62% to 152.88%) | 16.45% (-17.68% to 69.96%) |
|  |  | YLLs | All age number | 8684.16 (6403.55 to 12392.52) | 2309.55 (1410.32 to 3764.25) | 6374.61 (4506.47 to 9210.16) | 19964.21 (15345.45 to 25747.06) | 6251.59 (4127.47 to 8875.74) | 13712.62 (10595.9 to 17948.57) | 129.89% (74.25% to 211.62%) | 170.68% (91.62% to 288.17%) | 115.11% (50.18% to 225.08%) |
|  |  |  | Age-standardized rate (per 100,000) | 789.44 (584.58 to 1118.29) | 418.44 (249.5 to 682.09) | 1117.57 (800.31 to 1603.66) | 657.04 (505.31 to 849.1) | 397.65 (261.63 to 562.59) | 931.3 (715.16 to 1213.8) | -16.77% (-35.6% to 12.0%) | -4.97% (-32.42% to 35.31%) | -16.67% (-41.0% to 21.92%) |
|  | Behavioral risks | DALYs | All age number | 7774.49 (5518.79 to 11565.95) | 1882.43 (1056.05 to 3212.45) | 5892.06 (4060.58 to 8609.09) | 16038.78 (11771.52 to 21437.65) | 4167.88 (2468.74 to 6622.51) | 11870.9 (8880.08 to 16055.75) | 106.3% (54.79% to 174.56%) | 121.41% (56.88% to 218.82%) | 101.47% (38.64% to 200.51%) |
|  |  |  | Age-standardized rate (per 100,000) | 707.81 (504.55 to 1047.52) | 339.22 (189.99 to 586.44) | 1033.53 (725.88 to 1508.19) | 525.64 (385.58 to 702.75) | 259.15 (154.69 to 411.53) | 807.34 (599.23 to 1100.81) | -25.74% (-43.94% to -0.89%) | -23.6% (-44.58% to 8.03%) | -21.89% (-45.06% to 13.57%) |
|  |  | Deaths | All age number | 266.33 (189.3 to 392.77) | 57.7 (32.3 to 99.85) | 208.62 (145.28 to 303.52) | 601.26 (440.98 to 804.8) | 145.04 (87.02 to 228.26) | 456.21 (336.99 to 623.32) | 125.76% (68.8% to 203.25%) | 151.36% (84.13% to 257.63%) | 118.68% (52.62% to 219.76%) |
|  |  |  | Age-standardized rate (per 100,000) | 29.5 (21.31 to 43.11) | 13.23 (7.26 to 22.9) | 44.39 (31.52 to 64.23) | 22.92 (16.67 to 30.63) | 10.62 (6.41 to 16.76) | 36.16 (26.46 to 48.83) | -22.3% (-40.93% to 1.99%) | -19.73% (-40.23% to 14.37%) | -18.54% (-41.62% to 15.21%) |
|  |  | YLDs | All age number | 103.09 (64.76 to 150.72) | 34.56 (16.37 to 59.75) | 68.53 (43.21 to 105.7) | 351.58 (210.23 to 528.09) | 157.41 (57.64 to 270.05) | 194.16 (128.77 to 280.75) | 241.04% (145.97% to 355.76%) | 355.44% (188.53% to 551.61%) | 183.34% (92.82% to 318.61%) |
|  |  |  | Age-standardized rate (per 100,000) | 9.69 (6.2 to 14.04) | 6.21 (2.95 to 10.54) | 12.72 (8.11 to 19.2) | 11.27 (6.94 to 16.53) | 9.2 (3.54 to 15.53) | 13.62 (9.04 to 19.78) | 16.3% (-13.99% to 53.47%) | 48.22% (-2.12% to 108.46%) | 7.07% (-24.22% to 55.07%) |
|  |  | YLLs | All age number | 7671.4 (5451.57 to 11428.1) | 1847.87 (1040.67 to 3159.49) | 5823.53 (4012.37 to 8512.27) | 15687.2 (11579.15 to 21050.44) | 4010.47 (2398.19 to 6372.28) | 11676.73 (8746.79 to 15798.26) | 104.49% (53.32% to 171.83%) | 117.03% (54.15% to 212.95%) | 100.51% (38.0% to 199.3%) |
|  |  |  | Age-standardized rate (per 100,000) | 698.12 (496.98 to 1034.36) | 333.01 (187.24 to 577.19) | 1020.81 (715.47 to 1487.65) | 514.38 (378.39 to 689.13) | 249.95 (149.45 to 396.7) | 793.72 (590.85 to 1082.56) | -26.32% (-44.52% to -1.67%) | -24.94% (-45.6% to 7.13%) | -22.25% (-45.33% to 12.97%) |
|  | Environmental/occupational risks | DALYs | All age number | 1279.57 (716.37 to 2089.76) | 224.54 (112.95 to 387.47) | 1055.03 (581.2 to 1781.42) | 3116.79 (1924.14 to 4562.5) | 770.89 (409.44 to 1275.18) | 2345.9 (1447.89 to 3484.13) | 143.58% (58.99% to 273.65%) | 243.31% (94.55% to 484.76%) | 122.35% (33.64% to 265.55%) |
|  |  |  | Age-standardized rate (per 100,000) | 115.73 (64.63 to 186.71) | 42.85 (21.86 to 72.76) | 180.85 (98.8 to 305.42) | 101.18 (61.77 to 150.47) | 50.17 (26.36 to 82.45) | 154.22 (94.08 to 231.58) | -12.57% (-42.87% to 34.32%) | 17.09% (-32.57% to 101.81%) | -14.73% (-48.13% to 39.81%) |
|  |  | Deaths | All age number | 43.92 (24.52 to 71.6) | 7.35 (3.75 to 12.58) | 36.58 (20.02 to 61.58) | 116.12 (70.63 to 172.6) | 29.62 (15.67 to 48.66) | 86.5 (52.4 to 130.44) | 164.35% (74.59% to 302.76%) | 303.17% (131.61% to 594.15%) | 136.47% (43.4% to 286.21%) |
|  |  |  | Age-standardized rate (per 100,000) | 4.87 (2.67 to 7.81) | 1.82 (0.92 to 3.11) | 7.67 (4.18 to 12.95) | 4.37 (2.61 to 6.57) | 2.25 (1.18 to 3.75) | 6.62 (3.95 to 10.17) | -10.27% (-40.43% to 37.67%) | 23.9% (-29.12% to 121.56%) | -13.75% (-46.97% to 37.84%) |
|  |  | YLDs | All age number | 10.33 (5.72 to 18.21) | 1.78 (0.9 to 3.12) | 8.55 (4.62 to 15.56) | 27.44 (14.62 to 43.98) | 6.62 (3.1 to 11.75) | 20.82 (11.2 to 33.81) | 165.55% (67.71% to 315.49%) | 271.12% (109.73% to 539.9%) | 143.53% (42.25% to 311.61%) |
|  |  |  | Age-standardized rate (per 100,000) | 1.01 (0.56 to 1.81) | 0.38 (0.19 to 0.68) | 1.58 (0.85 to 2.84) | 0.94 (0.49 to 1.52) | 0.46 (0.21 to 0.83) | 1.44 (0.77 to 2.35) | -7.31% (-39.79% to 45.91%) | 20.49% (-31.99% to 110.95%) | -8.92% (-45.46% to 49.02%) |
|  |  | YLLs | All age number | 1269.24 (710.63 to 2073.87) | 222.76 (111.98 to 384.75) | 1046.48 (576.71 to 1768.56) | 3089.35 (1903.96 to 4528.85) | 764.27 (405.38 to 1265.8) | 2325.08 (1436.93 to 3448.91) | 143.4% (58.82% to 273.58%) | 243.09% (94.41% to 485.09%) | 122.18% (33.38% to 265.42%) |
|  |  |  | Age-standardized rate (per 100,000) | 114.72 (64.0 to 185.19) | 42.47 (21.66 to 72.08) | 179.27 (97.93 to 303.07) | 100.24 (61.13 to 149.04) | 49.71 (26.08 to 81.82) | 152.78 (93.27 to 229.46) | -12.62% (-42.95% to 34.11%) | 17.06% (-32.59% to 101.75%) | -14.78% (-48.21% to 39.67%) |
|  | Metabolic risks | DALYs | All age number | 665.32 (275.41 to 1065.09) | 354.35 (159.42 to 593.43) | 310.97 (112.68 to 552.48) | 3853.12 (1254.66 to 6525.11) | 2054.0 (694.63 to 3463.31) | 1799.12 (517.06 to 3120.04) | 479.14% (299.89% to 643.86%) | 479.65% (253.22% to 703.74%) | 478.56% (284.95% to 739.39%) |
|  |  |  | Age-standardized rate (per 100,000) | 59.45 (21.97 to 97.4) | 65.17 (27.03 to 112.33) | 54.25 (17.42 to 98.48) | 129.65 (40.31 to 222.65) | 135.9 (44.7 to 234.47) | 123.5 (33.43 to 217.11) | 118.07% (56.7% to 174.21%) | 108.55% (37.61% to 186.6%) | 127.66% (56.47% to 226.62%) |
|  |  | Deaths | All age number | 21.9 (8.06 to 36.02) | 11.26 (4.64 to 19.23) | 10.63 (3.37 to 19.42) | 147.4 (44.2 to 255.32) | 78.66 (24.79 to 135.6) | 68.74 (17.91 to 121.91) | 573.19% (379.67% to 747.48%) | 598.34% (354.39% to 858.3%) | 546.55% (338.39% to 831.3%) |
|  |  |  | Age-standardized rate (per 100,000) | 2.47 (0.81 to 4.14) | 2.62 (0.97 to 4.61) | 2.34 (0.65 to 4.2) | 5.78 (1.68 to 10.08) | 5.98 (1.83 to 10.27) | 5.58 (1.38 to 9.91) | 134.13% (77.23% to 187.73%) | 128.83% (55.87% to 213.35%) | 138.87% (68.96% to 238.87%) |
|  |  | YLDs | All age number | 12.51 (4.04 to 21.72) | 7.9 (2.56 to 14.11) | 4.61 (1.5 to 8.5) | 125.4 (39.31 to 209.0) | 84.8 (23.35 to 145.06) | 40.6 (14.19 to 74.19) | 902.32% (616.21% to 1213.66%) | 973.9% (600.06% to 1399.03%) | 779.84% (501.76% to 1202.22%) |
|  |  |  | Age-standardized rate (per 100,000) | 1.19 (0.35 to 2.09) | 1.56 (0.48 to 2.77) | 0.87 (0.26 to 1.63) | 4.18 (1.21 to 7.08) | 5.48 (1.5 to 9.47) | 2.86 (0.96 to 5.3) | 250.95% (161.87% to 351.02%) | 252.26% (143.29% to 382.84%) | 227.03% (127.91% to 382.47%) |
|  |  | YLLs | All age number | 652.81 (270.83 to 1045.31) | 346.45 (156.15 to 578.9) | 306.35 (111.12 to 545.09) | 3727.71 (1217.13 to 6342.95) | 1969.2 (668.2 to 3335.18) | 1758.52 (503.64 to 3052.52) | 471.03% (294.78% to 634.99%) | 468.39% (245.31% to 689.3%) | 474.02% (282.27% to 732.33%) |
|  |  |  | Age-standardized rate (per 100,000) | 58.26 (21.61 to 95.46) | 63.61 (26.5 to 109.44) | 53.38 (17.18 to 97.04) | 125.47 (39.13 to 215.95) | 130.42 (43.02 to 225.04) | 120.64 (32.51 to 212.41) | 115.35% (54.78% to 171.15%) | 105.04% (35.27% to 181.97%) | 126.03% (55.3% to 224.81%) |
| Yazd | All risk factors | DALYs | All age number | 2164.48 (1648.46 to 2819.15) | 775.17 (517.09 to 1144.03) | 1389.3 (998.66 to 1888.18) | 5346.19 (3941.89 to 6911.72) | 2067.92 (1261.85 to 3021.59) | 3278.27 (2422.32 to 4341.01) | 147.0% (88.72% to 237.96%) | 166.77% (84.96% to 279.28%) | 135.96% (61.45% to 267.32%) |
|  |  |  | Age-standardized rate (per 100,000) | 572.91 (437.25 to 748.42) | 406.96 (270.82 to 595.45) | 755.05 (548.47 to 1019.67) | 522.02 (389.04 to 672.98) | 405.43 (249.4 to 590.46) | 634.03 (467.62 to 836.5) | -8.88% (-30.07% to 24.85%) | -0.38% (-30.6% to 40.58%) | -16.03% (-41.88% to 28.65%) |
|  |  | Deaths | All age number | 76.07 (58.06 to 99.0) | 25.89 (17.3 to 37.5) | 50.18 (36.15 to 68.17) | 207.39 (154.14 to 266.46) | 78.05 (48.55 to 113.09) | 129.34 (95.89 to 168.13) | 172.63% (108.82% to 274.32%) | 201.41% (112.53% to 320.24%) | 157.78% (79.09% to 295.51%) |
|  |  |  | Age-standardized rate (per 100,000) | 22.84 (17.42 to 29.61) | 15.29 (10.34 to 21.91) | 31.76 (23.34 to 42.92) | 22.33 (16.54 to 28.63) | 16.82 (10.39 to 24.38) | 27.79 (20.79 to 35.74) | -2.25% (-24.44% to 33.2%) | 10.0% (-22.15% to 52.75%) | -12.5% (-37.79% to 31.62%) |
|  |  | YLDs | All age number | 38.02 (24.13 to 55.17) | 18.7 (9.43 to 30.82) | 19.32 (12.26 to 28.58) | 169.12 (94.52 to 271.08) | 100.52 (42.23 to 175.76) | 68.6 (44.52 to 100.22) | 344.82% (233.99% to 480.23%) | 437.51% (263.97% to 651.96%) | 255.1% (149.63% to 441.99%) |
|  |  |  | Age-standardized rate (per 100,000) | 10.24 (6.57 to 14.72) | 9.83 (4.99 to 16.11) | 10.96 (7.05 to 15.95) | 16.17 (9.2 to 25.5) | 19.08 (8.2 to 32.95) | 13.54 (8.92 to 19.65) | 57.85% (20.01% to 104.2%) | 93.98% (34.23% to 168.27%) | 23.52% (-12.09% to 85.74%) |
|  |  | YLLs | All age number | 2126.46 (1620.37 to 2772.66) | 756.47 (506.39 to 1116.67) | 1369.99 (984.19 to 1865.52) | 5177.07 (3841.87 to 6661.32) | 1967.4 (1221.39 to 2868.33) | 3209.67 (2372.53 to 4248.49) | 143.46% (85.65% to 232.82%) | 160.08% (82.17% to 266.52%) | 134.28% (60.2% to 264.84%) |
|  |  |  | Age-standardized rate (per 100,000) | 562.66 (429.54 to 736.01) | 397.13 (265.05 to 580.45) | 744.08 (540.64 to 1007.04) | 505.86 (376.88 to 650.97) | 386.36 (241.15 to 561.28) | 620.49 (457.89 to 816.03) | -10.1% (-31.12% to 23.23%) | -2.71% (-32.01% to 37.31%) | -16.61% (-42.34% to 27.47%) |
|  | Behavioral risks | DALYs | All age number | 1772.05 (1306.82 to 2391.16) | 565.58 (351.78 to 858.94) | 1206.47 (864.56 to 1673.66) | 3678.03 (2582.31 to 4734.23) | 1166.78 (634.92 to 1814.14) | 2511.25 (1841.58 to 3271.03) | 107.56% (53.02% to 179.91%) | 106.3% (38.18% to 188.36%) | 108.15% (41.65% to 215.59%) |
|  |  |  | Age-standardized rate (per 100,000) | 468.57 (347.43 to 629.98) | 296.3 (184.45 to 449.68) | 656.16 (474.33 to 899.93) | 356.08 (254.0 to 455.18) | 220.03 (123.51 to 338.04) | 486.72 (356.97 to 633.7) | -24.01% (-43.23% to 1.55%) | -25.74% (-48.7% to 1.52%) | -25.82% (-49.67% to 9.33%) |
|  |  | Deaths | All age number | 62.3 (46.23 to 82.6) | 18.53 (11.79 to 28.0) | 43.77 (31.38 to 60.65) | 140.55 (101.93 to 179.21) | 41.28 (24.18 to 61.57) | 99.27 (72.47 to 129.17) | 125.6% (69.06% to 201.0%) | 122.78% (58.76% to 204.24%) | 126.8% (55.5% to 235.04%) |
|  |  |  | Age-standardized rate (per 100,000) | 18.56 (13.87 to 24.63) | 10.9 (6.9 to 16.4) | 27.54 (19.94 to 37.48) | 15.01 (11.04 to 19.19) | 8.68 (5.17 to 12.83) | 21.29 (15.56 to 27.65) | -19.12% (-38.74% to 7.16%) | -20.39% (-41.83% to 7.16%) | -22.69% (-46.55% to 14.24%) |
|  |  | YLDs | All age number | 31.2 (18.98 to 46.06) | 14.09 (6.42 to 24.45) | 17.11 (10.78 to 25.61) | 116.8 (60.79 to 186.43) | 63.61 (20.76 to 123.96) | 53.19 (34.35 to 77.93) | 274.37% (171.12% to 383.35%) | 351.61% (175.61% to 559.56%) | 210.8% (116.3% to 367.08%) |
|  |  |  | Age-standardized rate (per 100,000) | 8.36 (5.12 to 12.38) | 7.36 (3.35 to 12.82) | 9.68 (6.18 to 14.21) | 10.87 (5.9 to 16.76) | 11.33 (3.8 to 21.66) | 10.54 (6.86 to 15.41) | 29.93% (-4.61% to 65.79%) | 53.93% (-2.41% to 121.58%) | 8.85% (-23.4% to 59.36%) |
|  |  | YLLs | All age number | 1740.85 (1285.14 to 2347.86) | 551.49 (345.11 to 837.79) | 1189.36 (853.12 to 1649.69) | 3561.23 (2519.83 to 4534.71) | 1103.17 (616.2 to 1705.0) | 2458.06 (1801.09 to 3204.91) | 104.57% (50.86% to 175.95%) | 100.03% (35.16% to 178.02%) | 106.67% (40.46% to 213.26%) |
|  |  |  | Age-standardized rate (per 100,000) | 460.2 (342.06 to 616.79) | 288.94 (180.95 to 439.43) | 646.48 (467.54 to 886.04) | 345.21 (247.03 to 439.07) | 208.7 (119.97 to 318.8) | 476.18 (349.17 to 620.77) | -24.99% (-43.97% to 0.33%) | -27.77% (-49.32% to -1.57%) | -26.34% (-50.09% to 8.59%) |
|  | Environmental/occupational risks | DALYs | All age number | 367.84 (225.19 to 558.14) | 83.01 (45.01 to 138.68) | 284.83 (171.71 to 441.56) | 897.54 (587.93 to 1282.12) | 260.89 (154.77 to 385.58) | 636.65 (410.7 to 930.96) | 144.01% (60.6% to 284.37%) | 214.29% (76.55% to 466.99%) | 123.52% (34.92% to 270.26%) |
|  |  |  | Age-standardized rate (per 100,000) | 97.08 (58.77 to 147.99) | 44.88 (24.38 to 74.1) | 151.98 (90.61 to 237.36) | 87.97 (57.15 to 125.09) | 52.3 (30.91 to 77.5) | 121.8 (78.33 to 178.87) | -9.39% (-40.45% to 40.53%) | 16.54% (-33.86% to 111.55%) | -19.86% (-51.29% to 30.16%) |
|  |  | Deaths | All age number | 13.19 (7.96 to 19.84) | 3.01 (1.64 to 4.93) | 10.19 (6.11 to 15.92) | 35.6 (22.97 to 50.63) | 10.82 (6.48 to 16.14) | 24.78 (15.64 to 36.04) | 169.87% (79.47% to 314.49%) | 260.04% (106.25% to 551.33%) | 143.25% (50.65% to 292.35%) |
|  |  |  | Age-standardized rate (per 100,000) | 3.94 (2.39 to 6.02) | 1.86 (1.01 to 3.03) | 6.32 (3.78 to 10.04) | 3.84 (2.46 to 5.46) | 2.38 (1.41 to 3.59) | 5.27 (3.3 to 7.6) | -2.71% (-35.44% to 46.95%) | 27.99% (-25.75% to 130.34%) | -16.62% (-47.71% to 31.43%) |
|  |  | YLDs | All age number | 3.14 (1.68 to 5.21) | 0.7 (0.32 to 1.22) | 2.44 (1.24 to 4.01) | 8.21 (4.85 to 12.6) | 2.38 (1.27 to 3.84) | 5.83 (3.45 to 9.16) | 161.62% (67.66% to 326.42%) | 240.59% (82.25% to 538.42%) | 139.02% (43.72% to 323.51%) |
|  |  |  | Age-standardized rate (per 100,000) | 0.87 (0.47 to 1.42) | 0.4 (0.19 to 0.69) | 1.38 (0.73 to 2.26) | 0.84 (0.5 to 1.29) | 0.5 (0.26 to 0.8) | 1.16 (0.69 to 1.83) | -3.73% (-37.07% to 55.04%) | 24.66% (-31.26% to 136.67%) | -15.72% (-49.45% to 45.47%) |
|  |  | YLLs | All age number | 364.7 (223.36 to 552.88) | 82.31 (44.7 to 137.61) | 282.39 (170.36 to 438.02) | 889.33 (582.66 to 1267.72) | 258.51 (153.38 to 382.91) | 630.82 (406.23 to 922.58) | 143.85% (60.53% to 284.22%) | 214.07% (76.46% to 466.44%) | 123.39% (34.81% to 269.89%) |
|  |  |  | Age-standardized rate (per 100,000) | 96.22 (58.28 to 146.76) | 44.48 (24.2 to 73.47) | 150.61 (89.89 to 235.08) | 87.14 (56.64 to 123.88) | 51.81 (30.61 to 76.84) | 120.64 (77.45 to 177.45) | -9.44% (-40.5% to 40.37%) | 16.46% (-33.88% to 111.34%) | -19.89% (-51.34% to 30.13%) |
|  | Metabolic risks | DALYs | All age number | 304.35 (107.68 to 512.23) | 174.8 (61.79 to 297.43) | 129.54 (42.35 to 224.89) | 1569.89 (476.37 to 2681.14) | 823.25 (242.9 to 1454.98) | 746.64 (251.64 to 1347.96) | 415.82% (274.92% to 571.85%) | 370.96% (195.6% to 565.93%) | 476.36% (281.57% to 786.59%) |
|  |  |  | Age-standardized rate (per 100,000) | 80.62 (27.22 to 136.53) | 91.66 (31.37 to 155.4) | 69.92 (21.6 to 123.39) | 157.59 (45.73 to 270.8) | 171.23 (48.64 to 300.13) | 144.56 (46.01 to 261.46) | 95.47% (43.98% to 150.06%) | 86.82% (18.29% to 159.23%) | 106.74% (40.13% to 209.03%) |
|  |  | Deaths | All age number | 10.69 (3.49 to 18.24) | 6.12 (2.05 to 10.58) | 4.57 (1.38 to 8.14) | 63.05 (17.59 to 108.59) | 33.57 (9.17 to 58.54) | 29.48 (8.86 to 53.39) | 489.96% (338.67% to 644.08%) | 448.67% (252.16% to 654.13%) | 545.24% (335.19% to 864.34%) |
|  |  |  | Age-standardized rate (per 100,000) | 3.26 (1.02 to 5.59) | 3.59 (1.17 to 6.16) | 2.94 (0.84 to 5.33) | 6.91 (1.88 to 11.99) | 7.46 (2.0 to 13.04) | 6.37 (1.83 to 11.59) | 111.91% (59.2% to 164.2%) | 108.07% (35.71% to 183.18%) | 116.78% (51.74% to 213.03%) |
|  |  | YLDs | All age number | 7.03 (1.89 to 12.85) | 4.91 (1.22 to 9.44) | 2.13 (0.67 to 3.99) | 60.17 (16.31 to 110.23) | 41.56 (9.38 to 78.66) | 18.61 (6.13 to 34.22) | 755.72% (536.92% to 1046.95%) | 747.11% (453.09% to 1150.33%) | 775.58% (489.45% to 1245.64%) |
|  |  |  | Age-standardized rate (per 100,000) | 1.92 (0.51 to 3.52) | 2.62 (0.64 to 5.04) | 1.22 (0.37 to 2.27) | 6.13 (1.66 to 11.33) | 8.77 (1.82 to 16.84) | 3.65 (1.15 to 6.68) | 218.61% (137.38% to 318.17%) | 235.33% (121.71% to 390.03%) | 198.46% (105.42% to 340.27%) |
|  |  | YLLs | All age number | 297.31 (105.91 to 501.36) | 169.9 (60.7 to 289.16) | 127.42 (41.8 to 220.41) | 1509.72 (458.51 to 2578.33) | 781.69 (231.66 to 1376.46) | 728.03 (246.06 to 1306.36) | 407.79% (267.47% to 556.86%) | 360.1% (188.82% to 546.54%) | 471.37% (277.28% to 776.92%) |
|  |  |  | Age-standardized rate (per 100,000) | 78.7 (26.78 to 133.19) | 89.04 (30.8 to 151.45) | 68.7 (21.25 to 121.42) | 151.46 (44.03 to 260.08) | 162.46 (46.37 to 283.37) | 140.91 (44.94 to 254.63) | 92.46% (41.61% to 145.34%) | 82.46% (15.05% to 151.45%) | 105.11% (38.73% to 206.49%) |
| Zanjan | All risk factors | DALYs | All age number | 2381.48 (1742.17 to 3365.99) | 626.46 (407.93 to 957.4) | 1755.02 (1229.24 to 2587.78) | 4971.78 (3856.61 to 6463.5) | 1525.43 (979.14 to 2129.01) | 3446.34 (2699.84 to 4479.1) | 108.77% (57.99% to 181.74%) | 143.5% (72.68% to 254.47%) | 96.37% (34.86% to 185.54%) |
|  |  |  | Age-standardized rate (per 100,000) | 522.77 (385.04 to 740.29) | 284.04 (184.14 to 437.23) | 738.44 (524.76 to 1078.06) | 460.98 (357.52 to 597.1) | 269.27 (173.99 to 375.09) | 668.28 (525.96 to 869.91) | -11.82% (-32.75% to 17.52%) | -5.2% (-32.67% to 36.83%) | -9.5% (-36.23% to 28.14%) |
|  |  | Deaths | All age number | 81.93 (59.83 to 115.95) | 19.96 (13.04 to 30.6) | 61.97 (43.58 to 91.27) | 194.08 (151.86 to 251.32) | 56.55 (37.36 to 79.24) | 137.53 (107.32 to 178.32) | 136.89% (81.1% to 214.99%) | 183.26% (103.25% to 306.87%) | 121.95% (56.52% to 218.32%) |
|  |  |  | Age-standardized rate (per 100,000) | 20.7 (15.35 to 29.58) | 10.65 (6.9 to 16.22) | 30.57 (21.85 to 44.56) | 19.32 (15.05 to 24.96) | 10.66 (7.02 to 14.94) | 28.86 (22.56 to 37.43) | -6.66% (-27.82% to 22.31%) | 0.06% (-27.51% to 42.67%) | -5.59% (-32.03% to 31.36%) |
|  |  | YLDs | All age number | 35.21 (22.85 to 51.19) | 12.68 (6.7 to 21.08) | 22.53 (14.27 to 33.65) | 126.8 (78.87 to 185.3) | 59.94 (28.65 to 95.05) | 66.86 (44.88 to 96.24) | 260.17% (173.28% to 374.23%) | 372.77% (235.04% to 574.93%) | 196.79% (107.18% to 317.2%) |
|  |  |  | Age-standardized rate (per 100,000) | 7.97 (5.26 to 11.57) | 5.81 (3.08 to 9.61) | 9.92 (6.4 to 14.75) | 11.65 (7.35 to 16.86) | 10.36 (4.96 to 16.2) | 13.23 (8.95 to 19.11) | 46.3% (11.84% to 91.13%) | 78.23% (26.79% to 153.29%) | 33.35% (-5.83% to 86.89%) |
|  |  | YLLs | All age number | 2346.28 (1714.99 to 3312.66) | 613.78 (400.94 to 935.19) | 1732.49 (1211.97 to 2559.64) | 4844.98 (3755.51 to 6283.05) | 1465.49 (952.11 to 2042.73) | 3379.49 (2651.46 to 4395.75) | 106.5% (56.23% to 178.2%) | 138.76% (69.8% to 247.89%) | 95.06% (33.91% to 183.61%) |
|  |  |  | Age-standardized rate (per 100,000) | 514.81 (378.74 to 729.37) | 278.23 (180.48 to 428.62) | 728.51 (517.05 to 1066.8) | 449.32 (349.34 to 580.68) | 258.91 (169.43 to 360.64) | 655.05 (514.43 to 853.22) | -12.72% (-33.48% to 16.42%) | -6.94% (-33.72% to 34.26%) | -10.08% (-36.65% to 27.36%) |
|  | Behavioral risks | DALYs | All age number | 2069.74 (1504.35 to 3062.63) | 489.08 (284.87 to 798.16) | 1580.66 (1094.31 to 2406.88) | 3853.54 (2983.74 to 5240.43) | 959.74 (563.36 to 1445.66) | 2893.8 (2256.91 to 3907.39) | 86.18% (39.51% to 152.88%) | 96.24% (41.5% to 185.37%) | 83.08% (26.15% to 162.92%) |
|  |  |  | Age-standardized rate (per 100,000) | 454.41 (330.99 to 673.69) | 221.48 (129.79 to 361.67) | 664.13 (465.35 to 1009.3) | 357.18 (277.22 to 483.33) | 167.11 (99.09 to 251.61) | 562.48 (438.67 to 753.24) | -21.4% (-40.52% to 5.14%) | -24.55% (-45.18% to 8.26%) | -15.31% (-40.94% to 20.1%) |
|  |  | Deaths | All age number | 71.49 (52.59 to 106.49) | 15.49 (9.19 to 25.36) | 56.0 (38.85 to 84.71) | 150.36 (115.01 to 203.62) | 34.56 (21.17 to 52.66) | 115.8 (90.18 to 154.43) | 110.32% (59.03% to 181.75%) | 123.09% (62.62% to 214.24%) | 106.79% (45.14% to 192.59%) |
|  |  |  | Age-standardized rate (per 100,000) | 17.96 (13.09 to 26.89) | 8.24 (4.88 to 13.37) | 27.44 (19.37 to 41.03) | 14.95 (11.45 to 20.31) | 6.45 (3.99 to 9.87) | 24.3 (18.98 to 32.27) | -16.75% (-36.02% to 10.59%) | -21.67% (-42.07% to 9.23%) | -11.44% (-36.32% to 24.02%) |
|  |  | YLDs | All age number | 30.66 (19.71 to 45.8) | 10.16 (4.93 to 17.85) | 20.49 (12.85 to 31.31) | 95.6 (56.87 to 144.28) | 39.88 (16.06 to 69.01) | 55.73 (36.97 to 81.73) | 211.86% (134.28% to 313.68%) | 292.43% (161.49% to 468.05%) | 171.92% (91.03% to 284.45%) |
|  |  |  | Age-standardized rate (per 100,000) | 6.92 (4.45 to 10.39) | 4.61 (2.26 to 8.03) | 9.0 (5.69 to 13.65) | 8.72 (5.31 to 12.98) | 6.7 (2.73 to 11.53) | 11.06 (7.34 to 16.22) | 26.06% (-2.99% to 65.74%) | 45.33% (-0.79% to 106.1%) | 22.94% (-13.01% to 71.89%) |
|  |  | YLLs | All age number | 2039.08 (1483.43 to 3022.23) | 478.91 (279.14 to 780.23) | 1560.17 (1078.04 to 2378.82) | 3757.93 (2918.22 to 5117.02) | 919.86 (547.45 to 1387.81) | 2838.07 (2212.6 to 3825.54) | 84.3% (38.51% to 150.27%) | 92.07% (38.06% to 178.91%) | 81.91% (25.41% to 161.78%) |
|  |  |  | Age-standardized rate (per 100,000) | 447.49 (325.03 to 664.06) | 216.87 (127.12 to 354.33) | 655.13 (458.94 to 997.07) | 348.46 (270.99 to 472.44) | 160.41 (96.49 to 243.02) | 551.42 (429.9 to 738.84) | -22.13% (-41.02% to 4.16%) | -26.03% (-46.46% to 5.76%) | -15.83% (-41.27% to 19.49%) |
|  | Environmental/occupational risks | DALYs | All age number | 369.08 (205.15 to 572.88) | 64.97 (28.22 to 125.06) | 304.11 (171.24 to 483.23) | 739.57 (476.39 to 1155.32) | 187.48 (101.12 to 325.43) | 552.09 (357.1 to 858.26) | 100.38% (30.72% to 232.94%) | 188.55% (45.75% to 498.97%) | 81.54% (15.19% to 219.4%) |
|  |  |  | Age-standardized rate (per 100,000) | 80.5 (44.88 to 124.77) | 30.41 (13.16 to 56.87) | 126.38 (71.96 to 196.61) | 68.27 (44.24 to 107.57) | 33.45 (17.96 to 58.25) | 105.43 (68.09 to 165.21) | -15.19% (-44.19% to 39.33%) | 10.01% (-43.47% to 128.23%) | -16.58% (-47.14% to 45.93%) |
|  |  | Deaths | All age number | 12.79 (7.07 to 19.92) | 2.19 (0.95 to 4.12) | 10.6 (5.99 to 16.79) | 28.83 (18.62 to 45.68) | 7.43 (3.93 to 13.3) | 21.4 (13.9 to 33.59) | 125.37% (46.97% to 276.04%) | 238.78% (75.46% to 600.71%) | 101.91% (27.42% to 254.56%) |
|  |  |  | Age-standardized rate (per 100,000) | 3.22 (1.79 to 5.03) | 1.23 (0.53 to 2.28) | 5.19 (2.94 to 7.9) | 2.85 (1.84 to 4.54) | 1.41 (0.75 to 2.55) | 4.43 (2.89 to 6.94) | -11.26% (-42.78% to 45.15%) | 15.42% (-40.65% to 138.33%) | -14.75% (-45.33% to 47.61%) |
|  |  | YLDs | All age number | 3.1 (1.62 to 5.16) | 0.53 (0.21 to 1.04) | 2.57 (1.32 to 4.46) | 6.87 (3.88 to 10.68) | 1.69 (0.84 to 3.09) | 5.18 (2.87 to 8.06) | 121.41% (41.23% to 278.31%) | 215.95% (61.04% to 549.62%) | 101.76% (19.73% to 266.2%) |
|  |  |  | Age-standardized rate (per 100,000) | 0.72 (0.37 to 1.18) | 0.27 (0.11 to 0.52) | 1.14 (0.59 to 1.92) | 0.65 (0.37 to 1.02) | 0.31 (0.15 to 0.57) | 1.02 (0.56 to 1.57) | -8.91% (-41.43% to 52.37%) | 14.41% (-41.07% to 138.8%) | -10.16% (-44.97% to 62.09%) |
|  |  | YLLs | All age number | 365.98 (203.17 to 567.39) | 64.44 (27.98 to 124.13) | 301.54 (169.82 to 478.96) | 732.7 (471.44 to 1145.95) | 185.79 (100.21 to 322.23) | 546.91 (353.56 to 851.31) | 100.2% (30.6% to 233.13%) | 188.32% (45.55% to 498.43%) | 81.37% (15.05% to 219.03%) |
|  |  |  | Age-standardized rate (per 100,000) | 79.78 (44.45 to 123.79) | 30.14 (13.05 to 56.41) | 125.25 (71.22 to 194.9) | 67.62 (43.76 to 106.59) | 33.14 (17.8 to 57.82) | 104.41 (67.37 to 163.89) | -15.24% (-44.21% to 39.23%) | 9.97% (-43.47% to 127.94%) | -16.64% (-47.33% to 45.7%) |
|  | Metabolic risks | DALYs | All age number | 206.62 (86.63 to 326.68) | 105.21 (45.94 to 170.52) | 101.4 (38.79 to 172.73) | 1028.41 (366.79 to 1675.65) | 509.8 (179.68 to 842.25) | 518.62 (178.35 to 864.34) | 397.74% (255.51% to 540.13%) | 384.53% (203.33% to 590.73%) | 411.45% (247.44% to 633.3%) |
|  |  |  | Age-standardized rate (per 100,000) | 44.82 (17.9 to 72.23) | 47.56 (19.34 to 76.75) | 42.44 (15.63 to 72.32) | 96.15 (33.72 to 157.45) | 92.64 (32.11 to 153.73) | 100.19 (33.29 to 168.62) | 114.54% (56.63% to 173.85%) | 94.79% (24.68% to 174.69%) | 136.1% (62.42% to 234.09%) |
|  |  | Deaths | All age number | 6.88 (2.68 to 11.06) | 3.4 (1.38 to 5.58) | 3.48 (1.21 to 6.02) | 40.32 (13.74 to 67.11) | 19.78 (6.7 to 32.89) | 20.54 (6.59 to 35.08) | 486.0% (327.62% to 647.27%) | 480.88% (279.19% to 707.66%) | 491.01% (303.66% to 748.95%) |
|  |  |  | Age-standardized rate (per 100,000) | 1.76 (0.66 to 2.88) | 1.8 (0.7 to 2.97) | 1.73 (0.57 to 3.01) | 4.04 (1.35 to 6.76) | 3.79 (1.27 to 6.34) | 4.32 (1.35 to 7.41) | 129.94% (72.11% to 190.13%) | 110.98% (41.21% to 188.22%) | 149.06% (78.44% to 251.96%) |
|  |  | YLDs | All age number | 4.32 (1.4 to 7.62) | 2.6 (0.76 to 4.9) | 1.72 (0.63 to 3.17) | 36.11 (12.05 to 62.76) | 22.81 (7.21 to 41.61) | 13.3 (5.06 to 22.82) | 736.52% (495.77% to 1009.24%) | 777.14% (482.88% to 1176.35%) | 674.98% (419.56% to 1034.36%) |
|  |  |  | Age-standardized rate (per 100,000) | 0.98 (0.31 to 1.73) | 1.23 (0.35 to 2.29) | 0.76 (0.28 to 1.4) | 3.4 (1.1 to 5.92) | 4.17 (1.24 to 7.61) | 2.61 (0.98 to 4.53) | 248.64% (156.27% to 354.58%) | 238.25% (130.32% to 386.44%) | 243.81% (139.37% to 389.56%) |
|  |  | YLLs | All age number | 202.3 (85.48 to 320.41) | 102.61 (45.39 to 166.57) | 99.69 (38.08 to 169.43) | 992.3 (355.3 to 1620.32) | 486.99 (172.64 to 807.21) | 505.31 (173.82 to 844.94) | 390.51% (250.95% to 531.75%) | 374.58% (197.55% to 576.33%) | 406.91% (243.98% to 628.67%) |
|  |  |  | Age-standardized rate (per 100,000) | 43.84 (17.62 to 70.59) | 46.32 (19.02 to 74.6) | 41.68 (15.32 to 70.93) | 92.75 (32.68 to 152.08) | 88.47 (30.87 to 146.63) | 97.58 (32.42 to 164.93) | 111.55% (54.51% to 170.6%) | 90.97% (21.92% to 169.37%) | 134.14% (61.14% to 231.33%) |

**S3 Table Footnote:** DALYs: Disability-Adjusted Life Years. YLDs: Years Lived with Disability. YLLs: Years of Life Lost.
