# Supplementary material for: Temporal trend in the national and sub-national burden of cancers attributable to risk factors in Iran from 1990 to 2021: Findings from the global burden of disease study 2021
Source: PLoS One. 2025 Aug 26;20(8):e0330993. doi: 10.1371/journal.pone.0330993 (PMC12380304; doi:10.1371/journal.pone.0330993)

**Temporal trend in the national and sub-national burden of cancers attributable to risk factors in Iran from 1990 to 2021: findings from the Global Burden of Disease Study 2021**

Seyede Maryam Mousavi^1, 2^¶, Sobhan Younesian^1,2^¶, Saba Katebian^1^, Ali Golestani^1^, Shaghayegh Khanmohammadi^1,3^, Sepehr Khosravi^1^, Yasaman Etemadi^1^, Nazila Rezaei^1^, Sina Azadnajafabad^1*^, Bagher Larijani^4*^

**Authors’ affiliations:**

1. **Non-Communicable Diseases Research Center, Endocrinology and Metabolism Population Sciences Institute, Tehran University of Medical Sciences, Tehran, Iran**
2. **School of Medicine, Tehran University of Medical Sciences, Tehran, Iran**
3. **Research Center for Immunodeficiencies, Pediatrics Center of Excellence, Children’s Medical Center, Tehran University of Medical Sciences, Tehran, Iran**
4. **Endocrinology and Metabolism Research Center, Endocrinology and Metabolism Clinical Sciences Institute, Tehran University of Medical Sciences, Tehran, Iran**

***Corresponding authors:**

Sina Azadnajafabad (E-mail: [sina.azad.u@gmail.com](mailto:sina.azad.u@gmail.com))

Bagher Larijani (E-mail: [emrc@tums.ac.ir](mailto:emrc@tums.ac.ir))

¶ These authors contributed equally to this work.

Supplementary methods and results to “Temporal trend in the national and sub-national burden of cancers attributable to risk factors in Iran from 1990 to 2021: findings from the Global Burden of Disease Study 2021”

**S1 Figure Caption:** Trends of age-standardized DALY rates of cancer attributable to all and level 2 risk factors by gender in Iran from 1990 to 2021.


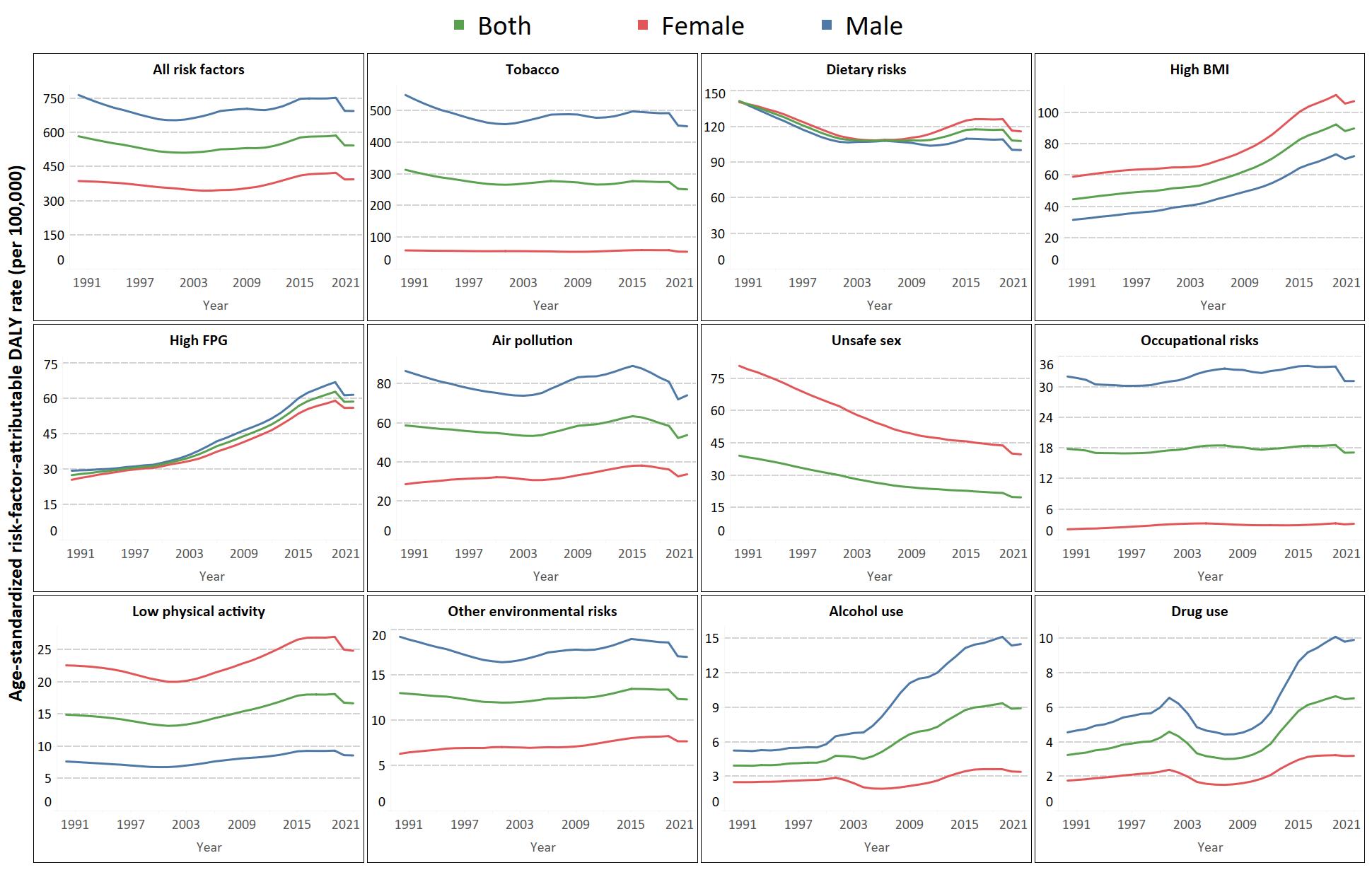

Supplement: S1 Fig — (DOCX) [file pone.0330993.s004.docx]
