# Supplementary material for: Temporal trend in the national and sub-national burden of cancers attributable to risk factors in Iran from 1990 to 2021: Findings from the global burden of disease study 2021
Source: PLoS One. 2025 Aug 26;20(8):e0330993. doi: 10.1371/journal.pone.0330993 (PMC12380304; doi:10.1371/journal.pone.0330993)
Supplement: Attachment — Submitted filename: Response to Reviewers.docx [file pone.0330993.s010.docx]

Dear Editorial Team and Esteemed Reviewers,

We sincerely thank the editors and reviewers for their valuable comments and insightful suggestions, which have significantly improved our manuscript both methodologically and in terms of presenting our findings. Your constructive feedback allowed us to refine critical aspects of the study, including the refinement of the Introduction, the clarification and strengthening of the Methods, improvements in the figures, and enhancement of the Discussion. We have provided a detailed response to each comment in the following sections of this letter. Additionally, we have revised the manuscript using tracked changes for your convenience. We hope these updates meet your expectations and facilitate the review process. Thank you once again for your thoughtful and meticulous review.

**Reviewer #1 comments:**
**Comment:** Figure 5 shows quantification of DALY and death rates for ovarian cancer in males. On top of that this quantification is negative for dietary risks. As ovarian cancers do not happen in males (and would not be significantly detectable even including trans-men) this is likely an artifact of the model used which should be corrected.

**Response:** We sincerely thank the reviewer for carefully pointing out this issue. The problem with Figure 5 was a mislabeling in the legend. We have recreated the figure to correct this error. The estimated DALY and death rates for prostate cancer attributable to dietary risks in males are negative in the dataset.

**Comment:** While the data used for the analysis is publicly available, the code used to perform it is not which prevents the evaluation of the correctness of the analysis. Please make the code available with the manuscript.

**Response:** Thanks for your comment! The code used to perform all estimations used in this manuscript are publicly available at <https://github.com/ihmeuw/ihme-modeling/tree/main/gbd_2021>. We have also added the link to the manuscript, emphasizing that the code is also publicly available. We have also clearly stated the version of R (version 4.4.0) and Python (version 3.12.4) used and specified the date on which the GBD data were accessed (October 20, 2024) in the manuscript. Additionally, we would like to clarify that Tableau, which was used for some data visualizations, does not require coding scripts. Other visualizations were generated using standard procedures from the ggplot2 package in R and pandas library in Python, and LOESS regression was implemented using base R function [loess (val ~ sdi)].

**Comment:** Typos: COIVD

**Response:** Thanks! We have corrected this fault in the text.

**Comment:**  The text of some cells in Table 1 is truncated (e.g. all DALY cells). Please extend the cells to fix it.

**Response:** Thanks for noticing that. We have extended the cells vertically and horizontally so that the whole UI interval can now be seen.

**Comment:** I would like to see a discussion of the decrease in YLL but increase in YLD of behavioral risks seen in Table 1.

**Response:** Thanks for your suggestion. In response we have a added a discussion of this observation to the Discussion section in the revised manuscript, which reads: “Since 1990, there has been a reduction in age-standardized rate of cancer YLLs attributed to behavioral risks, while YLDs have increased. This observation might be derived by advances in cancer survival in Iran over time due to enhanced therapies and increased healthcare access. Also, the survival rates of some leading cancers associated with behavioral risks, including breast cancer, have improved in Iran, although it is still lower than developed countries.”

**Reviewer #2 comments:**

**Comment 1:** It is not clear how the study is different than reframing the GBD data. The authors need to highlight how their study adds novel insights rather than just GBD.

**Response:** We appreciate your thoughtful feedback. We respectfully contend that our study offers substantial value beyond just re-reporting GBD data and contributes to understanding the cancer burden attributable to risk factors in Iran at both national and subnational levels. Here, we outline the key aspects that distinguish our work and underscore its significance. First, Iran currently lacks sufficient peer-reviewed publications comprehensively reporting the burden of cancers attributable to risk factors, particularly at the subnational level. Our study addresses this gap by providing a detailed analysis of cancer types and attributable risk factors across Iran’s provinces from 1990 to 2021. This will inform localized public health policies, which are often constrained by the absence of such data in the Iranian context.

Moreover, our study enhances the utility of GBD data by developing original visualizations that elucidate temporal patterns and spatial variations in risk-attributable cancer burden over 32 years. These visualizations, tailored to highlight trends and patterns, offer an accessible and exploratory tool for policymakers, extending beyond the raw data outputs typically provided by GBD. Including such figures represents a novel contribution, enabling a deeper interpretation of the data not readily available in the GBD database.

Also, you’re right that our initial analysis did not include heavy statistical methods. We have since incorporated Locally Estimated Scatterplot Smoothing (LOESS) regression analysis to examine the relationship between SDI and the percentage of attributable cancer DALYs for top five risk factors at the subnational level. We have also employed the annualized rate of change (ARC) analysis to enhance the interpretation of data. The ARC analysis assessed the relationship between the annual change in summary exposure values (SEV) for specific risk factors and the corresponding annual change in cancer disability-adjusted life years (DALYs) attributable to those risk factors at the subnational level, with estimates derived for the period 1990 to 2021. The findings from this analysis are illustrated in Figure 6, which highlights the top four risk factors and categorizes subnational regions by SDI quintiles, providing deeper insights into the underlying drivers of these trends. Additionally, the LOESS regression modeled the non-linear association between SDI and the percentage of attributable DALYs for specific risk factors, with results presented in Figure 7. This analysis, incorporating data from all provinces over the 1990–2021 period, reveals that the proportion of cancer DALYs attributed to high body-mass index and high fasting plasma glucose increases with rising SDI values. These novel findings elucidate the relationship between SDI and the contribution of risk factors to cancer burden. Therefore, our study includes several analyses.

Finally, our study's originality lies in its contextual relevance and synthesis. By focusing on Iran, a country with a unique demographic, epidemiological, and socio-economic profile, we critically adapt global data to a national and subnational framework. This localized perspective is vital for translating global estimates into actionable insights, a step that GBD alone cannot achieve without region-specific interpretation and analysis.

We believe that these contributions, including filling a literature gap, enhancing data through visualizations, and applying statistical methods, demonstrate that our study is far more than a re-reporting of GBD data. Instead, it represents a valuable extension that supports evidence-based decision-making in Iran’s public health landscape. We hope this clarification addresses your concerns and reinforces the merit of our work.

**Comment 2:** The methods section is basically re-writing the GBD methods. No information about what “authors” have done; version? Which R scripts? Date accession? This will ensure reproducibility.

**Response:** Thanks for pointing out this issue. You are correct that the initial version of the Methods section devoted considerable space to describing the GBD 2021 methodology, with relatively limited emphasis on our own analytical procedures. In response, we have revised the section to provide a more concise summary of the GBD methodology and have placed greater focus on the analyses we conducted, including the calculation of the annualized rate of change (ARC) and the application of LOESS regression. We have also clearly stated the version of R (version 4.4.0) used and specified the date on which the GBD data were accessed (October 20, 2024) in the manuscript. Additionally, we would like to clarify that Tableau, which was used for some data visualizations, does not require coding scripts. Other visualizations were generated using standard procedures from the ggplot2 package, and LOESS regression was implemented using base R function [loess (val ~ sdi)]. The results of LOESS analysis are depicted in the Figure 7, which has been newly added to our manuscript in the revised version and is shown below:

**Comment 3:** How local MIRs were derived

**Response:** Thanks for your question. We’ll provide relatively detailed answer here based on the GBD 2021 methodology documentation. However, we have also added a few sentences clarifying your question in the manuscript. Accordingly, cancer incidence and mortality data from registries were matched by cancer type, age, sex, year, and location to calculate mortality-to-incidence ratios (MIRs), which were used as inputs for further modeling. For most cancers, MIRs were estimated using a three-step spatiotemporal Gaussian process regression (ST-GPR) approach, where logit-transformed MIRs were modeled with covariates including sex, categorical age groups, and the Healthcare Access and Quality (HAQ) Index in a linear mixed-effects model, followed by spatiotemporal smoothing and Gaussian process regression. The ST-GPR model employed fixed hyperparameters (lambda = 0.05 for time, omega = 0.5 for age, zeta = 0.01 for geography, amplitude = 1, and scale = 10) to smooth data across dimensions, with manual outlier removal for unrealistic data points. For rare cancers in young age groups, data were aggregated to the youngest five-year age bin with at least 50 cases from 1990–2015 SEER data, with MIRs applied to younger age groups. MIRs were capped at the 95th percentile by age group to allow values above 1, with pediatric age groups (under 20) capped at 1 for flexibility; values exceeding caps were Winsorised, and inputs were scaled for ST-GPR and rescaled post-modeling, with lower caps at the fifth percentile to constrain underestimates. This methodology ensures robust and flexible MIR estimates across diverse cancers and demographics.

We have briefly explained this in the Methods section: “MIRs were estimated using cancer registry data matched by cancer type, age, sex, year, and location. A spatiotemporal Gaussian process regression (ST-GPR) model incorporated covariates such as age, sex, and the Healthcare Access and Quality Index, with smoothing across time, age, and geography. Additionally, adjustments were made for rare cancers and outliers to ensure reliable estimates across all cancer types and demographic groups.”

**Comment 4:**  The introduction reads like separate paragraphs that are not linked to each other. It needs to be tied around one idea and needs to flow seamlessly.

**Response:** Many thanks for your valuable insight! We have revised and reframed the introduction section to improve the coherence between paragraphs. Changes can be tracked in the “Revised Manuscript with Track Changes” file.

**Comment 5:** There are differences between ecological modeling and causation. The authors have to be very clear when saying that this risk factor cause X DALYs

**Response:** Thanks for pointing out this important consideration! We have explicitly avoided the use of the word “caused by” while referring to the cancer burden attributed to risk factors and have used the word “attributed to” instead. There was one instance in the manuscript that we wrote “cancer DALYs due to metabolic risks”, which we have now edited and replaced it with “cancer DALYs attributed to metabolic risks”. It is also worth mentioning that in order to be consistent with the GBD terminology, we have used the term “causes of death” to refer to the cancer types, as this is the standard GBD terminology.

**Comment 6:** Have the authors applied FDR in their statistical analysis for multiple comparisons?

**Response:** Thanks for your insightful question! Multiple comparisons are a major issue when the model involves many statistical tests and thus multiple comparisons, as this is the case in our work. In order to provide an answer, we should delve deeper into the GBD 2021 methodology. GBD 2021 relies on Bayesian hierarchical models, meta-regression (e.g., MR-BR), and ensemble modeling (e.g., DisMod-MR) rather than FDR for handling multiple comparisons. These methods focus on uncertainty intervals (UIs) from Bayesian posterior distributions or bootstrap methods to quantify variability. Therefore, FDR is not applied, but this aligns with the study’s reliance on Bayesian methods, which inherently handle multiple comparisons through hierarchical modeling and uncertainty quantification rather than p-value adjustments. For instance, in estimating risk factor-attributable burden, mediated-adjusted population attributable fractions (PAFs) were used to account for correlations between risk factors, suggesting a different approach to managing multiple testing issues than FDR.

**Comment 7:** In figure 3 the x axis min and max are large making it hard to see the differences and the intervals

**Response:** Thanks for your helpful feedback. We had used fixed scale for the Figure 3, which made it difficult for some values to be seen. We have now recreated the Figure 3 and used free scales in the x-axis for each risk factor. Now the percent changes and intervals can be more easily observed. Given that the X-axis scale is now tailored to each risk factor rather than being uniform across all graphs, it is important to interpret comparisons between risk factors with caution, as the differing scales may affect visual interpretation. Of note, because we now have added a spatial map plot, the name of the figure we are discussing in this comment has changed to Figure 4 in the revised manuscript.

**Comment 8:** Figure 4 have abbreviations that I assume they are countries names. But no reader will be familiar of all these abbreviations

**Response:** You are absolutely right in highlighting this issue. These abbreviations stand for the names of Iran’s provinces. As these names are too long, it was not possible to include the full names of the provinces in the figure. We have added the explanation for these abbreviations in the caption of the Figure 3.

**Comment 9:** The colors in Figure 5 are very similar to each other for example, bladder cancer and uterine cancer are both having the same shade of blue.

**Response:** Thanks for your valuable feedback. We have recreated the Figure 5, utilizing more distinct colors to improve clarity.

**Comment 10:** There are no mention of study limitations and GBD limitations at all in the discussion. This is very crucial.

**Response:** We truly appreciate your attention to this crucial point. We have added a comprehensive paragraph discussing the study’s limitations and GBD limitations at the end of the Discussion section in the manuscript, which reads:

“This study is the first to assess the temporal trends in cancer burden attributable to risk factors over a three-decade interval, utilizing the most updated GBD data. Nevertheless, this study inherits limitations of the GBD methodology. GBD estimates rely heavily on statistical modeling using national input data when available. However, high-quality and updated data, mostly from Iran National Cancer Registry, may not be frequently incorporated into the GBD estimation methods. While subnational estimates were provided in this study, they might be biased in provinces with sparse data, affecting the accuracy and relevance of the subnational estimations. Another limitation is that the GBD 2021 utilizes universal relative risks for cancer risk factors, which may reduce the reliability of the attributable burden estimates specific to the Iranian population. In addition, the PAFs and cancer burden were estimated in the same year, disregarding the time required for a risk factor to contribute to cancer. The sub-classifications of risk factors and cancer types in the GBD 2021 data are not comprehensive, limiting the ability to include pathologically distinct cancer subtypes and to adequately represent etiologically significant risk factors, such as viral infections. Of note, this study captured trends until 2021, not accounting for changes in risk factor-attributable cancer burden during the post-pandemic era.”

**Comment 11:** How was percent change was calculated because there are no UIs in table 1?

**Response:** We appreciate your insightful comment. The Table 1 in .docx format did include UIs for percent changes. We have extended the cells both horizontally and vertically so that the whole UI interval can now be seen. Percent changes were estimated as the relative difference between estimates for two years, such as:

$$Percent cahnge: \frac{{Estimate}_{t2}- {Estimate}_{t1}}{{Estimate}_{t1}}$$

Uncertainty intervals were estimated using Bayesian methods: 1000 draws from the posterior distribution were generated, accounting for data variability and modeling choices. For percent change, these draws were paired across years, and the 95% UI was determined by the 25^th^ and 975^th^ values of the ordered percent change calculations, representing the 2.5^th^ and the 97.5^th^ percentiles.

We have also provided this explanation in the manuscript text: “The 95% uncertainty interval (UI) for each metric was calculated using the 2.5th and 97.5th percentiles from the uncertainty distribution, based on 1000 draws from the posterior distribution”

**Comment 12:** Analyzing the subnational level data is very important so it would benefit from exploring the reasons behind these differences. A map with matrix plot like colors for showing the differences in risk factors and DALYs would be beneficial to the manuscript (spatial map)

**Response:** Thanks for your insightful suggestion. In response, we have created subnational-level spatial plots illustrating the age-standardized DALY rates attributable to all and the top-five risk factors. These plots have a matrix plot-like color gradient to clearly depict regional variations. We have designated them as Figure 3 in the revised manuscript. This figure aims to provide a clearer understanding of the patterns in geographical and risk-specific cancer DALY rates, in alignment with your valuable suggestion. We have also discussed these geographical patterns in risk-attributable cancer burden, specifically the pattern of tobacco-attributable cancer burden, in the Discussion section.

**Comment 13:** Why the authors attribute the reduction to COVID19?

**Response:** The observed decline in cancer deaths and DALYs during the COVID-19 pandemic can be explained by several factors, such as:

1. Under diagnosis and reduced healthcare utilization: During the pandemic, there was a significant decrease in outpatient visits and cancer screenings in Iran due to patients' fear of contracting COVID-19, lockdowns, and healthcare system disruptions. This led to fewer cancer diagnoses being recorded, which can artificially reduce the reported cancer burden and related DALYs in the short term. Essentially, many cancer cases went undetected or were diagnosed later, which temporarily lowered the recorded cancer mortality and morbidity figures.
2. Delayed reporting and misclassification: Some cancer patients who died during the pandemic may have been misclassified as COVID-19 deaths or their deaths may have been underreported due to the overwhelmed health systems. This can distort mortality statistics, leading to an apparent decline in cancer-related DALYs.
3. Data collection and reporting limitations: The pandemic disrupted routine data collection and reporting systems, which may have led to incomplete or delayed cancer mortality data, thus affecting DALY estimates.

We have briefly discussed these possible underlying factors in the Discussion section: “The net reduction in risk-attributable age-standardized DALY and death rates may have resulted from their major reduction in 2020, likely resulting from the COVID-19 pandemic effect on delayed cancer screening and diagnosis. During the COVID-19 pandemic, many cancer screening programs were interrupted, primarily because of lockdown measures and the requirement to reallocate healthcare resources. In addition, a reduced quality of data collection and reporting, including possible misclassification of the cause of death, might had resulted in underestimation of cancer burden in Iran throughout the pandemic.”

**Comment 14:** The discussion would benefit from comparing Iran to the other similar countries to should the similarities and the differences

**Response:** We are grateful for your helpful suggestion. In the revised manuscript, we have pointed out to the differences and similarities between Iran and other countries in terms of risk-attributable cancer burden and policy strategies in many instances. Below, I’ll rewrite examples from the revised version of our manuscript:

1. “Iran shares its leading cancer risk factors, namely tobacco smoking, dietary risks, and obesity, with many other countries in the North Africa and Middle East (NAME) region.”
2. “The global age-standardized cancer DALY rates attributable to high FPG and high BMI increased by less than a fifth from 1990 to 2019; nevertheless, the age-standardized cancer DALY rate in Iran for high FPG and high BMI each increased over twofold, indicating that Iran is facing a greater surge than the global average.”
3. “This is while other countries in the Eastern Mediterranean region, such as Saudi Arabia, have successfully reduced SSB consumption through taxation. Iran’s lower taxation rates, lack of consistent enforcement, and inefficient allocation of tax revenues have limited the effectiveness of its SSB taxation program compared to Saudi Arabia. In order to overcome these, implementing a strongly enforced higher taxation rate and redirecting tax revenues toward subsidizing fruits and vegetables are strongly recommended policy options in Iran’s context.”
4. “Therefore, Iran will benefit from emulating prosperous countries' media policies to reduce tobacco smoking prevalence. Brazil, an outstanding model case in combating tobacco smoking, is the leading country in tobacco use prevalence reduction; Brazil’s anti-tobacco media policies include banning tobacco advertisement except at enclosed points of sale and prohibiting tobacco brands' sponsorship of public events.”

**Comment 15:** Exploring the reasons behind this surge in opium use is important in the discussion

**Response:** Thanks for your valuable insight. In response, we have added the following sentences in the manuscript to explore the reasons behind opium surge in Iranian males: “The surge in opium use among Iranian males can be attributed to a combination of factors, including longstanding cultural tolerance of opium and socioeconomic deprivation, such as unemployment and low education. Also, Iran’s geopolitical position along the drug trafficking routes and domestic economic instability further contribute to widespread availability and use.”

**Comment 16:** Table one seems to have been cut in the first couple of rows (the upper UI is not found.) for example all age numbers 169057.47 (135238.74 to )

**Response:** Thanks for noticing that. We have extended the cells vertically and horizontally so that the whole UI interval can now be seen.

**Comment 17:** Risk-attributable cancer burden in Iran had an overall upward trend” contradicts earlier claim that ASDR declined

**Response:** Thanks for your attention to these two important sentences. While they may initially appear contradictory, they are, in fact, complementary. As noted in the Results section and illustrated in S1 Figure, the ASDR attributable to risk factors exhibited an overall upward trend over most of the study period. However, when considering the net change between 1990 and 2021, a decline is observed, primarily driven by a sharp reduction in 2020. To minimize potential confusion, we have revised the sentences as follows:

“Although age-standardized death and DALY rates attributable to risk factors declined between 1990 and 2021, both rates displayed an upward trend throughout most of the intervening years. The net reduction in risk-attributable age-standardized DALY and death rates may largely be explained by their substantial decline in 2020, potentially linked to the COVID-19 pandemic’s impact on delayed cancer screening and diagnosis.”

**Comment 18:** The discussion section is overwhelming while it does not have a deep, evidence-based discussion linking findings to actionable policies.

**Response:** We truly appreciate your feedback. We have thoroughly revised the Discussion section. Along with addressing the other comments of yours regarding the Discussion section, including discussing opium use surge and comparing Iran to other countries, we have also added a range of policy intervention suggestions in the Discussion section. Below, I’ll highlight some examples of of evidence-based actionable policies we have recommended in the manuscript:

1. “Therefore, the rising impact of high BMI and high FPG as significant risk factors on cancer burden in Iran demands urgent attention and policy intervention. Iran enacted the Sugar-Sweetened Beverages (SSBs) taxation program in 2013, which was revised in 2021, imposing a 16% and 36% tax on domestic and imported SSBs, respectively; however, household expenditure on SSBs has not declined in Iran despite taxation. This is while other countries in the Eastern Mediterranean region, such as Saudi Arabia, have successfully reduced SSB consumption through taxation. Iran’s lower taxation rates, lack of consistent enforcement, and inefficient allocation of tax revenues have limited the effectiveness of its SSB taxation program compared to Saudi Arabia. In order to overcome these, implementing a strongly enforced higher taxation rate and redirecting tax revenues toward subsidizing fruits and vegetables are strongly recommended policy options in Iran’s context.”
2. “Therefore, Iran will benefit from emulating prosperous countries' media policies to reduce tobacco smoking prevalence. Brazil, an outstanding model case in combating tobacco smoking, is the leading country in tobacco use prevalence reduction. Brazil’s anti-tobacco media policies include banning tobacco advertisement except at enclosed points of sale and prohibiting tobacco brands' sponsorship of public events.”
3. “Importantly, the cancer burden is projected to continuously grow in Golestan province, calling for policy interventions, including risk factor prevention and screening programs, to mitigate cancer incidence in this high-risk area.”
4. Hence, the top three cancers with the highest increase in risk-attributable DALY rate shared high BMI as their primary risk factor, which further raises concern about the increasing trend of high BMI-attributable cancer burden in the country. Effective policy interventions to reduce BMI among Iranian adults should focus on integrating primary healthcare services for population-level weight management and reducing inequalities in healthy lifestyle behaviors.
